# Quantifying Crustal Thickness in Continental Collisional Belts: Global Perspective and a Geologic Application

**DOI:** 10.1038/s41598-017-07849-7

**Published:** 2017-08-01

**Authors:** Fangyang Hu, Mihai N. Ducea, Shuwen Liu, James B. Chapman

**Affiliations:** 1 Key Laboratory of Orogenic Belts and Crustal Evolution, Ministry of Education, Peking University, Beijing, 100871 P.R. China; 2 Department of Geosciences, University of Arizona, 1040 E. 4th Street, Tucson, Arizona 85721 USA; 3 Faculty of Geology and Geophysics, University of Bucharest, 010041 Bucharest, Romania

## Abstract

We present compiled geochemical data of young (mostly Pliocene-present) intermediate magmatic rocks from continental collisional belts and correlations between their whole-rock Sr/Y and La/Yb ratios and modern crustal thickness. These correlations, which are similar to those obtained from subduction-related magmatic arcs, confirm that geochemistry can be used to track changes of crustal thickness changes in ancient collisional belts. Using these results, we investigate temporal variations of crustal thickness in the Qinling Orogenic Belt in mainland China. Our results suggest that crustal thickness remained constant in the North Qinling Belt (~45–55 km) during the Triassic to Jurassic but fluctuates in the South Qinling Belt, corresponding to independently determined tectonic changes. In the South Qinling Belt, crustal thickening began at ~240 Ma and culminated with 60–70-km-thick crust at ~215 Ma. Then crustal thickness decreased to ~45 km at ~200 Ma and remained the same to the present. We propose that coupled use of Sr/Y and La/Yb is a feasible method for reconstructing crustal thickness through time in continental collisional belts. The combination of the empirical relationship in this study with that from subduction-related arcs can provide the crustal thickness evolution of an orogen from oceanic subduction to continental collision.

## Introduction

Present day thickness of the continental crust is relatively well known at regional and global scales and ranges from just a few kilometers thicker than oceanic crust (~6–10 km) to over 80 km at some convergent margins, such as in Himalaya-Tibet^1^. In consideration of continental crust is one of the most unique product on Earth, recognizing variations in crustal thickness is fundamental to understanding tectonic and geodynamic processes, especially for tracking the switch of tectonic setting and development of the lithosphere of Earth at the moment. Monitoring the crustal thickness throughout the geologic time could also provide valuable information for continental crustal growth because the crustal growth in the vertical direction is significant for continental collisional belts. While it is difficult to quantify past crustal thickness changes throughout the geological record, one approach is the use of geochemical information from magmatic rocks as proxies for paleo-crustal thickness^2–9^.

For example, the ratio of Sr/Y and La/Yb in modern intermediate composition volcanic rocks in subduction-related arcs appear to correlate well with crustal thickness at global and regional scales^5–7^. During fractionation and differentiation in deep crustal environments (>~1.0 GPa), Y and Yb will preferentially incorporate into cumulate garnet or amphibole, but Sr and La will enter the liquid phase, resulting in high Sr/Y and La/Yb ratios. In contrast, in shallow crustal environments (<~1.0 GPa), Sr will preferentially partition into plagioclase, whereas Y and Yb enter the liquid phase, which leads to low Sr/Y and La/Yb ratios. In addition, the crustal assimilation at shallow level in the crust will also lower Sr/Y and La/Yb ratios^5, 7^. The relationship between Sr/Y, La/Yb, and crustal thickness are based entirely on studies of subduction-related arcs^5, 7^ and draw from models that infer magmatic differentiation via assimilation and fractional crystallization (AFC) processes near the base of the crust in subduction-related arcs^10–14^. A critical question to ask moving beyond the studies conducted so far is whether the correlations between trace elements and crustal thickness can be applied to other tectonic settings. This paper examines compositions of magmas in continental collisional orogens as they may correlate with crustal thickness.

Continental collisional orogens evolve from subduction margins. For example, prior to the collision of India and Asia, Tibet was a subduction margin marking the consumption of Neo-Tethys oceanic lithosphere and had a long lived continental magmatic arc on the upper plate^15, 16^. Several studies have qualitatively applied Sr/Y and La/Yb ratios from syn-collisional magmatic rocks to evaluate past crustal thickness in Tibet^17, 18^ and recently Zhu *et al*. (2017)^19^ applied the La/Yb correlation from Profeta *et al*. (2015)^7^ to quantitatively track changes in crustal thickness in Tibet before and after India-Asia collision. The study by Zhu *et al*. (2017)^19^ is consistent with independent geologic evidence including stable isotopic studies^20–23^. The empirical relationship between La/Yb, Sr/Y, and crustal thickness for places like Tibet can be improved by recalibrating it to collisional orogens rather than continental arcs.

Here we explore the relationship between geochemical features of rocks and crustal thickness using previously published intermediate magmatic rocks from six continental collisional belts (Supplementary Information). Some of the areas investigated are formed in a “grey” area of syn-collision to post-collisional settings (e.g., the Carpathians^24^), which often include intra-plate extension related to gravitational collapse^14–19, 24^. Collision-related magmatism is volumetrically not as abundant as arc-related magmatism, and unfortunately few collisional belts are active on the planet nowadays. The scarcity of collisional settings compared to subduction margins forced us to investigate data of a slightly wider age range - Pliocene to modern (Middle Miocene for special cases), as opposed to only Quaternary rocks^5–7^.

To evaluate our new calibration of La/Yb and Sr/Y in continental collisional orogens, we examine an ancient collisional orogen, the Qinling Orogenic Belt (QOB) in central China. The QOB was mainly formed by continental collision between the North China Craton (NCC) and South China Block (SCB) during the Triassic and then the QOB evolved into an intracontinental collisional orogen during the Jurassic. Collisional-related granitoids are prevalent throughout the QOB, making it an excellent area to test and apply this method.

## Geological background

### Global continental collisional orogens

We compiled major and trace element data on Miocene and younger intrusive and extrusive rocks from continental collisional orogens establishing empirical relationships between geochemical indices and Moho depth or crustal thickness (Supplementary Information). The compiled data are from the following continental collisional orogens: Eastern Anatolia–Central Anatolia; Eastern Carpathians–Apuseni Mountains; Greater Caucasus, Lesser Caucasus; Northwestern Iran–Northern Iran, and Northern Tibet–Southern Tibet.

All of these collisional belts represent various segments along the Alpine–Himalayan orogen, which records the closure of the Tethys Ocean and related basins closed at various times during the Cenozoic^25^. All of the data complied here are Pliocene or younger except for data from southern Tibet, which were emplaced or erupted during Middle Miocene (Supplementary Information). While each area examined has regional geologic complexity associated with it, all of the areas were formed in compressional settings and were demonstrably not related to oceanic subduction. Previous regional studies have proposed that some of these areas are experiencing post-collisional extension and/or gravitational collapse, although most of these regions are in an overall compressional regime^24, 26–29^.

### Qinling Orogenic Belt

**Figure 1:**
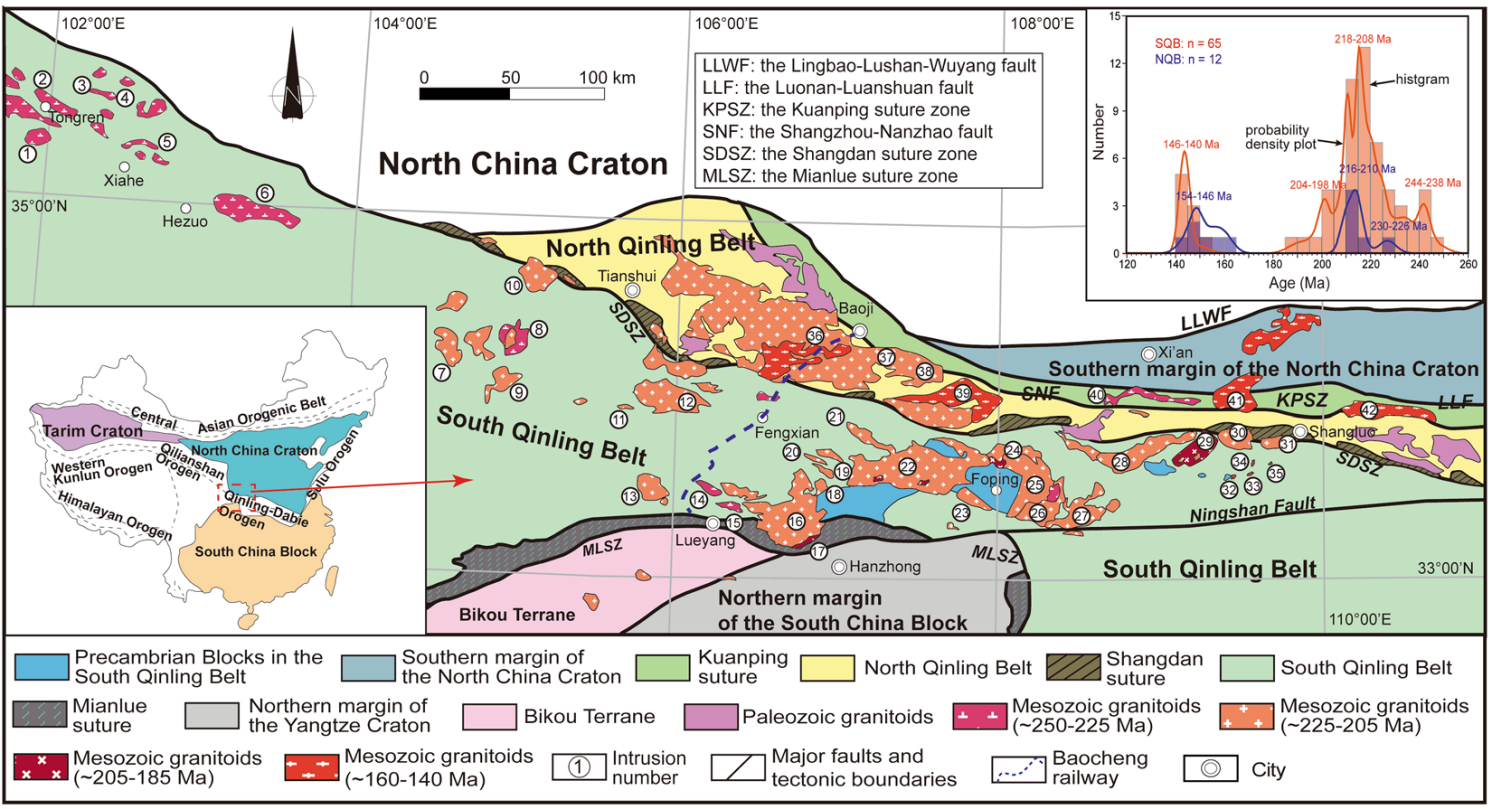
Simplified geological map showing the distribution of tectonic units, sedimentary sequences, and the early Mesozoic granitoid rocks in the Qinling Orogenic Belt (modified from Dong and Santosh, 2016^30^ and Li *et al*., 2013^37^). The blue dashed line (Baoji-Chengdu railway) separates the eastern and western Qinling Orogenic Belt^31^. The inset at the top right corner is a histogram of crystallization ages (Ma) and a probability distribution plot of the granitoid intrusions from the Qinling Orogenic Belt. To minimize sampling bias, only one age datum is selected for each pluton if between-sample age difference is lower than 3 Myr. SQB = South Qinling Belt, NQB = North Qinling Belt. The inset at the bottom left corner is a schematic map showing the North China Craton, South China Craton, Tarim Craton, and major orogenic belts in China (modified from Hu *et al*., 2016^32^). The plotted data are listed in Supplementary Information. Numbers for intrusions: 1-Maixiu; 2-Tongren; 3-Shuangpenxi; 4-Xiekeng; 5-Xiahe; 6-Meiwu; 7-Lüjing; 8-Zhongchuang; 9-Luchuba; 10-Wenquan; 11-Huangzhuguan; 12-Mishuling; 13-Miba; 14-Xinyuan; 15-Zhangjiaba; 16-Guangtoushan; 17-Erdaohexiang; 18-Huoshaodian; 19-Liuba; 20-Taoyuanpu; 21-Xiba; 22-Huayang; 23-Xichahe; 24-Longcaoping; 25-Wulong; 26-Laocheng; 27-Yanzhiba; 28-Dongjiangkou; 29-Zhashui; 30-Caoping; 31-Shahewan; 32-Lengshuigou; 33-Baishagou + Chigou + Tudigou + Shuangyuangou; 34-Xiaohekou + Wagou + Yuanjiagou; 35-Xiaguanfang + Yuanzijie; 36-Baoji; 37-Laojunshan; 38-Qinlingliang; 39-Taibai; 40-Cuihuashan; 41-Muhuguan; 42-Mangling.

The Qinling Orogenic Belt (QOB) is one of the most important ancient continental collisional orogens in East Asia (Fig. 1). It is located between the North China Craton (NCC) and South China Block (SCB), connecting the Qilian-Kunlun Orogens to the west and Dabie-Sulu Orogens to the east^30^ (Fig. 1). The QOB is subdivided into four tectonic domains separated by three well-documented sutures: the southern margin of the NCC, North Qinling Belt (NQB), South Qinling Belt (SQB), and northern margin of the SCB, respectively^30^ (Fig. 1). Zhang *et al*. (2001)^31^ separated the eastern and western QOB based on the Baocheng railway (Fig. 1).

The Qinling Orogenic Belt has experienced multiple phases of subduction-collision evolution since Neoproterozoic^30, 32, 33^. Most granitoid intrusions preserved in the QOB are formed during ~250–140 Ma related to Triassic subduction-collision and Jurassic-Cretaceous intracontinental collision processes^30, 32, 33^. Widespread ~250–140 Ma granitoid intrusions in the QOB provide targets to study variations of crustal thickness during collision^30, 32–36^ (Fig. 1; Supplementary Information). Relative to the SQB, the magmatism in the NQB is scarcer, but the timing of magmatism in these two areas is similar (Fig. 1). Early to Middle Triassic (~250–240) magmatism in the QOB is thought to be arc-related and is located mainly in the western QOB^30, 32, 37^. Previous studies proposed that the collision between the NCC and SCB commenced during the Middle to Late Triassic (~240–230 Ma), based on the age of ultrahigh pressure eclogite in the adjacent Dabie Orogen and paleomagnetic data^38, 39^. Most of magmatic rocks in the QOB were generated during the Late Triassic and is thought to be related to post-collisional slab break-off or delamination of lower crust^30, 32, 33^ (Fig. 1). After the Triassic, the QOB evolved into an intracontinental collisional belt during the Jurassic and minor granitoid magmatism was generated during the Late Jurassic to Cretaceous^30, 33^. We compiled geochemical data for ~250–140 Ma granitoid intrusions located in the NQB and SQB, to study the changes in crustal thickness during collisional process.

## Results

### Global correlations and its limits

**Figure 2:**
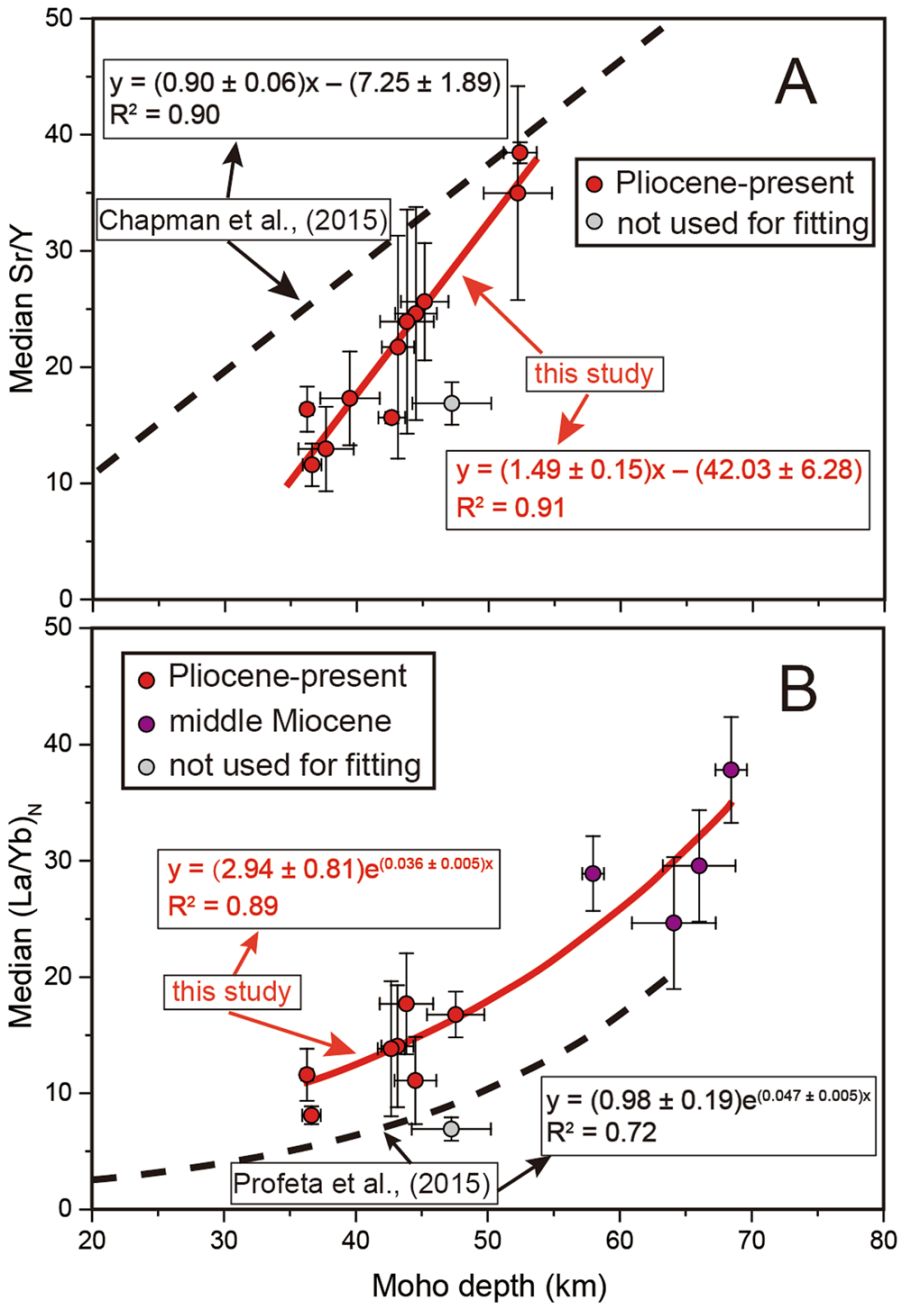
Global correlations between geophysically determined crustal depth (CRUST 1.0) and median Sr/Y (**A**) and median (La/Yb)_N_ (**B**) from continental collisional belts. The red circles represent the rocks formed during Pliocene to present and purple circles represent the rocks formed at Middle Miocene. The grey circles represent data subset from continental collisional belts but not used for calculating correlation equations. Median Sr/Y and (La/Yb)_N_ was calculated after filtration and discard of outliers; see text for detailed methods. Regression line and empirical relationship with R^2^ are shown on each diagram. The red solid lines represent the results of this study and the black dash lines represent the result of study on subduction arcs by Chapman *et al*. (2015)^5^ and Profeta *et al*. (2015)^7^. The plotted data are listed in Supplementary Information.

The median Sr/Y, (La/Yb)_N_ and calculated average crustal thickness for all rocks are plotted in Fig. 2 (the subscript “N” for La/Yb implies that that ratio was normalized to chondritic values of McDonough and Sun, 1995^40^). We performed a simple least-squares regression through these data subsets, except for the data subset of North Eastern Anatolia (E). There appears to be only negligible differences between the correlations constructed from sub-alkaline samples and from all samples (Supplementary Information). Therefore, we suggest that alkaline rocks do not have significant influence on the correlations and hereafter we only use the correlations constructed from all samples. The empirical relationship between Sr/Y and La/Yb to crustal thickness are modeled by:

1 $$Sr/Y=1.49{D}_{M}-42.03,\,or\,{D}_{M}=0.67Sr/Y+28.21$$

2 $${(La/Yb)}_{N}=2.94{e}^{(0.036{D}_{M})},\,or\,{D}_{M}=27.78\,\mathrm{ln}\,[0.34{(La/Yb)}_{N}]$$

where Sr/Y and (La/Yb)_N_ are whole rock median values, and D_M_ is average crustal thickness or Moho depth.

### Application to Qinling Orogenic Belts

The geochemical data sources and detailed data compilation for the QOB are listed in the Supplementary Information. We separated samples into several subsets based on their location and age. Then we use the procedure discussed above to process all these data. The only difference is that we do not remove outliers of subsets with standard deviation (std) <5 to retain the original data as much as possible. We used the correlations between Sr/Y, (La/Yb)_N_ and Moho depth obtained above (equation (1) and (2)) to calculate the crustal thicknesses in the QOB through time. We propagate uncertainty from the regression into our calculation of the uncertainty for Moho depth.

Previous studies proposed that >240 Ma granitoid rocks were related to oceanic subduction, however, these intrusions where far away from the suture zone were more likely the products in back-arc basin due to slab roll-back^30, 33, 37, 41^. Therefore, equations from Chapman *et al*. (2015)^5^ and Profeta *et al*. (2015)^7^ are not suitable for these rocks. Instead, we use the equations from this study to estimate the crustal thickness of those subsets. Five subsets (Xiekeng, Maixiu, Xinyuan, Longcaoping, and Liuba) have relatively low (La/Yb)_N_ and the calculated Moho depth are lower than 35 km. However, the calculated Moho depth based on (La/Yb)_N_ by using the fit from Profeta *et al*. (2015)^7^ are quite similar to the Moho depth that are calculated from Sr/Y by using equation (1) (Supplementary Information). Therefore, we use equation from Profeta *et al*. (2015)^7^ to calculate (La/Yb)_N_-based Moho depth of those four subsets.

In general, the calculated Moho depth from Sr/Y and (La/Yb)_N_ show a good agreement and have acceptable uncertainties. The average difference between results acquired from Sr/Y and (La/Yb)_N_ is 5.2 km with std of 4.4 and the average uncertainty of the Moho depth is 9.4 km with std of 1.2 (Supplementary Information). The uncertainty of Moho depth in this study is similar to the result from Chapman *et al*. (2015)^5^. However, the uncertainty of Moho depth calculated from (La/Yb)_N_ is larger than calculated from Sr/Y. This is mainly caused by the higher uncertainty in the equation (2) than equation (1), since we considered uncertainty propagation.

## Discussion

### Comparison with correlations obtained from magmatic arcs

Generally, the correlations obtained in this study are similar to the ones established based on subduction-related arcs (Fig. 2). Both fits show positive linear correlation between Sr/Y and Moho depth and exponential correlation between (La/Yb)_N_ and Moho depth. However, some differences with the results of Chapman *et al*. (2015)^5^ and Profeta *et al*. (2015)^7^ exist. First, our data subsets show a much narrower range of crustal thickness and the average crustal thickness of our data subsets (~45 km) is larger than in magmatic arcs (~30 km)^5, 6^. Even though the crustal thickness in the Andes reaches ~70 km, the average crustal thickness in modern subduction magmatic arcs is still thinner than continental collisional belts. Secondly, at a given Moho depth, the median Sr/Y in continental collisional belts is smaller than rocks in magmatic arcs, especially when the crust is shallower than 45 km, whereas the median (La/Yb)_N_ in continental collisional belts is higher than that from subduction-related magmatic arcs.

**Figure 3:**
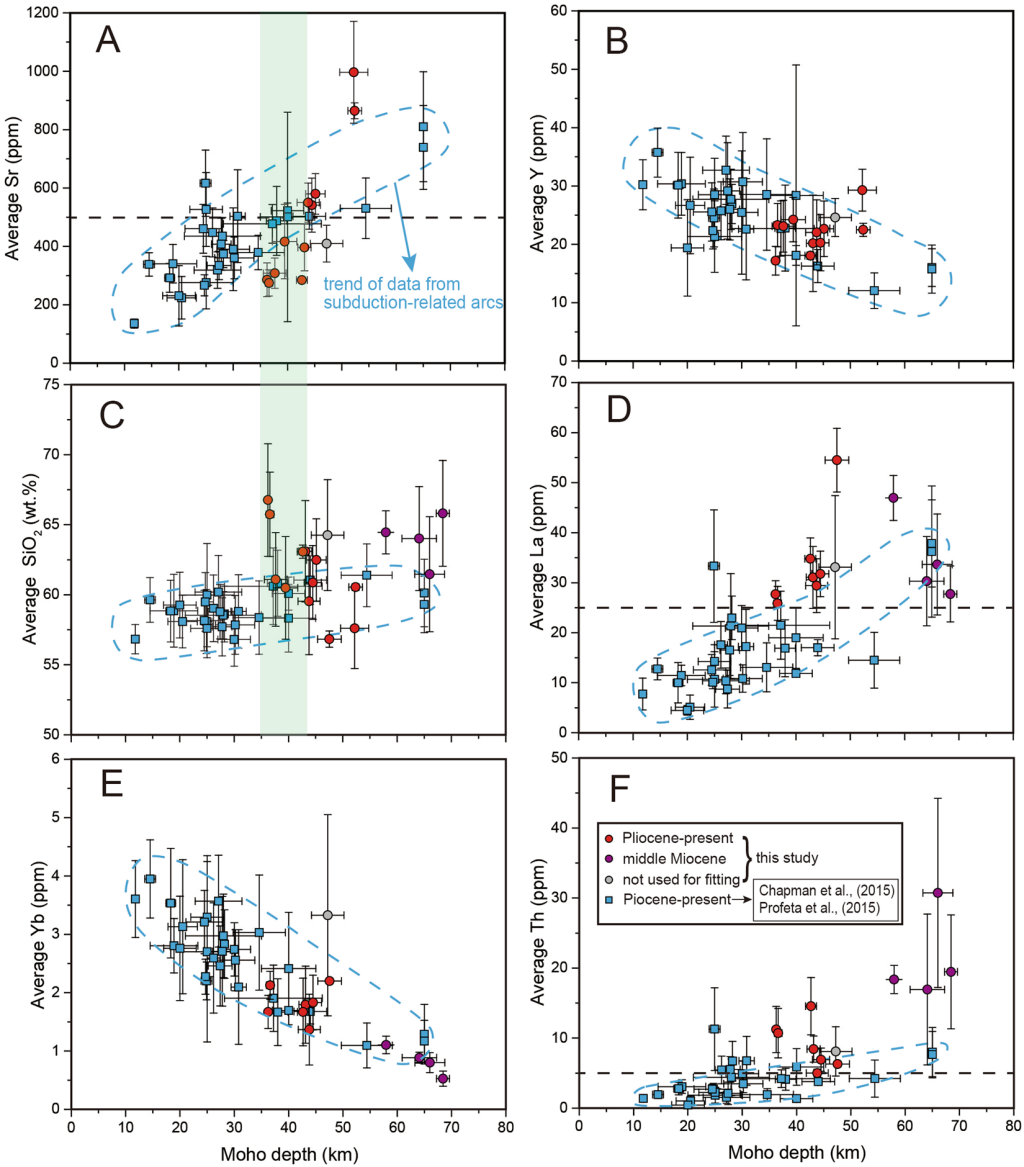
Comparison between data subsets in continental collisional belts (this study) and magmatic arcs^5, 7^ in whole-rock geochemical features at different Moho depth. (**A**) Average Sr (ppm); (**B**) Average Y (ppm); (**C**) Average SiO_2_ (wt.%); (**D**) Average La (ppm); (**E**) Average Yb (ppm); and (**F**) Average Th (ppm). The red, purple and grey circles represent data subsets from continental collisional belts (same to Fig. 2) and the blue squares represent data subsets from magmatic arcs. The blue dashed line represents trend of data from subduction-related arcs^5, 7^. The plotted data are listed in Supplementary Information. See text for details.

**Figure 4:**
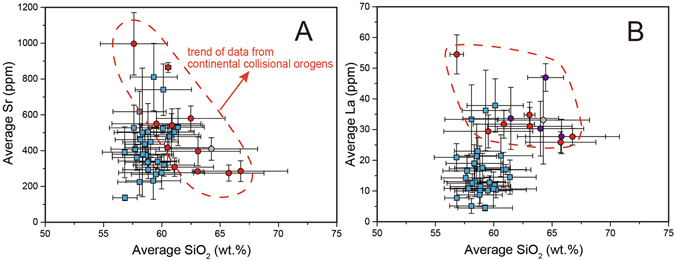
Relationship between whole-rock average Sr (ppm) (**A**), average La (ppm) (**B**) and average SiO_2_ (wt.%) of data subsets in continental collisional belts (this study) and subduction-related arcs^5, 7^, reflecting lower Sr content in collisional belts being related to early plagioclase crystallization and uniformly high La content in collisional belts. The symbols are same to Fig. 3. The red dashed line represents trend of data from continental collisional orogens. The plotted data are listed in Supplementary Information.

We show that the Sr content of collisional belts is lower than that of subduction arcs when crust is shallower than 45 km, although they share similar Y content (Fig. 3A and B). In general, the Y content of rocks in continental collisional belts is within the range of those in magmatic arcs (Fig. 3B). The distinct low Sr content in collisional belts where Moho depth <45 km seems to be related to their comparatively higher SiO_2_ concentration and higher Rb/Sr (Figs﻿. 3C and 4A; Supplementary Information). In addition, the Sr/Y difference between data subsets with lower SiO_2_ and Rb/Sr of collisional belts and magmatic arcs are nearly within the error. This observation reflects that low Sr/Y is due to early plagioclase crystallization leading to a decrease in Sr content and increase in SiO_2_ and Rb/Sr (Fig. 4A). Therefore, the difference of Sr/Y at thinner crust is mainly caused by variability of differentiation on a regional scale.

As for the (La/Yb)_N_ ratio, almost all data subsets in this study are higher than 10, whereas only few points (e.g. Andes) from magmatic arcs are higher than 8^7^ (Fig. 2B). They have similar Y content, whereas subduction arcs have lower La concentrations (generally <25 ppm)^7^ (Fig. 3D and E). Since La content does not correlate with SiO_2_ (Fig. 4B), the higher La concentrations in rocks from continental collisional belts must reflect a fundamental difference in the partial melting/differentiation mechanisms that collisional belts and magmatic arcs undergo. Collisional belts also have higher Th content of rocks than subduction arcs (Fig. 3F). Only in the central Andes have comparable La and Th contents (Fig. 3D and F). One possible explanation for these differences is that monazite saturation and dissolution during partial melting of the lower crust in collisional belts provides the excess Th and LREE compared to subduction arcs. Monazite is a principal sink for Th and LREE in the crust; its breakdown during lower crustal anatexis is related to a saturation threshold^42^ which may be exceeded in collisional arcs^43^ more often than in subduction arcs.

### Paleo-Moho depth in the Qinling Orogenic Belt

**Figure 5:**
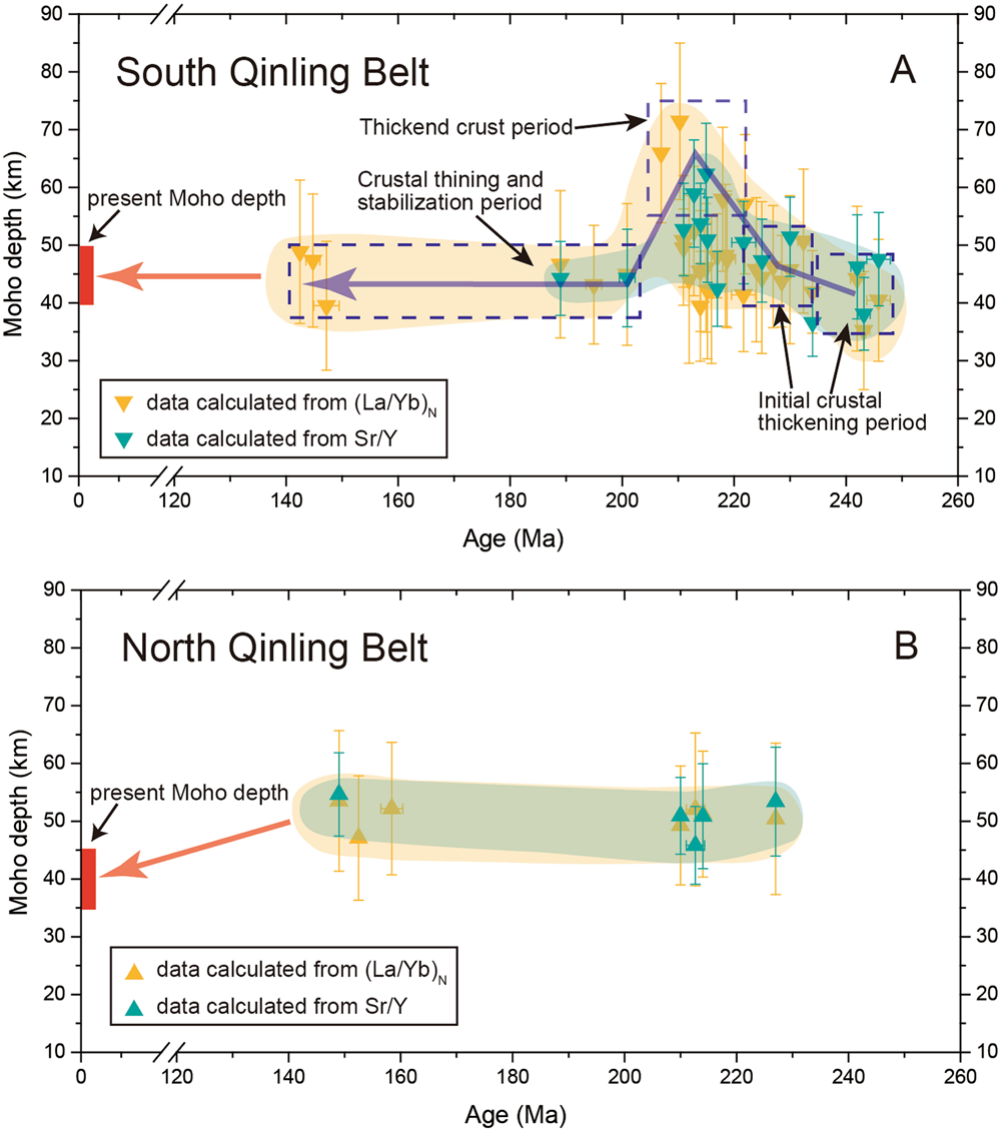
Changes of calculated crustal thickness from Sr/Y and (La/Yb)_N_ over time for the South Qinling Belt (**A**) and North Qinling Belt (**B**). Crustal thickness with uncertainty is calculated by using the correlation equations in this study. Note that five subsets (Xiekeng, Maixiu, Xinyuan, Longcaoping, and Liuba) are used fits from Profeta *et al*. (2015)^7^ to calculate (La/Yb)_N_-based Moho depth. The plotted data are listed in Supplementary Information. See text for details.

**Figure 6:**
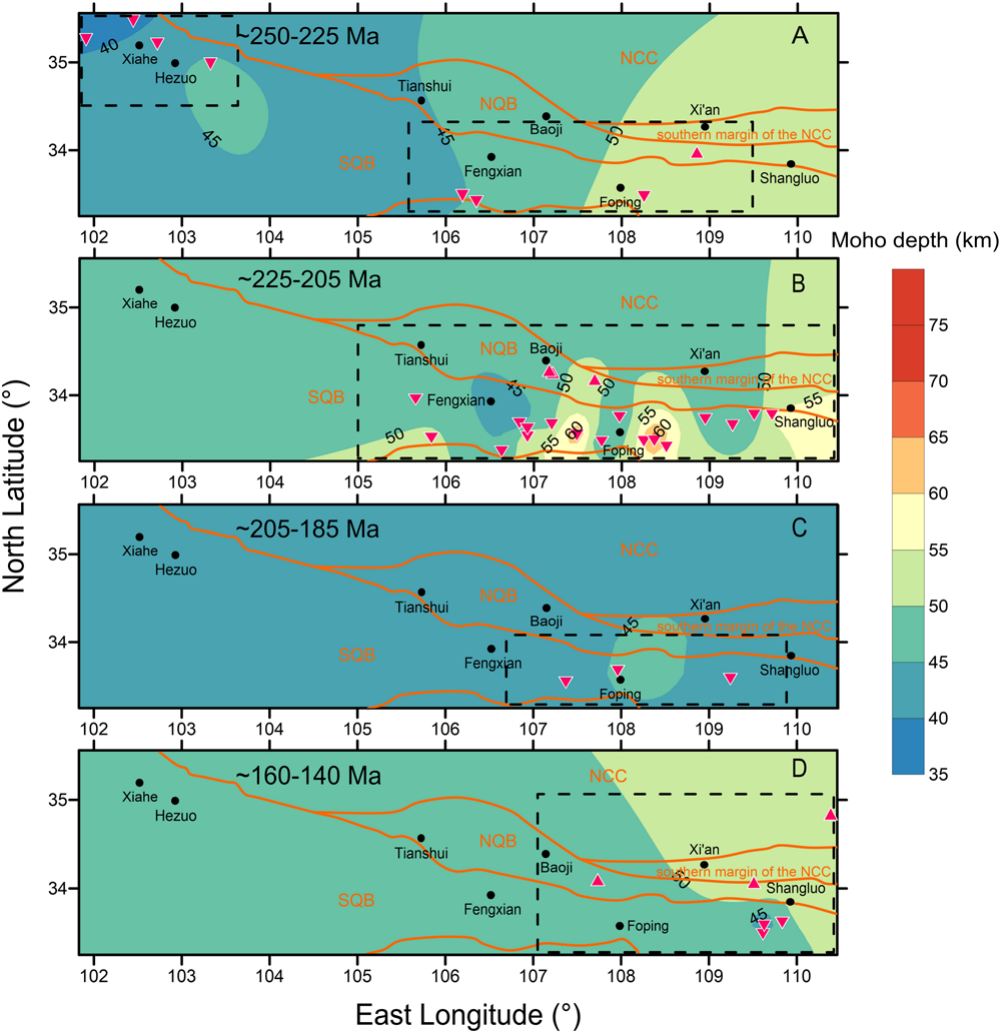
Contour plots of crustal thickness in the Qinling Orogenic Belt at different time periods. (**A**) ~250–225 Ma; (**B**) ~225–205 Ma; (**C**) ~205–185 Ma; and (**D**) ~160–140 Ma. The dashed rectangles represent areas that have more reliable crustal thickness values constrained by calculated data points. Triangles represent data from the North Qinling Belt and inverted triangles represent data from the South Qinling Belt. The plotted data are listed in Supplementary Information. Abbreviation: NCC = North China Craton; NQB = North Qinling Belt; SQB = South Qinling Belt.

The Sr/Y and (La/Yb)_N_ data complied here suggest that crustal thickness has changed significantly though time in the SQB (Fig. 5A), whereas it remained almost constant in the NQB during the ~250–140 Ma period (Fig. 5B). In the SQB, crustal thickness began to increase in the Early to Middle Triassic at a slow rate, increasing from ~40 km to ~50 km from ~250 to 230 Ma (Fig. 5A). This period is thought to be related to the oceanic subduction and initial continental collision^34, 37, 41^. The crustal thickness remained the same during ~230–225 Ma in the SQB (Fig. 5A). The contour map suggests that the crust is relatively thinner in the western QOB during this period and has started to thickening in the eastern QOB (Fig. 6A).

Then the crustal thickness in the QOB began to increase significantly starting at ~225 Ma and reached its maximum thickness (~60–70 km) at ~216 Ma in the SQB (Fig. 5A). The contour map shows significant crustal thickening in the eastern SQB during this period (Fig. 6B). The timing of maximum crustal thickening in the SQB has been constrained by the Foping granulite at the core of the SQB, which was formed at ~220 Ma^44^. This estimate is broadly consistent with our calculation (Fig. 5A). In addition, the widely distributed upper Triassic molasse of the Xujiahe Formation in the southern foreland fold belt (Dabashan) exhibits paleo-current directions originating from the north, suggesting that uplift of the SQB occurred during the Late Triassic^30^.

Crustal thickening was likely related to tectonic shortening. For instance, the outcrop trends of most granitoid intrusions are generally parallel to the trend of the orogen, reflecting N-S compression (Fig. 1). In addition, fold and thrust structures are thought to be active during the Late Triassic^30^. Moreover, eclogite in the Dabie Orogen suggested the SCB was subducted to great depths (~200 km)^38^ and some studies have proposed that very negative zircon εHf values (< −20)^32, 45^ indicating the subducted continental crust was involved in the generation of the high-Mg magmatic rocks in the SQB.

Additionally, we propose that underplating of abundant basaltic magma may also contribute to the crustal thickening. It has been proposed that underplated magma could increase crustal thicknesses by as much as 20 km (e.g. Tibet^19^). Previous studies have suggested that a slab tear (break-off) event occurred leading to underplating of basaltic magma during the Late Triassic (~225–205 Ma)^32, 34, 35^. This interpretation is supported by: (1) the migration of depocenter of foreland basins^46^, (2) the westward younging trend of magmatic enclaves in granitoid intrusions and mafic dykes during ~220–210 Ma^32^, (3) lamprophyre dykes at ~219 Ma^47^, and (4) a peak in magmatic activity during ~218–208 Ma (Fig. 1). The onset of more rapid crustal thickening and mantle-derived magma (magmatic enclaves, mafic dykes, and lamprophyre dykes) are almost simultaneous in the QOB, indicating that the input of mantle-derived magma may be contributing to the rate of crustal thickening. Such a process is also responsible for the crustal growth in the continental collisional belts^19, 32, 34, 35^. We propose that underplating in conjunction with tectonic shortening may have thickened the continental crust from ~50 km to 70 km during the Triassic.

The La/Yb and Sr/Y data suggests that the crustal thickness in the eastern SQB decreased after ~210 Ma, and reached ~45 km at ~200 Ma (Figs. 5A and 6C). This requires a mechanism to thin the crust over a relatively short time period. Previous field and geochemical studies suggested that the SQB experienced rapid uplift and exhumation since ~210 Ma^30, 48, 49^. Magmatic activity decreased significantly after ~208 Ma as well and was dominated by volumetrically small and felsic plutonism indicating that mafic magmatic additions have contributed little to magma generation and crustal thickness (Fig. 1). Previous studies have suggested that slab break-off finished at ~205 Ma and the thickened lower crust may have delaminated between 210 and 200 Ma^30, 34^. Such a mechanism could be responsible for crustal thinning and uplift in the SQB. The data suggests that Moho depth became stable (~45 km) in the SQB during ~200 Ma to ~190 Ma.

The QOB experienced a magmatic lull from ~190–160 Ma (Fig. 1), and evolved into an intracontinental orogen stage during the Jurassic-Paleogene. Only minor magmatism was generated during this stage where mainly in the eastern QOB^33^ (Fig. 1). The prominent fold-thrust belts that formed during the Jurassic to Early Cretaceous (~160–140 Ma) suggest that the QOB was in a compressional tectonic setting. These fold-thrust belts are generally developed at the northern margin of SCB and southern SQB where no magmatic rocks are exposed^30, 50^. However, the crustal thickness calculated from Sr/Y and (La/Yb)_N_ remains almost the same during this period, which is ~45 km in the SQB and ~50 km in the NQB (Figs. 5 and 6D). Therefore, our data can only reflect that the crustal thickness at central area of QOB does not have distinct changes during ~160–140 Ma. Seismic reflection experimental studies suggest that the modern-day Moho depth is about ~45 km in the SQB and ~40 km in the NQB^51, 52^. This indicates the crust may have been stable in the eastern SQB since the Jurassic. However, apatite fission track and apatite and zircon (U–Th)/He data^53^ suggest tectonic collapse in the NQB during Cenozoic.

### The combined utilization of correlation equations

This study extends the recent work of Chapman *et al*. (2015)^5^, Chiaradia (2015)^6^, Profeta *et al*. (2015)^7^, and Turner and Langmuir (2015)^8^, which focused on the relationship between geochemical indices and crustal thickness in magmatic arcs. We correlated Sr/Y and La/Yb with modern crustal thickness in continental collisional orogens and then used this correlation to examine changes in crustal thickness in an ancient collisional orogen, the QOB. The results discussed above give us confidence in the ability of coupled Sr/Y and La/Yb correlations to help estimate paleo-crustal evolution in continental collisional belts. Combination of our regression equations with those from Chapman *et al*. (2015)^5^ and Profeta *et al*. (2015)^7^ can provide a way to track changes in crustal thickness as an orogen evolves from a continental arc experiencing oceanic subduction to a final continental collision. With these knowledge, we could acquire more detailed information about the stages of geodynamic evolution and ways of crustal thickening and crustal growth during orogenic processes.

## Conclusions

Establishing empirical relationships between Sr/Y and La/Yb and crustal thickness in continental collisional orogens extends recent similar relationships established for subduction-related magmatic arcs. We show that the correlations between whole-rock Sr/Y, La/Yb and Moho depth for intermediate magmatic rocks from modern continental collisional belts at global scale are similar to the correlations for modern subduction-related magmatic arcs (similar slope values). These correlations can be used to track changes in crustal thickness through time in ancient collisional belts. In the case of the Qinling Orogenic Belt, we explore the changes of crustal thickness during collision using this empirical relationship, which reflects important shifts in the geodynamics and are in good agreement with independent geologic studies. In addition, the calculated Moho depth from Sr/Y and (La/Yb)_N_ in the QOB are generally consistent and the uncertainty of Moho depth is ~±9 km. We propose that the coupled La/Yb and Sr/Y can help track crustal thickness changes of ancient collisional belts.

## Methods

The geochemical data sources and a detailed data compilation of present global continental collisional belt crustal thickness are in the Supplementary Information. The average crustal thickness for each subset is based on the location and regional crustal thicknesses adapted from CRUST 1.0, and the uncertainty is the standard deviation of crustal thickness for that area^1^. The data we collected spans sub-alkaline to alkaline fields and most sub-alkaline rocks belong to calc-alkaline series (Supplementary Information). To examine the potential influence of alkalinity on Sr/Y and La/Yb ratios, we examined sub-alkaline samples by themselves and then all samples together. For all of the datasets, we selected samples with a relatively wider range of SiO_2_ (55–72 wt%) and MgO (0.5–6.0 wt.%) compared to the windows used by Chapman *et al*. (2015)^5^ and Profeta *et al*. (2015)^7^. We then removed Sr/Y and (La/Yb)_N_ outliers from each data subsets by using modified Thompson tau statistical method. We discarded data subsets with average Rb/Sr > 0.35 or with std > 10. Finally we calculated the median Sr/Y and (La/Yb)_N_ of each subset. The Rb/Sr filter is used to remove samples that are strongly influenced by fractionation within the crust. We eliminate high Sr/Y (average > 60) and high La (average > 60) data subsets from our fits to calculate crustal thickness due to their few data subsets and undefined petrogenesis with adakitic features (Supplementary Information). Tibet is the archetypal continental collisional orogen and to expand our data set we included data from Middle Miocene (<18 Ma) magmatism in southern Tibet. To estimate crustal thickness in southern Tibet in the Middle Miocene, we converted paleoelevation to paleo-crustal thickness using an Airy isostasy model^54^. Using the available paleoaltimetry data^22, 23^ (4650 to 5200 m) and average densities of upper mantle^55^ (3.27 g/cm^3^) and crust^55^ (2.77 g/cm^3^), suggests a Middle Miocene crustal thickness of 66 to 69 km in southern Tibet. These estimates are slightly less than the modern crustal thickness estimate from CRUST 1.0 (~74 km), which may suggest that crustal thickness in southern Tibet has increased ~7 km since Middle Miocene. Both Sr/Y and (La/Yb)_N_ data subsets in this study do not have data points with Moho depth shallower than 35 km or higher than 70 km. The Moho depth in continental collisional belts is likely never shallower than 35 km, although it is possible that it may be higher than 70 km. Caution should be applied when using these correlations if the results obtained suggest a Moho depth shallower than 35 km or deeper than 70 km. All data collected and complied during this study are included in this published article and Supplementary Information.

## Electronic supplementary material

Supplementary Information
