# Supplementary material for: Quantifying Crustal Thickness in Continental Collisional Belts: Global Perspective and a Geologic Application
Source: Sci Rep. 2017 Aug 1;7:7058. doi: 10.1038/s41598-017-07849-7 (PMC5539297; doi:10.1038/s41598-017-07849-7)
Supplement: Supplementary file 1 — Supplementary Information [file 41598_2017_7849_MOESM1_ESM.pdf]

Quantifying Crustal Thickness in Continental Collisional Belts: Global perspective and a Geologic Application

Fangyang Hu, Mihai N. Ducea, Shuwen Liu, James B. Chapman

Supplementary Information

Contents:

PART I: Global Data Compilation

- **Methods**
- **Supplementary Figure S1**
- **Supplementary Figure S2**
- **Supplementary Figure S3**
- **Supplementary Table S1**
- **Supplementary Table S2**
- **Supplementary Table S3**
- **Supplementary Table S4**
- **Supplementary Table S5**
- **Supplementary Table S6**

PART II: Qinling Data Compilation

- **Supplementary Figure S4**
- **Supplementary Table S7**
- **Supplementary Table S8**
- **Supplementary Table S9**
- **Supplementary Table S10**

PART I
(Global Data Compilation)

Methods

The reasons why we primarily chose intermediate rocks for constructing correlation between Sr/Y, La/Yb and Moho depth are as follows. First, basalt geochemistry may not be directly indicative of crustal thickness even though some recent compilations[1,2] clearly to show that geochemistry of basalts too, surprisingly, correlate with crustal thickness. However, to help directly compare continental collision magmatism with subduction-related magmatism regarding Sr/Y and La/Yb, we investigated only intermediate rocks. Secondly, collision-related magmatism often includes relatively shallowly derived S-type silicic melts formed by partial melting of hot regional metamorphic domains at depths as shallow as 15-20 km. For example, Himalayan-type leucogranites[3] are believed to be some of the most common products of "collisional magmatism". Such high silica granitoids (or rhyolites at the surface) are excluded (SiO_2 > 72 wt.%) from our analysis because they are not differentiated via deep crustal processes that operate in a typical deep crustal MASH zone, yet they can mimic the behavior of deep crustal sourced rocks due to the presence of peritectic garnet[4,5]. Therefore, we wanted to eliminate rocks that are mafic as well as high silica (rhyolite) rocks (Figure S1).

Rb/Sr filter is used to eliminate rocks that affected by highly fractionation of feldspar. This is because Sr concentration in the melt will decline significantly as feldspar crystallizes, resulting in an increase in Rb/Sr and decrease in Sr/Y (Figure S1).

We also deleted data subsets with average Sr/Y > 60 and average La > 60 ppm. This is because even with the SiO_2 MgO, Rb/Sr filters, the correlations to the Moho depth of these samples are different with other subsets. Some of the high Sr/Y (> 60) subsets are discarded because of their large standard deviation. However, regional studies suggested that these high Sr/Y subsets may be derived from melting of subducted slab or high Sr/Y rocks in thickened lower crust or even enriched lithospheric mantle[5-9]. They represent a small proportion of data in our compilation and

we have no straightforward explanation for them. Consequently, we eliminate these high Sr/Y (> 60) analyses from our fits to calculate crustal thickness.

Samples with extremely high La/Yb (>50) are characterized by enriched La rather than depleted Yb (Figure S2). The variations in La concentrations may be governed by the geochemical features of the source rocks or mineral effects during partial melting and fractionation[5]. According to Moyen (2009)[5], high La/Yb rocks that formed because of residual garnet in the cumulate should have relatively high La/Yb (>20) with low Yb content (< 1ppm). Regional studies of the high La/Yb samples proposed that these particular rocks were formed by melting of thickened lower crust or enriched metasomatized lithospheric mantle or even asthenosphere[10-13]. Therefore, we consider that these high La/Yb samples are not suitable for estimating crustal thickness because their chemistry may be controlled by mantle source rocks and not magmatic processes in the lower crust. We then discarded $(La/Yb)_N$ subsets with average La > 60 ppm to reduce the influence of these notably high La/Yb samples.

References for methods:

1. Chiaradia, M. Crustal thickness control on Sr/Y signatures of recent arc magmas: an Earth scale perspective. *Sci. Rep.* **5**, doi:10.1038/srep08115 (2015).
2. Turner, S. J. & Langmuir, C. H. The global chemical systematics of arc front stratovolcanoes: Evaluating the role of crustal processes. *Earth Planet Sc. Lett.* **422**, 182–193 (2015).
3. Gaillard, F., Scaillet, B. & Pichavant, M. Evidence for present-day leucogranite pluton growth in Tibet. *Geology* **32**, 801–804 (2004).
4. Stevens, G., Villaros, A. & Moyen, J.-F. Selective peritectic garnet entrainment as the origin of geochemical diversity in S-type granites. *Geology* **35**, 9–12 (2007).
5. Moyen, J. F. High Sr/Y and La/Yb ratios: The meaning of the “adakitic signature”. *Lithos* **112**, 556–574 (2009).
6. Defant, M. J. & Drummond, M. S. Derivation of some modern arc magmas by melting of young subducted lithosphere. *Nature* **347**, 662–665 (1990).
7. Dilek, Y., Imamverdiyev, N. & Altunkaynak, Ş. Geochemistry and tectonics of

Cenozoic volcanism in the Lesser Caucasus (Azerbaijan) and the peri-Arabian region: collision-induced mantle dynamics and its magmatic fingerprint. Int. Geol. Rev. **52**, 536–578 (2010).

8. Mirnejad, H., Hassanzadeh, J., Cousens, B. L. & Taylor, B. E. Geochemical evidence for deep mantle melting and lithospheric delamination as the origin of the inland Damavand volcanic rocks of northern Iran. . *J. Volcanol. Geoth. Res.* **198**, 288–296 (2010).
9. Hou, Z. Q. et al. Eocene–Oligocene granitoids in southern Tibet: Constraints on crustal anatexis and tectonic evolution of the Himalayan orogen. *Earth Planet Sc. Lett.* **349–350**, 38–52 (2012).
10. Wang, Q. et al. Pliocene-Quaternary crustal melting in central and northern Tibet and insights into crustal flow. *Nat. Commun.* **7**, 11888, doi:10.1038/ncomms11888 (2016).
11. Davidson, J. et al. The geology of Damavand volcano, Alborz Mountains, northern Iran. *Geol. Soc. Am. Bull.* 116, 16–29 (2004).
12. Williams, H. M., Turner, S. P., Pearce, J. A., Kelley, S. P. & Harris, N. B. W. Nature of the Source Regions for Post-collisional, Potassic Magmatism in Southern and Northern Tibet from Geochemical Variations and Inverse Trace Element Modelling. *J. Petrol.* **45**, 555–607 (2004).
13. Zhu, D.-C., Wang, Q., Cawood, P. A., Zhao, Z.-D. & Mo, X.-X. Raising the Gangdese Mountains in southern Tibet. *J. Geophys. Res-Sol. Ea.* **122**, 214–223 (2017).

Supplementary Figures

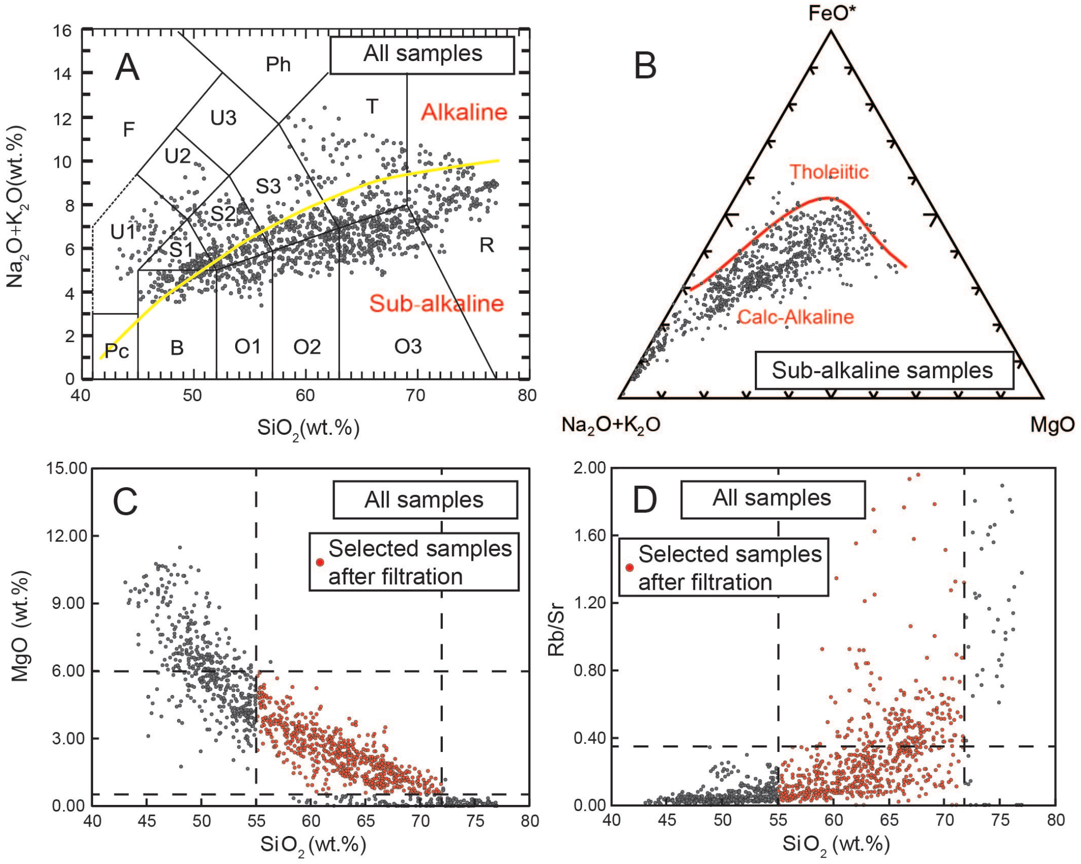

Figure S1. Basic geochemical features of young rocks from global continental collisional orogens. (A) Total alkali versus SiO_2 (wt.%) diagram (Le Maitre, 2002)[1]. Pc – picrobasalt; B – basalt; O1 – basaltic andesite; O2 – andesite; O3 – dacite; R – rhyolite; S1 – trachybasalt; S2 – basaltic trachy-andesite; S3 – trachyandesite; T – trachyte or trachydacite; U1 – tephrite or basanite; U2 – phonotephrite; U3 – tephriphonolite; Ph – phonolite; F – foidite. The yellow curve line separates sub-alkaline series and alkaline series (Irvine and Baragar, 1971)[2]. (B) AFM diagram (Irvine and Baragar, 1971)[2]. Only sub-alkaline data are plotted. (C) MgO (wt.%) versus SiO_2 (wt.%) diagram. (D) Rb/Sr versus SiO_2 (wt.%) diagram. The red circle represent data are used after filtration by SiO_2 (55-72 wt.%) and MgO (0.5-6 wt.%). The dashed lines represent SiO_2, MgO and Rb/Sr filters. Data are listed in Table S1.

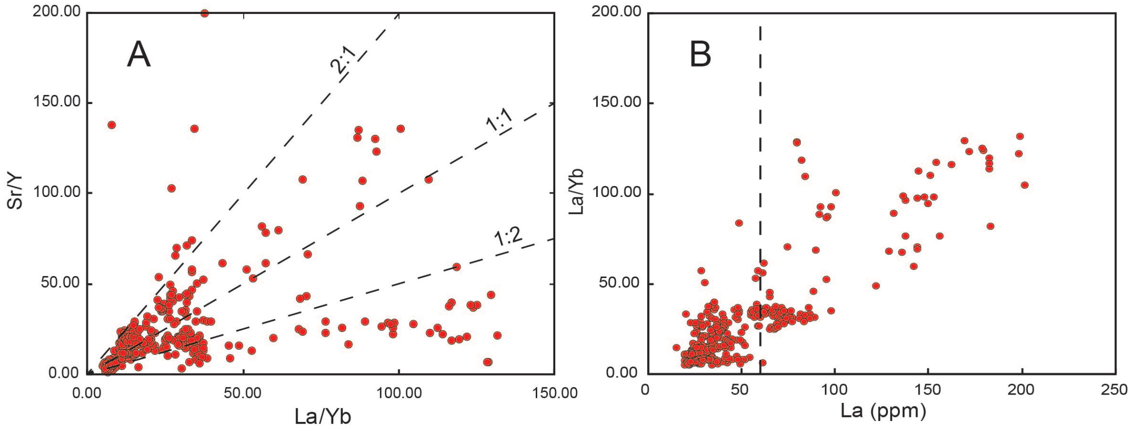

Figure S2. The relationships between Sr/Y, La (ppm) and La/Yb. (A) Sr/Yb versus La/Yb showing variations of ratio of Sr/Y to La/Yb. (B) La/Yb versus La (ppm) displaying positive correlation between La/Yb and La. Data are listed in Table S2.

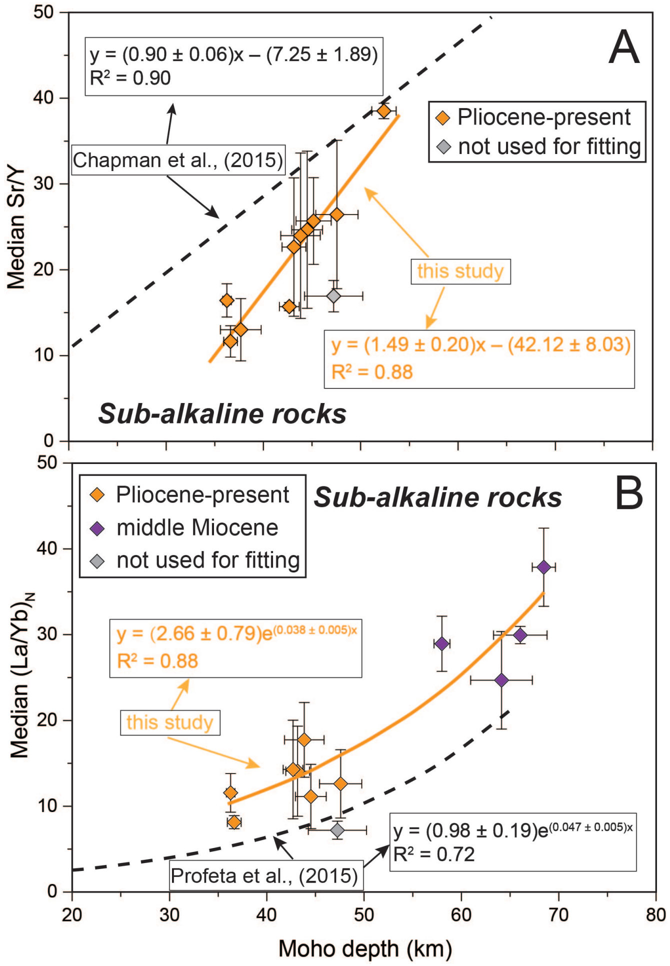

Figure S3. Global correlations between geophysically determined crustal depth (CRUST 1.0)[3] and median Sr/Y (A) and median $(La/Yb)_N$ (B) of calc-alkaline rocks from

continental collisional belts. The yellow diamonds represent the rocks formed during Pliocene to present and purple diamonds represent the rocks formed at middle Miocene. The grey circles represent data subset from continental collisional belts but not used for calculating correlation equations. Regression line and empirical relationship with R^2 are shown on each diagram. The yellow solid lines represent the results of this study and the black dash lines represent the result of study on subduction arcs by Chapman et al. (2015)[4] and Profeta et al. (2015)[5]. Data are listed in Table S3.

References for supplementary figures:

1. Le Maitre, R.W. Classification and nomenclature in *Igneous Rocks* (ed. Le Maitre, R.W.) 35–36 (Cambridge, 2002).
2. Irvine, T. N. & Baragar, W. R. A. A Guide to the Chemical Classification of the Common Volcanic Rocks. *Can. J. Earth Sci.* **8**, 523–548 (1971).
3. Laske, G., Masters., G., Ma, Z. & Pasyanos, M. Update on CRUST1.0 - A 1-degree Global Model of Earth's Crust, *Geophys. Res.* Abstracts **15**, http://meetingorganizer.copernicus.org/EGU2013/EGU2013-2658.pdf (2013).
4. Chapman, J. B., Ducea, M. N., Profeta, L. & DeCelles, P. G. Tracking changes in crustal thickness during orogenic evolution with Sr/Y; an example from the Western U.S. Cordillera. *Geology* **43**, 919–923 (2015).
5. Profeta, L., et al. Quantifying crustal thickness over time in magmatic arcs: *Sci. Rep.* **5**, doi:10.1038/srep17786 (2015).

Supplementary Tables

Table S1. Global young (Pliocene-present) continental collisional rock data used to plot Figure S1.

Source	Location	Sample	Latitude	Longitude	SiO2(wt.%)	MgO(wt.%)	K2O(wt.%)	Na2O(wt.%)	Rb(ppm)	Sr(ppm)	Y(ppm)	La(ppm)	Yb(ppm)	Rb/Sr	Sr/Y	La/Yb
GEOROC[1]	North Eastern Anatolia (W)	MK261	40.06	41.36	64.30	2.21	2.74	3.89	85	395	18.00	26.00		0.22	21.94	
GEOROC[1]	North Eastern Anatolia (W)	MK228	40.06	41.36	63.40	2.39	2.74	3.83	60	461	12.00	30.00		0.13	38.42	
GEOROC[1]	North Eastern Anatolia (W)	MK251	40.06	41.36	65.10	2.17	2.35	4.21	59	454	14.00	33.10	1.04	0.13	32.43	31.83
GEOROC[1]	North Eastern Anatolia (W)	MK36	40.06	41.36	63.80	2.07	2.51	4.00	72	443	14.00	28.00		0.16	31.64	
GEOROC[1]	North Eastern Anatolia (W)	MK49	40.06	41.36	66.30	1.89	2.85	3.88	83	318	16.00	33.90	1.36	0.26	19.88	24.93
GEOROC[1]	North Eastern Anatolia (W)	MK52A	40.06	41.36	63.80	2.29	2.70	3.80	71	434	13.00	39.00		0.16	33.38	
GEOROC[1]	North Eastern Anatolia (W)	MK93	40.06	41.36	63.50	2.43	2.76	3.58	72	443	15.00	38.50	1.35	0.16	29.53	28.52
GEOROC[1]	North Eastern Anatolia (W)	MK265	40.12	41.58	63.90	3.65	2.73	3.53	70	453	16.00	31.40	1.14	0.15	28.31	27.54
GEOROC[1]	North Eastern Anatolia (W)	MK277	40.12	41.58	60.60	4.08	2.18	4.11	62	489	16.00	28.10	1.33	0.13	30.56	21.13
GEOROC[1]	North Eastern Anatolia (W)	MK90	40.12	41.58	65.10	2.46	2.91	3.71	74	546	10.00	23.00		0.14	54.60	
GEOROC[1]	North Eastern Anatolia (W)	MK63	40.12	41.58	54.40	4.87	1.22	3.39	10	517	32.00	37.20	2.79	0.02	16.16	13.33
GEOROC[1]	North Eastern Anatolia (W)	MK268	40.12	41.58	65.50	2.03	3.27	3.92	91	350	15.00	29.20	1.37	0.26	23.33	21.31
GEOROC[1]	North Eastern Anatolia (W)	MK281	40.12	41.58	57.00	2.23	2.00	4.68	52	417	31.00	24.70	2.67	0.12	13.45	9.25
GEOROC[1]	North Eastern Anatolia (W)	MK289	40.12	41.58	49.10	7.02	0.51	3.11	11	455	25.00	12.30	2.61	0.02	18.20	4.71
GEOROC[1]	North Eastern Anatolia (W)	MK125	39.98	41.68	73.60	0.02	4.99	4.18	142	31	36.00	33.00		4.58	0.86	
GEOROC[1]	North Eastern Anatolia (W)	MK338	39.98	41.68	71.00	0.01	5.34	4.51	133	49	34.00	63.30	2.72	2.71	1.44	23.27
GEOROC[1]	North Eastern Anatolia (W)	MK101	39.98	41.68	57.00	4.56	2.00	3.48	54	434	21.00	30.20	2.22	0.12	20.67	13.60
GEOROC[1]	North Eastern Anatolia (W)	MK112	39.98	41.68	66.80	1.24	3.26	3.68	78	350	18.00	31.60	1.55	0.22	19.44	20.39
GEOROC[1]	North Eastern Anatolia (W)	MK118	39.98	41.68	66.40	1.24	2.58	3.95	63	413	11.00	19.00		0.15	37.55	
GEOROC[1]	North Eastern Anatolia (W)	MK337	39.98	41.68	66.60	1.23	3.35	3.57	76	367	17.00	21.00		0.21	21.59	
GEOROC[1]	North Eastern Anatolia (W)	MK117	39.98	41.68	62.10	1.99	2.34	4.03	59	369	21.00	20.00	2.00	0.16	17.57	10.00
GEOROC[1]	North Eastern Anatolia (W)	MK106	39.98	41.68	67.50	0.70	4.30	5.01	111	175	33.00	45.00		0.63	5.30	
GEOROC[1]	North Eastern Anatolia (W)	MK318	39.98	41.68	65.10	1.13	4.02	4.94	100	244	37.00	49.00		0.41	6.59	
GEOROC[1]	North Eastern Anatolia (W)	MK319	39.98	41.68	70.70	0.13	4.65	5.19	130	102	35.00	49.00		1.27	2.91	
GEOROC[1]	North Eastern Anatolia (W)	MK359	39.98	41.68	69.20	0.44	4.88	4.57	128	128	33.00	47.00		1.00	3.88	
GEOROC[1]	North Eastern Anatolia (W)	MK343	39.98	41.68	54.60	2.70	2.85	3.16	55	371	35.00	45.00		0.15	10.60	
GEOROC[1]	North Eastern Anatolia (W)	2011-06-13-03	38.70	41.96	63.22	0.41	4.63	5.21	115	137	47.00	55.00		0.84	2.91	
GEOROC[1]	North Eastern Anatolia (W)	4	39.07	41.37	45.65	4.58	1.16	4.58	19	1082	30.40	25.00	2.80	0.02	35.58	8.93
GEOROC[1]	North Eastern Anatolia (W)	26	39.07	41.37	45.17	4.86	1.21	4.22	25	578	50.10	27.00	4.10	0.04	11.54	6.59
GEOROC[1]	North Eastern Anatolia (W)	2	39.07	41.37	56.90	2.88	1.75	4.53	46	425	34.20	28.00	2.50	0.11	12.43	11.20
GEOROC[1]	North Eastern Anatolia (W)	6	39.07	41.37	53.04	2.05	1.33	3.86	44	542	41.00	22.00	3.10	0.08	13.21	7.10
GEOROC[1]	North Eastern Anatolia (W)	40	39.07	41.37	53.91	2.44	1.28	3.96	28	550	28.50			0.05	19.30	
GEOROC[1]	North Eastern Anatolia (W)	42	39.07	41.37	47.10	9.91	0.96	3.32	14	825	21.40			0.02	38.53	
GEOROC[1]	North Eastern Anatolia (W)	43	39.07	41.37	47.60	9.82	0.98	2.80	15	876	19.80			0.02	44.23	
GEOROC[1]	North Eastern Anatolia (W)	44	39.07	41.37	46.80	9.51	1.15	2.93	14	891	19.90			0.02	44.75	
GEOROC[1]	North Eastern Anatolia (W)	7	39.07	41.37	54.30	1.64	1.60	4.12	44	582	33.00	23.00	2.80	0.08	17.64	8.21
GEOROC[1]	North Eastern Anatolia (W)	1	39.07	41.37	56.35	3.36	1.95	4.40	54	492	31.60	44.00	3.00	0.11	15.57	14.67
GEOROC[1]	North Eastern Anatolia (W)	3	39.07	41.37	51.11	4.78	1.34	3.96	27	505	29.60	27.00	2.50	0.05	17.04	10.80
GEOROC[1]	North Eastern Anatolia (W)	11	39.07	41.37	51.23	5.89	0.83	3.65	7	429	28.20	16.00	2.40	0.02	15.21	6.67
GEOROC[1]	North Eastern Anatolia (W)	13	39.07	41.37	52.51	6.06	1.25	3.74	29	497	27.50	26.00	2.40	0.06	18.06	10.83

Source	Location	Sample	Latitude	Longitude	SiO2(wt.%)	MgO(wt.%)	K2O(wt.%)	Na2O(wt.%)	Rb(ppm)	Sr(ppm)	Y(ppm)	La(ppm)	Yb(ppm)	Rb/Sr	Sr/Y	La/Yb
GEOROC[1]	North Eastern Anatolia (W)	22	39.07	41.37	54.71	3.93	1.76	4.12	48	522	33.80	35.00	2.30	0.09	15.45	15.22
GEOROC[1]	North Eastern Anatolia (W)	23	39.07	41.37	54.62	3.93	1.62	4.20	41	469	31.70	25.00	2.70	0.09	14.79	9.26
GEOROC[1]	North Eastern Anatolia (W)	24	39.07	41.37	54.39	4.08	1.72	4.19	43	534	28.40	33.00	2.60	0.08	18.79	12.69
GEOROC[1]	North Eastern Anatolia (M)	MK131	40.05	42.17	56.30	3.86	1.47	3.91	27	399	27.00	16.00		0.07	14.78	
GEOROC[1]	North Eastern Anatolia (M)	MK135	40.05	42.17	50.00	6.29	0.69	3.96	10	423	30.00	11.00	2.62	0.02	14.10	4.20
GEOROC[1]	North Eastern Anatolia (M)	MK130	40.05	42.17	54.40	4.56	0.99	4.87	15	415	33.00	12.80	3.05	0.04	12.58	4.20
GEOROC[1]	North Eastern Anatolia (M)	MK134	40.05	42.17	64.00	2.12	2.45	3.86	61	308	21.00	22.00		0.20	14.67	
GEOROC[1]	North Eastern Anatolia (M)	MK139	40.05	42.17	49.70	7.06	0.73	4.06	9	507	25.00	13.50	2.37	0.02	20.28	5.70
GEOROC[1]	North Eastern Anatolia (M)	MK132	40.05	42.17	64.20	1.48	2.44	4.48	60	699	15.00	39.60	1.19	0.09	46.60	33.28
GEOROC[1]	North Eastern Anatolia (M)	MK133	40.05	42.17	68.40	1.11	3.00	3.65	80	375	9.00	22.00		0.21	41.67	
GEOROC[1]	North Eastern Anatolia (M)	MK140	40.05	42.17	68.60	1.25	2.93	3.84	68	371	10.00	23.00		0.18	37.10	
GEOROC[1]	North Eastern Anatolia (M)	MK144	40.05	42.17	48.80	8.40	1.08	3.29	18	867	26.00	30.60	2.18	0.02	33.35	14.04
GEOROC[1]	North Eastern Anatolia (M)	MK150	40.18	42.82	64.10	1.81	1.83	4.53	38	646	11.00	23.00		0.06	58.73	
GEOROC[1]	North Eastern Anatolia (M)	MK151	40.18	42.82	67.60	1.50	2.39	4.31	66	504	9.00	22.00		0.13	56.00	
GEOROC[1]	North Eastern Anatolia (M)	MK152	40.18	42.82	69.20	0.60	2.41	4.24	65	513	7.00	23.00		0.13	73.29	
GEOROC[1]	North Eastern Anatolia (M)	MK153	40.18	42.82	66.80	1.57	2.58	4.37	77	525	8.00	21.00		0.15	65.63	
GEOROC[1]	North Eastern Anatolia (M)	MK146	40.18	42.82	69.90	0.23	3.80	4.77	94	144	32.00	37.00		0.65	4.50	
GEOROC[1]	North Eastern Anatolia (M)	MK171	40.18	42.82	74.50	0.01	4.94	3.72	77	8	49.00	71.00		9.63	0.16	
GEOROC[1]	North Eastern Anatolia (M)	MK172	40.18	42.82	74.50	0.01	5.11	3.60	79	77	43.00	64.00		1.03	1.79	
GEOROC[1]	North Eastern Anatolia (M)	MK173	40.18	42.82	74.60	0.02	5.13	3.59	74	27	41.00	68.00		2.74	0.66	
GEOROC[1]	North Eastern Anatolia (M)	MK156	40.18	42.82	62.80	2.64	2.42	3.74	67	73	27.00	23.00		0.92	2.70	
GEOROC[1]	North Eastern Anatolia (M)	MK145	40.18	42.82	60.30	3.95	2.03	3.56	59	44	19.00	19.00		1.34	2.32	
GEOROC[1]	North Eastern Anatolia (M)	MK147	40.18	42.82	62.40	3.43	2.40	3.46	64	270	26.00	24.00		0.24	10.38	
GEOROC[1]	North Eastern Anatolia (M)	MK148	40.18	42.82	61.90	2.94	2.54	3.62	66	285	27.00	27.00		0.23	10.56	
GEOROC[1]	North Eastern Anatolia (M)	MK149	40.18	42.82	62.80	3.38	2.49	3.47	65	268	24.00	21.00		0.24	11.17	
GEOROC[1]	North Eastern Anatolia (M)	MK159	40.18	42.82	60.30	1.83	2.05	4.12	55	286	32.00	23.60	2.97	0.19	8.94	7.95
GEOROC[1]	North Eastern Anatolia (M)	MK160	40.18	42.82	61.80	2.86	2.43	3.87	62	292	33.00	28.00		0.21	8.85	
GEOROC[1]	North Eastern Anatolia (M)	MK165	40.18	42.82	63.50	2.50	2.71	3.64	68	265	24.00	23.00		0.26	11.04	
GEOROC[1]	North Eastern Anatolia (M)	MK166A	40.18	42.82	63.60	2.69	2.49	3.55	69	267	25.00	24.00		0.26	10.68	
GEOROC[1]	North Eastern Anatolia (M)	MK166B	40.18	42.82	63.50	2.70	2.46	3.60	70	268	25.00	21.00		0.26	10.72	
GEOROC[1]	North Eastern Anatolia (M)	MK161	40.18	42.82	73.50	0.04	3.94	4.46	60	75	37.00	35.00		0.80	2.03	
GEOROC[1]	North Eastern Anatolia (M)	MK154	40.18	42.82	66.90	1.62	2.50	4.41	74	522	9.00	30.50	0.60	0.14	58.00	50.83
GEOROC[1]	North Eastern Anatolia (M)	MK168	40.18	42.82	60.50	3.39	1.77	3.95	46	313	26.00	17.00		0.15	12.04	
GEOROC[1]	North Eastern Anatolia (M)	MK158	40.18	42.82	61.70	1.41	2.79	4.52	71	368	35.00	34.20	3.41	0.19	10.51	10.03
GEOROC[1]	North Eastern Anatolia (M)	MK162	40.16	43.14	55.30	4.79	1.26	3.47	30	420	23.00	20.20	1.90	0.07	18.26	10.63
GEOROC[1]	North Eastern Anatolia (M)	MK167A	40.16	43.14	74.00	0.03	4.37	4.08	48	30	27.00	38.00		1.60	1.11	
GEOROC[1]	North Eastern Anatolia (M)	MK167B	40.16	43.14	74.20	0.08	4.36	4.08	51	31	31.00	33.00		1.65	1.00	
GEOROC[1]	North Eastern Anatolia (M)	MK169	40.16	43.14	66.80	0.20	3.36	5.16	92	286	42.00	38.00		0.32	6.81	
GEOROC[1]	North Eastern Anatolia (M)	MK170	40.16	43.14	66.00	0.32	3.35	5.09	93	279	39.00	40.00		0.33	7.15	
GEOROC[1]	North Eastern Anatolia (M)	MK163	40.16	43.14	67.50	0.66	3.66	4.33	94	233	37.00	27.00		0.40	6.30	
GEOROC[1]	North Eastern Anatolia (M)	MK175	40.16	43.14	53.70	4.98	1.61	4.07	28	573	28.00	32.60	2.93	0.05	20.46	11.13
GEOROC[1]	North Eastern Anatolia (M)	MK174	40.16	43.14	66.50	0.81	3.16	4.70	77	236	37.00	28.40	2.87	0.33	6.38	9.90
GEOROC[1]	North Eastern Anatolia (M)	BD-3	38.32	42.01	48.36	3.68	2.26	4.14	61	550	59.40	47.70	5.03	0.11	9.26	9.48
GEOROC[1]	North Eastern Anatolia (M)	2011-06-13-04	38.71	42.04	70.67	0.03	4.57	4.81	221	8	129.00	109.00		27.63	0.06	

Source	Location	Sample	Latitude	Longitude	SiO2(wt.%)	MgO(wt.%)	K2O(wt.%)	Na2O(wt.%)	Rb(ppm)	Sr(ppm)	Y(ppm)	La(ppm)	Yb(ppm)	Rb/Sr	Sr/Y	La/Yb
GEOROC[1]	North Eastern Anatolia (M)	2011-06-13-05	38.71	42.04	66.94	0.14	4.70	5.35	143	45	68.00	85.00		3.18	0.66	
GEOROC[1]	North Eastern Anatolia (M)	CU-34	38.73	42.14	46.14	5.90	0.81	3.46	14	513	36.60	20.20	3.41	0.03	14.01	5.92
GEOROC[1]	North Eastern Anatolia (M)	YOZ 92	38.62	42.19	60.31	1.28	3.40	5.00	101	329	49.40	47.20	4.86	0.31	6.67	9.71
GEOROC[1]	North Eastern Anatolia (M)	N-016	38.67	42.20	51.85	3.27	1.94	4.30	44	357	42.63	32.00	4.24	0.12	8.38	7.55
GEOROC[1]	North Eastern Anatolia (M)	N-209	38.67	42.20	53.05	3.07	2.10	4.30	52	333	42.01	31.24	4.09	0.16	7.92	7.64
GEOROC[1]	North Eastern Anatolia (M)	N-240	38.67	42.20	51.46	2.94	2.46	4.61	68	339	65.47	52.48	6.38	0.20	5.18	8.22
GEOROC[1]	North Eastern Anatolia (M)	N-064	38.67	42.20	68.84	0.01	5.11	5.32	185	8	82.41	70.53	9.04	22.37	0.10	7.80
GEOROC[1]	North Eastern Anatolia (M)	N-136	38.67	42.20	59.82	0.91	4.35	6.53	135	211	54.93	51.99	6.37	0.64	3.84	8.17
GEOROC[1]	North Eastern Anatolia (M)	N-141	38.67	42.20	67.77	0.06	4.76	5.27	154	79	80.39	83.03	6.98	1.95	0.98	11.89
GEOROC[1]	North Eastern Anatolia (M)	N-080	38.67	42.20	67.24	0.03	5.01	5.86	103	2	27.78	41.99	4.36	55.26	0.07	9.64
GEOROC[1]	North Eastern Anatolia (M)	N-151	38.67	42.20	68.60	0.02	4.74	5.74	133	3	115.00	88.38	12.20	43.66	0.03	7.24
GEOROC[1]	North Eastern Anatolia (M)	N-162	38.67	42.20	65.37	0.30	5.46	5.69	101	10	52.51	51.66	6.13	10.60	0.18	8.43
GEOROC[1]	North Eastern Anatolia (M)	N-181	38.67	42.20	65.99	0.06	4.95	6.13	120	3	73.08	53.64	7.74	45.58	0.04	6.93
GEOROC[1]	North Eastern Anatolia (M)	N-185	38.67	42.20	68.58	0.04	5.10	5.28	169	11	78.68	67.83	8.62	15.64	0.14	7.87
GEOROC[1]	North Eastern Anatolia (M)	N-239	38.67	42.20	61.59	0.44	5.13	5.82	161	46	77.66	66.94	8.96	3.51	0.59	7.47
GEOROC[1]	North Eastern Anatolia (M)	N-281	38.67	42.20	66.42	0.14	5.09	5.32	113	5	66.46	55.06	7.25	21.64	0.08	7.59
GEOROC[1]	North Eastern Anatolia (M)	N-135	38.67	42.20	71.27	0.00	4.65	5.18	220	2	111.90	118.50	12.61	112.23	0.02	9.40
GEOROC[1]	North Eastern Anatolia (M)	N-227	38.67	42.20	73.66	0.00	4.62	5.06	219	3	105.20	88.75	13.00	84.92	0.02	6.83
GEOROC[1]	North Eastern Anatolia (M)	N-098	38.67	42.20	70.73	0.00	4.37	5.98	234	0	140.30	107.50	15.03	0.00	0.00	7.15
GEOROC[1]	North Eastern Anatolia (M)	N-188	38.67	42.20	66.97	0.09	4.44	5.33	124	20	127.20	96.52	13.80	6.31	0.15	6.99
GEOROC[1]	North Eastern Anatolia (M)	N-022	38.67	42.20	63.73	0.26	4.65	4.68	136	84	56.63	52.16	6.38	1.62	1.49	8.17
GEOROC[1]	North Eastern Anatolia (M)	N-036	38.67	42.20	60.25	0.96	3.72	4.81	109	180	58.76	50.36	6.23	0.61	3.06	8.08
GEOROC[1]	North Eastern Anatolia (M)	N-037	38.67	42.20	61.55	0.56	4.53	6.05	94	138	33.77	34.33	3.80	0.68	4.08	9.05
GEOROC[1]	North Eastern Anatolia (M)	N-046	38.67	42.20	62.40	0.42	4.08	5.86	59	152	33.55	27.81	3.64	0.39	4.52	7.65
GEOROC[1]	North Eastern Anatolia (M)	N-010	38.67	42.20	47.69	6.21	0.88	3.80	19	388	37.36	18.54	3.65	0.05	10.39	5.09
GEOROC[1]	North Eastern Anatolia (M)	N-258	38.67	42.20	46.75	6.49	0.81	3.77	13	395	34.04	16.50	3.20	0.03	11.61	5.16
GEOROC[1]	North Eastern Anatolia (M)	N-264	38.67	42.20	46.91	6.28	0.86	3.95	17	399	37.37	18.40	3.67	0.04	10.67	5.02
GEOROC[1]	North Eastern Anatolia (M)	N-013	38.67	42.20	56.44	3.97	2.17	4.52	84	244	60.62	37.19	6.39	0.34	4.03	5.82
GEOROC[1]	North Eastern Anatolia (M)	N-050	38.67	42.20	57.12	2.26	2.02	5.30	44	337	41.16	28.01	3.96	0.13	8.20	7.08
GEOROC[1]	North Eastern Anatolia (M)	N-256	38.67	42.20	59.51	3.45	2.44	4.53	100	203	67.06	42.35	7.08	0.49	3.02	5.98
GEOROC[1]	North Eastern Anatolia (M)	N-220	38.67	42.20	63.76	0.42	4.62	6.14	95	76	49.19	41.31	5.66	1.24	1.55	7.30
GEOROC[1]	North Eastern Anatolia (M)	N-272	38.67	42.20	60.63	0.63	3.70	5.77	53	186	34.70	26.95	3.57	0.29	5.37	7.55
GEOROC[1]	North Eastern Anatolia (M)	N-014	38.67	42.20	67.35	1.62	3.56	4.86	158	100	90.73	61.08	9.57	1.57	1.11	6.38
GEOROC[1]	North Eastern Anatolia (M)	N-051	38.67	42.20	73.85	0.03	4.50	5.36	195	2	99.45	68.88	10.74	116.04	0.02	6.41
GEOROC[1]	North Eastern Anatolia (M)	N-065	38.67	42.20	74.11	0.00	4.45	4.93	212	0	107.20	72.10	11.69	0.00	0.00	6.17
GEOROC[1]	North Eastern Anatolia (M)	N-131	38.67	42.20	73.61	0.00	4.47	4.94	202	0	102.70	70.18	11.25	0.00	0.00	6.24
GEOROC[1]	North Eastern Anatolia (M)	N-254	38.67	42.20	74.45	0.07	4.47	4.96	200	4	103.00	66.40	11.34	46.85	0.04	5.86
GEOROC[1]	North Eastern Anatolia (M)	N-305	38.67	42.20	74.04	0.02	4.35	5.00	185	2	94.79	64.26	10.68	91.63	0.02	6.02
GEOROC[1]	North Eastern Anatolia (M)	NE13	38.67	42.20	66.93	0.07	5.01	5.76	85	44	72.00	52.40	7.83	1.93	0.61	6.69
GEOROC[1]	North Eastern Anatolia (M)	NE15	38.67	42.20	72.44	0.01	4.61	5.54	85	0	129.00	97.80	14.50	0.00	0.00	6.74
GEOROC[1]	North Eastern Anatolia (M)	NE17	38.67	42.20	72.92	0.01	4.50	5.01	90	0	118.00	65.50	12.00	0.00	0.00	5.46
GEOROC[1]	North Eastern Anatolia (M)	NE19	38.67	42.20	73.84	0.02	4.59	5.13	92	0	107.00			0.00	0.00	
GEOROC[1]	North Eastern Anatolia (M)	NE20	38.67	42.20	73.77	0.03	4.52	5.46	91	0	114.00			0.00	0.00	
GEOROC[1]	North Eastern Anatolia (M)	NE22	38.67	42.20	50.57	3.42	2.33	4.93	50	595	50.00	38.00	4.45	0.08	11.90	8.54

Source	Location	Sample	Latitude	Longitude	SiO2(wt.%)	MgO(wt.%)	K2O(wt.%)	Na2O(wt.%)	Rb(ppm)	Sr(ppm)	Y(ppm)	La(ppm)	Yb(ppm)	Rb/Sr	Sr/Y	La/Yb
GEOROC[1]	North Eastern Anatolia (M)	NE24	38.67	42.20	71.14	0.02	4.75	5.51	92	0	79.00			0.00	0.00	
GEOROC[1]	North Eastern Anatolia (M)	NE26	38.67	42.20	47.50	6.12	0.82	3.67	15	392	40.00	16.70	3.45	0.04	9.80	4.84
GEOROC[1]	North Eastern Anatolia (M)	NE27	38.67	42.20	46.79	7.07	0.74	3.55	8	518	31.00	14.20	2.83	0.02	16.71	5.02
GEOROC[1]	North Eastern Anatolia (M)	Z-31	38.63	42.20	69.02	0.01	4.40	5.26	133	4	131.40	105.70	13.44	32.41	0.03	7.86
GEOROC[1]	North Eastern Anatolia (M)	CU-22A	38.63	42.20	66.44	0.13	4.34	4.85	165	94	80.50	68.90	8.68	1.76	1.16	7.94
GEOROC[1]	North Eastern Anatolia (M)	ZV-6	38.58	42.20	62.39	0.36	5.42	5.76	101	110	37.10	36.60	4.22	0.92	2.96	8.67
GEOROC[1]	North Eastern Anatolia (M)	CU-33	38.74	42.20	67.87	0.03	4.82	5.92	136	3	62.60	59.50	7.17	41.15	0.05	8.30
GEOROC[1]	North Eastern Anatolia (M)	YOZ 65	38.60	42.21	61.65	0.47	5.87	6.49	125	49	41.90	40.80	4.63	2.52	1.18	8.81
GEOROC[1]	North Eastern Anatolia (M)	YOZ 84	38.61	42.21	73.14	0.01	4.75	5.24	238	1	138.10	111.20	13.57	264.89	0.01	8.19
GEOROC[1]	North Eastern Anatolia (M)	Z-37	38.61	42.21	68.40	0.03	4.58	5.56	156	3	91.50	79.40	10.19	45.76	0.04	7.79
GEOROC[1]	North Eastern Anatolia (M)	YOZ 105	38.55	42.21	70.25	0.05	5.54	5.12	188	4	82.40	78.30	8.98	43.63	0.05	8.72
GEOROC[1]	North Eastern Anatolia (M)	NR-25/1	38.57	42.22	53.49	2.62	2.05	4.47	52	365	39.20	32.90	4.50	0.14	9.31	7.31
GEOROC[1]	North Eastern Anatolia (M)	YOZ 81	38.57	42.22	51.75	3.20	1.80	4.19	39	361	43.40	31.30	4.04	0.11	8.32	7.75
GEOROC[1]	North Eastern Anatolia (M)	Z-39	38.60	42.22	75.02	0.01	4.63	4.66	196	3	114.60	110.70	10.45	67.72	0.03	10.59
GEOROC[1]	North Eastern Anatolia (M)	ÖKA-37	38.69	42.22	74.94	0.05	4.77	4.89	223	4	113.90	73.70	11.80	53.07	0.04	6.25
GEOROC[1]	North Eastern Anatolia (M)	NR-14	38.69	42.22	64.23	2.44	3.10	4.92	123	164	70.40	49.50	8.80	0.75	2.33	5.63
GEOROC[1]	North Eastern Anatolia (M)	CU-17	38.65	42.22	46.49	6.25	1.34	3.05	38	494	34.40	23.20	3.00	0.08	14.35	7.73
GEOROC[1]	North Eastern Anatolia (M)	2010-09-03-02	38.70	42.23	72.95	0.03	4.55	4.93	184	12	104.00	90.00		15.33	0.12	
GEOROC[1]	North Eastern Anatolia (M)	CU-22B	38.60	42.23	70.13	0.02	4.94	5.33	189	82	63.70	64.70	6.52	2.32	1.28	9.92
GEOROC[1]	North Eastern Anatolia (M)	NR-12	38.68	42.23	74.11	0.28	4.35	5.12	238	17	91.70	71.50	12.50	14.08	0.18	5.72
GEOROC[1]	North Eastern Anatolia (M)	Z-30	38.60	42.23	68.89	0.03	5.05	4.75	237	2	135.70	124.30	14.95	103.17	0.02	8.31
GEOROC[1]	North Eastern Anatolia (M)	NR-11	38.68	42.23	56.33	4.61	1.95	4.31	71	269	52.10	34.10	6.40	0.27	5.16	5.33
GEOROC[1]	North Eastern Anatolia (M)	ÖKA-23	38.68	42.24	47.00	6.08	0.78	3.47	16	409	37.20	17.50	3.64	0.04	10.99	4.81
GEOROC[1]	North Eastern Anatolia (M)	NR-24	38.56	42.24	61.40	0.99	3.56	5.22	98	217	47.00	44.90	5.60	0.45	4.62	8.02
GEOROC[1]	North Eastern Anatolia (M)	Z-36	38.60	42.24	67.88	0.03	4.51	5.49	151	6	90.50	81.70	11.07	26.93	0.06	7.38
GEOROC[1]	North Eastern Anatolia (M)	NR-10	38.67	42.24	48.10	6.46	0.79	3.71	15	387	32.10	17.60	3.70	0.04	12.06	4.76
GEOROC[1]	North Eastern Anatolia (M)	Z-27	38.62	42.24	72.86	0.02	5.13	4.60	206	4	105.60	71.10	11.60	54.26	0.04	6.13
GEOROC[1]	North Eastern Anatolia (M)	2008-07-06-19	38.56	42.25	68.74	0.01	4.64	4.93	213	8	107.00	96.00		26.63	0.07	
GEOROC[1]	North Eastern Anatolia (M)	2009-07-16-03	38.56	42.25	61.68	0.81	3.74	5.26	105	205	49.00	41.00		0.51	4.18	
GEOROC[1]	North Eastern Anatolia (M)	2009-07-15-01	38.55	42.26	59.04	0.19	4.87	6.68	221	31	107.00	117.00		7.13	0.29	
GEOROC[1]	North Eastern Anatolia (M)	YOZ 19	38.61	42.26	74.61	0.01	4.89	4.80	217	2	110.90	71.50	11.24	127.88	0.02	6.36
GEOROC[1]	North Eastern Anatolia (M)	2011-06-05-02	38.53	42.26	62.78	0.21	4.68	5.83	63	122	32.00	23.00		0.52	3.81	
GEOROC[1]	North Eastern Anatolia (M)	NR-23	38.57	42.27	72.84	0.06	4.26	5.41	285	1	153.00	134.00	16.30	570.00	0.00	8.22
GEOROC[1]	North Eastern Anatolia (M)	CU-1	38.67	42.27	61.16	0.73	3.59	5.28	95	236	54.40	42.00	5.50	0.40	4.33	7.64
GEOROC[1]	North Eastern Anatolia (M)	NR-1	38.63	42.28	73.81	0.06	4.43	5.20	236	3	91.70	73.20	11.90	90.77	0.03	6.15
GEOROC[1]	North Eastern Anatolia (M)	ÖKA15	38.72	42.28	64.31	0.13	5.04	5.86	215	12	77.70	79.10	10.62	17.74	0.16	7.45
GEOROC[1]	North Eastern Anatolia (M)	YOZ 7	38.58	42.28	72.85	0.01	5.11	5.17	236	2	119.60	100.40	12.55	131.00	0.02	8.00
GEOROC[1]	North Eastern Anatolia (M)	ÖKA-5	38.69	42.30	63.31	0.44	4.57	6.28	93	176	36.30	35.60	3.79	0.53	4.86	9.39
GEOROC[1]	North Eastern Anatolia (M)	ÖKA-20	38.70	42.30	72.46	0.03	4.75	5.33	214	2	116.30	89.30	12.10	101.95	0.02	7.38
GEOROC[1]	North Eastern Anatolia (M)	CU-11	38.62	42.30	70.15	0.02	4.42	5.52	249	2	150.00	109.60	15.03	108.09	0.02	7.29
GEOROC[1]	North Eastern Anatolia (M)	ÖKA-8	38.68	42.32	62.99	0.46	4.65	6.12	102	164	38.70	37.50	4.52	0.62	4.23	8.30
GEOROC[1]	North Eastern Anatolia (M)	Z-11	38.59	42.35	68.45	0.03	5.11	5.64	211	2	64.20	62.00	12.14	95.91	0.03	5.11
GEOROC[1]	North Eastern Anatolia (M)	Z-12	38.55	42.35	58.58	1.72	3.75	5.40	91	340	55.10	48.10	5.94	0.27	6.17	8.10
GEOROC[1]	North Eastern Anatolia (M)	2011-06-12-02	38.81	42.37	59.85	0.28	5.24	6.59	149	14	46.00	54.00		10.64	0.30	

Source	Location	Sample	Latitude	Longitude	SiO2(wt.%)	MgO(wt.%)	K2O(wt.%)	Na2O(wt.%)	Rb(ppm)	Sr(ppm)	Y(ppm)	La(ppm)	Yb(ppm)	Rb/Sr	Sr/Y	La/Yb
GEOROC[1]	North Eastern Anatolia (M)	2009-07-14-08	38.49	42.37	67.92	0.04	4.71	5.05	228	10	126.00	115.00		22.80	0.08	
GEOROC[1]	North Eastern Anatolia (M)	2008-07-06-17	38.57	42.38	62.94	0.31	5.11	6.76	110	91	42.00	41.00		1.21	2.17	
GEOROC[1]	North Eastern Anatolia (M)	2009-07-14-05b	38.48	42.38	67.34	0.07	4.79	5.04	180	27	98.00	85.00		6.67	0.28	
GEOROC[1]	North Eastern Anatolia (M)	2008-07-05-01	38.60	42.38	60.46	0.14	5.11	6.40	181	4	64.00	72.00		45.25	0.06	
GEOROC[1]	North Eastern Anatolia (M)	2010-08-25-32	38.77	42.40	66.56	0.20	4.31	4.78	203	10	122.00	92.00		20.30	0.08	
GEOROC[1]	North Eastern Anatolia (M)	2009-07-18-06	38.77	42.40	67.15	0.01	4.53	5.12	211	8	138.00	104.00		26.38	0.06	
GEOROC[1]	North Eastern Anatolia (M)	2009-07-18-08	38.77	42.40	68.84	0.05	5.55	4.08	211	4	91.00	89.00		52.75	0.04	
GEOROC[1]	North Eastern Anatolia (M)	2009-07-14-04A	38.45	42.40	64.80	0.06	5.55	4.61	153	49	67.00	80.00		3.12	0.73	
GEOROC[1]	North Eastern Anatolia (M)	2009-07-19-02	38.75	42.42	68.08	0.02	4.50	4.98	183	9	112.00	97.00		20.33	0.08	
GEOROC[1]	North Eastern Anatolia (M)	2009-07-20-02	38.79	42.43	62.23	0.04	5.19	5.02	223	16	100.00	109.00		13.94	0.16	
GEOROC[1]	North Eastern Anatolia (M)	2009-07-20-01	38.79	42.43	61.60	0.05	5.27	5.20	193	20	82.00	100.00		9.65	0.24	
GEOROC[1]	North Eastern Anatolia (M)	2011-06-15-01	38.63	42.44	70.64	0.01	4.35	4.87	221	3	129.00	138.00		73.67	0.02	
GEOROC[1]	North Eastern Anatolia (M)	2010-08-23-12	38.77	42.45	63.66	0.10	5.31	4.77	138	79	62.00	60.00		1.75	1.27	
GEOROC[1]	North Eastern Anatolia (M)	2010-08-23-15	38.77	42.45	59.68	0.17	4.99	5.29	116	44	63.00	52.00		2.64	0.70	
GEOROC[1]	North Eastern Anatolia (M)	2011-06-08-08	38.77	42.47	69.74	0.02	4.45	4.85	206	2	127.00	115.00		103.00	0.02	
GEOROC[1]	North Eastern Anatolia (M)	2011-06-14-05	38.78	42.50	63.56	0.08	5.67	5.00	186	13	67.00	76.00		14.31	0.19	
GEOROC[1]	North Eastern Anatolia (M)	2011-06-14-03	38.81	42.51	58.47	0.35	4.96	6.96	170	31	71.00	74.00		5.48	0.44	
GEOROC[1]	North Eastern Anatolia (M)	V-3A	38.00	43.00	72.52	0.08	4.66	4.79	186	5	99.00	73.00		37.20	0.05	
GEOROC[1]	North Eastern Anatolia (E)	RK 4	38.00	44.30	48.23	4.67	2.99	5.13	50	1983	28.00	82.02	2.25	0.03	70.82	36.45
GEOROC[1]	North Eastern Anatolia (E)	MU 14.19	38.24	44.34	52.21	6.58	2.73	4.24	65	839	24.00	61.94	2.04	0.08	34.96	30.36
GEOROC[1]	North Eastern Anatolia (E)	MU 15.20	38.24	44.34	48.11	11.46	2.01	3.40	47	1113	24.00	66.11	1.89	0.04	46.38	34.98
GEOROC[1]	North Eastern Anatolia (E)	MU 15.21	38.24	44.34	48.71	9.92	2.22	3.75	58	1125	24.00	72.00		0.05	46.88	
GEOROC[1]	North Eastern Anatolia (E)	MU 15.22	38.24	44.34	59.17	2.23	3.93	4.78	128	916	20.00	73.00		0.14	45.80	
GEOROC[1]	North Eastern Anatolia (E)	MU 16.23	38.24	44.34	46.00	8.47	1.99	3.94	44	1066	28.00	61.25	2.29	0.04	38.07	26.75
GEOROC[1]	North Eastern Anatolia (E)	TE01	39.20	44.00	58.53	1.45	3.71	6.90	99	291	55.00	54.50	5.86	0.34	5.29	9.30
GEOROC[1]	North Eastern Anatolia (E)	TE02	39.20	44.00	51.76	4.41	1.44	4.93	23	629	38.00	35.70	3.26	0.04	16.55	10.95
GEOROC[1]	North Eastern Anatolia (E)	MU 11.16	39.20	44.00	50.43	3.85	1.59	5.61	20	581	38.00	37.07	3.31	0.03	15.29	11.20
GEOROC[1]	North Eastern Anatolia (E)	MU 12.17	39.20	44.00	50.10	4.21	1.63	5.39	25	652	38.00	39.86	3.41	0.04	17.16	11.69
GEOROC[1]	North Eastern Anatolia (E)	31	39.24	44.01	51.00	4.10	1.65	5.22	23	504	27.86	44.24	2.94	0.05	18.08	15.05
GEOROC[1]	North Eastern Anatolia (E)	28	39.47	44.11	53.50	2.88	2.13	5.31	37	525	33.60	57.01	3.91	0.07	15.62	14.58
GEOROC[1]	North Eastern Anatolia (E)	MU 13.18	39.07	44.39	49.03	6.16	1.36	3.54	9	1724	27.00	54.83	2.15	0.01	63.85	25.50
GEOROC[1]	North Eastern Anatolia (E)	AR01	39.75	44.40	63.83	1.64	1.85	5.03	41	389	23.00	26.00	2.06	0.11	16.91	12.62
GEOROC[1]	North Eastern Anatolia (E)	AR02	39.75	44.40	62.64	2.33	2.15	4.92	56	381	26.00	27.50	2.48	0.15	14.65	11.09
GEOROC[1]	North Eastern Anatolia (E)	AR11	39.75	44.40	58.73	3.36	1.81	4.41	37	446	33.00	24.40	2.91	0.08	13.52	8.38
GEOROC[1]	North Eastern Anatolia (E)	119	39.75	44.40	51.50	3.80	0.60	4.50	9	552	29.00			0.02	19.03	
GEOROC[1]	North Eastern Anatolia (E)	110	39.75	44.40	61.30	1.70	1.70	4.80	42	512	21.00			0.08	24.38	
GEOROC[1]	North Eastern Anatolia (E)	117	39.75	44.40	63.00	2.80	1.70	4.30	55	465	19.00			0.12	24.47	
GEOROC[1]	North Eastern Anatolia (E)	106	39.75	44.40	66.00	2.10	1.40	4.20	48	480	12.00			0.10	40.00	
GEOROC[1]	North Eastern Anatolia (E)	104	39.75	44.40	69.70	0.60	1.80	4.20	58	497	11.00			0.12	45.18	
GEOROC[1]	North Eastern Anatolia (E)	105	39.75	44.40	69.90	1.00	1.80	3.80	58	492	13.00			0.12	37.85	
GEOROC[1]	North Eastern Anatolia (E)	97	39.75	44.40	69.90	0.60	1.80	4.30	67	489	10.00			0.14	48.90	
GEOROC[1]	North Eastern Anatolia (E)	103	39.75	44.40	70.00	0.90	1.80	4.20	51	482	11.00			0.11	43.82	
GEOROC[1]	North Eastern Anatolia (E)	98	39.75	44.40	70.50	0.80	1.90	4.30	65	482	11.00			0.13	43.82	
GEOROC[1]	North Eastern Anatolia (E)	115	39.75	44.40	72.00	1.00	1.80	4.00	59	439	9.00			0.13	48.78	

Source	Location	Sample	Latitude	Longitude	SiO2(wt.%)	MgO(wt.%)	K2O(wt.%)	Na2O(wt.%)	Rb(ppm)	Sr(ppm)	Y(ppm)	La(ppm)	Yb(ppm)	Rb/Sr	Sr/Y	La/Yb
GEOROC[1]	North Eastern Anatolia (E)	116	39.75	44.40	72.40	1.20	1.90	3.90	64	440	8.00			0.15	55.00	
GEOROC[1]	North Eastern Anatolia (E)	109	39.75	44.40	61.50	2.00	1.90	4.60	58	485	31.00			0.12	15.65	
GEOROC[1]	North Eastern Anatolia (E)	112	39.75	44.40	61.80	2.00	1.90	4.70	60	492	27.00			0.12	18.22	
GEOROC[1]	North Eastern Anatolia (E)	108A	39.75	44.40	62.50	1.60	1.80	4.80	53	453	25.00			0.12	18.12	
GEOROC[1]	North Eastern Anatolia (E)	93	39.75	44.40	62.70	1.70	2.00	4.70	62	473	25.00			0.13	18.92	
GEOROC[1]	North Eastern Anatolia (E)	108B	39.75	44.40	62.80	1.50	1.80	4.90	55	447	24.00			0.12	18.63	
GEOROC[1]	North Eastern Anatolia (E)	90	39.75	44.40	63.10	1.80	2.10	4.60	66	463	26.00			0.14	17.81	
GEOROC[1]	North Eastern Anatolia (E)	118	39.75	44.40	63.10	1.80	1.70	4.80	49	469	24.00			0.10	19.54	
GEOROC[1]	North Eastern Anatolia (E)	94	39.75	44.40	66.60	1.10	2.10	4.40	69	406	23.00			0.17	17.65	
GEOROC[1]	North Eastern Anatolia (E)	88	39.75	44.40	66.70	1.10	2.20	4.40	62	423	25.00			0.15	16.92	
GEOROC[1]	North Eastern Anatolia (E)	89	39.75	44.40	67.20	1.20	2.20	4.60	63	416	23.00			0.15	18.09	
GEOROC[1]	North Eastern Anatolia (E)	95	39.75	44.40	68.30	1.10	2.10	4.40	67	312	20.00			0.21	15.60	
GEOROC[1]	North Eastern Anatolia (E)	101	39.75	44.40	69.30	0.70	2.20	4.70	68	323	21.00			0.21	15.38	
GEOROC[1]	North Eastern Anatolia (E)	100	39.75	44.40	69.60	0.70	2.30	4.60	72	311	22.00			0.23	14.14	
GEOROC[1]	North Eastern Anatolia (E)	102	39.75	44.40	70.70	0.60	2.60	4.60	74	307	22.00			0.24	13.95	
GEOROC[1]	North Eastern Anatolia (E)	113	39.75	44.40	75.00	0.20	3.30	4.80	104	121	27.00			0.86	4.48	
GEOROC[1]	North Eastern Anatolia (E)	MU 2.1	39.75	44.40	50.38	6.01	0.79	4.83	11	564	32.00	15.00		0.02	17.63	
GEOROC[1]	North Eastern Anatolia (E)	MU 3.9	39.75	44.40	50.19	5.30	0.64	4.92	9	492	31.00	15.00		0.02	15.87	
GEOROC[1]	North Eastern Anatolia (E)	MU 5.10	39.75	44.40	49.81	6.10	0.71	4.65	11	519	27.00	16.00		0.02	19.22	
GEOROC[1]	North Eastern Anatolia (E)	MU 6.11	39.75	44.40	52.45	6.68	0.99	4.16	19	364	25.00	16.08	2.25	0.05	14.56	7.15
GEOROC[1]	North Eastern Anatolia (E)	MU 7.12	39.75	44.40	54.95	5.64	1.29	4.45	31	387	29.00	20.00		0.08	13.34	
GEOROC[1]	North Eastern Anatolia (E)	MU 8.13	39.75	44.40	55.43	5.93	1.12	4.27	28	376	23.00	17.00		0.07	16.35	
GEOROC[1]	North Eastern Anatolia (E)	MU 9.14	39.75	44.40	53.67	6.05	1.18	4.14	29	376	26.00	19.00		0.08	14.46	
GEOROC[1]	North Eastern Anatolia (E)	MU 10.15	39.75	44.40	50.35	7.57	1.03	4.50	16	658	31.00	27.00		0.02	21.23	
GEOROC[1]	North Eastern Anatolia (E)	MU 17.24	39.75	44.40	51.40	5.31	0.68	5.02	10	460	37.00	15.00		0.02	12.43	
GEOROC[1]	North Eastern Anatolia (E)	MU 18.25	39.75	44.40	48.95	5.16	0.55	4.49	7	523	27.00	11.05	2.40	0.01	19.37	4.60
GEOROC[1]	North Eastern Anatolia (E)	MU 20.26	39.75	44.40	49.64	6.93	1.06	4.51	15	571	29.00	20.79	2.61	0.03	19.69	7.97
GEOROC[1]	Northeastern Central Anatolia	M1	39.40	38.00	46.52	7.59	0.74	2.79	13	508	26.60	10.90	2.40	0.03	19.10	4.54
GEOROC[1]	Northeastern Central Anatolia	M2	39.40	38.00	46.32	7.60	0.69	2.84	14	516	26.90	11.00	2.75	0.03	19.20	4.00
GEOROC[1]	Northeastern Central Anatolia	M3	39.40	38.00	47.64	7.66	0.51	3.17	12	552	30.80	11.70	3.00	0.02	17.92	3.90
GEOROC[1]	Northeastern Central Anatolia	M3A	39.40	38.00	45.47	4.15	0.54	3.22	10	354	25.10	10.70	2.79	0.03	14.10	3.84
GEOROC[1]	Northeastern Central Anatolia	M6	39.40	38.00	47.41	4.85	0.55	3.41	11	351	27.90	11.00	2.34	0.03	12.59	4.70
GEOROC[1]	Northeastern Central Anatolia	M7	39.40	38.00	47.80	4.41	0.56	3.45	11	356	26.20	10.40	2.66	0.03	13.57	3.91
GEOROC[1]	Northeastern Central Anatolia	M8	39.40	38.00	46.00	5.19	0.55	3.40	12	344	27.00	11.00	2.53	0.03	12.75	4.35
GEOROC[1]	Northeastern Central Anatolia	M9	39.40	38.00	48.84	4.89	0.76	3.53	16	391	27.80	15.70	2.90	0.04	14.07	5.41
GEOROC[1]	Northeastern Central Anatolia	M10	39.40	38.00	49.10	4.82	0.76	3.55	15	388	26.70	15.90	2.57	0.04	14.53	6.19
GEOROC[1]	Northeastern Central Anatolia	M11	39.40	38.00	48.09	4.49	0.57	3.38	12	347	28.50	12.10	2.71	0.03	12.17	4.46
GEOROC[1]	Northeastern Central Anatolia	M12	39.40	38.00	45.22	4.87	0.51	3.10	9	334	26.70	10.90	2.55	0.03	12.49	4.27
GEOROC[1]	Northeastern Central Anatolia	M13	39.40	38.00	47.09	4.71	0.69	3.34	14	416	28.60	15.30	2.56	0.03	14.56	5.98
GEOROC[1]	Northeastern Central Anatolia	M14	39.40	38.00	48.59	5.62	0.74	3.46	18	404	30.20	19.50	2.85	0.04	13.37	6.84
GEOROC[1]	Northeastern Central Anatolia	M15	39.40	38.00	48.91	5.33	0.52	3.50	11	372	27.50	11.60	2.68	0.03	13.51	4.33
GEOROC[1]	Northeastern Central Anatolia	M16	39.40	38.00	48.52	5.91	0.54	3.51	12	361	27.00	11.30	2.60	0.03	13.36	4.35
GEOROC[1]	Northeastern Central Anatolia	M17	39.40	38.00	54.42	4.94	1.11	3.60	37	354	24.30	16.70	2.07	0.10	14.56	8.07

Source	Location	Sample	Latitude	Longitude	SiO2(wt.%)	MgO(wt.%)	K2O(wt.%)	Na2O(wt.%)	Rb(ppm)	Sr(ppm)	Y(ppm)	La(ppm)	Yb(ppm)	Rb/Sr	Sr/Y	La/Yb
GEOROC[1]	Northeastern Central Anatolia	M18	39.40	38.00	54.99	4.50	1.18	3.75	37	362	24.20	17.80	2.30	0.10	14.98	7.74
GEOROC[1]	Northeastern Central Anatolia	M19	39.40	38.00	50.30	6.36	1.31	3.97	33	536	25.90	26.30	2.41	0.06	20.70	10.91
GEOROC[1]	Northeastern Central Anatolia	M20	39.40	38.00	50.71	5.23	1.38	4.05	36	569	24.90	26.50	2.24	0.06	22.85	11.83
GEOROC[1]	Northeastern Central Anatolia	M21	39.40	38.00	54.34	4.82	1.13	3.74	33	361	23.10	26.10	2.51	0.09	15.61	10.40
GEOROC[1]	Northeastern Central Anatolia	M22	39.40	38.00	55.07	4.85	1.20	3.70	33	3552	25.70	19.30	2.60	0.01	138.21	7.42
GEOROC[1]	Northeastern Central Anatolia	M23	39.40	38.00	49.44	4.64	1.19	4.05	23	529	28.70	22.10	2.56	0.04	18.44	8.63
GEOROC[1]	Northeastern Central Anatolia	M24	39.40	38.00	49.89	4.43	1.22	4.17	25	531	29.40	23.20	2.58	0.05	18.05	8.99
GEOROC[1]	Northeastern Central Anatolia	M27	39.40	38.00	45.21	3.10	0.64	3.30	16	387	27.60	13.30	2.52	0.04	14.02	5.28
GEOROC[1]	Northeastern Central Anatolia	M29	39.40	38.00	48.89	5.37	0.65	3.12	18	371	24.90	13.20	2.73	0.05	14.90	4.84
GEOROC[1]	Northeastern Central Anatolia	M30	39.40	38.00	46.39	3.98	0.70	3.50	18	403	28.40	10.40	2.77	0.04	14.18	3.75
GEOROC[1]	Northeastern Central Anatolia	E3	38.90	38.57	47.65	7.85	0.54	3.07	11	306	24.70	16.10		0.04	12.39	
GEOROC[1]	Northeastern Central Anatolia	E5	38.90	38.57	47.60	7.51	0.55	3.11	11	302	24.70	17.00		0.04	12.23	
GEOROC[1]	Northeastern Central Anatolia	IL2	39.60	39.50	56.70	2.63	3.21	4.65	59	504	27.90	41.30	2.77	0.12	18.06	14.91
GEOROC[1]	Northeastern Central Anatolia	IL3	39.60	39.50	56.77	2.65	2.66	4.65	56	542	27.20	41.90	2.67	0.10	19.91	15.69
GEOROC[1]	Northeastern Central Anatolia	IL6	39.60	39.50	56.80	2.70	2.81	4.80	51	525	26.10	37.50	2.68	0.10	20.13	13.99
GEOROC[1]	Northeastern Central Anatolia	KT3	39.60	39.50	62.60	2.70	3.05	3.98	74	341	17.90	33.40	1.68	0.22	19.06	19.88
GEOROC[1]	Northeastern Central Anatolia	KT4	39.60	39.50	62.80	2.22	3.11	3.91	95	294	19.20	37.20	1.52	0.32	15.31	24.47
GEOROC[1]	Northeastern Central Anatolia	YT2	39.60	39.50	62.69	2.44	3.23	3.83	83	301	18.20	35.00	1.78	0.28	16.53	19.66
GEOROC[1]	Northeastern Central Anatolia	IR2	39.60	39.50	62.99	2.33	2.93	3.89	91	268	19.00	37.40	1.56	0.34	14.13	23.97
GEOROC[1]	Northeastern Central Anatolia	IR3	39.60	39.50	62.90	2.34	2.88	3.96	91	282	18.10	35.30	1.40	0.32	15.55	25.21
GEOROC[1]	Northeastern Central Anatolia	IR5	39.60	39.50	62.79	2.35	3.09	3.95	80	273	17.40	32.90	1.61	0.29	15.70	20.43
GEOROC[1]	Northeastern Central Anatolia	IR8	39.60	39.50	62.46	2.41	3.09	4.02	83	281	18.90	33.50	1.68	0.30	14.85	19.94
GEOROC[1]	Northeastern Central Anatolia	IR9	39.60	39.50	62.96	2.30	3.09	4.02	83	288	17.80	34.60	1.69	0.29	16.16	20.47
GEOROC[1]	Northeastern Central Anatolia	IR10	39.60	39.50	62.81	2.33	3.01	3.95	84	288	17.80	33.70	1.90	0.29	16.17	17.74
GEOROC[1]	Northeastern Central Anatolia	IR4A	39.60	39.50	63.70	2.26	3.24	3.84	89	274	17.00	34.60	1.60	0.32	16.14	21.63
GEOROC[1]	Northeastern Central Anatolia	IR7	39.60	39.50	63.60	2.41	3.04	4.00	85	294	18.50	35.20	1.90	0.29	15.90	18.53
GEOROC[1]	Northeastern Central Anatolia	IR11	39.60	39.50	63.20	2.32	3.00	4.01	81	281	17.90	34.00	1.74	0.29	15.68	19.54
GEOROC[1]	Northeastern Central Anatolia	YT3	39.60	39.50	63.90	1.87	3.20	4.11	89	276	18.00	36.70	1.82	0.32	15.33	20.16
GEOROC[1]	Northeastern Central Anatolia	PT1	39.60	39.50	69.40	0.58	3.87	4.02	113	262	12.70	35.70	1.00	0.43	20.66	35.70
GEOROC[1]	Northeastern Central Anatolia	AT2	39.60	39.50	69.77	0.56	3.87	4.17	97	283	9.50	36.20	0.98	0.34	29.77	36.94
GEOROC[1]	Northeastern Central Anatolia	AT3	39.60	39.50	70.76	0.55	3.52	4.10	93	265	9.10	31.40	0.91	0.35	29.12	34.51
GEOROC[1]	Northeastern Central Anatolia	T1	39.60	39.50	70.74	0.57	3.59	4.03	109	283	9.70	34.00	0.89	0.39	29.13	38.20
GEOROC[1]	Northeastern Central Anatolia	T2	39.60	39.50	72.03	0.54	3.52	4.22	99	258	9.30	31.90	0.96	0.38	27.70	33.23
GEOROC[1]	Northeastern Central Anatolia	T3	39.60	39.50	70.69	0.57	3.60	4.04	111	291	9.90	35.20	0.89	0.38	29.41	39.55
GEOROC[1]	Northeastern Central Anatolia	PT2	39.60	39.50	70.56	0.59	3.70	3.98	105	277	11.50	34.00	1.11	0.38	24.09	30.63
GEOROC[1]	Northeastern Central Anatolia	MT2	39.60	39.50	73.21	0.03	4.69	3.96	191	20	23.50	10.50	2.51	9.69	0.84	4.18
GEOROC[1]	Northeastern Central Anatolia	MT3	39.60	39.50	73.55	0.01	4.60	4.11	170	19	22.60	11.60	2.32	8.94	0.84	5.00
GEOROC[1]	Northeastern Central Anatolia	CT1	39.60	39.50	74.08	0.05	4.81	4.44	126	109	12.10	43.90	1.26	1.16	8.97	34.84
GEOROC[1]	Northeastern Central Anatolia	CT7	39.60	39.50	73.84	0.05	4.64	3.90	180	14	16.90	12.30	1.61	12.96	0.82	7.64
GEOROC[1]	Northeastern Central Anatolia	ST1	39.60	39.50	73.47	0.20	4.84	3.90	142	58	12.50	34.50	1.44	2.46	4.62	23.96
GEOROC[1]	Northeastern Central Anatolia	ST2	39.60	39.50	72.67	0.19	5.32	3.79	154	62	12.40	42.70	1.25	2.49	4.98	34.16
GEOROC[1]	Northeastern Central Anatolia	KU1	39.60	39.50	73.02	0.13	4.23	3.86	131	170	9.10	41.00	0.84	0.77	18.69	48.81
GEOROC[1]	Northeastern Central Anatolia	AG2	39.60	39.50	73.39	0.13	4.19	3.92	117	165	8.60	38.70	0.89	0.71	19.19	43.48

Source	Location	Sample	Latitude	Longitude	SiO2(wt.%)	MgO(wt.%)	K2O(wt.%)	Na2O(wt.%)	Rb(ppm)	Sr(ppm)	Y(ppm)	La(ppm)	Yb(ppm)	Rb/Sr	Sr/Y	La/Yb
GEOROC[1]	South Central Anatolia (W)	KP15	37.50	33.50	47.62	10.72	0.84	2.99	17	569	24.00	27.80	2.30	0.03	23.71	12.09
GEOROC[1]	South Central Anatolia (W)	KP16	37.50	33.50	49.81	10.47	0.93	3.18	20	566	24.00	29.50	2.24	0.04	23.58	13.17
GEOROC[1]	South Central Anatolia (W)	KP17	37.50	33.50	52.29	7.86	1.19	3.36	25	606	24.00	34.60	2.17	0.04	25.25	15.94
GEOROC[1]	South Central Anatolia (W)	KP18	37.50	33.50	56.38	6.40	2.09	3.51	58	669	21.00	40.80	2.14	0.09	31.86	19.07
GEOROC[1]	South Central Anatolia (W)	C94-1	37.71	33.58	52.50	7.21	1.38	3.33	32	546	16.84	39.84	1.86	0.06	32.42	21.42
GEOROC[1]	South Central Anatolia (W)	C94-2	37.71	33.67	48.80	7.21	0.92	3.09	20	549	16.08	18.28	1.78	0.04	34.12	10.27
GEOROC[1]	South Central Anatolia (W)	KT11	38.20	34.00	51.84	6.58	1.57	4.00	30	619	28.00	29.00	2.25	0.05	22.11	12.89
GEOROC[1]	South Central Anatolia (W)	KT12	38.20	34.00	48.97	7.38	1.09	3.44	17	624	27.00	29.30	2.41	0.03	23.11	12.16
GEOROC[1]	South Central Anatolia (W)	KT13	38.20	34.00	48.50	7.16	1.00	3.34	19	605	27.00	29.50	2.29	0.03	22.41	12.88
GEOROC[1]	South Central Anatolia (W)	KT14	38.20	34.00	48.83	7.77	1.05	3.55	18	614	27.00	29.60	2.38	0.03	22.74	12.44
GEOROC[1]	South Central Anatolia (W)	KT16	38.20	34.00	50.57	8.14	1.20	3.46	21	606	27.00	30.20	2.44	0.03	22.44	12.38
GEOROC[1]	South Central Anatolia (W)	EG12	37.80	34.00	48.52	10.20	1.03	3.08	16	697	22.00	20.30	2.05	0.02	31.68	9.90
GEOROC[1]	South Central Anatolia (W)	EG14	37.80	34.00	49.11	10.59	0.86	2.96	13	783	17.00	24.90	1.67	0.02	46.06	14.91
GEOROC[1]	South Central Anatolia (W)	EG15	37.80	34.00	50.38	8.10	1.10	3.57	13	535	26.00	20.20	2.46	0.02	20.58	8.21
GEOROC[1]	South Central Anatolia (W)	EG16	37.80	34.00	47.73	7.97	0.99	3.49	13	503	27.00	17.80	2.24	0.03	18.63	7.95
GEOROC[1]	South Central Anatolia (W)	EG17	37.80	34.00	49.67	7.52	1.12	3.41	13	659	27.00	25.60	2.18	0.02	24.41	11.74
GEOROC[1]	South Central Anatolia (W)	EG18	37.80	34.00	49.46	8.10	1.52	3.56	26	743	26.00	31.80	2.12	0.03	28.58	15.00
GEOROC[1]	South Central Anatolia (W)	EG19	37.80	34.00	50.21	7.51	1.33	3.77	22	572	27.00	24.10	2.28	0.04	21.19	10.57
GEOROC[1]	South Central Anatolia (W)	EG21	37.80	34.00	48.82	7.90	1.48	3.60	26	715	27.00	31.50	2.27	0.04	26.48	13.88
GEOROC[1]	South Central Anatolia (W)	EG22	37.80	34.00	48.46	10.21	0.87	3.45	13	574	25.00			0.02	22.96	
GEOROC[1]	South Central Anatolia (W)	C94-5	37.98	34.12	49.57	7.75	1.04	3.07	14	486	19.00	21.42	2.16	0.03	25.60	9.92
GEOROC[1]	South Central Anatolia (W)	88-24	38.13	34.18	62.33	2.74	1.87	3.97	62	364	20.00			0.17	18.20	
GEOROC[1]	South Central Anatolia (W)	90-62	38.13	34.18	67.19	1.18	2.94	3.86	96	243	18.00	30.50	1.43	0.40	13.50	21.33
GEOROC[1]	South Central Anatolia (W)	90-1	38.13	34.18	53.00	6.19	1.47	4.08	32	580	27.00	29.90	2.07	0.06	21.48	14.44
GEOROC[1]	South Central Anatolia (W)	88-30	38.13	34.18	51.50	7.64	1.25	3.69	25	559	25.00	34.60	2.46	0.04	22.36	14.07
GEOROC[1]	South Central Anatolia (W)	89-73	38.13	34.18	48.03	6.15	0.96	3.64	20	575	27.00	35.70	2.50	0.03	21.30	14.28
GEOROC[1]	South Central Anatolia (W)	90-5	38.13	34.18	60.30	3.13	1.90	3.60	58	443	18.00	24.80	1.78	0.13	24.61	13.93
GEOROC[1]	South Central Anatolia (W)	90-36	38.13	34.18	64.04	2.75	1.97	3.83	66	350	21.00	31.30	2.03	0.19	16.67	15.42
GEOROC[1]	South Central Anatolia (W)	89-74	38.13	34.18	76.41	0.22	3.71	4.45	115	89	17.00	31.60	1.56	1.29	5.24	20.26
GEOROC[1]	South Central Anatolia (W)	90-12	38.13	34.18	71.60	0.65	3.18	4.20	104	199	16.00			0.52	12.44	
GEOROC[1]	South Central Anatolia (W)	90-64	38.13	34.18	71.78	0.63	3.30	4.04	91	264	17.00			0.34	15.53	
GEOROC[1]	South Central Anatolia (W)	88-28	38.13	34.18	51.90	6.38	1.64	4.20	31	581	27.00			0.05	21.52	
GEOROC[1]	South Central Anatolia (W)	89-67	38.13	34.18	62.80	2.57	2.24	4.17	71	417	19.00			0.17	21.95	
GEOROC[1]	South Central Anatolia (W)	90-40	38.13	34.18	64.86	2.29	2.24	3.79	70	332	20.00			0.21	16.60	
GEOROC[1]	South Central Anatolia (W)	90-45	38.13	34.18	64.68	2.13	2.22	3.54	74	312	19.00			0.24	16.42	
GEOROC[1]	South Central Anatolia (W)	90-52	38.13	34.18	63.79	2.74	1.87	3.92	66	369	19.00			0.18	19.42	
GEOROC[1]	South Central Anatolia (W)	90-65	38.13	34.18	75.29	0.12	4.07	4.13	121	64	15.00			1.89	4.27	
GEOROC[1]	South Central Anatolia (W)	89-38	38.13	34.18	67.67	1.21	3.03	4.18	98	258	16.00			0.38	16.13	
GEOROC[1]	South Central Anatolia (W)	89-62	38.13	34.18	53.10	5.93	1.33	3.35	34	846	24.00	41.50	2.14	0.04	35.25	19.39
GEOROC[1]	South Central Anatolia (W)	88-23	38.13	34.18	73.75	0.19	3.85	3.99	123	60	14.00			2.05	4.29	
GEOROC[1]	South Central Anatolia (W)	89-24	38.13	34.18	66.00	2.13	2.37	4.30	79	332	19.00			0.24	17.47	
GEOROC[1]	South Central Anatolia (W)	89-65	38.13	34.18	51.43	6.80	1.60	4.12	30	617	27.00			0.05	22.85	
GEOROC[1]	South Central Anatolia (W)	89-66	38.13	34.18	50.25	7.40	1.36	3.72	29	612	28.00	37.10	2.44	0.05	21.86	15.20
GEOROC[1]	South Central Anatolia (W)	HA01	38.13	34.18	49.61	6.01	1.02	4.21	19	612	27.00	28.40	2.47	0.03	22.67	11.50

Source	Location	Sample	Latitude	Longitude	SiO2(wt.%)	MgO(wt.%)	K2O(wt.%)	Na2O(wt.%)	Rb(ppm)	Sr(ppm)	Y(ppm)	La(ppm)	Yb(ppm)	Rb/Sr	Sr/Y	La/Yb
GEOROC[1]	South Central Anatolia (W)	U-357	38.10	34.20	72.62	0.35	5.19	2.49	194	120	5.00			1.61	24.00	
GEOROC[1]	South Central Anatolia (W)	U-517	38.10	34.20	73.43	0.33	4.67	3.08	193	127	9.10	31.26	1.31	1.52	14.00	23.86
GEOROC[1]	South Central Anatolia (W)	U-543	38.10	34.20	65.95	1.23	3.62	3.64	114	227	36.40			0.50	6.25	
GEOROC[1]	South Central Anatolia (W)	U-545	38.10	34.20	66.24	0.95	3.39	3.82	115	215	36.50			0.54	5.89	
GEOROC[1]	South Central Anatolia (W)	U-416	38.10	34.20	72.83	0.10	4.38	3.86	208	64	18.10			3.24	3.56	
GEOROC[1]	South Central Anatolia (W)	U-513	38.10	34.20	73.09	0.01	4.48	3.66	209	67	121.40	26.89	2.73	3.13	0.55	9.85
GEOROC[1]	South Central Anatolia (W)	C94-12	37.92	34.21	50.50	8.38	1.18	3.53	22	496	16.62	20.89	1.83	0.04	29.87	11.42
GEOROC[1]	South Central Anatolia (W)	C94-15 (1)	37.98	34.25	55.75	3.73	2.12	3.50	41	473	18.13	28.30	2.08	0.09	26.07	13.61
GEOROC[1]	South Central Anatolia (W)	K102P	38.12	34.28	67.80	1.13	3.01	4.03	107	270	14.40	28.60	1.43	0.40	18.75	20.00
GEOROC[1]	South Central Anatolia (W)	K114	38.06	34.28	51.90	4.74	1.39	4.02	26	760	21.00	30.00	1.86	0.03	36.19	16.13
GEOROC[1]	South Central Anatolia (W)	K-84	38.11	34.29	71.80	0.66	3.17	4.40	93	229	14.10	29.30	1.42	0.41	16.24	20.63
GEOROC[1]	South Central Anatolia (W)	K-81	38.07	34.29	51.80	5.27	1.26	4.07	23	766	20.90	30.70	1.84	0.03	36.65	16.68
GEOROC[1]	South Central Anatolia (W)	K-83	38.09	34.29	50.70	7.25	1.32	3.14	28	1007	20.10	31.30	1.74	0.03	50.10	17.99
GEOROC[1]	South Central Anatolia (W)	K110	38.11	34.30	70.70	0.64	3.26	4.12	107	221	13.90	28.20	1.51	0.48	15.90	18.68
GEOROC[1]	South Central Anatolia (W)	OC92-1	37.60	34.30	45.02	9.09	2.35	4.44	25	888	26.00	42.40	2.10	0.03	34.15	20.19
GEOROC[1]	South Central Anatolia (W)	OC92-4	37.60	34.30	43.63	9.56	1.88	3.74	22	871	26.00	38.80	1.72	0.02	33.50	22.56
GEOROC[1]	South Central Anatolia (W)	OC92-7	37.60	34.30	43.39	9.03	1.83	4.10	24	954	27.00	38.20	1.79	0.03	35.33	21.34
GEOROC[1]	South Central Anatolia (W)	OC92?1	37.60	34.30	44.33	9.01	2.34	4.78	32	899	26.00	41.00	1.86	0.04	34.58	22.04
GEOROC[1]	South Central Anatolia (W)	OC92?	37.60	34.30	44.34	7.19	1.59	3.93	16	1082	28.00	45.60	2.00	0.01	38.64	22.80
GEOROC[1]	South Central Anatolia (W)	OC92-3	37.60	34.30	44.27	7.63	1.48	3.55	19	964	27.00	41.50	2.12	0.02	35.70	19.58
GEOROC[1]	South Central Anatolia (W)	OC92-8	37.60	34.30	43.47	9.18	1.32	3.24	13	928	27.00	39.70	1.92	0.01	34.37	20.68
GEOROC[1]	South Central Anatolia (W)	OC92-10	37.60	34.30	44.34	8.85	1.28	3.62	13	1037	25.00	36.30	1.67	0.01	41.48	21.74
GEOROC[1]	South Central Anatolia (W)	OC92-13	37.60	34.30	43.13	9.95	1.41	3.68	14	936	26.00	41.50	1.87	0.01	36.00	22.19
GEOROC[1]	South Central Anatolia (W)	OC92-14	37.60	34.30	44.03	9.62	1.30	3.50	13	1059	25.00	37.90	1.91	0.01	42.36	19.84
GEOROC[1]	South Central Anatolia (W)	C94-13	37.86	34.30	46.55	9.79	0.74	3.01	12	575	16.00	30.52	1.76	0.02	35.91	17.34
GEOROC[1]	South Central Anatolia (W)	C94-14	37.86	34.31	48.75	8.58	1.03	3.46	14	570	19.30	24.58	2.12	0.02	29.52	11.59
GEOROC[1]	South Central Anatolia (W)	K-75	38.00	34.34	50.00	5.55	1.59	3.61	44	947	22.20	44.40	1.99	0.05	42.66	22.31
GEOROC[1]	South Central Anatolia (W)	K-61	38.20	34.36	49.00	6.75	0.57	3.88	8	508	26.00	14.10	2.53	0.02	19.54	5.57
GEOROC[1]	South Central Anatolia (W)	K-63	38.18	34.36	50.20	7.00	1.38	4.17	22	656	25.10	25.30	2.40	0.03	26.14	10.54
GEOROC[1]	South Central Anatolia (W)	KEC-1	38.02	34.39	72.80	0.13	4.85	3.14	117	65	12.20	15.70	1.22	1.80	5.33	12.87
GEOROC[1]	South Central Anatolia (W)	K-51	38.19	34.39	50.90	7.22	1.11	3.64	17	648	26.90	28.30	2.71	0.03	24.09	10.44
GEOROC[1]	South Central Anatolia (W)	KEC-2	38.18	34.42	69.50	0.77	3.45	3.77	112	217	13.70	24.20	1.42	0.52	15.84	17.04
GEOROC[1]	South Central Anatolia (W)	G-K2	38.37	34.52	74.40	0.05	4.85	3.80	179	16	21.10	24.40	2.13	11.19	0.76	11.46
GEOROC[1]	South Central Anatolia (W)	G-C2	38.34	34.53	49.30	6.12	0.55	3.76	7	502	27.70	13.90	2.45	0.01	18.12	5.67
GEOROC[1]	South Central Anatolia (W)	GOELL-P	38.23	34.54	71.10	0.16	4.47	3.84	163	72	17.00	24.00	2.70	2.26	4.24	8.89
GEOROC[1]	South Central Anatolia (W)	GOELL	38.28	34.55	74.20	0.07	4.43	3.84	183	8	16.00	19.00	2.90	22.88	0.50	6.55
GEOROC[1]	South Central Anatolia (W)	C94-16 (2)	38.38	34.57	54.57	6.24	1.25	3.21	33	356	12.98	24.02	1.45	0.09	27.39	16.57
GEOROC[1]	South Central Anatolia (W)	C94-31	38.42	34.63	57.64	3.51	1.72	3.25	33	325	16.83	24.12	1.93	0.10	19.28	12.50
GEOROC[1]	South Central Anatolia (E)	E-188	38.46	35.23	60.40	3.08	1.83	3.95	63	378	18.40	19.46	1.80	0.17	20.53	10.81
GEOROC[1]	South Central Anatolia (E)	E-185	38.49	35.26	60.00	3.15	1.69	3.61	46	352	18.20	20.64	1.88	0.13	19.36	10.98
GEOROC[1]	South Central Anatolia (E)	E-194	38.60	35.27	53.83	4.29	1.62	3.96	43	501	22.90	24.85	2.31	0.09	21.87	10.76
GEOROC[1]	South Central Anatolia (E)	C94-24	38.59	35.32	52.12	5.74	1.44	3.71	21	420	17.85	23.68	1.93	0.05	23.51	12.27
GEOROC[1]	South Central Anatolia (E)	C94-22	38.59	35.32	51.57	5.67	1.59	3.75	25	436	19.63	30.55	2.12	0.06	22.21	14.41

Source	Location	Sample	Latitude	Longitude	SiO2(wt.%)	MgO(wt.%)	K2O(wt.%)	Na2O(wt.%)	Rb(ppm)	Sr(ppm)	Y(ppm)	La(ppm)	Yb(ppm)	Rb/Sr	Sr/Y	La/Yb
GEOROC[1]	South Central Anatolia (E)	C94-26	38.54	35.33	54.97	4.79	1.19	3.25	23	382	14.14	23.99	1.45	0.06	27.03	16.54
GEOROC[1]	South Central Anatolia (E)	E-196	38.45	35.36	51.24	5.59	1.34	3.92	25	558	22.50	22.57	2.32	0.04	24.80	9.73
GEOROC[1]	South Central Anatolia (E)	C94-30	38.60	35.43	51.16	6.38	1.01	3.37	14	419	21.87	23.89	2.39	0.03	19.14	10.00
GEOROC[1]	South Central Anatolia (E)	ERC96-195	38.53	35.45	62.10	2.73	2.14	3.88	77	334	29.70			0.23	11.26	
GEOROC[1]	South Central Anatolia (E)	ERC95-64	38.53	35.45	51.98	6.02	0.95	3.75	14	528	30.10	19.70	2.43	0.03	17.55	8.11
GEOROC[1]	South Central Anatolia (E)	ERC91-13	38.53	35.45	53.57	5.74	1.27	3.84	27	516	23.60	31.80	2.19	0.05	21.87	14.52
GEOROC[1]	South Central Anatolia (E)	ERC91-3	38.53	35.45	54.66	5.72	1.05	3.54	23	460	22.00	23.90	2.12	0.05	20.93	11.27
GEOROC[1]	South Central Anatolia (E)	ERC95-66	38.53	35.45	53.51	6.58	1.17	3.41	22	482	25.80			0.04	18.69	
GEOROC[1]	South Central Anatolia (E)	ERC95-92	38.53	35.45	63.98	2.52	2.03	3.71	62	307	22.00			0.20	13.97	
GEOROC[1]	South Central Anatolia (E)	ERC96-129	38.53	35.45	62.99	2.61	2.22	3.90	70	308	27.10			0.23	11.35	
GEOROC[1]	South Central Anatolia (E)	ERC91-29	38.53	35.45	57.11	3.66	1.34	4.21	30	422	29.00	27.90	2.58	0.07	14.54	10.81
GEOROC[1]	South Central Anatolia (E)	ERC96-175	38.53	35.45	61.09	3.30	1.36	3.48	38	365	19.90			0.10	18.34	
GEOROC[1]	South Central Anatolia (E)	ERC95-99	38.53	35.45	67.03	1.92	2.69	3.83	96	242	22.10			0.39	10.97	
GEOROC[1]	South Central Anatolia (E)	ERC95-100	38.53	35.45	69.92	1.14	3.04	3.81	116	213	22.70			0.55	9.37	
GEOROC[1]	South Central Anatolia (E)	ERC91-9	38.53	35.45	65.71	2.15	1.99	3.91	78	275	26.10			0.28	10.55	
GEOROC[1]	South Central Anatolia (E)	ERC95-83	38.53	35.45	67.06	1.69	2.27	3.68	83	258	23.80	20.30	1.84	0.32	10.82	11.03
GEOROC[1]	South Central Anatolia (E)	ERC95-90	38.53	35.45	70.55	0.79	2.83	3.92	113	201	22.00			0.56	9.13	
GEOROC[1]	South Central Anatolia (E)	ERC96-109	38.53	35.45	62.07	3.07	1.37	3.37	37	335	21.00			0.11	15.97	
GEOROC[1]	South Central Anatolia (E)	ERC96-112	38.53	35.45	59.44	2.70	1.73	3.78	27	376	30.80			0.07	12.22	
GEOROC[1]	South Central Anatolia (E)	ERC96-113	38.53	35.45	62.04	2.11	2.04	4.39	73	343	33.80			0.21	10.16	
GEOROC[1]	South Central Anatolia (E)	ERC96-115	38.53	35.45	66.49	1.00	3.06	4.55	97	254	42.00	25.30	2.81	0.38	6.05	9.00
GEOROC[1]	South Central Anatolia (E)	ERC96-116	38.53	35.45	49.21	6.61	0.71	3.75	6	458	29.70	16.40	2.82	0.01	15.41	5.82
GEOROC[1]	South Central Anatolia (E)	C88-75	38.53	35.45	53.28	6.55	1.46	4.08	29	497	25.00	24.90	2.42	0.06	19.87	10.29
GEOROC[1]	South Central Anatolia (E)	C88-79	38.53	35.45	48.39	8.39	0.18	3.36	2	399	22.40	5.65	2.28	0.01	17.79	2.48
GEOROC[1]	South Central Anatolia (E)	C88-80	38.53	35.45	62.76	3.23	1.80	3.57	2	402	23.30			0.00	17.25	
GEOROC[1]	South Central Anatolia (E)	C88-89	38.53	35.45	71.24	0.93	2.85	4.10	111	202	19.80			0.55	10.19	
GEOROC[1]	South Central Anatolia (E)	ERC91-21	38.53	35.45	65.84	1.98	2.09	3.95	85	295	27.30	25.90	2.10	0.29	10.81	12.33
GEOROC[1]	South Central Anatolia (E)	ERC91-28	38.53	35.45	70.50	0.96	2.79	4.00	134	210	25.00			0.64	8.40	
GEOROC[1]	South Central Anatolia (E)	ERC96-138	38.53	35.45	54.57	5.37	1.18	3.45	22	457	22.80			0.05	20.06	
GEOROC[1]	South Central Anatolia (E)	ERC96-145	38.53	35.45	52.15	4.62	1.59	4.14	30	657	28.60			0.05	22.97	
GEOROC[1]	South Central Anatolia (E)	ERC96-151	38.53	35.45	60.45	3.26	1.23	3.44	36	330	18.70			0.11	17.63	
GEOROC[1]	South Central Anatolia (E)	ERC96-186	38.53	35.45	65.26	2.01	2.27	3.46	87	264	24.40			0.33	10.83	
GEOROC[1]	South Central Anatolia (E)	C2	38.53	35.45	55.83	5.23	1.21	3.52	29	494	22.10	24.20	2.22	0.06	22.36	10.90
GEOROC[1]	South Central Anatolia (E)	C3C	38.53	35.45	55.54	5.29	1.17	3.46	29	512	23.20	24.40	2.26	0.06	22.07	10.80
GEOROC[1]	South Central Anatolia (E)	C6	38.53	35.45	55.30	5.07	1.19	3.41	30	502	22.10	23.70	2.07	0.06	22.72	11.45
GEOROC[1]	South Central Anatolia (E)	C21	38.53	35.45	55.27	5.02	1.18	3.43	27	515	23.00	24.80	2.11	0.05	22.41	11.75
GEOROC[1]	South Central Anatolia (E)	C23	38.53	35.45	55.68	4.95	1.18	3.63	28	494	21.20	23.80	1.95	0.06	23.29	12.21
GEOROC[1]	South Central Anatolia (E)	2C13	38.53	35.45	55.64	4.91	1.17	3.48	31	510	22.80	24.60	2.03	0.06	22.35	12.12
GEOROC[1]	South Central Anatolia (E)	C11C	38.53	35.45	55.81	4.96	1.18	3.52	27	498	21.90	23.30	1.90	0.05	22.75	12.26
GEOROC[1]	South Central Anatolia (E)	C22	38.53	35.45	55.39	5.06	1.18	3.48	28	525	21.30	23.60	2.04	0.05	24.63	11.57
GEOROC[1]	South Central Anatolia (E)	C14	38.53	35.45	55.45	5.04	1.15	3.44	27	508	21.60	23.30	2.19	0.05	23.51	10.64
GEOROC[1]	South Central Anatolia (E)	2C1	38.53	35.45	55.45	5.26	1.19	3.51	28	501	21.40	24.00	2.05	0.06	23.42	11.71
GEOROC[1]	South Central Anatolia (E)	2C6	38.53	35.45	55.66	5.06	1.19	3.48	29	508	22.20	24.20	2.04	0.06	22.87	11.86
GEOROC[1]	South Central Anatolia (E)	A2	38.53	35.45	60.48	3.08	1.89	3.87	51	348	25.30	25.10	2.54	0.15	13.77	9.88

Source	Location	Sample	Latitude	Longitude	SiO2(wt.%)	MgO(wt.%)	K2O(wt.%)	Na2O(wt.%)	Rb(ppm)	Sr(ppm)	Y(ppm)	La(ppm)	Yb(ppm)	Rb/Sr	Sr/Y	La/Yb
GEOROC[1]	South Central Anatolia (E)	A4	38.53	35.45	61.42	3.30	1.83	3.71	54	327	22.70	24.20	2.32	0.16	14.42	10.43
GEOROC[1]	South Central Anatolia (E)	CM2G	38.53	35.45	64.35	2.40	2.22	3.86	77	321	23.50	29.30	2.35	0.24	13.68	12.47
GEOROC[1]	South Central Anatolia (E)	CM8	38.53	35.45	64.91	2.29	2.26	3.91	81	308	22.70	28.50	2.21	0.26	13.55	12.90
GEOROC[1]	South Central Anatolia (E)	CM1E	38.53	35.45	64.75	2.41	2.24	3.90	77	298	22.60	26.70	2.07	0.26	13.17	12.90
GEOROC[1]	South Central Anatolia (E)	CM5	38.53	35.45	61.92	2.60	1.99	3.82	67	329	25.10	27.30	2.27	0.20	13.11	12.03
GEOROC[1]	South Central Anatolia (E)	CM5K	38.53	35.45	63.61	2.42	2.13	3.82	76	304	22.70	26.80	2.27	0.25	13.40	11.81
GEOROC[1]	South Central Anatolia (E)	ERC95-128	38.53	35.45	61.02	3.03	1.85	3.72	61	323	26.90			0.19	12.02	
GEOROC[1]	South Central Anatolia (E)	ERC98-3	38.53	35.45	62.52	3.37	1.76	3.83	59	291	16.20	19.50	1.83	0.20	17.96	10.66
GEOROC[1]	South Central Anatolia (E)	ERC95-9	38.53	35.45	63.34	2.19	2.37	4.10	80	286	27.70			0.28	10.32	
GEOROC[1]	South Central Anatolia (E)	ERC96-71	38.53	35.45	64.72	2.02	2.03	4.54	69	265	20.40	26.20	2.21	0.26	12.97	11.86
GEOROC[1]	South Central Anatolia (E)	ERC91-27	38.53	35.45	68.87	1.28	2.82	3.85	114	210	25.60			0.54	8.21	
GEOROC[1]	South Central Anatolia (E)	ERC95-111	38.53	35.45	65.84	1.60	2.26	3.60	91	249	24.30			0.36	10.24	
GEOROC[1]	South Central Anatolia (E)	ERC96-55	38.53	35.45	63.51	2.04	1.79	3.55	65	281	22.10			0.23	12.71	
GEOROC[1]	South Central Anatolia (E)	ERC95-14	38.53	35.45	62.25	1.55	3.01	4.20	88	298	36.40			0.30	8.19	
GEOROC[1]	South Central Anatolia (E)	ERC95-75	38.53	35.45	63.72	1.23	2.52	4.60	87	297	38.70			0.29	7.67	
GEOROC[1]	South Central Anatolia (E)	ERC91-1	38.53	35.45	63.24	2.78	1.60	3.80	55	315	25.20			0.18	12.50	
GEOROC[1]	South Central Anatolia (E)	ERC92-1	38.53	35.45	65.06	2.21	2.15	3.78	92	275	24.30			0.33	11.33	
GEOROC[1]	South Central Anatolia (E)	ERC92-3	38.53	35.45	70.10	0.92	2.96	3.90	147	195	23.80	28.50	2.10	0.75	8.17	13.57
GEOROC[1]	South Central Anatolia (E)	ER11	38.53	35.45	62.02	2.93	1.63	3.65	45	324	22.00	23.80	2.15	0.14	14.73	11.07
GEOROC[1]	South Central Anatolia (E)	ER13	38.53	35.45	66.44	1.40	2.99	4.54	106	229	27.00	30.10	2.70	0.46	8.48	11.15
GEOROC[1]	South Central Anatolia (E)	ER14	38.53	35.45	63.76	2.24	2.45	4.17	84	286	27.00	30.20	2.67	0.29	10.59	11.31
GEOROC[1]	South Central Anatolia (E)	ER15	38.53	35.45	68.97	1.15	2.79	3.96	106	217	21.00	27.40	2.14	0.49	10.33	12.80
GEOROC[1]	South Central Anatolia (E)	ER16	38.53	35.45	69.14	0.78	2.85	3.89	117	207	21.00	30.00	2.18	0.57	9.86	13.76
GEOROC[1]	South Central Anatolia (E)	ER17	38.53	35.45	64.24	2.08	2.36	3.77	81	290	27.00	36.10	2.51	0.28	10.74	14.38
GEOROC[1]	South Central Anatolia (E)	ER20	38.53	35.45	65.69	2.19	2.32	3.75	79	281	25.00	33.20	2.37	0.28	11.24	14.01
GEOROC[1]	South Central Anatolia (E)	ER21	38.53	35.45	63.18	2.62	1.81	3.84	64	318	23.00	24.90	2.23	0.20	13.83	11.17
GEOROC[1]	South Central Anatolia (E)	ER22	38.53	35.45	66.20	2.20	2.55	4.41	95	259	22.00			0.37	11.77	
GEOROC[1]	South Central Anatolia (E)	ER23	38.53	35.45	63.48	2.91	2.23	3.49	70	315	22.00	23.50	1.76	0.22	14.32	13.35
GEOROC[1]	South Central Anatolia (E)	ER24	38.53	35.45	47.56	5.80	0.80	3.91	6	464	41.00	20.70	3.75	0.01	11.32	5.52
GEOROC[1]	South Central Anatolia (E)	ER25	38.53	35.45	64.33	2.43	1.78	3.87	48	303	21.00	23.20	1.95	0.16	14.43	11.90
GEOROC[1]	South Central Anatolia (E)	ER26	38.53	35.45	57.90	3.90	1.50	3.96	26	394	27.00	27.80	2.71	0.07	14.59	10.26
GEOROC[1]	South Central Anatolia (E)	IEY-001	38.53	35.45	68.92	1.58	1.93	3.83	89	273	19.90	23.40	1.86	0.33	13.72	12.58
GEOROC[1]	South Central Anatolia (E)	IEY-003	38.53	35.45	67.93	1.75	2.22	3.79	98	269	23.40	26.90	2.26	0.36	11.50	11.90
GEOROC[1]	South Central Anatolia (E)	IEY-005	38.53	35.45	69.10	1.77	2.16	3.80	93	262	22.10	27.60	2.00	0.35	11.86	13.80
GEOROC[1]	South Central Anatolia (E)	IEY-0017	38.53	35.45	67.54	1.91	2.08	3.82	90	256	22.10	26.20	2.21	0.35	11.58	11.86
GEOROC[1]	South Central Anatolia (E)	IY-033	38.53	35.45	67.50	1.77	2.34	3.96	84	258	22.20	25.00	2.01	0.33	11.62	12.44
GEOROC[1]	South Central Anatolia (E)	IY-0317	38.53	35.45	67.50	1.82	2.42	3.82	83	256	22.80	25.00	1.99	0.32	11.23	12.56
GEOROC[1]	South Central Anatolia (E)	EY-005	38.53	35.45	66.96	2.50	2.53	3.64	74	313	16.80	23.80	2.15	0.24	18.63	11.07
GEOROC[1]	South Central Anatolia (E)	EY-0012	38.53	35.45	65.48	2.15	2.31	4.21	72	323	21.20	29.70	2.18	0.22	15.24	13.62
GEOROC[1]	South Central Anatolia (E)	EY-0016	38.53	35.45	65.96	2.43	2.56	3.81	87	248	22.00	28.40	2.31	0.35	11.27	12.29
GEOROC[1]	South Central Anatolia (E)	IEY-00X6	38.53	35.45	59.57	3.92	1.53	3.29	49	438	21.40	27.50	1.84	0.11	20.47	14.95
GEOROC[1]	South Central Anatolia (E)	IEY-00X16	38.53	35.45	62.74	2.80	1.76	3.65	60	342	20.00	25.30	2.04	0.18	17.10	12.40
GEOROC[1]	South Central Anatolia (E)	IEY-007	38.53	35.45	66.85	2.67	2.91	3.42	99	245	19.30	25.00	2.03	0.40	12.69	12.32
GEOROC[1]	South Central Anatolia (E)	IEY-009	38.53	35.45	69.60	1.65	2.94	3.74	89	264	22.40	28.40	2.23	0.34	11.79	12.74

Source	Location	Sample	Latitude	Longitude	SiO2(wt.%)	MgO(wt.%)	K2O(wt.%)	Na2O(wt.%)	Rb(ppm)	Sr(ppm)	Y(ppm)	La(ppm)	Yb(ppm)	Rb/Sr	Sr/Y	La/Yb
GEOROC[1]	South Central Anatolia (E)	IEY-0013	38.53	35.45	66.53	2.05	2.22	3.74	86	269	23.50	26.70	2.32	0.32	11.45	11.51
GEOROC[1]	South Central Anatolia (E)	IEY-00X2	38.53	35.45	65.77	2.69	2.39	3.34	81	300	21.00	28.90	2.02	0.27	14.29	14.31
GEOROC[1]	South Central Anatolia (E)	IEY-00X4	38.53	35.45	65.23	2.71	1.99	3.31	71	316	21.00	30.00	2.01	0.22	15.05	14.93
GEOROC[1]	South Central Anatolia (E)	IEY-00X8	38.53	35.45	65.83	3.10	2.05	3.25	70	313	18.00	24.10	1.92	0.22	17.39	12.55
GEOROC[1]	South Central Anatolia (E)	IEY-00X10	38.53	35.45	67.04	2.57	1.63	3.22	54	304	16.80	24.40	1.58	0.18	18.10	15.44
GEOROC[1]	South Central Anatolia (E)	IEY-00X12	38.53	35.45	66.66	2.04	2.14	3.73	89	280	24.50	27.40	2.40	0.32	11.43	11.42
GEOROC[1]	South Central Anatolia (E)	IEY-00X14	38.53	35.45	66.70	2.23	1.97	3.68	81	280	23.20	26.30	2.21	0.29	12.07	11.90
GEOROC[1]	South Central Anatolia (E)	IEY-00X18	38.53	35.45	68.65	1.27	2.99	3.71	126	232	25.20	30.80	2.56	0.54	9.21	12.03
GEOROC[1]	South Central Anatolia (E)	IEY-00X20	38.53	35.45	68.85	1.47	2.74	3.68	85	257	20.40	27.90	2.03	0.33	12.60	13.74
GEOROC[1]	South Central Anatolia (E)	IEY-00X22	38.53	35.45	69.90	1.26	3.05	3.73	94	230	20.60	30.20	2.09	0.41	11.17	14.45
GEOROC[1]	South Central Anatolia (E)	IEY-00X24	38.53	35.45	70.00	1.50	2.48	3.48	86	229	20.00	28.50	2.06	0.38	11.45	13.83
GEOROC[1]	South Central Anatolia (E)	IEY-00X26	38.53	35.45	68.43	1.75	2.40	3.58	86	250	21.70	27.50	2.22	0.34	11.52	12.39
GEOROC[1]	South Central Anatolia (E)	IEY-00X30	38.53	35.45	69.12	1.50	2.92	3.67	99	254	22.20	29.50	2.22	0.39	11.44	13.29
GEOROC[1]	South Central Anatolia (E)	IEY-0011	38.53	35.45	71.11	1.28	3.11	3.92	97	233	20.90	30.10	1.95	0.42	11.15	15.44
GEOROC[1]	South Central Anatolia (E)	IEY-0015A	38.53	35.45	70.51	1.03	3.10	3.84	114	213	21.40	29.50	2.28	0.54	9.95	12.94
GEOROC[1]	South Central Anatolia (E)	IEY-0015B	38.53	35.45	70.62	1.02	3.11	3.85	116	225	21.70	30.80	2.11	0.52	10.37	14.60
GEOROC[1]	South Central Anatolia (E)	IEY-00X28	38.53	35.45	70.94	0.75	3.03	3.83	57	113	10.10	15.50	1.05	0.50	11.19	14.76
GEOROC[1]	South Central Anatolia (E)	OU-034	38.53	35.45	55.19	5.76	1.18	3.41	23	467	22.00	23.20	2.08	0.05	21.23	11.15
GEOROC[1]	South Central Anatolia (E)	OU-0305A	38.53	35.45	54.62	5.95	1.16	3.40	23	513	21.90	26.20	2.05	0.04	23.42	12.78
GEOROC[1]	South Central Anatolia (E)	OU-0312A	38.53	35.45	54.40	6.10	1.42	3.34	25	493	22.80	23.80	1.91	0.05	21.62	12.46
GEOROC[1]	South Central Anatolia (E)	OU-0313A	38.53	35.45	55.81	5.38	1.04	3.68	21	452	20.40	23.30	1.78	0.05	22.16	13.09
GEOROC[1]	South Central Anatolia (E)	OU-0321B	38.53	35.45	55.52	4.63	1.48	4.02	33	517	27.00	34.80	2.54	0.06	19.15	13.70
GEOROC[1]	South Central Anatolia (E)	OU-0322-7	38.53	35.45	55.01	5.14	1.38	3.86	26	503	27.30	30.20	2.43	0.05	18.42	12.43
GEOROC[1]	South Central Anatolia (E)	OU-0322-8	38.53	35.45	55.43	4.58	1.36	3.95	25	476	25.80	28.80	2.30	0.05	18.45	12.52
GEOROC[1]	South Central Anatolia (E)	OU-0331-2	38.53	35.45	54.62	4.87	1.34	3.82	26	513	26.50	30.30	2.49	0.05	19.36	12.17
GEOROC[1]	South Central Anatolia (E)	OU-0319	38.53	35.45	67.82	2.10	1.82	3.89	56	265	20.20	23.70	1.93	0.21	13.12	12.28
GEOROC[1]	South Central Anatolia (E)	OU-0322-5B	38.53	35.45	65.28	2.46	1.82	3.90	57	300	22.40	25.60	2.03	0.19	13.39	12.61
GEOROC[1]	South Central Anatolia (E)	OU-0322-6	38.53	35.45	65.88	2.42	1.85	3.83	58	284	20.90	23.00	1.93	0.20	13.59	11.92
GEOROC[1]	South Central Anatolia (E)	OU-0322-9	38.53	35.45	65.46	2.17	2.54	3.82	79	295	20.80	29.60	2.18	0.27	14.18	13.58
GEOROC[1]	South Central Anatolia (E)	OU-0326-1	38.53	35.45	67.03	1.74	2.27	3.89	81	265	21.10	24.80	1.93	0.31	12.56	12.85
GEOROC[1]	South Central Anatolia (E)	OU-0328B	38.53	35.45	66.95	1.88	2.30	3.90	81	265	20.10	21.30	1.97	0.31	13.18	10.81
GEOROC[1]	South Central Anatolia (E)	C94-18	38.41	35.46	53.84	5.98	1.27	3.22	23	425	14.11	28.72	1.49	0.05	30.15	19.28
GEOROC[1]	South Central Anatolia (E)	E-175	38.41	35.46	53.03	6.14	1.14	3.52	26	531	18.20	25.54	1.87	0.05	29.15	13.66
GEOROC[1]	South Central Anatolia (E)	E-103	38.55	35.54	62.19	2.04	2.94	3.77	98	300	32.20	29.41	2.70	0.33	9.33	10.89
GEOROC[1]	South Central Anatolia (E)	E-86	38.67	35.55	64.08	1.91	2.03	3.85	65	316	19.60	23.36	1.98	0.21	16.10	11.80
GEOROC[1]	South Central Anatolia (E)	E-108	38.56	35.55	58.71	3.46	1.96	3.43	61	376	20.70	22.93	1.91	0.16	18.15	12.01
GEOROC[1]	South Central Anatolia (E)	E-88	38.66	35.56	62.19	2.01	1.98	3.74	69	295	20.20	22.63	1.99	0.24	14.62	11.37
GEOROC[1]	South Central Anatolia (E)	E-133	38.43	35.58	57.31	4.34	1.41	3.36	44	396	18.60	19.86	1.79	0.11	21.30	11.09
GEOROC[1]	South Central Anatolia (E)	E-152	38.33	35.62	47.26	6.59	0.73	3.86	27	451	28.50	18.63	2.68	0.06	15.83	6.95
GEOROC[1]	South Central Anatolia (E)	E-149	38.30	35.63	47.96	6.78	0.73	3.79	17	557	31.20	20.92	2.98	0.03	17.85	7.02
GEOROC[1]	South Central Anatolia (E)	E-148	38.33	35.63	47.76	6.79	0.67	3.80	26	515	27.60	17.63	2.76	0.05	18.67	6.39
GEOROC[1]	South Central Anatolia (E)	E-150	38.29	35.64	47.76	6.52	0.77	3.92	15	488	29.00	19.64	2.87	0.03	16.82	6.84
GEOROC[1]	South Central Anatolia (E)	E-233	38.29	35.64	47.56	6.75	0.73	3.81	27	471	28.00	19.12	2.65	0.06	16.84	7.22
GEOROC[1]	South Central Anatolia (E)	E-146	38.32	35.64	47.96	6.28	0.77	3.93	18	492	29.50	19.73	3.18	0.04	16.67	6.20

Source	Location	Sample	Latitude	Longitude	SiO2(wt.%)	MgO(wt.%)	K2O(wt.%)	Na2O(wt.%)	Rb(ppm)	Sr(ppm)	Y(ppm)	La(ppm)	Yb(ppm)	Rb/Sr	Sr/Y	La/Yb
GEOROC[1]	South Central Anatolia (E)	E-169	38.32	35.64	64.08	2.17	2.47	3.76	96	279	21.30	26.72	2.01	0.34	13.10	13.29
GEOROC[1]	South Central Anatolia (E)	E-141	38.34	35.65	61.39	2.71	2.07	4.06	71	318	19.20	20.97	1.93	0.22	16.55	10.87
GEOROC[1]	South Central Anatolia (E)	E-102-B	38.57	35.65	58.61	3.12	2.16	4.01	87	330	28.30	33.99	2.51	0.26	11.66	13.54
GEOROC[1]	South Central Anatolia (E)	ZD03-04	38.60	35.90	66.34	1.78	2.24	3.96	85	261	22.50	23.80	2.14	0.33	11.60	11.12
GEOROC[1]	South Central Anatolia (E)	ZD03-08A	38.60	35.90	66.44	1.76	2.34	3.93	86	254	22.00	24.10	2.11	0.34	11.55	11.42
GEOROC[1]	South Central Anatolia (E)	ZD03-08B	38.60	35.90	66.49	1.77	2.34	3.93	86	256	21.80	24.00	2.05	0.34	11.74	11.71
GEOROC[1]	South Central Anatolia (E)	ZD03-40	38.60	35.90	66.91	1.82	2.27	3.87	83	253	23.00	25.50	2.14	0.33	11.00	11.92
GEOROC[1]	South Central Anatolia (E)	ZD03-12	38.60	35.90	66.00	1.73	2.31	3.94	87	256	22.30	24.90	1.96	0.34	11.48	12.70
GEOROC[1]	South Central Anatolia (E)	ZD03-16	38.60	35.90	66.35	1.74	2.24	3.92	85	248	21.20	24.30	2.10	0.34	11.70	11.57
GEOROC[1]	South Central Anatolia (E)	ZD03-26	38.60	35.90	66.73	1.87	2.27	3.91	86	258	21.80	24.60	1.92	0.33	11.83	12.81
GEOROC[1]	South Central Anatolia (E)	ZD03-34	38.60	35.90	66.60	1.72	2.29	3.93	88	260	22.20	25.60	2.04	0.34	11.71	12.55
GEOROC[1]	South Central Anatolia (E)	ZD03-11	38.60	35.90	65.20	1.99	2.21	3.76	79	258	21.90	24.50	2.11	0.31	11.78	11.61
GEOROC[1]	South Central Anatolia (E)	ZD03-15	38.60	35.90	66.03	2.05	2.20	3.86	77	254	21.50	22.90	2.16	0.30	11.81	10.60
GEOROC[1]	South Central Anatolia (E)	ZD03-19	38.60	35.90	65.92	2.03	2.18	3.82	84	263	22.60	26.60	2.28	0.32	11.64	11.67
GEOROC[1]	South Central Anatolia (E)	ZD-03-23	38.60	35.90	66.48	1.84	2.30	3.87	82	261	22.40	24.70	2.09	0.31	11.65	11.82
GEOROC[1]	South Central Anatolia (E)	ZD03-01	38.60	35.90	66.76	1.56	2.30	3.92	87	251	21.90	25.30	2.13	0.35	11.46	11.88
GEOROC[1]	South Central Anatolia (E)	ZD03-03	38.60	35.90	66.14	1.69	2.22	3.87	85	264	21.70	23.20	1.99	0.32	12.17	11.66
GEOROC[1]	South Central Anatolia (E)	ZD03-05	38.60	35.90	66.18	1.68	2.30	3.82	88	250	21.50	23.60	2.11	0.35	11.63	11.18
GEOROC[1]	Southwestern Central Anatolia	KO-92-10	38.17	32.50	67.66	1.36	3.83	3.11	157	460	20.40			0.34	22.54	
GEOROC[1]	Southwestern Central Anatolia	KO-92-13	38.17	32.50	66.47	1.44	3.32	3.87	97	828	18.10			0.12	45.74	
GEOROC[1]	Southwestern Central Anatolia	KO-94-6	38.17	32.50	66.70	1.45	3.65	3.20	146	434	20.10			0.34	21.61	
GEOROC[1]	Southwestern Central Anatolia	KO-94-7	38.17	32.50	63.55	1.66	3.27	3.23	122	557	25.60			0.22	21.75	
GEOROC[1]	Southwestern Central Anatolia	KO-94-9	38.17	32.50	65.95	0.44	3.34	4.01	114	920	24.20			0.12	38.00	
GEOROC[1]	Southwestern Central Anatolia	KO-94-10	38.17	32.50	65.16	1.03	3.21	4.08	101	916	17.30			0.11	52.97	
GEOROC[1]	Southwestern Central Anatolia	KO94-11	38.17	32.50	58.04	2.85	2.59	3.40	91	629	24.90			0.14	25.28	
GEOROC[1]	Southwestern Central Anatolia	KO94-21	38.17	32.50	62.44	2.29	3.11	3.28	119	472	29.10			0.25	16.20	
GEOROC[1]	Southwestern Central Anatolia	KO-94-24	38.17	32.50	62.48	2.24	2.85	3.99	81	1041	20.80			0.08	50.04	
GEOROC[1]	Southwestern Central Anatolia	KO-92-4	38.17	32.50	66.48	1.64	3.70	3.66	108	788	18.60			0.14	42.38	
GEOROC[1]	Southwestern Central Anatolia	KO-92-5	38.17	32.50	56.97	5.66	3.36	3.29	86	593	24.30			0.15	24.39	
GEOROC[1]	Southwestern Central Anatolia	KO-92-6	38.17	32.50	59.96	2.32	2.59	3.79	82	635	26.20			0.13	24.25	
GEOROC[1]	Southwestern Central Anatolia	KO-92-7	38.17	32.50	57.80	3.22	2.51	3.51	73	679	26.10			0.11	26.01	
GEOROC[1]	Southwestern Central Anatolia	KO-92-18	38.17	32.50	58.36	3.66	1.64	3.60	45	580	23.50			0.08	24.67	
GEOROC[1]	Southwestern Central Anatolia	KO-92-19	38.17	32.50	60.41	3.37	2.52	3.55	77	887	19.00			0.09	46.66	
GEOROC[1]	Southwestern Central Anatolia	KO-92-128	38.17	32.50	59.17	2.96	2.48	3.44	77	906	20.00			0.08	45.31	
GEOROC[1]	Southwestern Central Anatolia	KO-93-129	38.17	32.50	59.02	2.59	2.07	3.15	79	629	25.70			0.12	24.47	
GEOROC[1]	Southwestern Central Anatolia	KO-93-136	38.17	32.50	63.47	1.84	3.17	3.68	114	630	21.10			0.18	29.85	
GEOROC[1]	Southwestern Central Anatolia	KO-94-14	38.17	32.50	57.68	3.68	2.74	3.17	88	1047	23.00			0.08	45.53	
GEOROC[1]	Southwestern Central Anatolia	KO-94-15	38.17	32.50	59.39	2.49	3.07	3.61	92	1334	22.30			0.07	59.83	
GEOROC[1]	Southwestern Central Anatolia	KO-94-16	38.17	32.50	60.49	2.47	3.06	3.64	98	1149	21.20			0.09	54.17	
GEOROC[1]	Southwestern Central Anatolia	KO-94-17	38.17	32.50	56.73	3.76	2.08	3.42	49	957	19.20			0.05	49.86	
GEOROC[1]	Southwestern Central Anatolia	KO-94-18	38.17	32.50	62.24	1.98	3.41	3.34	121	829	22.30			0.15	37.17	
GEOROC[1]	Southwestern Central Anatolia	KO-94-53	38.17	32.50	58.00	3.40	2.48	3.31	76	934	20.70			0.08	45.11	
GEOROC[1]	Southwestern Central Anatolia	KO-92-1	38.17	32.50	62.12	2.32	2.70	3.90	2	1294	22.30			0.00	58.02	
GEOROC[1]	Southwestern Central Anatolia	KO-92-15	38.17	32.50	68.41	1.45	3.68	3.53	90	634	12.70			0.14	49.95	

Source	Location	Sample	Latitude	Longitude	SiO2(wt.%)	MgO(wt.%)	K2O(wt.%)	Na2O(wt.%)	Rb(ppm)	Sr(ppm)	Y(ppm)	La(ppm)	Yb(ppm)	Rb/Sr	Sr/Y	La/Yb
GEOROC[1]	Southwestern Central Anatolia	KO-92-16	38.17	32.50	62.76	3.01	3.05	4.14	78	1057	19.40			0.07	54.48	
GEOROC[1]	Southwestern Central Anatolia	KO-92-17	38.17	32.50	61.68	2.80	2.55	4.05	72	701	21.50			0.10	32.59	
GEOROC[1]	Southwestern Central Anatolia	KO-94-29	38.17	32.50	66.96	1.21	3.27	3.55	115	840	17.40			0.14	48.28	
GEOROC[1]	Southwestern Central Anatolia	KO-94-36	38.17	32.50	63.46	1.89	2.79	3.74	98	602	21.60			0.16	27.85	
GEOROC[1]	Southwestern Central Anatolia	KO-94-37	38.17	32.50	66.06	1.22	3.01	3.12	108	521	19.80			0.21	26.29	
GEOROC[1]	Northwestern Iran	YK6	38.71	45.37	49.10	7.40	5.40	1.94	108	1155	26.30	57.20	1.95	0.09	43.92	29.33
GEOROC[1]	Northwestern Iran	YK1	38.69	45.39	54.40	2.78	3.07	5.13	45	1540	26.00	102.50	2.27	0.03	59.23	45.15
GEOROC[1]	Northwestern Iran	YS6	38.71	45.39	48.00	3.78	2.04	4.41	25	1130	34.60	61.80	2.89	0.02	32.66	21.38
GEOROC[1]	Northwestern Iran	YS1	38.70	45.39	50.00	2.42	3.20	5.26	42	800	28.80	55.60	2.53	0.05	27.78	21.98
GEOROC[1]	Northwestern Iran	GL5	38.53	45.50	50.00	6.58	5.55	2.35	170	1110	25.80	61.70	1.93	0.15	43.02	31.97
GEOROC[1]	Northwestern Iran	GL6	38.53	45.50	49.60	6.55	5.07	2.21	188	993	24.90	56.90	1.91	0.19	39.88	29.79
GEOROC[1]	Northwestern Iran	GL4	38.54	45.51	50.90	5.92	5.17	2.83	215	1085	26.10	61.00	2.04	0.20	41.57	29.90
GEOROC[1]	Northwestern Iran	L-1	38.62	45.51	65.10	0.98	3.77	4.30	143	972	18.00	42.60	1.88	0.15	54.00	22.66
GEOROC[1]	Northwestern Iran	GL3	38.54	45.51	50.30	6.51	5.13	2.42	173	1005	25.40	57.30	1.91	0.17	39.57	30.00
GEOROC[1]	Northwestern Iran	GL12	38.53	45.51	49.20	5.77	6.39	2.81	256	1200	27.00	65.40	2.05	0.21	44.44	31.90
GEOROC[1]	Northwestern Iran	GL13	38.53	45.51	50.00	6.59	5.14	2.67	249	1005	25.70	57.60	2.00	0.25	39.11	28.80
GEOROC[1]	Northwestern Iran	D3	38.61	45.51	49.60	6.29	6.05	1.73	193	1135	24.30	64.70	2.02	0.17	46.71	32.03
GEOROC[1]	Northwestern Iran	GL11	38.53	45.51	48.90	6.55	5.88	2.39	374	1085	26.20	59.90	1.98	0.34	41.41	30.25
GEOROC[1]	Northwestern Iran	GL14	38.53	45.51	49.40	6.58	5.17	2.52	231	1000	25.50	57.50	1.89	0.23	39.22	30.42
GEOROC[1]	Northwestern Iran	GAB1	38.54	45.51	51.50	5.88	4.40	2.61	84	1045	24.20	65.50	2.04	0.08	43.18	32.11
GEOROC[1]	Northwestern Iran	D2	38.63	45.51	49.50	5.88	5.70	2.83	159	1625	24.30	66.00	1.97	0.10	66.87	33.50
GEOROC[1]	Northwestern Iran	D2-4	38.61	45.51	48.10	6.01	3.10	3.79	72	1290	23.90	75.70	2.18	0.06	53.97	34.72
GEOROC[1]	Northwestern Iran	GAB2	38.52	45.51	51.40	5.82	4.49	2.59	88	1110	23.80	64.80	1.98	0.08	46.64	32.73
GEOROC[1]	Northwestern Iran	GL2	38.54	45.51	50.60	6.50	5.26	2.34	252	985	25.00	56.10	1.88	0.26	39.40	29.84
GEOROC[1]	Northwestern Iran	GL7	38.53	45.52	50.10	6.67	5.18	2.49	200	1000	25.20	57.10	2.00	0.20	39.68	28.55
GEOROC[1]	Northwestern Iran	GL1	38.54	45.52	51.10	6.63	4.92	2.64	228	1000	24.30	58.00	1.88	0.23	41.15	30.85
GEOROC[1]	Northwestern Iran	GB1	38.54	45.52	50.50	5.78	6.38	3.40	239	1135	26.00	68.70	2.04	0.21	43.65	33.68
GEOROC[1]	Northwestern Iran	GB3	38.53	45.52	50.20	5.73	6.46	3.35	240	1125	25.80	67.90	2.08	0.21	43.60	32.64
GEOROC[1]	Northwestern Iran	DL13	38.55	45.52	48.90	5.92	5.82	1.34	187	2680	24.60	66.00	2.01	0.07	108.94	32.84
GEOROC[1]	Northwestern Iran	DB2	38.55	45.52	52.40	5.29	5.91	2.24	160	941	20.80	56.10	1.75	0.17	45.24	32.06
GEOROC[1]	Northwestern Iran	DB3	38.55	45.52	52.30	5.13	5.90	2.26	161	947	20.80	56.50	1.76	0.17	45.53	32.10
GEOROC[1]	Northwestern Iran	BA2	38.55	45.53	57.50	3.50	2.76	5.27	56	1030	16.80	64.90	1.51	0.05	61.31	42.98
GEOROC[1]	Northwestern Iran	DBA2	38.55	45.53	49.20	5.33	2.97	3.67	47	1265	23.50	76.20	2.04	0.04	53.83	37.35
GEOROC[1]	Northwestern Iran	GDB5	38.56	45.54	52.40	5.58	2.94	3.90	67	1125	20.90	68.50	1.76	0.06	53.83	38.92
GEOROC[1]	Northwestern Iran	DA14	38.56	45.54	62.40	2.21	2.87	5.31	69	1045	12.70	61.40	1.10	0.07	82.28	55.82
GEOROC[1]	Northern Iran	TBQ1.1	36.12	47.31	49.57	9.47	3.33	2.77	103	547	26.90	49.20	2.19	0.19	20.33	22.47
GEOROC[1]	Northern Iran	TBQ1.2	36.12	47.31	50.58	9.46	3.06	3.33	97	532	25.80	45.40	2.15	0.18	20.62	21.12
GEOROC[1]	Northern Iran	TBQ3.4	35.68	47.62	48.19	7.49	2.93	4.92	56	1100	25.70	88.50	1.63	0.05	42.80	54.29
GEOROC[1]	Northern Iran	TBQ4.7	35.68	47.62	46.43	10.07	2.38	4.26	44	1340	24.00	91.20	1.68	0.03	55.83	54.29
GEOROC[1]	Northern Iran	TBQ5.4	35.68	47.62	49.46	8.94	1.39	3.17	21	697	21.90	36.80	1.92	0.03	31.83	19.17
GEOROC[1]	Northern Iran	TBQ5.11	35.68	47.62	48.47	8.79	3.24	4.64	58	1534	24.60	163.00	1.65	0.04	62.36	98.79
GEOROC[1]	Northern Iran	TBQ4.1A	35.69	47.63	48.88	8.10	2.06	3.79	37	1445	25.70	94.60	1.82	0.03	56.23	51.98

Source	Location	Sample	Latitude	Longitude	SiO2(wt.%)	MgO(wt.%)	K2O(wt.%)	Na2O(wt.%)	Rb(ppm)	Sr(ppm)	Y(ppm)	La(ppm)	Yb(ppm)	Rb/Sr	Sr/Y	La/Yb
GEOROC[1]	Northern Iran	TBQ4.5A	35.69	47.63	47.35	9.31	2.68	4.01	44	1378	24.20	97.20	1.73	0.03	56.94	56.18
GEOROC[1]	Northern Iran	TBQ4.6A	35.69	47.63	46.49	9.04	2.67	4.14	45	1334	23.80	93.40	1.66	0.03	56.05	56.27
GEOROC[1]	Northern Iran	TBQ20.1	35.53	47.84	48.16	8.48	3.32	4.45	62	1676	26.60	165.00	1.70	0.04	63.01	97.06
GEOROC[1]	Northern Iran	TBQ6.1	35.39	47.87	46.07	9.48	1.82	4.52	38	1393	23.90	110.00	1.65	0.03	58.28	66.67
GEOROC[1]	Northern Iran	TBQ16.1	35.40	47.89	44.46	9.48	1.19	4.45	12	1408	25.50	79.00	1.64	0.01	55.22	48.17
GEOROC[1]	Northern Iran	TBQ11.1	35.47	47.90	46.56	10.67	3.18	3.81	47	2002	23.80	136.00	1.52	0.02	84.12	89.47
GEOROC[1]	Northern Iran	TBQ10.1	35.48	47.91	46.25	10.68	3.06	4.15	43	1981	23.00	127.00	1.51	0.02	86.13	84.11
GEOROC[1]	Northern Iran	TBQ10.2	35.48	47.91	46.14	10.60	3.04	4.17	44	1999	22.90	128.00	1.52	0.02	87.29	84.21
GEOROC[1]	Northern Iran	TBQ10.3	35.48	47.91	46.13	10.51	2.05	4.57	26	2081	23.10	128.00	1.50	0.01	90.09	85.33
GEOROC[1]	Northern Iran	TBQ9.1	35.48	47.91	45.96	10.76	1.84	3.56	38	2382	22.50	129.00	1.52	0.02	105.87	84.87
GEOROC[1]	Northern Iran	TBQ7.1	35.41	47.92	46.09	9.60	2.75	4.21	48	1352	23.90	109.00	1.60	0.04	56.57	68.13
GEOROC[1]	Northern Iran	TBQ7.2	35.41	47.92	45.54	9.44	2.69	4.21	46	1360	23.50	109.00	1.64	0.03	57.87	66.46
GEOROC[1]	Northern Iran	TBQ7.3	35.41	47.92	45.04	10.23	2.66	4.26	47	1323	23.90	112.00	1.64	0.04	55.36	68.29
GEOROC[1]	Northern Iran	TBQ7.4	35.41	47.92	46.15	9.40	2.68	4.80	44	1376	24.10	110.80	1.68	0.03	57.10	65.95
GEOROC[1]	Northern Iran	TBQ8.1	35.45	47.92	44.80	10.17	2.78	4.12	45	1407	24.70	94.00	1.69	0.03	56.96	55.62
GEOROC[1]	Northern Iran	TBQ8.2	35.45	47.92	45.13	9.81	2.74	4.31	48	1455	23.60	112.00	1.78	0.03	61.65	62.92
GEOROC[1]	Northern Iran	TBQ8.3	35.45	47.92	45.04	9.83	0.72	5.09	43	1488	23.10	109.00	1.71	0.03	64.42	63.74
GEOROC[1]	Northern Iran	TBQ14.1	35.40	47.96	44.45	9.54	2.69	4.65	59	1293	26.30	76.60	1.66	0.05	49.16	46.14
GEOROC[1]	Northern Iran	TBQ14.2	35.40	47.96	44.84	9.61	2.64	4.50	55	1278	25.30	78.00	1.66	0.04	50.51	46.99
GEOROC[1]	Northern Iran	TBQ15.1	35.39	47.96	66.44	1.06	3.08	5.67	65	970	12.20	61.70	1.01	0.07	79.51	61.09
GEOROC[1]	Northern Iran	TBQ18.1	35.36	47.97	45.57	8.21	3.28	4.40	56	1859	23.60	140.00	1.40	0.03	78.77	100.00
GEOROC[1]	Northern Iran	TBQ19.1	35.36	47.97	46.58	9.27	3.26	4.78	69	1534	26.80	152.00	1.61	0.04	57.24	94.41
GEOROC[1]	Northern Iran	TBQ13.1	35.37	47.97	47.13	8.39	3.35	4.96	59	1912	24.10	155.00	1.51	0.03	79.34	102.65
GEOROC[1]	Northern Iran	TBQ13.2	35.37	47.97	48.00	8.18	3.37	4.89	58	1869	24.00	144.00	1.47	0.03	77.88	97.96
GEOROC[1]	Northern Iran	TBQ12.2	35.37	47.98	49.19	8.62	2.64	4.01	49	1158	20.70	97.30	1.49	0.04	55.94	65.30
GEOROC[1]	Northern Iran	TBQ17.1	35.38	47.99	50.73	8.86	2.81	4.23	55	1182	22.00	90.30	1.53	0.05	53.73	59.02
GEOROC[1]	Northern Iran	TBQ17.2	35.38	47.99	50.84	8.99	2.78	4.42	54	1169	21.70	88.80	1.48	0.05	53.87	60.00
GEOROC[1]	Northern Iran	DMV10	36.00	52.00	48.92	8.06	1.77	4.40	75	3209	29.00	144.10	1.70	0.02	110.66	84.76
GEOROC[1]	Northern Iran	DMV12	35.95	52.11	60.68	2.72	4.42	4.75	125	1704	13.00	95.20	1.10	0.07	131.08	86.55
GEOROC[1]	Northern Iran	DMV13	35.95	52.11	62.60	2.15	4.61	4.56	163	1209	13.00	96.10	1.10	0.13	93.00	87.36
GEOROC[1]	Northern Iran	DMV19G	35.95	52.11	59.18	2.59	4.41	4.84	135	1492	11.00	79.00		0.09	135.64	
GEOROC[1]	Northern Iran	DMV19B	35.95	52.11	59.30	2.73	4.37	4.74	125	1632	12.00	100.40	1.00	0.08	136.00	100.40
GEOROC[1]	Northern Iran	DMV34	35.95	52.11	60.63	2.76	4.36	4.78	117	1406	13.00	89.40	1.30	0.08	108.15	68.77
GEOROC[1]	Northern Iran	DMV147	35.95	52.11	61.20	2.22	4.32	4.87	128	1220	14.00	80.00		0.10	87.14	
GEOROC[1]	Northern Iran	DMV07	35.95	52.11	60.24	2.53	4.39	4.82	126	1621	12.00	95.50	1.10	0.08	135.08	86.82
GEOROC[1]	Northern Iran	DMV116	35.95	52.11	62.04	2.69	4.43	4.73	141	1300	13.00	86.00		0.11	100.00	
GEOROC[1]	Northern Iran	DMV39	35.95	52.11	59.39	2.75	4.43	4.80	121	1430	11.00	92.30	1.00	0.08	130.00	92.30
GEOROC[1]	Northern Iran	DMV132A	35.95	52.11	61.32	2.61	4.39	4.79	130	1340	14.00	84.00		0.10	95.71	
GEOROC[1]	Northern Iran	DMV114	35.95	52.11	62.31	2.37	4.31	4.69	126	1210	15.00	68.00		0.10	80.67	
GEOROC[1]	Northern Iran	DMV108	35.95	52.11	60.13	3.02	4.26	4.71	117	1340	14.00	86.00		0.09	95.71	
GEOROC[1]	Northern Iran	DH30	35.95	52.11	48.76	8.39	3.68	3.79	42	2210	20.90	102.40	1.49	0.02	105.74	68.72
GEOROC[1]	Northern Iran	DH27	35.95	52.11	50.27	6.66	4.00	4.53	51	2200	19.10	148.10	1.29	0.02	115.18	114.81
GEOROC[1]	Northern Iran	DH28	35.95	52.11	50.86	6.66	3.98	5.14	53	2850	8.40	148.40	1.36	0.02	339.29	109.12
GEOROC[1]	Northern Iran	DA5400	35.95	52.11	59.61	1.27	4.30	4.55	125	1340	12.40	84.20	0.77	0.09	108.06	109.35

Source	Location	Sample	Latitude	Longitude	SiO2(wt.%)	MgO(wt.%)	K2O(wt.%)	Na2O(wt.%)	Rb(ppm)	Sr(ppm)	Y(ppm)	La(ppm)	Yb(ppm)	Rb/Sr	Sr/Y	La/Yb
GEOROC[1]	Northern Iran	DA P2	35.95	52.11	59.98	2.61	5.00	5.04	127	1580	12.80	98.00	1.06	0.08	123.44	92.45
GEOROC[1]	Northern Iran	DA4800	35.95	52.11	61.32	2.46	4.36	4.86	131	1370	12.77	91.60	1.04	0.10	107.28	88.08
GEOROC[1]	Northern Iran	Q111	37.00	57.00	48.69	6.95	1.67	4.71	15	730	22.00	31.90	1.50	0.02	33.18	21.27
GEOROC[1]	Northern Iran	Q112	37.00	57.00	49.25	6.85	1.72	4.71	15	733	23.10	32.90	1.40	0.02	31.73	23.50
GEOROC[1]	Northern Iran	Q113	37.00	57.00	47.81	6.98	1.37	3.82	86	1098	22.70	36.00	1.40	0.08	48.37	25.71
GEOROC[1]	Northern Iran	Q115	37.00	57.00	48.34	6.91	1.85	4.57	16	710	22.20	33.00	1.50	0.02	31.98	22.00
GEOROC[1]	Northern Iran	Q116	37.00	57.00	48.12	7.07	1.50	4.36	75	796	23.30	36.20	1.40	0.09	34.16	25.86
GEOROC[1]	Northern Iran	Q117	37.00	57.00	48.75	6.84	2.09	4.69	21	718	22.50	32.70	1.50	0.03	31.91	21.80
GEOROC[1]	Northern Iran	Q120	37.00	57.00	47.03	7.40	1.58	4.67	43	977	22.80	36.40	1.50	0.04	42.85	24.27
GEOROC[1]	Northern Iran	Q122	37.00	57.00	48.69	6.80	1.90	3.44	39	1399	22.40	34.60	1.50	0.03	62.46	23.07
GEOROC[1]	Northern Iran	Q123	37.00	57.00	47.27	7.34	1.49	4.31	56	1128	22.30	34.90	1.50	0.05	50.58	23.27
GEOROC[1]	Northern Iran	Q124	37.00	57.00	47.46	8.05	1.68	4.08	43	883	22.10	33.80	1.40	0.05	39.95	24.14
GEOROC[1]	Eastern Carpathian	C14	47.28	25.22	63.52	2.54	2.40	3.11	154	323	27.80	24.00		0.48	11.62	
GEOROC[1]	Eastern Carpathian	C26	47.22	25.20	59.43	3.32	1.61	3.25	63	337	29.30	21.00		0.19	11.50	
GEOROC[1]	Eastern Carpathian	C24	47.20	25.38	51.58	4.90	1.41	2.79	40	462	29.70	14.00		0.09	15.56	
GEOROC[1]	Eastern Carpathian	C16	47.18	25.42	70.98	0.22	3.22	3.85	124	299	12.20	26.00		0.41	24.51	
GEOROC[1]	Eastern Carpathian	C47	47.13	25.15	51.01	5.73	1.34	2.72	39	448	28.40	14.00		0.09	15.77	
GEOROC[1]	Eastern Carpathian	C18	47.13	25.42	55.83	3.85	0.99	3.11	32	264	26.10	11.00		0.12	10.11	
GEOROC[1]	Eastern Carpathian	C21	47.10	25.40	60.74	1.67	1.91	3.33	67	364	33.30	35.00		0.18	10.93	
GEOROC[1]	Eastern Carpathian	C13	47.10	25.22	63.57	3.89	2.24	3.65	80	366	16.70	26.00		0.22	21.92	
GEOROC[1]	Eastern Carpathian	C5	47.08	25.35	61.76	3.08	3.14	3.48	133	303	25.60	35.00		0.44	11.84	
GEOROC[1]	Eastern Carpathian	C4/PM-C4	47.08	25.35	51.23	4.78	0.82	3.26	21	272	21.28	7.07	1.99	0.08	12.78	3.55
GEOROC[1]	Eastern Carpathian	C7	47.05	25.27	62.35	1.89	1.21	3.80	50	289	21.20	14.00		0.17	13.63	
GEOROC[1]	Eastern Carpathian	C8	47.05	25.30	64.11	1.12	0.92	3.95	32	283	16.00	11.00		0.11	17.69	
GEOROC[1]	Eastern Carpathian	C2	47.05	25.35	66.36	0.82	1.00	4.03	26	290	13.40	14.00		0.09	21.64	
GEOROC[1]	Eastern Carpathian	C3	47.05	25.37	68.60	0.71	1.29	4.43	44	296	14.90	16.00		0.15	19.87	
GEOROC[1]	Eastern Carpathian	C65	47.05	25.25	50.73	5.14	0.87	3.12	23	319	23.70	14.00		0.07	13.46	
GEOROC[1]	Eastern Carpathian	C6	47.05	25.33	53.94	4.42	1.89	3.10	52	481	24.00	21.00		0.11	20.04	
GEOROC[1]	Eastern Carpathian	C10	46.97	25.18	58.03	2.57	1.81	3.37	72	250	30.50	20.00		0.29	8.20	
GEOROC[1]	Eastern Carpathian	C9	46.95	25.32	62.16	3.31	2.12	3.74	63	391	15.80	24.00		0.16	24.75	
GEOROC[1]	Eastern Carpathian	G12	46.90	24.95	54.97	4.23	1.17	2.96	37	283	25.20	13.00		0.13	11.23	
GEOROC[1]	Eastern Carpathian	G9	46.73	25.37	61.23	3.11	1.80	3.50	60	244	21.90	24.00		0.25	11.14	
GEOROC[1]	Eastern Carpathian	G7	46.67	25.32	57.86	3.38	1.33	3.55	40	301	25.10	22.00		0.13	11.99	
GEOROC[1]	Eastern Carpathian	G1	46.62	25.55	56.83	3.86	1.14	3.35	34	281	23.00	17.00		0.12	12.22	
GEOROC[1]	Eastern Carpathian	G32	46.62	25.18	57.87	3.62	1.44	3.63	46	322	23.20	27.00		0.14	13.88	
GEOROC[1]	Eastern Carpathian	G3	46.62	25.43	59.38	3.19	1.38	3.49	41	290	22.30	17.00		0.14	13.00	
GEOROC[1]	Eastern Carpathian	G6	46.60	25.40	66.82	3.32	1.20	3.53	37	274	29.00	19.00		0.13	9.45	
GEOROC[1]	Eastern Carpathian	H28	46.60	25.67	69.93	0.34	2.83	4.28	108	241	14.90	28.00		0.45	16.17	
GEOROC[1]	Eastern Carpathian	G31	46.58	24.95	54.04	4.79	0.78	3.19	18	343	27.00	9.00		0.05	12.70	
GEOROC[1]	Eastern Carpathian	G39	46.58	25.48	58.78	3.01	1.57	3.53	50	316	21.30	22.00		0.16	14.84	
GEOROC[1]	Eastern Carpathian	G40	46.57	25.42	59.03	3.42	1.19	3.72	37	234	23.40	14.00		0.16	10.00	
GEOROC[1]	Eastern Carpathian	H30	46.57	25.58	60.94	3.07	2.07	3.23	76	376	20.60	24.00		0.20	18.25	
GEOROC[1]	Eastern Carpathian	H44	46.53	25.63	67.75	0.10	2.64	4.52	95	305	18.20	27.00		0.31	16.76	

Source	Location	Sample	Latitude	Longitude	SiO2(wt.%)	MgO(wt.%)	K2O(wt.%)	Na2O(wt.%)	Rb(ppm)	Sr(ppm)	Y(ppm)	La(ppm)	Yb(ppm)	Rb/Sr	Sr/Y	La/Yb
GEOROC[1]	Eastern Carpathian	H27	46.45	25.67	65.88	0.79	2.26	4.68	81	274	24.80	35.00		0.30	11.05	
GEOROC[1]	Eastern Carpathian	H24	46.42	25.68	63.15	2.91	1.98	3.46	78	317	21.00	26.00		0.25	15.10	
GEOROC[1]	Eastern Carpathian	H23	46.38	25.65	60.18	3.40	1.74	3.56	63	324	22.00	20.00		0.19	14.73	
GEOROC[1]	Eastern Carpathian	H45	46.38	25.83	64.48	3.12	2.44	2.99	85	754	16.50	25.00		0.11	45.70	
GEOROC[1]	Eastern Carpathian	H21	46.37	25.67	57.90	3.20	1.42	3.50	42	469	23.70	26.00		0.09	19.79	
GEOROC[1]	Eastern Carpathian	H18	46.37	25.70	62.31	2.73	1.94	3.11	78	317	21.20	25.00		0.25	14.95	
GEOROC[1]	Eastern Carpathian	H8	46.35	25.85	61.85	3.12	2.11	3.05	80	308	21.50	25.00		0.26	14.33	
GEOROC[1]	Eastern Carpathian	H20	46.33	25.68	60.60	3.64	2.17	2.82	87	374	26.10	30.00		0.23	14.33	
GEOROC[1]	Eastern Carpathian	H16	46.30	25.80	65.95	2.81	2.55	2.88	102	223	20.30	27.00		0.46	10.99	
GEOROC[1]	Eastern Carpathian	H10	46.18	25.92	67.97	1.66	3.38	4.40	99	1028	11.50	33.00		0.10	89.39	
GEOROC[1]	Eastern Carpathian	H11	46.17	25.82	54.90	3.70	1.61	3.72	35	948	25.60	20.00		0.04	37.03	
GEOROC[1]	Eastern Carpathian	H12	46.15	25.80	63.16	2.18	2.58	4.38	70	871	15.70	38.00		0.08	55.48	
GEOROC[1]	Eastern Carpathian	H17	46.13	25.73	59.06	3.32	1.81	3.47	63	337	26.90	25.00		0.19	12.53	
GEOROC[1]	Eastern Carpathian	H4	46.12	25.85	59.72	3.59	2.18	4.03	53	1181	17.50	28.00		0.05	67.49	
GEOROC[1]	Eastern Carpathian	H7	46.12	25.97	64.61	1.88	3.25	4.64	83	1266	13.70	41.00		0.07	92.41	
GEOROC[1]	Eastern Carpathian	H6	46.12	25.90	68.45	1.38	3.58	4.88	88	1319	8.40	29.00		0.07	157.02	
GEOROC[1]	Eastern Carpathian	H51	46.10	25.67	56.02	3.94	1.22	3.13	42	372	21.70	17.00		0.11	17.14	
GEOROC[1]	Eastern Carpathian	RAC1	46.10	25.55	46.87	9.68	1.63	3.79	38	814	21.60	32.30	1.97	0.05	37.69	16.40
GEOROC[1]	Eastern Carpathian	RAKO	46.08	25.53	47.35	10.26	1.45	3.43	31	709	21.00	29.00	2.05	0.04	33.76	14.15
GEOROC[1]	Eastern Carpathian	RACO	46.08	25.53	47.90	10.29	1.41	3.48	33	692	22.40	29.00	0.33	0.05	30.89	87.88
GEOROC[1]	Eastern Carpathian	RAC2	46.08	25.53	46.80	10.62	1.52	3.31	20	703	20.60	27.00	1.87	0.03	34.13	14.44
GEOROC[1]	Eastern Carpathian	H3	46.07	25.82	58.05	4.49	4.04	3.92	66	2264	18.50	101.00		0.03	122.38	
GEOROC[1]	Eastern Carpathian	H2	46.05	25.82	57.49	4.29	3.32	3.85	54	1540	15.90	48.00		0.03	96.86	
GEOROC[1]	Eastern Carpathian	H13	46.05	25.80	59.51	2.93	1.90	3.86	59	558	18.60	31.00		0.11	30.00	
GEOROC[1]	Eastern Carpathian	RUPEA/RUP	46.03	25.22	54.50	4.51	0.82	4.47	17	653	16.52	19.49	1.39	0.03	39.53	14.02
GEOROC[1]	Eastern Carpathian	MATEIA	46.02	25.37	46.12	9.66	2.03	4.05	52	863	23.50	53.00		0.06	36.72	
GEOROC[1]	Eastern Carpathian	SARATA	46.00	26.00	46.10	7.87	1.62	3.16	12	1050	24.60	62.10	0.33	0.01	42.68	188.18
GEOROC[1]	Eastern Carpathian	HOGHIZ	46.00	26.00	47.21	9.06	1.01	3.70	27	1329	25.00	19.00		0.02	53.16	
GEOROC[1]	Eastern Carpathian	LA GRUIU	46.00	26.00	50.53	9.24	2.30	2.50	26	703	16.00	12.00		0.04	43.94	
GEOROC[1]	Eastern Carpathian	ERES1689	46.00	27.00	45.54	6.83	1.66	4.73	61	873	27.00	62.18	2.66	0.07	32.33	23.38
GEOROC[1]	Eastern Carpathian	BOGATA 1	46.00	26.00	47.00	8.93	1.85	4.00	51	740	24.90	37.00		0.07	29.72	
GEOROC[1]	Eastern Carpathian	BOGATA 2	46.00	26.00	49.01	7.83	1.66	3.54	36	177	23.70	33.80	0.32	0.20	7.47	105.63
GEOROC[1]	Eastern Carpathian	BARC	46.00	26.00	46.94	8.96	1.89	4.11	49	739	23.60	31.30	2.01	0.07	31.31	15.57
GEOROC[1]	Eastern Carpathian	COMANA	46.00	25.20	48.19	1.77	1.82	3.82	43	808	23.10	36.80	0.32	0.05	34.98	115.00
GEOROC[1]	Eastern Carpathian	ER	46.00	27.00	46.31	7.25	1.79	4.58	60	925	28.00	52.00		0.06	33.04	
GEOROC[1]	Eastern Carpathian	GRU2	46.00	25.00	46.08	8.82	2.02	3.46	40	820	22.00	31.70	1.83	0.05	37.27	17.32
GEOROC[1]	Eastern Carpathian	RP8N	46.00	26.00	63.51	2.23	3.01	4.68	73	1400	7.00	32.00	0.85	0.05	200.00	37.65
GEOROC[1]	Eastern Carpathian	BARC	45.97	25.00	46.63	9.22	1.91	3.91	43	775	23.80	32.80	2.21	0.06	32.56	14.84
GEOROC[1]	Eastern Carpathian	C1/PM-C1	46.97	25.40	52.22	6.14	2.04	3.28	75	250	27.07	22.26	2.60	0.30	9.24	8.56
GEOROC[1]	Eastern Carpathian	G19	46.82	25.20	57.86	3.26	1.34	3.65	43	236	22.80	14.00		0.18	10.35	
GEOROC[1]	Greater Caucasus (W)	50-8	43.53	42.84	57.81	3.64	2.43	3.90	61	603	20.00			0.10	30.15	
GEOROC[1]	Greater Caucasus (W)	50-3	43.53	42.84	55.93	4.37	2.55	4.02	59	738	20.00			0.08	36.90	
GEOROC[1]	Greater Caucasus (W)	E-37	43.52	42.78	64.26	2.13	3.14	4.03	99	348	20.00			0.28	17.40	

Source	Location	Sample	Latitude	Longitude	SiO2(wt.%)	MgO(wt.%)	K2O(wt.%)	Na2O(wt.%)	Rb(ppm)	Sr(ppm)	Y(ppm)	La(ppm)	Yb(ppm)	Rb/Sr	Sr/Y	La/Yb
GEOROC[1]	Greater Caucasus (W)	E-38	43.52	42.78	65.12	1.92	3.20	4.00	122	347	17.00			0.35	20.41	
GEOROC[1]	Greater Caucasus (W)	E-42	43.52	42.78	64.39	2.29	2.98	3.97	113	353	18.00			0.32	19.61	
Chernyshev et al., (2014)[2]	Greater Caucasus (W)	MI-39/97	43.35	42.44	72.21	0.73	3.88	2.89	211	225	11.00			0.94	20.45	
Chernyshev et al., (2014)[2]	Greater Caucasus (W)	MI-40/97	43.35	42.44	71.61	0.76	3.68	3.27	202	231	8.00			0.87	28.88	
Chernyshev et al., (2014)[2]	Greater Caucasus (W)	218-3/0	43.35	42.44	71.47	1.23	3.63	3.73	165	225	21.00			0.73	10.71	
Chernyshev et al., (2014)[2]	Greater Caucasus (W)	221-1/0	43.35	42.44	71.14	0.87	3.97	3.47	180	202	22.00			0.89	9.18	
Chernyshev et al., (2014)[2]	Greater Caucasus (W)	236-1/0	43.35	42.44	70.82	1.57	3.99	2.89	191	254	24.00			0.75	10.58	
Chernyshev et al., (2014)[2]	Greater Caucasus (W)	Bt-15/97	43.35	42.44	70.91	1.13	3.47	3.68	142	226	18.00			0.63	12.56	
Chernyshev et al., (2014)[2]	Greater Caucasus (W)	Bt-5/97	43.35	42.44	67.26	1.54	3.00	4.27	121	263	21.00			0.46	12.52	
Chernyshev et al., (2014)[2]	Greater Caucasus (W)	209/0	43.35	42.44	69.38	1.29	3.35	3.87	153	270	20.00			0.57	13.50	
Chernyshev et al., (2014)[2]	Greater Caucasus (W)	217-1/0	43.35	42.44	69.83	1.09	3.41	4.16	159	243	21.00			0.65	11.57	
Chernyshev et al., (2014)[2]	Greater Caucasus (W)	Bt-8/97	43.35	42.44	69.40	0.99	3.42	4.08	158	273	22.00			0.58	12.41	
Chernyshev et al., (2014)[2]	Greater Caucasus (W)	Bt-13/97	43.35	42.44	68.68	1.14	3.72	4.17	191	287	7.00			0.67	41.00	
Chernyshev et al., (2014)[2]	Greater Caucasus (W)	Bt-2/97	43.35	42.44	69.01	1.16	3.65	4.03	183	287	6.00			0.64	47.83	
Chernyshev et al., (2014)[2]	Greater Caucasus (W)	210-2/0	43.35	42.44	72.61	0.14	3.70	3.87	165	255	16.00			0.65	15.94	
Chernyshev et al., (2014)[2]	Greater Caucasus (W)	211/0	43.35	42.44	70.11	1.39	3.54	3.77	153	251	18.00			0.61	13.94	
GEOROC[1]	Greater Caucasus (W)	E-20	43.33	42.68	68.43	1.36	3.55	3.91	161	234	16.00			0.69	14.63	
GEOROC[1]	Greater Caucasus (W)	E-23	43.33	42.68	65.60	1.85	3.21	4.13	131	311	24.00			0.42	12.96	
GEOROC[1]	Greater Caucasus (W)	E-34	43.33	42.68	67.89	1.44	3.77	3.87	167	247	17.00			0.68	14.53	
GEOROC[1]	Greater Caucasus (W)	Bt-6	43.30	42.47	68.08	0.91	3.19	3.89	135	368	11.00			0.37	33.45	
GEOROC[1]	Greater Caucasus (W)	Bt-17	43.30	42.47	70.11	0.54	3.51	3.91	142	378	11.00			0.38	34.36	
GEOROC[1]	Greater Caucasus (W)	MI-36	43.30	42.47	68.00	0.85	3.39	4.00	159	389	6.00			0.41	64.83	
GEOROC[1]	Greater Caucasus (W)	MI-37	43.30	42.47	68.45	0.88	3.35	3.77	160	359	8.00			0.45	44.88	
GEOROC[1]	Greater Caucasus (W)	MI-41	43.30	42.47	67.62	1.02	3.24	4.02	148	392	8.00			0.38	49.00	
GEOROC[1]	Greater Caucasus (W)	MI-43	43.30	42.47	68.20	0.86	3.41	3.92	158	365	15.00			0.43	24.33	
GEOROC[1]	Greater Caucasus (W)	Ea-19	43.30	42.47	66.13	1.86	3.31	4.00	151	413	6.00			0.37	68.83	
GEOROC[1]	Greater Caucasus (W)	Ea-21	43.30	42.47	65.64	1.84	2.77	3.77	114	433	8.00			0.26	54.13	
GEOROC[1]	Greater Caucasus (W)	Ea-22	43.30	42.47	66.27	1.82	2.72	4.27	126	345	9.00			0.37	38.33	
GEOROC[1]	Greater Caucasus (W)	Ea-23	43.30	42.47	65.18	2.10	2.84	3.99	114	355	8.00			0.32	44.38	
GEOROC[1]	Greater Caucasus (W)	Ea-25	43.30	42.47	68.11	1.60	3.90	3.99	177	283	7.00			0.63	40.43	
GEOROC[1]	Greater Caucasus (W)	Ea-26	43.30	42.47	67.33	1.77	3.20	3.27	139	375	9.00			0.37	41.67	
GEOROC[1]	Greater Caucasus (W)	Ea-28	43.30	42.47	66.42	1.82	3.13	4.00	138	392	6.00			0.35	65.33	
GEOROC[1]	Greater Caucasus (W)	Ea-29	43.30	42.47	67.22	1.89	3.24	3.63	144	375	9.00			0.38	41.67	
GEOROC[1]	Greater Caucasus (W)	Ea-30	43.30	42.47	66.58	2.25	3.18	3.27	140	398	7.00			0.35	56.86	
GEOROC[1]	Greater Caucasus (W)	Ea-31	43.30	42.47	66.59	1.92	3.40	3.53	143	390	7.00			0.37	55.71	
GEOROC[1]	Greater Caucasus (W)	E-1	43.30	42.47	70.10	0.93	3.54	3.63	152	298	21.00			0.51	14.19	
GEOROC[1]	Greater Caucasus (W)	E-2	43.30	42.47	67.87	1.05	3.15	3.91	132	384	21.00			0.34	18.29	
GEOROC[1]	Greater Caucasus (W)	E-3	43.30	42.47	68.25	0.85	3.26	3.87	139	373	21.00			0.37	17.76	
GEOROC[1]	Greater Caucasus (W)	E-4	43.30	42.47	68.35	1.19	3.00	3.90	125	335	20.00			0.37	16.75	
GEOROC[1]	Greater Caucasus (W)	E-5	43.30	42.47	69.14	1.08	3.16	3.88	128	316	20.00			0.41	15.80	
GEOROC[1]	Greater Caucasus (W)	E-6	43.30	42.47	69.38	1.09	3.59	3.89	162	296	21.00			0.55	14.10	
GEOROC[1]	Greater Caucasus (W)	E-7	43.30	42.47	70.41	1.18	3.72	3.56	166	279	19.00			0.59	14.68	
GEOROC[1]	Greater Caucasus (W)	E-8	43.30	42.47	69.78	1.09	3.36	3.87	147	288	20.00			0.51	14.40	
GEOROC[1]	Greater Caucasus (W)	LIP90L	43.72	44.05	75.75	0.11	4.42	4.21	280	53	22.00	20.10	1.79	5.28	2.41	11.23

Source	Location	Sample	Latitude	Longitude	SiO2(wt.%)	MgO(wt.%)	K2O(wt.%)	Na2O(wt.%)	Rb(ppm)	Sr(ppm)	Y(ppm)	La(ppm)	Yb(ppm)	Rb/Sr	Sr/Y	La/Yb
GEOROC[1]	Greater Caucasus (W)	25	43.35	42.44	70.10	0.33	3.30	4.06	213	284	6.00	40.70	1.40	0.75	47.33	29.07
GEOROC[1]	Greater Caucasus (W)	20	43.35	42.44	68.70	1.46	4.00	3.76	142	317	9.00	48.90	1.40	0.45	35.22	34.93
GEOROC[1]	Greater Caucasus (W)	26	43.35	42.44	68.52	0.20	3.90	3.88	184	211	11.00	41.40	1.50	0.87	19.18	27.60
GEOROC[1]	Greater Caucasus (W)	27	43.35	42.44	67.20	1.49	2.93	4.06	174	51	14.00	48.10	1.60	3.41	3.64	30.06
GEOROC[1]	Greater Caucasus (W)	43	43.35	42.44	67.27	1.06	3.22	3.74	172	604	14.00	55.00	1.70	0.28	43.14	32.35
GEOROC[1]	Greater Caucasus (W)	32	43.35	42.44	67.20	1.54	3.39	4.21	146	389	11.00	46.90	1.50	0.38	35.36	31.27
GEOROC[1]	Greater Caucasus (W)	45	43.35	42.44	66.46	1.05	3.23	3.60	168	256	13.00	54.70	1.60	0.66	19.69	34.19
GEOROC[1]	Greater Caucasus (W)	48	43.35	42.44	66.40	1.44	3.31	4.31	154	278	13.00	56.60	1.80	0.55	21.38	31.44
GEOROC[1]	Greater Caucasus (W)	22	43.35	42.44	67.70	0.82	3.37	4.31	183	378	11.00	40.20	1.30	0.48	34.36	30.92
GEOROC[1]	Greater Caucasus (W)	23	43.35	42.44	67.50	1.45	3.37	4.32	152	207	12.00	43.00	1.50	0.73	17.25	28.67
GEOROC[1]	Greater Caucasus (W)	7	43.35	42.44	66.80	0.82	3.49	4.20	115	318	9.00	36.10	1.20	0.36	35.33	30.08
GEOROC[1]	Greater Caucasus (W)	6	43.35	42.44	66.79	0.92	3.70	3.34	140	295	14.00	40.20	1.40	0.47	21.07	28.71
GEOROC[1]	Greater Caucasus (W)	396	43.35	42.44	66.80	1.40	3.47	4.23	166	320	25.00	46.30	1.31	0.52	12.80	35.34
GEOROC[1]	Greater Caucasus (W)	338	43.35	42.44	67.40	1.57	3.66	4.00	158	332	28.00	48.40	1.43	0.48	11.86	33.85
GEOROC[1]	Greater Caucasus (W)	10	43.35	42.44	67.50	1.86	3.26	4.31	92	342	11.00	40.30	1.52	0.27	31.09	26.51
GEOROC[1]	Greater Caucasus (W)	9	43.35	42.44	67.40	1.44	4.00	4.15	110	370	5.00	47.90	1.44	0.30	74.00	33.26
GEOROC[1]	Greater Caucasus (W)	340	43.35	42.44	64.40	1.64	3.22	4.54	118	308	29.00	39.80	1.55	0.38	10.62	25.68
Lebedev et al., (2009)[3]	Greater Caucasus (E)	SU-III	42.70	44.43	60.64	5.24	2.16	4.83	59	712	20.00			0.08	35.60	
Lebedev et al., (2009)[3]	Greater Caucasus (E)	SU-II	42.70	44.43	60.09	4.15	1.72	5.20	45	598	20.00			0.08	29.90	
Lebedev et al., (2009)[3]	Greater Caucasus (E)	SU-I	42.70	44.43	58.45	4.56	1.90	5.11	55	608	25.00			0.09	24.32	
Lebedev et al., (2009)[3]	Greater Caucasus (E)	SU-24	42.70	44.43	60.92	4.34	1.62	5.31	45	541	25.00			0.08	21.64	
Lebedev et al., (2009)[3]	Greater Caucasus (E)	SU-23	42.70	44.43	58.22	4.79	1.79	5.13	40	691	24.00			0.06	28.79	
Lebedev et al., (2009)[3]	Greater Caucasus (E)	SU-22	42.70	44.43	65.29	3.15	1.65	5.40	45	570	18.00			0.08	31.67	
Lebedev et al., (2009)[3]	Greater Caucasus (E)	SU-21	42.69	44.43	63.87	2.90	1.86	5.07	55	479	19.00			0.11	25.21	
Lebedev et al., (2009)[3]	Greater Caucasus (E)	SU-20	42.69	44.43	64.24	3.75	1.78	5.37	50	564	19.00			0.09	29.68	
Lebedev et al., (2009)[3]	Greater Caucasus (E)	GZ-71	42.69	44.43	66.86	3.50	0.48	4.43	16	521	21.00			0.03	24.81	
Lebedev et al., (2009)[3]	Greater Caucasus (E)	GZ-70*	42.69	44.43	64.23	3.62	2.15	3.81	63	523	20.00			0.12	26.15	
Lebedev et al., (2009)[3]	Greater Caucasus (E)	SU-8	42.69	44.43	60.99	4.42	1.87	3.81	86	535	26.00			0.16	20.58	
Lebedev et al., (2009)[3]	Greater Caucasus (E)	SU-6	42.69	44.43	65.90	3.19	0.33	5.07	13	620	35.00			0.02	17.71	
GEOROC[1]	Southeastern Lesser Caucasus	105	39.86	46.02	51.23	6.04	1.42	4.22	16	910	31.00	40.00	2.40	0.02	29.35	16.67
GEOROC[1]	Southeastern Lesser Caucasus	129	39.86	46.02	48.35	6.74	1.96	3.61	30	1310	30.00	65.00	2.70	0.02	43.67	24.07
GEOROC[1]	Southeastern Lesser Caucasus	132	39.86	46.02	48.88	6.29	1.92	4.00	32	1360	34.00	63.00	2.40	0.02	40.00	26.25
GEOROC[1]	Southeastern Lesser Caucasus	134	39.86	46.02	48.05	6.81	1.73	4.18	34	1490	29.00	62.00	2.20	0.02	51.38	28.18
GEOROC[1]	Southeastern Lesser Caucasus	21	39.86	46.02	51.84	4.42	2.92	4.14	37	2400	16.00	76.00	1.80	0.02	150.00	42.22
GEOROC[1]	Southeastern Lesser Caucasus	57	39.86	46.02	49.42	5.27	2.48	3.22	27	2600	23.00	77.00	1.90	0.01	113.04	40.53
GEOROC[1]	Southeastern Lesser Caucasus	208	39.86	46.02	52.97	3.65	3.16	4.39	53	1900	23.00	77.00	2.30	0.03	82.61	33.48
GEOROC[1]	Southeastern Lesser Caucasus	19/P	39.86	46.02	50.50	5.30	2.90	4.50	43	1780	15.00	73.50	2.35	0.02	118.67	31.28
GEOROC[1]	Southeastern Lesser Caucasus	53	39.86	46.02	53.32	3.81	2.80	5.03	34	1420	16.00	59.00	1.80	0.02	88.75	32.78
GEOROC[1]	Southeastern Lesser Caucasus	87	39.86	46.02	53.05	4.12	2.77	4.27	36	1615	16.00	66.00	2.10	0.02	100.94	31.43
GEOROC[1]	Southeastern Lesser Caucasus	109	39.86	46.02	54.92	3.76	2.17	3.70	39	1130	24.00	69.00	2.00	0.03	47.08	34.50
GEOROC[1]	Southeastern Lesser Caucasus	36/P	39.86	46.02	54.90	3.90	3.00	4.60	42	1433	15.00	72.00	2.35	0.03	95.53	30.64
GEOROC[1]	Southeastern Lesser Caucasus	120	39.86	46.02	55.67	4.66	2.60	4.22	55	730	21.00	52.00	2.00	0.08	34.76	26.00

Source	Location	Sample	Latitude	Longitude	SiO2(wt.%)	MgO(wt.%)	K2O(wt.%)	Na2O(wt.%)	Rb(ppm)	Sr(ppm)	Y(ppm)	La(ppm)	Yb(ppm)	Rb/Sr	Sr/Y	La/Yb
GEOROC[1]	Southeastern Lesser Caucasus	167	39.86	46.02	54.31	3.84	2.96	4.78	37	1700	27.00	69.00	2.20	0.02	62.96	31.36
GEOROC[1]	Southeastern Lesser Caucasus	174	39.86	46.02	54.01	3.37	3.25	4.53	43	1700	25.00	80.00	2.00	0.03	68.00	40.00
GEOROC[1]	Southeastern Lesser Caucasus	180	39.86	46.02	55.21	2.50	3.11	5.04	43	1190	27.00	69.00	2.20	0.04	44.07	31.36
GEOROC[1]	Southeastern Lesser Caucasus	13	39.86	46.02	57.66	3.18	3.01	3.85	55	1360	24.00	60.00	1.80	0.04	56.67	33.33
GEOROC[1]	Southeastern Lesser Caucasus	25	39.86	46.02	58.52	3.23	2.80	4.00	49	1275	32.00	60.00	1.90	0.04	39.84	31.58
GEOROC[1]	Southeastern Lesser Caucasus	33	39.86	46.02	59.85	2.67	3.11	4.38	66	1615	32.00	70.00	2.00	0.04	50.47	35.00
GEOROC[1]	Southeastern Lesser Caucasus	143	39.86	46.02	57.08	2.29	2.87	4.53	40	1647	16.00	59.00	2.20	0.02	102.94	26.82
GEOROC[1]	Southeastern Lesser Caucasus	160	39.86	46.02	59.28	2.79	3.46	4.65	56	1360	19.00	67.00	2.10	0.04	71.58	31.90
GEOROC[1]	Southeastern Lesser Caucasus	185	39.86	46.02	57.85	2.77	2.89	4.53	48	790	15.00	48.00	1.30	0.06	52.67	36.92
GEOROC[1]	Southeastern Lesser Caucasus	73/P	39.86	46.02	67.80	1.10	4.00	5.50	70	1356	10.00	72.00	2.10	0.05	135.60	34.29
GEOROC[1]	Southeastern Lesser Caucasus	TO141.1	39.20	46.47	76.54	0.12	4.34	4.61	184	0	10.00	18.90	1.28	0.00	0.00	14.77
GEOROC[1]	Southeastern Lesser Caucasus	MA22	39.20	46.47	77.09	0.03	4.49	4.37	198	0	10.00	23.80	1.32	0.00	0.00	18.03
GEOROC[1]	Southeastern Lesser Caucasus	142.1	39.20	46.47	76.68	0.12	4.61	4.19	169	19	12.00	32.70	1.39	8.89	1.58	23.53
GEOROC[1]	Southeastern Lesser Caucasus	MA21	39.20	46.47	77.10	0.04	4.66	4.21	170	19	10.00	33.70	1.33	8.95	1.90	25.34
GEOROC[1]	Southeastern Lesser Caucasus	OA409	39.20	46.47	76.75	0.11	4.59	4.44	209	0	11.00	23.50	1.30	0.00	0.00	18.08
GEOROC[1]	Southeastern Lesser Caucasus	OA410	39.20	46.47	76.63	0.11	4.48	4.50	187	26	11.00	19.10	1.11	7.19	2.36	17.21
GEOROC[1]	Southeastern Lesser Caucasus	MA18	39.20	46.47	76.93	0.04	4.47	4.41	181	19	9.00			9.53	2.11	
GEOROC[1]	Southeastern Lesser Caucasus	MA19	39.20	46.47	77.01	0.05	4.86	4.06	174	16	11.00	30.70	1.32	10.88	1.45	23.26
GEOROC[1]	Southeastern Lesser Caucasus	MA20	39.20	46.47	76.98	0.03	4.45	4.48	185	0	9.00			0.00	0.00	
GEOROC[1]	Southeastern Lesser Caucasus	OA411	39.90	45.85	76.82	0.09	4.79	4.14	159	11	13.00	38.80	0.33	14.45	0.85	117.58
GEOROC[1]	Southeastern Lesser Caucasus	MA16	39.90	45.85	77.13	0.04	4.68	4.21	151	16	11.00			9.44	1.45	
GEOROC[1]	Northern Lesser Caucasus	S21.1	41.13	43.69	63.94	2.63	2.84	4.07	58	395	21.47	38.74	1.92	0.15	18.37	20.17
GEOROC[1]	Northern Lesser Caucasus	S1.1	41.05	43.82	57.88	3.05	1.98	4.00	44	516	27.21	31.01	2.49	0.08	18.97	12.43
GEOROC[1]	Northern Lesser Caucasus	S1.2	41.05	43.82	57.84	3.11	1.98	4.07	46	529	27.86	30.28	2.50	0.09	18.97	12.12
GEOROC[1]	Northern Lesser Caucasus	S10.1	41.04	44.08	54.07	5.80	1.17	4.08	17	650	25.39	28.54	2.24	0.03	25.61	12.76
GEOROC[1]	Northern Lesser Caucasus	S11.1	41.01	43.94	60.03	2.95	1.97	3.71	48	538	22.02	28.37	1.92	0.09	24.42	14.76
GEOROC[1]	Northern Lesser Caucasus	S12.1	41.07	44.10	65.45	1.51	2.33	3.95	69	477	11.17	27.78	0.96	0.14	42.71	29.07
GEOROC[1]	Northern Lesser Caucasus	S14.1	41.03	44.13	51.85	6.38	1.15	3.97	18	574	27.79	23.42	2.43	0.03	20.66	9.64
GEOROC[1]	Northern Lesser Caucasus	S14.2	41.03	44.14	51.28	5.46	1.08	4.25	18	746	28.58	23.02	2.51	0.02	26.08	9.19
GEOROC[1]	Northern Lesser Caucasus	S14.3	41.04	44.15	50.94	6.45	1.07	3.83	17	610	31.95	22.95	2.76	0.03	19.08	8.30
GEOROC[1]	Northern Lesser Caucasus	S14.4	41.04	44.15	50.68	6.61	1.09	4.11	17	718	30.50	22.69	2.72	0.02	23.54	8.34
GEOROC[1]	Northern Lesser Caucasus	S14.5	41.04	44.95	53.21	5.94	1.48	3.55	22	671	27.09	35.39	2.64	0.03	24.78	13.39
GEOROC[1]	Northern Lesser Caucasus	S15.1	41.02	43.91	59.31	4.06	1.96	4.07	41	504	18.84	33.89	1.82	0.08	26.75	18.64
GEOROC[1]	Northern Lesser Caucasus	S16.1	41.02	43.89	58.50	3.26	1.76	4.16	39	527	26.80	31.04	2.49	0.07	19.66	12.48
GEOROC[1]	Northern Lesser Caucasus	S17.1	41.01	43.86	58.86	3.23	1.72	4.06	37	519	24.73	29.87	2.45	0.07	21.00	12.19
GEOROC[1]	Northern Lesser Caucasus	S18.1	41.08	43.80	57.56	4.66	1.59	3.95	26	492	24.01	32.14	2.55	0.05	20.51	12.61
GEOROC[1]	Northern Lesser Caucasus	S19.1	41.07	43.66	52.33	5.98	1.19	4.06	14	617	27.55	27.50	2.66	0.02	22.38	10.32
GEOROC[1]	Northern Lesser Caucasus	S2.1	41.04	43.82	58.27	2.87	2.00	3.94	44	503	25.72	28.53	2.32	0.09	19.54	12.31
GEOROC[1]	Northern Lesser Caucasus	S2.2	41.05	43.80	52.65	5.88	1.21	4.39	18	606	30.44	26.67	2.58	0.03	19.90	10.33
GEOROC[1]	Northern Lesser Caucasus	S20.1	41.09	43.66	61.95	3.00	2.47	4.15	49	453	18.82	37.60	1.84	0.11	24.05	20.41
GEOROC[1]	Northern Lesser Caucasus	S20.2	41.09	43.66	61.84	3.06	2.57	4.11	51	458	18.58	36.38	1.81	0.11	24.67	20.12
GEOROC[1]	Northern Lesser Caucasus	S22.1	41.08	43.61	62.27	3.20	2.28	3.78	55	392	19.70	29.19	1.87	0.14	19.89	15.57
GEOROC[1]	Northern Lesser Caucasus	S23.1	41.09	43.59	62.39	3.30	2.23	4.18	58	405	19.15	26.71	1.84	0.14	21.16	14.50

Source	Location	Sample	Latitude	Longitude	SiO2(wt.%)	MgO(wt.%)	K2O(wt.%)	Na2O(wt.%)	Rb(ppm)	Sr(ppm)	Y(ppm)	La(ppm)	Yb(ppm)	Rb/Sr	Sr/Y	La/Yb
GEOROC[1]	Northern Lesser Caucasus	S24.1	41.10	43.56	62.70	2.63	2.48	4.01	64	435	17.77	26.65	1.63	0.15	24.49	16.40
GEOROC[1]	Northern Lesser Caucasus	S25.2	41.08	43.77	53.08	6.57	1.22	4.42	21	595	27.04	24.30	2.45	0.04	21.99	9.91
GEOROC[1]	Northern Lesser Caucasus	S26.2	40.91	43.86	52.24	4.72	1.19	4.41	18	555	31.69	21.21	2.94	0.03	17.52	7.22
GEOROC[1]	Northern Lesser Caucasus	S26.3	40.91	43.86	53.22	4.35	1.49	4.18	25	605	29.64	29.49	2.88	0.04	20.40	10.22
GEOROC[1]	Northern Lesser Caucasus	S27.1	41.02	43.80	54.41	3.71	1.50	4.22	31	618	27.90	28.64	2.67	0.05	22.14	10.72
GEOROC[1]	Northern Lesser Caucasus	S28.1	41.08	44.31	51.45	5.83	1.20	4.24	21	640	30.29	23.56	2.73	0.03	21.14	8.62
GEOROC[1]	Northern Lesser Caucasus	S28.2	41.08	44.31	50.38	6.13	1.00	4.19	14	543	33.16	21.64	3.37	0.02	16.39	6.41
GEOROC[1]	Northern Lesser Caucasus	S29.1	41.05	44.34	51.03	5.75	1.18	4.19	21	566	30.37	20.39	2.84	0.04	18.63	7.19
GEOROC[1]	Northern Lesser Caucasus	S3.2	41.07	43.94	58.28	3.18	1.91	4.12	44	523	28.75	31.33	2.52	0.08	18.20	12.41
GEOROC[1]	Northern Lesser Caucasus	S30.1	41.12	43.90	58.82	3.55	1.84	4.33	45	584	20.20	27.53	1.83	0.08	28.92	15.05
GEOROC[1]	Northern Lesser Caucasus	S30.2	41.11	43.92	58.97	3.46	1.90	3.98	47	603	20.42	27.83	1.87	0.08	29.53	14.86
GEOROC[1]	Northern Lesser Caucasus	S30.3	41.11	43.92	68.24	1.55	2.34	3.79	74	433	12.45	26.11	1.11	0.17	34.76	23.44
GEOROC[1]	Northern Lesser Caucasus	S4.1	41.08	43.95	59.73	3.44	1.90	3.96	46	531	21.54	27.34	1.86	0.09	24.66	14.72
GEOROC[1]	Northern Lesser Caucasus	S4.2	41.08	43.95	57.67	3.36	1.83	3.77	42	562	27.22	31.76	2.36	0.07	20.64	13.45
GEOROC[1]	Northern Lesser Caucasus	S5.1	41.09	43.94	61.57	2.95	2.04	3.85	48	635	16.12	33.31	1.38	0.08	39.38	24.22
GEOROC[1]	Northern Lesser Caucasus	S6.1	41.09	43.94	62.18	2.75	2.18	3.82	52	662	16.22	36.64	1.35	0.08	40.79	27.17
GEOROC[1]	Northern Lesser Caucasus	S7.1	41.05	43.67	60.67	3.14	2.26	4.27	53	676	19.49	39.07	1.63	0.08	34.67	23.92
GEOROC[1]	Northern Lesser Caucasus	S7.2	41.05	43.67	60.43	3.40	2.28	4.23	52	690	19.38	39.10	1.63	0.08	35.60	23.95
GEOROC[1]	Northern Lesser Caucasus	S7.3	41.05	43.67	60.86	3.39	2.24	3.87	53	696	19.19	38.31	1.60	0.08	36.27	23.93
GEOROC[1]	Northern Lesser Caucasus	S7.4	41.05	43.67	60.15	3.75	2.13	4.27	50	701	19.03	38.16	1.64	0.07	36.85	23.23
GEOROC[1]	Northern Lesser Caucasus	S7.5	41.05	43.67	60.30	3.66	2.13	4.14	50	705	19.31	38.12	1.62	0.07	36.50	23.51
GEOROC[1]	Northern Lesser Caucasus	S8.1	41.01	43.94	60.22	2.71	1.98	4.18	48	539	22.60	28.40	1.93	0.09	23.85	14.69
GEOROC[1]	Northern Lesser Caucasus	S9.1	41.07	44.00	64.15	1.95	2.04	4.34	45	558	11.28	26.00	0.98	0.08	49.47	26.54
GEOROC[1]	Northern Lesser Caucasus	S9.2	41.07	43.99	65.80	1.77	2.21	4.12	57	540	11.65	27.38	1.01	0.11	46.39	27.23
GEOROC[1]	Northern Lesser Caucasus	L3.1	41.09	44.70	50.64	6.68	1.36	3.81	23	595	29.70	29.00	2.70	0.04	20.03	10.74
GEOROC[1]	Northern Lesser Caucasus	L3.3	41.09	44.71	50.82	5.79	1.22	4.13	18	519	32.90	29.00	3.00	0.03	15.78	9.67
GEOROC[1]	Northern Lesser Caucasus	L5.1	41.07	44.61	50.61	5.13	1.16	3.95	12	502	28.10	22.00	2.50	0.02	17.86	8.80
GEOROC[1]	Northern Lesser Caucasus	L5.2	41.07	44.62	50.94	5.57	1.23	4.02	17	492	30.80	24.00	2.80	0.03	15.97	8.57
GEOROC[1]	Northern Lesser Caucasus	L5.3	41.07	44.61	48.83	6.72	0.81	3.65	11	473	31.80	18.00	2.90	0.02	14.87	6.21
GEOROC[1]	Northern Lesser Caucasus	L5.4	41.07	44.61	49.94	6.24	0.93	4.00	13	509	29.70	22.00	2.70	0.03	17.14	8.15
GEOROC[1]	Northern Lesser Caucasus	L5.5	41.07	44.61	50.15	6.27	1.01	4.08	15	502	32.20	23.00	2.90	0.03	15.59	7.93
GEOROC[1]	Northern Lesser Caucasus	L5.6	41.07	44.61	51.63	6.08	0.99	3.99	15	503	31.30	22.00	2.80	0.03	16.07	7.86
GEOROC[1]	Northern Lesser Caucasus	L5.7	41.07	44.61	50.94	6.08	1.11	4.01	16	556	29.60	25.00	2.60	0.03	18.78	9.62
GEOROC[1]	Northern Lesser Caucasus	L8.1	41.10	44.69	52.37	5.77	1.30	4.09	19	594	27.20	30.00	2.40	0.03	21.84	12.50
GEOROC[1]	Northern Lesser Caucasus	L8.2	41.10	44.69	52.34	5.18	1.42	4.13	21	574	28.00	30.00	2.50	0.04	20.50	12.00
GEOROC[1]	Northern Lesser Caucasus	L8.3	41.10	44.69	50.61	5.42	1.10	4.15	17	504	30.50	23.00	2.80	0.03	16.52	8.21
GEOROC[1]	Northern Lesser Caucasus	L8.4	41.09	44.69	50.62	5.39	1.08	4.11	16	513	30.40	23.00	2.80	0.03	16.88	8.21
GEOROC[1]	Northern Lesser Caucasus	L8.5	41.09	44.69	51.02	6.33	1.09	4.10	14	527	29.20	24.00	2.70	0.03	18.05	8.89
GEOROC[1]	Northern Lesser Caucasus	L8.6	41.09	44.69	51.92	5.64	1.15	4.31	15	552	30.30	25.00	2.70	0.03	18.22	9.26
GEOROC[1]	Northern Lesser Caucasus	L8.7	41.09	44.69	50.58	6.75	0.97	3.97	10	546	28.80	22.00	2.70	0.02	18.96	8.15
GEOROC[1]	Northern Lesser Caucasus	L8.8	41.10	44.70	51.47	6.40	1.13	3.88	14	508	29.20	26.00	2.70	0.03	17.40	9.63
GEOROC[1]	Northern Lesser Caucasus	L8.9	41.09	44.69	51.05	6.55	1.20	4.11	16	525	29.70	26.00	2.70	0.03	17.68	9.63
GEOROC[1]	Northwestern Lesser Caucasus	MA1	41.87	44.33	76.14	0.07	4.73	4.07	127	73	16.00			1.74	4.56	

Source	Location	Sample	Latitude	Longitude	SiO2(wt.%)	MgO(wt.%)	K2O(wt.%)	Na2O(wt.%)	Rb(ppm)	Sr(ppm)	Y(ppm)	La(ppm)	Yb(ppm)	Rb/Sr	Sr/Y	La/Yb
GEOROC[1]	Northwestern Lesser Caucasus	KAU10	41.87	44.33	75.88	0.13	4.68	4.01	119	98	15.00			1.21	6.53	
GEOROC[1]	Northwestern Lesser Caucasus	KAU1	41.87	44.33	75.56	0.22	4.67	3.96	115	117	17.00	33.30	1.46	0.98	6.88	22.81
GEOROC[1]	Northwestern Lesser Caucasus	MA2	41.87	44.33	75.34	0.20	4.56	4.04	104	147	15.00			0.71	9.80	
GEOROC[1]	Northwestern Lesser Caucasus	YUG-151	41.81	43.45	62.63	2.45	2.17	4.18	33	621	15.00			0.05	41.40	
GEOROC[1]	Northwestern Lesser Caucasus	YUG-150	41.84	43.41	62.50	2.53	2.07	4.21	36	607	13.00			0.06	46.69	
GEOROC[1]	Northwestern Lesser Caucasus	YUG-154	41.74	43.52	61.71	2.40	2.10	4.23	39	575	16.00			0.07	35.94	
GEOROC[1]	Northwestern Lesser Caucasus	YUG-79	41.73	43.46	59.47	2.56	1.94	4.27	25	634	26.00			0.04	24.38	
GEOROC[1]	Northwestern Lesser Caucasus	YUG-155	41.74	43.46	59.47	2.50	1.77	4.36	17	643	22.00			0.03	29.23	
GEOROC[1]	Northwestern Lesser Caucasus	YUG-152	41.80	43.49	59.46	2.58	1.99	4.58	29	632	22.00			0.05	28.73	
GEOROC[1]	Northwestern Lesser Caucasus	AR-1/07	41.36	44.46	58.45	5.28	1.99	3.41	60	480	20.00			0.13	24.00	
GEOROC[1]	Northwestern Lesser Caucasus	YUG-153	41.79	43.58	58.19	2.79	2.04	5.03	27	611	23.00			0.04	26.57	
GEOROC[1]	Northwestern Lesser Caucasus	YUG-156	41.73	43.36	54.65	6.18	1.63	3.91	34	534	23.00			0.06	23.22	
GEOROC[1]	Northwestern Lesser Caucasus	YUG-157	41.76	43.35	52.58	7.45	0.99	3.16	25	369	24.00			0.07	15.38	
Lebedev et al., (2008a)[4]	Northwestern Lesser Caucasus	YUG-2	41.40	43.50	72.21	0.78	2.59	3.50	68	538	6.00			0.13	89.67	
Lebedev et al., (2008a)[4]	Northwestern Lesser Caucasus	YUG-3	41.40	43.50	71.17	0.89	2.47	3.59	64	570	6.00			0.11	95.00	
Lebedev et al., (2008a)[4]	Northwestern Lesser Caucasus	YUG-17	41.42	43.48	64.97	2.13	2.31	3.31	60	402	21.00			0.15	19.14	
Lebedev et al., (2008a)[4]	Northwestern Lesser Caucasus	YUG-32	41.36	43.28	64.20	1.37	2.33	3.82	56	386	29.00			0.15	13.31	
Lebedev et al., (2008a)[4]	Northwestern Lesser Caucasus	YUG-51	41.49	43.38	56.07	3.32	0.83	2.52	12	467	30.00			0.03	15.57	
Lebedev et al., (2008a)[4]	Northwestern Lesser Caucasus	YUG-61	41.32	43.43	56.03	3.41	1.36	3.72	23	458	25.00			0.05	18.32	
Lebedev et al., (2008a)[4]	Northwestern Lesser Caucasus	YUG-50	41.49	43.38	55.91	3.92	0.89	2.98	14	457	27.00			0.03	16.93	
Lebedev et al., (2008a)[4]	Northwestern Lesser Caucasus	YUG-9	41.43	43.47	55.86	4.49	1.23	3.35	16	544	26.00			0.03	20.92	
Lebedev et al., (2008a)[4]	Northwestern Lesser Caucasus	YUG-60	41.32	43.43	55.82	3.59	1.54	3.73	29	469	27.00			0.06	17.37	
Lebedev et al., (2008a)[4]	Northwestern Lesser Caucasus	YUG-8	41.43	43.47	55.51	4.38	1.17	3.24	17	562	26.00			0.03	21.62	
Lebedev et al., (2008a)[4]	Northwestern Lesser Caucasus	YUG-52	41.49	43.38	55.01	5.36	0.85	2.89	11	458	27.00			0.02	16.96	
Lebedev et al., (2008a)[4]	Northwestern Lesser Caucasus	YUG-10	41.43	43.47	54.83	3.53	1.42	3.75	20	464	29.00			0.04	16.00	
Lebedev et al., (2008a)[4]	Northwestern Lesser Caucasus	YUG-26	41.37	43.28	54.51	3.64	0.95	3.44	15	410	27.00			0.04	15.19	
Lebedev et al., (2008a)[4]	Northwestern Lesser Caucasus	YUG-30	41.37	43.28	54.49	3.36	0.86	3.35	12	435	26.00			0.03	16.73	
Lebedev et al., (2008a)[4]	Northwestern Lesser Caucasus	YUG-53	41.48	43.38	54.21	5.64	0.84	2.90	14	448	27.00			0.03	16.59	
Lebedev et al., (2008a)[4]	Northwestern Lesser Caucasus	YUG-12	41.43	43.47	54.18	3.98	1.19	3.64	16	478	25.00			0.03	19.12	
Lebedev et al., (2008a)[4]	Northwestern Lesser Caucasus	YUG-56	41.33	43.38	54.17	3.38	1.34	3.91	19	555	30.00			0.03	18.50	
Lebedev et al., (2008a)[4]	Northwestern Lesser Caucasus	YUG-31	41.37	43.28	54.06	6.86	1.05	3.02	15	436	26.00			0.03	16.77	
Lebedev et al., (2008a)[4]	Northwestern Lesser Caucasus	YUG-18	41.42	43.48	53.98	3.81	0.82	3.50	10	413	23.00			0.02	17.96	
Lebedev et al., (2008a)[4]	Northwestern Lesser Caucasus	YUG-55	41.48	43.37	53.54	4.11	0.97	3.63	12	438	28.00			0.03	15.64	
Lebedev et al., (2008a)[4]	Northwestern Lesser Caucasus	YUG-54	41.48	43.37	53.27	4.64	0.73	3.16	11	457	23.00			0.02	19.87	
Lebedev et al., (2008a)[4]	Northwestern Lesser Caucasus	YUG-7	41.40	43.51	52.88	3.99	1.16	3.92	11	470	26.00			0.02	18.08	
Lebedev et al., (2008a)[4]	Northwestern Lesser Caucasus	YUG-47	41.49	43.38	52.83	4.39	1.20	3.62	13	454	27.00			0.03	16.81	
Lebedev et al., (2008a)[4]	Northwestern Lesser Caucasus	YUG-28	41.37	43.28	52.81	4.65	0.80	3.43	10	441	26.00			0.02	16.96	
Lebedev et al., (2008a)[4]	Northwestern Lesser Caucasus	YUG-46	41.49	43.38	52.57	4.53	1.10	3.65	13	457	29.00			0.03	15.76	
Lebedev et al., (2008a)[4]	Northwestern Lesser Caucasus	YUG-21	41.37	43.27	52.20	4.99	1.14	3.80	14	519	30.00			0.03	17.30	
Lebedev et al., (2008a)[4]	Northwestern Lesser Caucasus	YUG-48	41.49	43.38	52.14	4.81	1.05	3.59	13	472	30.00			0.03	15.73	
Lebedev et al., (2008a)[4]	Northwestern Lesser Caucasus	YUG-13	41.42	43.49	51.89	4.23	0.76	3.24	9	441	23.00			0.02	19.17	
Lebedev et al., (2008a)[4]	Northwestern Lesser Caucasus	YUG-22	41.37	43.28	51.68	7.42	1.11	3.31	15	480	30.00			0.03	16.00	
Lebedev et al., (2008a)[4]	Northwestern Lesser Caucasus	YUG-59	41.29	43.40	50.72	5.47	1.06	3.98	16	501	31.00			0.03	16.16	
Lebedev et al., (2008a)[4]	Northwestern Lesser Caucasus	YUG-35	41.51	43.28	50.70	6.10	1.21	3.97	18	549	34.00			0.03	16.15	

Source	Location	Sample	Latitude	Longitude	SiO2(wt.%)	MgO(wt.%)	K2O(wt.%)	Na2O(wt.%)	Rb(ppm)	Sr(ppm)	Y(ppm)	La(ppm)	Yb(ppm)	Rb/Sr	Sr/Y	La/Yb
Lebedev et al., (2008a)[4]	Northwestern Lesser Caucasus	YUG-57	41.31	43.41	50.44	4.65	0.96	4.34	7	628	33.00			0.01	19.03	
Lebedev et al., (2008a)[4]	Northwestern Lesser Caucasus	YUG-39	41.51	43.28	50.44	6.07	1.17	4.08	17	505	34.00			0.03	14.85	
Lebedev et al., (2008a)[4]	Northwestern Lesser Caucasus	YUG-42	41.51	43.28	50.33	5.52	1.06	3.82	11	506	36.00			0.02	14.06	
Lebedev et al., (2008a)[4]	Northwestern Lesser Caucasus	YUG-40	41.51	43.28	50.18	5.67	1.26	3.97	12	539	34.00			0.02	15.85	
Lebedev et al., (2008a)[4]	Northwestern Lesser Caucasus	YUG-41	41.51	43.28	49.62	5.87	1.15	3.87	12	555	34.00			0.02	16.32	
Lebedev et al., (2008a)[4]	Northwestern Lesser Caucasus	YUG-33	41.51	43.28	49.56	6.51	1.13	3.82	17	536	32.00			0.03	16.75	
Lebedev et al., (2008a)[4]	Northwestern Lesser Caucasus	YUG-58	41.30	43.35	48.85	5.40	0.62	4.03	6	501	33.00			0.01	15.18	
Lebedev et al., (2008b)[5]	Northwestern Lesser Caucasus	YUG-124	41.49	43.88	77.03	0.17	4.52	3.52	124	90	21.00			1.38	4.29	
Lebedev et al., (2008b)[5]	Northwestern Lesser Caucasus	YUG-118	41.50	44.11	69.32	0.65	2.37	3.63	68	615	13.00			0.11	47.31	
Lebedev et al., (2008b)[5]	Northwestern Lesser Caucasus	YUG-113	41.37	44.12	64.27	1.32	1.90	3.51	42	527	18.00			0.08	29.28	
Lebedev et al., (2008b)[5]	Northwestern Lesser Caucasus	YUG-112	41.37	44.13	63.77	1.30	1.80	3.52	42	543	17.00			0.08	31.94	
Lebedev et al., (2008b)[5]	Northwestern Lesser Caucasus	YUG-122	41.60	44.09	58.90	2.55	1.83	3.55	29	533	26.00			0.05	20.50	
Lebedev et al., (2008b)[5]	Northwestern Lesser Caucasus	YUG-115	41.42	44.10	58.42	2.90	2.04	3.47	34	547	28.00			0.06	19.54	
Lebedev et al., (2008b)[5]	Northwestern Lesser Caucasus	YUG-125	41.51	43.87	56.67	3.66	1.63	3.86	25	538	27.00			0.05	19.93	
Lebedev et al., (2008b)[5]	Northwestern Lesser Caucasus	YUG-121	41.60	44.08	56.67	4.07	1.75	3.67	27	582	25.00			0.05	23.28	
Lebedev et al., (2008b)[5]	Northwestern Lesser Caucasus	YUG-116	41.43	44.11	56.61	2.89	1.95	3.97	27	644	25.00			0.04	25.76	
Lebedev et al., (2008b)[5]	Northwestern Lesser Caucasus	YUG-123	41.58	43.97	56.47	3.06	1.82	3.83	27	604	30.00			0.04	20.13	
Lebedev et al., (2008b)[5]	Northwestern Lesser Caucasus	YUG-119	41.60	44.11	56.41	4.79	1.77	3.50	29	598	25.00			0.05	23.92	
Lebedev et al., (2008b)[5]	Northwestern Lesser Caucasus	YUG-120	41.60	44.08	56.27	3.67	1.36	3.58	19	539	23.00			0.04	23.43	
Lebedev et al., (2008b)[5]	Northwestern Lesser Caucasus	YUG-174	41.59	44.13	55.85	4.86	1.53	3.74	21	533	24.00			0.04	22.21	
Lebedev et al., (2008b)[5]	Northwestern Lesser Caucasus	YUG-117	41.45	44.10	53.39	4.34	1.49	3.77	18	672	30.00			0.03	22.40	
Lebedev et al., (2008b)[5]	Northwestern Lesser Caucasus	YUG-114	41.42	44.10	53.35	4.19	1.50	3.92	13	679	28.00			0.02	24.25	
Lebedev et al., (2008b)[5]	Northwestern Lesser Caucasus	YUG-109	41.39	44.42	52.70	5.24	1.40	3.75	27	531	30.00			0.05	17.70	
Lebedev et al., (2008b)[5]	Northwestern Lesser Caucasus	YUG-110	41.32	44.20	51.82	5.17	1.35	4.34	22	519	39.00			0.04	13.31	
Lebedev et al., (2008b)[5]	Northwestern Lesser Caucasus	YUG-101	41.50	44.61	51.38	5.67	0.88	3.87	12	449	34.00			0.03	13.21	
Lebedev et al., (2008b)[5]	Northwestern Lesser Caucasus	YUG-106	41.34	44.34	51.09	5.87	1.27	3.60	24	381	32.00			0.06	11.91	
Lebedev et al., (2008b)[5]	Northwestern Lesser Caucasus	YUG-111	41.32	44.20	50.72	5.29	1.31	4.27	20	508	38.00			0.04	13.37	
Lebedev et al., (2008b)[5]	Northwestern Lesser Caucasus	YUG-103	41.50	44.61	50.65	5.81	0.97	4.25	14	466	39.00			0.03	11.95	
Lebedev et al., (2008b)[5]	Northwestern Lesser Caucasus	YUG-173	41.59	44.13	50.46	7.25	0.68	3.34	9	351	26.00			0.03	13.50	
Lebedev et al., (2008b)[5]	Northwestern Lesser Caucasus	YUG-108	41.35	44.36	49.83	6.73	1.12	3.49	23	396	32.00			0.06	12.38	
Lebedev et al., (2008b)[5]	Northwestern Lesser Caucasus	YUG-100	41.49	44.54	49.78	6.56	0.97	4.18	16	530	34.00			0.03	15.59	
Lebedev et al., (2008b)[5]	Northwestern Lesser Caucasus	YUG-102	41.50	44.61	49.74	6.82	0.95	3.83	14	512	33.00			0.03	15.52	
Lebedev et al., (2008b)[5]	Northwestern Lesser Caucasus	YUG-104	41.34	44.34	49.56	5.91	1.15	3.75	22	349	34.00			0.06	10.26	
Lebedev et al., (2008b)[5]	Northwestern Lesser Caucasus	YUG-107	41.35	44.36	48.96	6.43	1.05	3.62	22	391	32.00			0.06	12.22	
Lebedev et al., (2008b)[5]	Northwestern Lesser Caucasus	YUG-105	41.34	44.34	48.73	7.36	0.98	2.53	23	322	30.00			0.07	10.73	
Nomade et al., (2016)[6]	Northwestern Lesser Caucasus	TS-10-08	41.53	44.12	57.20	4.05	1.97	3.81	39	555	24.60	34.40	2.20	0.07	22.56	15.64
Nomade et al., (2016)[6]	Northwestern Lesser Caucasus	TS-05-08	41.65	44.11	56.10	4.79	1.74	3.99	32	618	24.40	34.80	2.10	0.05	25.34	16.57
Nomade et al., (2016)[6]	Northwestern Lesser Caucasus	TS-08-08	41.57	43.90	55.60	4.86	1.64	4.08	26	572	27.20	34.20	2.40	0.05	21.03	14.25
Nomade et al., (2016)[6]	Northwestern Lesser Caucasus	TS-06-08	41.66	43.92	53.70	4.89	1.57	4.29	24	617	27.10	32.80	2.40	0.04	22.78	13.67
Nomade et al., (2016)[6]	Northwestern Lesser Caucasus	OROZ-04	41.31	44.20	53.10	4.37	1.42	4.23	22	664	26.80	30.40	2.30	0.03	24.79	13.22
Nomade et al., (2016)[6]	Northwestern Lesser Caucasus	T0S3-08	41.65	44.11	53.00	4.88	1.42	4.24	19	632	27.10	30.10	2.30	0.03	23.32	13.09
Nomade et al., (2016)[6]	Northwestern Lesser Caucasus	TS02-08	41.63	44.15	53.00	4.92	1.41	4.26	20	651	26.80	30.00	2.20	0.03	24.28	13.64
Nomade et al., (2016)[6]	Northwestern Lesser Caucasus	TS01-08	41.63	44.15	50.70	8.16	0.94	3.77	10	494	25.00	19.90	2.30	0.02	19.78	8.65
Nomade et al., (2016)[6]	Northwestern Lesser Caucasus	OROZ-06	41.31	44.20	50.60	7.06	1.00	3.89	13	517	27.50	19.90	2.50	0.03	18.79	7.96

Source	Location	Sample	Latitude	Longitude	SiO2(wt.%)	MgO(wt.%)	K2O(wt.%)	Na2O(wt.%)	Rb(ppm)	Sr(ppm)	Y(ppm)	La(ppm)	Yb(ppm)	Rb/Sr	Sr/Y	La/Yb
Nomade et al., (2016)[6]	Northwestern Lesser Caucasus	TS04-08	41.65	44.11	47.00	10.48	0.56	3.11	6	410	28.30	14.50	2.60	0.02	14.49	5.58
Nomade et al., (2016)[6]	Northwestern Lesser Caucasus	OBS-5	41.47	43.87	76.00	0.06	4.69	4.05	128	71	15.40	21.40	1.40	1.81	4.60	15.29
Nomade et al., (2016)[6]	Northwestern Lesser Caucasus	OBS-6	41.48	43.88	74.60	0.08	4.61	3.92	128	77	15.40	23.20	1.40	1.66	4.99	16.57
Nomade et al., (2016)[6]	Northwestern Lesser Caucasus	OBS-1	41.49	43.87	74.50	0.09	4.54	3.80	124	80	15.30	24.60	1.40	1.55	5.22	17.57
Nomade et al., (2016)[6]	Northwestern Lesser Caucasus	BORD-C-1	41.53	43.72	70.70	1.02	2.46	4.39	69	451	7.80	20.00	0.60	0.15	57.83	33.33
Nomade et al., (2016)[6]	Northwestern Lesser Caucasus	SAM-95	41.66	43.76	70.50	0.85	2.73	4.08	91	470	6.00	28.60	0.50	0.19	78.27	57.20
Nomade et al., (2016)[6]	Northwestern Lesser Caucasus	GODO-10-01	41.66	43.67	65.70	2.01	2.06	4.21	46	539	12.80	28.90	1.10	0.09	42.09	26.27
Nomade et al., (2016)[6]	Northwestern Lesser Caucasus	TS-07-08	41.65	43.84	64.90	2.55	1.94	4.04	42	526	11.70	28.70	0.90	0.08	44.97	31.89
Nomade et al., (2016)[6]	Northwestern Lesser Caucasus	SAM-94	41.65	43.73	64.80	1.95	2.07	4.23	45	544	12.40	29.10	1.10	0.08	43.85	26.45
Nomade et al., (2016)[6]	Northwestern Lesser Caucasus	MAR10-01	41.37	44.12	63.50	2.78	1.78	4.03	41	518	12.60	24.60	1.10	0.08	41.10	22.36
Nomade et al., (2016)[6]	Northwestern Lesser Caucasus	SAM-92	41.48	43.75	63.10	1.84	1.56	4.25	31	640	9.10	22.70	0.80	0.05	70.30	28.38
Nomade et al., (2016)[6]	Northwestern Lesser Caucasus	SAM-97	41.45	43.77	61.80	2.27	2.11	4.31	36	650	16.80	36.40	1.40	0.06	38.66	26.00
Nomade et al., (2016)[6]	Northwestern Lesser Caucasus	SAM-10-01	41.55	43.75	61.70	3.50	1.97	3.93	46	575	20.00	33.80	1.80	0.08	28.77	18.78
Nomade et al., (2016)[6]	Northwestern Lesser Caucasus	SAM-96	41.66	43.76	61.30	2.15	1.48	4.30	29	715	10.80	25.40	0.90	0.04	66.16	28.22
GEOROC[1]	Central Lesser Caucasus	G-70/03	40.50	44.89	51.26	4.96	1.97	4.59	25	883	29.00			0.03	30.45	
GEOROC[1]	Central Lesser Caucasus	32G/01	40.46	45.02	59.28	3.56	2.88	4.03	60	946	18.00			0.06	52.56	
GEOROC[1]	Central Lesser Caucasus	G-73/03	40.45	44.79	68.77	1.53	3.90	4.25	65	388	22.00			0.17	17.64	
GEOROC[1]	Central Lesser Caucasus	G-74/03	40.44	44.78	53.77	4.99	2.47	4.58	28	923	27.00			0.03	34.19	
GEOROC[1]	Central Lesser Caucasus	29G/01	40.41	44.69	76.26	0.37	3.81	3.81	133	117	23.00			1.14	5.09	
GEOROC[1]	Central Lesser Caucasus	31G/01	40.39	45.21	57.04	3.78	2.47	3.98	54	756	26.00			0.07	29.08	
GEOROC[1]	Central Lesser Caucasus	30G/01	40.39	45.20	56.94	4.62	2.33	3.86	52	744	18.00			0.07	41.33	
GEOROC[1]	Central Lesser Caucasus	G-5/03	40.38	45.13	56.60	4.04	2.49	4.11	43	736	25.00			0.06	29.44	
GEOROC[1]	Central Lesser Caucasus	28G/01	40.38	44.61	74.90	0.30	3.85	4.43	136	124	25.00			1.10	4.96	
GEOROC[1]	Central Lesser Caucasus	G-117/03	40.36	44.68	71.74	0.29	3.91	4.88	104	274	27.00			0.38	10.15	
GEOROC[1]	Central Lesser Caucasus	9G/01	40.33	44.91	54.76	4.16	2.48	4.61	50	1025	25.00			0.05	41.00	
GEOROC[1]	Central Lesser Caucasus	19G/01	40.32	44.69	70.86	0.59	3.61	5.04	99	332	21.00			0.30	15.81	
GEOROC[1]	Central Lesser Caucasus	26G/01	40.31	44.72	74.80	0.29	3.77	4.14	102	134	13.00			0.76	10.31	
GEOROC[1]	Central Lesser Caucasus	18G/01	40.31	44.68	53.97	4.20	2.38	4.73	40	1171	27.00			0.03	43.37	
GEOROC[1]	Central Lesser Caucasus	23G/01	40.31	44.71	53.85	4.82	2.40	4.69	35	1137	22.00			0.03	51.68	
GEOROC[1]	Central Lesser Caucasus	17G/01	40.31	44.68	54.25	4.12	2.49	4.73	41	1148	19.00			0.04	60.42	
GEOROC[1]	Central Lesser Caucasus	25G/01	40.31	44.73	72.25	0.69	3.40	4.56	85	291	19.00			0.29	15.32	
GEOROC[1]	Central Lesser Caucasus	8G/01	40.31	44.91	57.84	4.36	2.51	3.83	62	655	24.00			0.09	27.29	
GEOROC[1]	Central Lesser Caucasus	5bG/01	40.30	44.93	56.87	3.42	2.72	4.66	53	937	23.00			0.06	40.74	
GEOROC[1]	Central Lesser Caucasus	24G/01	40.30	44.72	75.01	0.22	3.73	4.18	94	155	13.00			0.61	11.92	
GEOROC[1]	Central Lesser Caucasus	5aG/01	40.30	44.92	54.29	4.51	2.18	5.44	49	837	28.00			0.06	29.89	
GEOROC[1]	Central Lesser Caucasus	7G/01	40.30	44.90	57.43	3.93	2.27	3.62	57	693	23.00			0.08	30.13	
GEOROC[1]	Central Lesser Caucasus	6G/01	40.30	44.92	58.99	3.74	2.56	4.20	65	659	25.00			0.10	26.36	
GEOROC[1]	Central Lesser Caucasus	G-47/03	40.30	45.00	57.60	4.56	2.51	3.82	45	765	25.00			0.06	30.60	
GEOROC[1]	Central Lesser Caucasus	4G/01	40.29	44.92	56.16	3.21	2.42	4.52	57	1097	24.00			0.05	45.71	
GEOROC[1]	Central Lesser Caucasus	2G/01	40.29	44.93	54.47	4.66	2.01	4.32	38	720	27.00			0.05	26.67	
GEOROC[1]	Central Lesser Caucasus	13G/01	40.29	44.69	75.29	0.24	3.73	4.20	105	118	15.00			0.89	7.87	
GEOROC[1]	Central Lesser Caucasus	3G/01	40.29	44.92	53.17	4.77	1.88	4.35	31	719	24.00			0.04	29.96	
GEOROC[1]	Central Lesser Caucasus	14G/01	40.29	44.68	75.24	0.27	3.81	4.04	105	127	14.00			0.83	9.07	

Source	Location	Sample	Latitude	Longitude	SiO2(wt.%)	MgO(wt.%)	K2O(wt.%)	Na2O(wt.%)	Rb(ppm)	Sr(ppm)	Y(ppm)	La(ppm)	Yb(ppm)	Rb/Sr	Sr/Y	La/Yb
GEOROC[1]	Central Lesser Caucasus	G-110/03	40.28	44.67	71.35	0.64	3.69	4.84	92	354	25.00			0.26	14.16	
GEOROC[1]	Central Lesser Caucasus	15G/01	40.27	44.70	75.59	0.22	3.90	4.10	111	109	17.00			1.02	6.41	
GEOROC[1]	Central Lesser Caucasus	16G/01	40.27	44.70	76.31	0.20	3.97	3.84	110	106	19.00			1.04	5.58	
GEOROC[1]	Central Lesser Caucasus	G-50/03	40.27	45.05	51.42	5.50	1.69	4.28	21	1321	24.00			0.02	55.04	
GEOROC[1]	Central Lesser Caucasus	G-51/03	40.26	45.05	50.05	5.57	1.61	4.06	21	1419	25.00			0.01	56.76	
GEOROC[1]	Central Lesser Caucasus	G-108/03	40.24	44.60	64.93	1.34	4.28	4.51	86	344	36.00			0.25	9.56	
GEOROC[1]	Central Lesser Caucasus	33G/01	40.19	44.48	57.79	4.17	2.73	4.42	46	982	22.00			0.05	44.64	
GEOROC[1]	Central Lesser Caucasus	34G/01	40.19	44.50	54.27	3.77	2.48	4.78	40	1155	21.00			0.03	55.00	
GEOROC[1]	Central Lesser Caucasus	G-93/03	40.18	45.01	76.01	0.12	4.56	4.34	169	14	28.00			12.07	0.50	
GEOROC[1]	Central Lesser Caucasus	G-43/03	40.11	45.15	48.14	6.80	1.98	4.65	22	1150	26.00			0.02	44.23	
GEOROC[1]	Central Lesser Caucasus	G-84/03	40.10	45.02	76.94	0.02	4.35	4.40	203	18	29.00			11.28	0.62	
GEOROC[1]	Central Lesser Caucasus	G-83/03	40.09	45.02	76.36	0.11	4.76	3.99	174	34	26.00			5.12	1.31	
GEOROC[1]	Central Lesser Caucasus	G-39/03	40.08	45.21	59.46	2.84	2.90	4.52	57	673	26.00			0.08	25.88	
GEOROC[1]	Central Lesser Caucasus	AR-9/07	40.40	44.46	68.25	1.12	2.74	4.47	120	320	20.00			0.38	16.00	
GEOROC[1]	Central Lesser Caucasus	AR-7/07	40.40	44.47	67.81	1.40	2.93	3.97	140	290	20.00			0.48	14.50	
GEOROC[1]	Central Lesser Caucasus	AR-6/07	40.40	44.49	67.79	1.35	3.05	3.91	130	280	20.00			0.46	14.00	
GEOROC[1]	Central Lesser Caucasus	AR-17/07	40.40	44.45	67.95	1.30	2.74	4.21	120	280	20.00			0.43	14.00	
GEOROC[1]	Central Lesser Caucasus	AR-3/07	40.40	44.46	57.89	4.87	1.94	3.61	70	530	20.00			0.13	26.50	
GEOROC[1]	Central Lesser Caucasus	AR-15/07	40.39	44.46	69.93	1.15	3.30	3.89	150	250	20.00			0.60	12.50	
GEOROC[1]	Central Lesser Caucasus	AR-13/07	40.39	44.46	68.15	1.38	3.39	3.87	130	280	10.00			0.46	28.00	
GEOROC[1]	Central Lesser Caucasus	AR-11/07	40.38	44.47	67.87	1.20	3.06	4.26	130	290	20.00			0.45	14.50	
GEOROC[1]	Central Lesser Caucasus	A1/03	40.34	43.78	76.42	0.06	4.57	4.02	180	30	20.00			6.00	1.50	
GEOROC[1]	Central Lesser Caucasus	OA105	40.30	43.75	76.46	0.08	4.44	4.43	147	18	34.00	12.60	3.28	8.17	0.53	3.84
GEOROC[1]	Central Lesser Caucasus	OA101	40.30	43.75	76.27	0.07	4.39	4.56	140	12	33.00			11.67	0.36	
GEOROC[1]	Central Lesser Caucasus	OA104.1	40.30	43.75	76.30	0.07	4.47	4.41	146	20	33.00			7.30	0.61	
GEOROC[1]	Central Lesser Caucasus	OA102.0	40.30	43.75	76.50	0.05	4.41	4.45	145	17	37.00			8.53	0.46	
GEOROC[1]	Central Lesser Caucasus	OA102.1	40.30	43.75	76.35	0.07	4.42	4.51	146	17	34.00	11.40	3.77	8.59	0.50	3.02
GEOROC[1]	Central Lesser Caucasus	OA102.2	40.30	43.75	76.47	0.06	4.41	4.41	148	23	30.00			6.43	0.77	
GEOROC[1]	Central Lesser Caucasus	TO138.1	40.30	43.75	76.34	0.15	4.55	4.21	116	35	25.00	20.90	2.40	3.31	1.40	8.71
GEOROC[1]	Central Lesser Caucasus	Y1.1	40.19	44.50	53.25	3.94	2.51	4.84	38	1215	23.60	67.00	2.10	0.03	51.48	31.90
GEOROC[1]	Central Lesser Caucasus	Y10.1	40.11	44.74	53.96	4.08	2.52	4.65	43	1325	26.10	67.00	2.30	0.03	50.77	29.13
GEOROC[1]	Central Lesser Caucasus	Y2.1	40.19	44.50	53.01	5.14	1.58	4.18	25	682	28.30	38.00	2.70	0.04	24.10	14.07
GEOROC[1]	Central Lesser Caucasus	Y2.2	40.19	44.50	50.87	6.03	1.07	4.17	19	595	31.00	25.00	2.90	0.03	19.19	8.62
GEOROC[1]	Central Lesser Caucasus	Y2.3	40.19	44.50	50.27	6.08	0.99	4.15	16	603	29.90	24.00	2.80	0.03	20.17	8.57
GEOROC[1]	Central Lesser Caucasus	Y2.4	40.19	44.50	49.72	6.26	0.97	4.18	15	613	30.80	24.00	2.80	0.03	19.90	8.57
GEOROC[1]	Central Lesser Caucasus	Y3.1	40.32	44.55	56.42	3.80	2.13	4.16	44	882	24.00	50.00	2.20	0.05	36.75	22.73
GEOROC[1]	Central Lesser Caucasus	Y4.1	40.32	44.58	57.24	4.39	2.80	4.49	45	1015	23.00	59.00	2.20	0.04	44.13	26.82
GEOROC[1]	Central Lesser Caucasus	Y5.1	40.31	44.59	51.77	3.70	2.15	5.04	26	967	32.50	58.00	2.90	0.03	29.75	20.00
GEOROC[1]	Central Lesser Caucasus	Y6.1	40.27	44.70	53.16	6.92	1.89	4.08	31	939	20.10	51.00	1.70	0.03	46.72	30.00
GEOROC[1]	Central Lesser Caucasus	Y7.1	40.25	44.65	51.04	6.06	1.31	3.88	20	595	26.20	30.00	2.40	0.03	22.71	12.50
GEOROC[1]	Central Lesser Caucasus	Y8.1	40.19	44.61	51.69	8.80	2.07	3.64	42	1205	22.10	53.00	1.90	0.04	54.52	27.89
GEOROC[1]	Central Lesser Caucasus	Y9.1	40.17	44.39	50.63	6.01	1.00	4.09	16	603	31.00	24.00	3.00	0.03	19.45	8.00
GEOROC[1]	Central Lesser Caucasus	Y9.2	40.17	44.38	58.75	3.97	1.62	3.82	36	519	18.90	26.00	1.80	0.07	27.46	14.44

Source	Location	Sample	Latitude	Longitude	SiO2(wt.%)	MgO(wt.%)	K2O(wt.%)	Na2O(wt.%)	Rb(ppm)	Sr(ppm)	Y(ppm)	La(ppm)	Yb(ppm)	Rb/Sr	Sr/Y	La/Yb
Gao et al., (2015)[7]	Southeastern Tibet	TC-44	25.17	98.53	66.11	1.37	3.92	3.26	189	279	21.30	95.20	1.81	0.68	13.10	52.60
Gao et al., (2015)[7]	Southeastern Tibet	TC-45	25.17	98.53	66.47	1.42	3.74	3.14	192	286	17.60	64.90	1.43	0.67	16.25	45.38
Gao et al., (2015)[7]	Southeastern Tibet	TC-46	25.17	98.53	65.83	0.95	3.45	3.71	159	336	21.60	62.30	1.72	0.47	15.56	36.22
Gao et al., (2015)[7]	Southeastern Tibet	TC-60C	25.17	98.53	65.24	1.33	4.11	2.81	153	315	29.10	97.90	2.81	0.49	10.82	34.84
Gao et al., (2015)[7]	Southeastern Tibet	TC-61	25.17	98.53	65.12	0.60	3.64	3.23	156	292	20.10	71.40	1.94	0.54	14.53	36.80
Gao et al., (2015)[7]	Southeastern Tibet	TC-62	25.17	98.53	66.59	1.15	3.59	3.34	150	326	19.50	67.80	1.94	0.46	16.72	34.95
Gao et al., (2015)[7]	Southeastern Tibet	TC-63	25.17	98.53	66.10	1.00	3.60	3.39	156	328	25.00	85.90	2.32	0.48	13.12	37.03
Gao et al., (2015)[7]	Southeastern Tibet	TC-64	25.17	98.53	66.64	1.31	3.70	3.56	151	339	19.40	64.20	1.90	0.44	17.47	33.79
Gao et al., (2015)[7]	Southeastern Tibet	TC-47	25.17	98.53	62.16	1.57	3.23	2.36	146	180	19.20	58.30	1.52	0.81	9.38	38.36
Gao et al., (2015)[7]	Southeastern Tibet	TC-48	25.17	98.53	63.49	1.56	3.29	2.65	161	206	18.60	55.70	1.57	0.78	11.08	35.48
Gao et al., (2015)[7]	Southeastern Tibet	TC-49	25.17	98.53	61.54	1.61	3.40	2.30	148	160	18.40	56.20	1.62	0.93	8.70	34.69
Gao et al., (2015)[7]	Southeastern Tibet	TC-50	25.17	98.53	62.12	1.57	3.45	3.05	158	189	17.90	60.10	1.67	0.84	10.56	35.99
Gao et al., (2015)[7]	Southeastern Tibet	TC-51	25.17	98.53	59.13	1.47	3.45	2.09	160	124	17.20	58.80	1.81	1.29	7.21	32.49
Gao et al., (2015)[7]	Southeastern Tibet	TC-52	25.17	98.53	63.35	1.57	3.44	3.29	150	210	17.50	58.50	1.65	0.71	12.00	35.45
Gao et al., (2015)[7]	Southeastern Tibet	TC-53	25.17	98.53	62.97	1.61	3.45	2.42	157	186	19.30	58.30	1.60	0.84	9.64	36.44
Gao et al., (2015)[7]	Southeastern Tibet	TC-54	25.17	98.53	59.04	1.78	3.20	2.22	119	129	21.80	62.70	1.75	0.92	5.92	35.83
Gao et al., (2015)[7]	Southeastern Tibet	TC-55	25.17	98.53	63.73	0.99	3.32	2.66	168	245	26.60	88.50	1.94	0.69	9.21	45.62
Zhou et al., (2012)[8]	Southeastern Tibet	TC-27 1	25.22	98.55	51.68	5.35	2.30	3.90	48	397	26.70	31.50	2.62	0.12	14.87	12.02
Zhou et al., (2012)[8]	Southeastern Tibet	TC-30 1	25.22	98.55	52.47	5.56	2.26	3.81	45	412	26.20	32.00	2.59	0.11	15.73	12.36
Zhou et al., (2012)[8]	Southeastern Tibet	TC-31 1	25.22	98.55	55.53	4.52	2.97	3.79	86	360	25.50	47.40	2.54	0.24	14.12	18.66
Zhou et al., (2012)[8]	Southeastern Tibet	TC-32 1	25.22	98.55	51.88	5.35	2.34	3.74	46	396	28.10	34.50	2.77	0.12	14.09	12.45
Zhou et al., (2012)[8]	Southeastern Tibet	TC-35 1	25.22	98.55	49.29	6.19	1.46	3.39	20	560	24.10	26.80	2.34	0.04	23.24	11.45
Zhou et al., (2012)[8]	Southeastern Tibet	TC-36 1	25.22	98.55	49.87	5.63	2.05	3.85	37	649	23.90	41.80	2.43	0.06	27.15	17.20
Zhou et al., (2012)[8]	Southeastern Tibet	TC-39 1	25.22	98.55	52.61	5.81	3.31	3.86	81	758	23.30	66.70	2.44	0.11	32.53	27.34
Zhou et al., (2012)[8]	Southeastern Tibet	TC-41 1	25.22	98.55	50.01	5.70	1.94	3.34	33	689	24.20	41.90	2.52	0.05	28.47	16.63
Zhou et al., (2012)[8]	Southeastern Tibet	TC-43 1	25.22	98.55	50.16	5.48	1.99	3.49	37	683	24.50	41.60	2.43	0.05	27.88	17.12
Zhou et al., (2012)[8]	Southeastern Tibet	TC-57	25.22	98.50	54.55	4.84	2.79	3.47	78	397	27.20	48.00	2.59	0.20	14.60	18.53
Zhou et al., (2012)[8]	Southeastern Tibet	TC-2	25.22	98.50	52.47	5.41	2.27	3.61	38	497	24.90	39.10	2.47	0.08	19.96	15.83
Zhou et al., (2012)[8]	Southeastern Tibet	TC-5	25.22	98.50	53.04	5.33	2.46	3.98	50	497	24.80	38.40	2.44	0.10	20.04	15.74
Zhou et al., (2012)[8]	Southeastern Tibet	TC-7	25.22	98.50	53.34	5.32	2.34	3.57	47	473	26.10	39.30	2.19	0.10	18.12	17.95
Zhou et al., (2012)[8]	Southeastern Tibet	TC-8	25.22	98.50	54.59	4.88	2.68	4.11	78	476	25.50	39.30	2.19	0.16	18.67	17.95
Zhou et al., (2012)[8]	Southeastern Tibet	TC-9	25.22	98.50	55.80	4.43	2.96	3.53	95	452	25.40	43.40	2.20	0.21	17.80	19.73
Zhou et al., (2012)[8]	Southeastern Tibet	TC-11	25.22	98.50	55.02	4.67	3.00	4.32	86	459	25.40	41.90	2.25	0.19	18.07	18.62
Zhou et al., (2012)[8]	Southeastern Tibet	TC-12	25.22	98.50	58.75	3.48	3.51	4.24	119	523	26.70	72.90	2.46	0.23	19.59	29.63
Zhou et al., (2012)[8]	Southeastern Tibet	TC-15	25.22	98.50	57.13	3.79	3.25	3.58	96	550	25.90	73.60	2.52	0.18	21.24	29.21
Zhou et al., (2012)[8]	Southeastern Tibet	TC-16	25.22	98.50	56.89	3.78	3.26	3.93	96	554	26.30	73.00	2.50	0.17	21.06	29.20
Zhou et al., (2012)[8]	Southeastern Tibet	TC-19	25.22	98.50	60.47	3.02	3.79	3.73	138	467	26.40	71.00	2.27	0.30	17.69	31.28
Zhou et al., (2012)[8]	Southeastern Tibet	TC-21	25.22	98.50	58.35	3.70	3.42	3.80	112	553	27.60	69.50	2.17	0.20	20.04	32.03
Zhou et al., (2012)[8]	Southeastern Tibet	TC-22	25.22	98.50	58.69	3.55	3.45	3.54	112	555	27.00	70.80	2.10	0.20	20.56	33.71
Zhou et al., (2012)[8]	Southeastern Tibet	TC-23	25.22	98.50	57.33	3.76	3.35	3.95	99	551	26.30	70.50	2.36	0.18	20.95	29.87
Zhou et al., (2012)[8]	Southeastern Tibet	TC-26	25.22	98.50	57.34	3.86	3.33	3.85	97	550	25.50	73.70	2.46	0.18	21.57	29.96
Tucker et al., (2013)[9]	Southeastern Tibet	DA09026	25.17	98.01	61.50	2.43	4.02	3.60	137	457	30.10	86.80	2.63	0.30	15.18	33.00
Zhang et al., (2012)[10]	Southeastern Tibet	CQ08-1	25.23	98.63	51.57	6.67	1.89	3.58	35	424	25.51	32.35	2.58	0.08	16.62	12.54
Zhang et al., (2012)[10]	Southeastern Tibet	DY08-1	25.13	98.47	62.04	2.51	3.99	3.63	132	444	30.33	83.31	2.65	0.30	14.62	31.44

Source	Location	Sample	Latitude	Longitude	SiO2(wt.%)	MgO(wt.%)	K2O(wt.%)	Na2O(wt.%)	Rb(ppm)	Sr(ppm)	Y(ppm)	La(ppm)	Yb(ppm)	Rb/Sr	Sr/Y	La/Yb
Zhang et al., (2012)[10]	Southeastern Tibet	GD08-1	25.31	98.48	55.38	5.98	2.42	3.43	80	450	23.15	45.08	2.31	0.18	19.45	19.52
Zhang et al., (2012)[10]	Southeastern Tibet	HT08-1	25.18	98.48	54.73	4.89	2.60	3.94	66	538	26.52	50.50	2.56	0.12	20.27	19.73
Zhang et al., (2012)[10]	Southeastern Tibet	LG08-3	25.04	98.43	54.47	5.23	2.23	3.63	68	417	25.88	38.66	2.45	0.16	16.12	15.78
Zhang et al., (2012)[10]	Southeastern Tibet	MA08-2	25.02	98.43	59.05	3.61	3.25	3.88	94	492	25.28	65.45	2.49	0.19	19.46	26.29
Zhang et al., (2012)[10]	Southeastern Tibet	MA08-3	25.02	98.43	58.73	3.55	3.36	3.83	99	514	26.81	65.10	2.61	0.19	19.15	24.94
Zhang et al., (2012)[10]	Southeastern Tibet	XC08-1	25.03	98.40	58.55	3.71	3.17	3.82	98	551	27.28	67.47	2.69	0.18	20.18	25.08
Zhang et al., (2012)[10]	Southeastern Tibet	CZ08-1	25.24	98.52	54.03	5.91	3.23	3.84	86	706	25.48	69.27	2.46	0.12	27.69	28.16
Zhang et al., (2012)[10]	Southeastern Tibet	CZ08-2	25.24	98.52	54.28	5.81	3.21	3.91	87	735	25.64	68.55	2.46	0.12	28.65	27.87
Zhang et al., (2012)[10]	Southeastern Tibet	HS08-1	25.28	98.48	58.81	3.38	3.11	3.30	110	484	24.68	75.99	2.19	0.23	19.60	34.70
Zhang et al., (2012)[10]	Southeastern Tibet	JS08-2	25.23	98.48	55.06	4.88	2.58	3.73	69	423	24.98	42.74	2.37	0.16	16.92	18.03
Zhang et al., (2012)[10]	Southeastern Tibet	JS08-3	25.23	98.48	55.39	4.79	2.60	3.78	69	437	25.33	43.67	2.47	0.16	17.24	17.68
Zhang et al., (2012)[10]	Southeastern Tibet	TS08-1	25.23	98.47	58.93	4.06	3.25	3.63	99	518	25.97	71.41	2.44	0.19	19.94	29.27
Zhang et al., (2012)[10]	Southeastern Tibet	BH08-1	25.11	98.55	66.92	1.79	3.61	3.47	157	314	19.95	67.23	1.96	0.50	15.74	34.30
Zhang et al., (2012)[10]	Southeastern Tibet	LW08-3	25.05	98.55	66.69	0.94	3.66	3.27	157	320	21.84	72.53	2.08	0.49	14.66	34.87
Zhang et al., (2012)[10]	Southeastern Tibet	TT08-2	24.75	98.63	62.05	2.76	3.25	3.62	95	564	25.38	76.97	2.45	0.17	22.21	31.42
Zhang et al., (2012)[10]	Southeastern Tibet	TT08-4	24.74	98.64	61.24	2.92	3.43	3.35	88	582	25.48	81.40	2.47	0.15	22.83	32.96
Zhang et al., (2012)[10]	Southeastern Tibet	YW08-2	25.42	98.50	62.12	1.92	4.13	0.91	177	114	28.21	51.60	3.22	1.55	4.03	16.02
Zhang et al., (2012)[10]	Southeastern Tibet	DP08-1	25.13	98.47	61.25	2.68	3.78	3.71	128	484	28.95	80.86	2.62	0.26	16.71	30.86
Zhang et al., (2012)[10]	Southeastern Tibet	HY08-1	25.22	98.58	56.04	4.90	2.84	3.54	84	355	25.97	49.32	2.60	0.24	13.67	18.97
Zhang et al., (2012)[10]	Southeastern Tibet	MB08-1	24.93	98.67	49.25	8.11	1.96	2.89	37	657	23.44	53.48	2.40	0.06	28.02	22.28
Zhang et al., (2012)[10]	Southeastern Tibet	TT08-6	24.68	98.65	49.08	8.22	1.68	3.53	38	734	21.05	41.68	2.14	0.05	34.85	19.48
Zhang et al., (2012)[10]	Southeastern Tibet	WH08-2	24.89	98.66	49.53	8.14	1.51	3.03	21	930	23.32	61.72	2.32	0.02	39.89	26.60
Zhang et al., (2012)[10]	Southeastern Tibet	XY08-1	25.21	98.58	50.91	5.18	1.93	3.78	39	510	24.51	34.42	2.51	0.08	20.81	13.71
Shi et al., (2012)[11]	Southeastern Tibet	SD018-1	24.72	98.65	62.93	1.88	3.64	3.54	118	521	31.50	84.20	2.58	0.23	16.52	32.64
Shi et al., (2012)[11]	Southeastern Tibet	SD018-2	24.72	98.65	62.85	1.77	3.62	3.53	113	502	33.80	88.90	2.83	0.22	14.85	31.41
Shi et al., (2012)[11]	Southeastern Tibet	SD024-2-1	24.72	98.64	60.84	2.36	3.33	3.62	102	651	26.00	75.30	2.33	0.16	25.03	32.32
Shi et al., (2012)[11]	Southeastern Tibet	SD024-2-2	24.72	98.64	60.77	2.32	3.34	3.66	100	643	25.50	75.70	2.42	0.16	25.21	31.28
Li et al., (2015)[12]	Southeastern Tibet	TC1	25.20	98.58	62.02	1.97	3.39	3.19	119	445	20.70	66.10	1.85	0.27	21.50	35.73
Li et al., (2015)[12]	Southeastern Tibet	TC7	25.20	98.58	66.90	0.67	3.58	3.20	145	309	17.40	63.00	1.71	0.47	17.76	36.84
Li et al., (2015)[12]	Southeastern Tibet	TC16	25.20	98.58	56.42	3.61	2.95	3.48	79	486	24.50	59.20	2.24	0.16	19.84	26.43
Xu et al., (2012)[13]	Southeastern Tibet	TC-01	25.21	98.49	55.61	3.53	2.20	3.80	46	471	22.65	36.10	2.58	0.10	20.79	13.99
Xu et al., (2012)[13]	Southeastern Tibet	TC-02	25.21	98.49	56.07	3.77	1.85	3.44	47	424	23.75	34.87	2.55	0.11	17.87	13.67
Xu et al., (2012)[13]	Southeastern Tibet	TC-04	25.21	98.49	55.69	3.87	2.16	4.21	46	463	22.78	37.20	2.47	0.10	20.34	15.06
Xu et al., (2012)[13]	Southeastern Tibet	TC-05	25.21	98.49	58.45	3.07	2.57	3.67	72	474	24.18	44.02	2.63	0.15	19.59	16.74
Xu et al., (2012)[13]	Southeastern Tibet	TC-06	25.21	98.49	55.54	3.29	2.30	3.79	52	374	22.19	38.48	2.53	0.14	16.85	15.21
Xu et al., (2012)[13]	Southeastern Tibet	TC-07	25.26	98.50	53.28	3.37	2.34	3.56	45	392	23.20	36.67	2.65	0.11	16.88	13.84
Xu et al., (2012)[13]	Southeastern Tibet	TC-08	25.26	98.50	52.20	4.66	1.84	3.42	35	424	23.76	40.47	2.62	0.08	17.86	15.45
Xu et al., (2012)[13]	Southeastern Tibet	TC-09	25.26	98.50	56.43	3.22	2.35	3.71	53	284	23.62	40.29	2.59	0.19	12.02	15.56
Xu et al., (2012)[13]	Southeastern Tibet	TC-10	25.26	98.50	52.34	3.52	2.70	2.87	62	528	22.51	49.37	2.58	0.12	23.44	19.14
Xu et al., (2012)[13]	Southeastern Tibet	TC-11	25.26	98.50	61.13	3.03	3.18	3.71	96	279	24.80	72.69	2.59	0.34	11.25	28.07
Li and Liu, (2012)[14]	Southeastern Tibet	HSK01	25.17	98.50	58.78	4.05	3.38	3.60	103	542	26.51	74.22	2.42	0.19	20.45	30.67
Li and Liu, (2012)[14]	Southeastern Tibet	HSK02	25.17	98.50	59.07	3.35	3.03	3.29	124	478	23.74	76.28	2.19	0.26	20.13	34.83
Li and Liu, (2012)[14]	Southeastern Tibet	HSK03	25.17	98.50	59.69	3.47	3.64	3.55	118	483	25.51	68.64	2.47	0.24	18.93	27.79
Li and Liu, (2012)[14]	Southeastern Tibet	HSK04	25.17	98.50	59.86	3.11	3.19	3.35	113	390	20.35	62.27	1.87	0.29	19.16	33.30

Source	Location	Sample	Latitude	Longitude	SiO2(wt.%)	MgO(wt.%)	K2O(wt.%)	Na2O(wt.%)	Rb(ppm)	Sr(ppm)	Y(ppm)	La(ppm)	Yb(ppm)	Rb/Sr	Sr/Y	La/Yb
Li and Liu, (2012)[14]	Southeastern Tibet	HSK05	25.17	98.50	54.51	4.64	2.71	3.97	60	442	28.17	46.00	2.77	0.14	15.69	16.61
Li and Liu, (2012)[14]	Southeastern Tibet	HSK06	25.17	98.50	53.80	5.01	2.57	3.83	47	468	23.78	44.64	2.28	0.10	19.68	19.58
Li and Liu, (2012)[14]	Southeastern Tibet	DYS01	25.17	98.50	61.39	2.44	3.98	3.55	130	481	30.37	86.84	2.84	0.27	15.84	30.58
Li and Liu, (2012)[14]	Southeastern Tibet	DYS02	25.17	98.50	60.54	2.53	3.88	3.61	129	448	27.35	77.32	2.64	0.29	16.38	29.29
Li and Liu, (2012)[14]	Southeastern Tibet	DYS03	25.17	98.50	67.68	1.69	3.75	3.25	187	294	20.21	68.13	1.83	0.64	14.55	37.23
Li and Liu, (2012)[14]	Southeastern Tibet	DYS04	25.17	98.50	64.64	1.91	3.94	2.91	153	273	19.34	61.75	1.81	0.56	14.12	34.12
Li and Liu, (2012)[14]	Southeastern Tibet	DYS05	25.17	98.50	58.58	2.68	3.68	3.52	114	489	28.85	83.51	2.88	0.23	16.95	29.00
Li and Liu, (2012)[14]	Southeastern Tibet	MAS01	25.17	98.50	49.16	7.21	2.09	3.07	44	489	26.26	43.02	2.50	0.09	18.62	17.21
Li and Liu, (2012)[14]	Southeastern Tibet	MAS02	25.17	98.50	50.65	6.74	2.11	3.53	45	615	28.05	44.96	2.58	0.07	21.93	17.43
Li and Liu, (2012)[14]	Southeastern Tibet	MAS03	25.17	98.50	50.85	6.72	2.07	3.62	44	638	27.89	43.19	2.56	0.07	22.88	16.87
Li and Liu, (2012)[14]	Southeastern Tibet	MAS04	25.17	98.50	51.26	5.67	2.08	3.81	45	558	28.78	43.66	2.60	0.08	19.39	16.79
Li and Liu, (2012)[14]	Southeastern Tibet	MAS05	25.17	98.50	57.44	3.62	3.26	3.72	95	590	27.33	68.36	2.66	0.16	21.59	25.70
Li and Liu, (2012)[14]	Southeastern Tibet	MAS06	25.17	98.50	51.53	5.65	1.76	3.49	36	466	22.22	32.19	2.36	0.08	20.97	13.64
Li and Liu, (2012)[14]	Southeastern Tibet	MAS07	25.17	98.50	58.45	3.65	3.36	3.71	109	566	27.53	69.70	2.68	0.19	20.56	26.01
Zhang et al., (2008)[15]	Northen Tibet (W)	YCN-1	36.28	81.48	53.70	4.16	3.73	3.70	99	1239	18.20	125.00	1.32	0.08	68.08	94.70
Zhang et al., (2008)[15]	Northen Tibet (W)	YCN-2	36.28	81.48	53.80	4.21	3.74	3.50	103	1281	15.00	125.00	1.08	0.08	85.40	115.74
Zhang et al., (2008)[15]	Northen Tibet (W)	YCN-3	36.28	81.48	53.71	4.36	3.94	3.80	107	1266	18.90	125.00	1.40	0.08	66.98	89.29
Zhang et al., (2008)[15]	Northen Tibet (W)	YCN-4	36.28	81.48	53.75	4.28	3.94	3.70	101	1235	20.50	131.00	1.53	0.08	60.24	85.62
Zhang et al., (2008)[15]	Northen Tibet (W)	YCN-6	36.28	81.48	54.08	4.49	3.98	3.62	98	1203	24.50	126.00	1.91	0.08	49.10	65.97
Zhang et al., (2008)[15]	Northen Tibet (W)	YCN-7	36.28	81.48	53.44	4.10	3.96	3.81	102	1155	24.60	131.00	1.89	0.09	46.95	69.31
Zhang et al., (2008)[15]	Northen Tibet (W)	YCN-8	36.28	81.48	52.96	4.23	3.94	3.93	132	1370	23.40	122.00	1.83	0.10	58.55	66.67
Zhang et al., (2008)[15]	Northen Tibet (W)	PL-2	36.28	81.48	50.81	3.39	4.13	5.46	113	1286	23.58	172.30	1.86	0.09	54.54	92.63
Zhang et al., (2008)[15]	Northen Tibet (W)	PL-3	36.28	81.48	52.91	3.69	4.13	3.83	119	1414	22.21	174.50	1.67	0.08	63.67	104.49
Zhang et al., (2008)[15]	Northen Tibet (W)	PL-5	36.28	81.48	53.11	4.05	4.14	3.99	125	1476	23.55	176.90	1.84	0.08	62.68	96.14
Zhang et al., (2008)[15]	Northen Tibet (W)	PL-7	36.28	81.48	52.87	3.99	3.98	4.44	115	1171	21.73	126.20	1.78	0.10	53.89	70.90
Zhang et al., (2008)[15]	Northen Tibet (W)	PL-9	36.28	81.48	55.18	3.97	4.14	3.64	119	1060	24.59	144.00	2.05	0.11	43.11	70.24
Zhang et al., (2008)[15]	Northen Tibet (W)	PL-12	36.28	81.48	54.61	4.14	4.06	3.52	123	1163	23.79	136.50	2.01	0.11	48.89	67.91
Wang and Zhang, (2011)[16]	Northen Tibet (W)	CY1-1	36.28	81.48	54.51	4.22	4.27	3.64	115	1157	29.20	132.00	2.08	0.10	39.62	63.46
Wang and Zhang, (2011)[16]	Northen Tibet (W)	CY1-2	36.28	81.48	54.08	4.21	4.20	3.52	109	1143	28.80	127.00	2.03	0.10	39.69	62.56
Wang and Zhang, (2011)[16]	Northen Tibet (W)	CY1-4	36.28	81.48	54.36	3.95	4.30	3.64	111	1192	28.30	124.00	1.92	0.09	42.12	64.58
Wang and Zhang, (2011)[16]	Northen Tibet (W)	CY1-5	36.28	81.48	54.36	3.98	4.31	3.66	112	1199	29.00	129.00	2.00	0.09	41.34	64.50
Wang and Zhang, (2011)[16]	Northen Tibet (W)	CY1-6	36.28	81.48	53.08	4.11	4.24	3.80	108	1410	28.10	158.00	1.79	0.08	50.18	88.27
Wang and Zhang, (2011)[16]	Northen Tibet (W)	CY1-7	36.28	81.48	54.24	4.00	4.27	3.64	108	1173	28.60	128.00	1.94	0.09	41.01	65.98
Wang and Zhang, (2011)[16]	Northen Tibet (W)	CY1-8	36.28	81.48	54.57	4.28	4.25	3.56	114	1142	27.70	123.00	1.94	0.10	41.23	63.40
Wang and Zhang, (2011)[16]	Northen Tibet (W)	CY1-9	36.28	81.48	54.54	4.18	4.29	3.56	114	1156	27.70	122.00	1.83	0.10	41.73	66.67
Wang and Zhang, (2011)[16]	Northen Tibet (W)	CY1-10	36.28	81.48	54.11	4.12	4.24	3.54	112	1145	28.60	128.00	1.95	0.10	40.03	65.64
Wang and Zhang, (2011)[16]	Northen Tibet (W)	CY1-11	36.28	81.48	54.58	4.25	4.27	3.57	113	1145	28.10	125.00	1.91	0.10	40.75	65.45
Wang and Zhang, (2011)[16]	Northen Tibet (W)	CY2-1	36.28	81.48	54.09	4.04	4.28	3.62	110	1222	29.30	130.00	1.98	0.09	41.71	65.66
Wang and Zhang, (2011)[16]	Northen Tibet (W)	CY2-2	36.28	81.48	53.57	3.98	4.20	3.56	109	1219	28.80	131.00	1.99	0.09	42.33	65.83
Wang and Zhang, (2011)[16]	Northen Tibet (W)	CY2-3	36.28	81.48	54.12	4.03	4.28	3.61	106	1185	28.50	129.00	1.96	0.09	41.58	65.82
Wang and Zhang, (2011)[16]	Northen Tibet (W)	CY2-4	36.28	81.48	54.17	4.21	4.24	3.52	112	1180	29.10	128.00	1.98	0.09	40.55	64.65
Wang and Zhang, (2011)[16]	Northen Tibet (W)	CY2-5	36.28	81.48	54.32	4.26	4.26	3.56	111	1168	28.60	128.00	1.93	0.10	40.84	66.32
Wang and Zhang, (2011)[16]	Northen Tibet (W)	CY2-6	36.28	81.48	55.12	3.80	3.96	3.40	107	1215	28.70	129.00	1.89	0.09	42.33	68.25

Source	Location	Sample	Latitude	Longitude	SiO2(wt.%)	MgO(wt.%)	K2O(wt.%)	Na2O(wt.%)	Rb(ppm)	Sr(ppm)	Y(ppm)	La(ppm)	Yb(ppm)	Rb/Sr	Sr/Y	La/Yb
Wang and Zhang, (2011)[16]	Northen Tibet (W)	CY2-7	36.28	81.48	54.37	4.15	4.27	3.60	113	1189	28.50	129.00	1.95	0.10	41.72	66.15
Wang and Zhang, (2011)[16]	Northen Tibet (W)	CY2-8	36.28	81.48	54.16	4.25	4.22	3.55	109	1137	27.90	124.00	1.89	0.10	40.75	65.61
Ning et al., (2009)[17]	Northen Tibet (W)	B33062	35.48	82.80	59.39	2.19	4.22	3.13	190	854	32.92	182.90	2.24	0.22	25.94	81.65
Ning et al., (2009)[17]	Northen Tibet (W)	B33065	35.48	82.80	60.70	1.97	4.30	3.44	218	858	30.96	201.60	1.93	0.25	27.71	104.46
Ning et al., (2009)[17]	Northen Tibet (W)	B33067	35.48	82.80	51.81	4.85	2.84	3.10	98	751	24.61	107.40	1.82	0.13	30.52	59.01
Ning et al., (2009)[17]	Northen Tibet (W)	B33070	35.48	82.80	53.43	5.13	2.72	3.36	90	693	24.54	99.70	1.84	0.13	28.24	54.18
Ning et al., (2009)[17]	Northen Tibet (W)	B33071	35.48	82.80	54.25	4.58	2.67	3.29	88	663	25.15	97.80	1.88	0.13	26.36	52.02
Wang et al., (2012)[18]	Northen Tibet (E)	2509*	35.97	90.80	73.13	0.26	5.08	3.21	586	29	7.01	27.60	0.51	20.42	4.09	54.12
Wang et al., (2012)[18]	Northen Tibet (E)	2059ay	35.97	90.80	71.86	0.24	5.03	3.26	545	43	6.83	31.90	0.46	12.76	6.25	69.35
Wang et al., (2012)[18]	Northen Tibet (E)	2511-1*	35.97	90.79	69.24	0.57	5.47	3.04	476	72	9.84	79.80	0.62	6.66	7.27	128.71
Wang et al., (2012)[18]	Northen Tibet (E)	2011a*	35.85	90.48	75.50	0.24	4.13	3.13	454	31	5.50	11.50	0.39	14.74	5.60	29.49
Wang et al., (2012)[18]	Northen Tibet (E)	1P2JD7-1*	35.79	90.43	72.64	0.25	3.95	4.17	923	7	4.02	10.00	0.32	136.74	1.68	31.25
Wang et al., (2012)[18]	Northen Tibet (E)	1P2JD7-1ay	35.79	90.43	72.43	0.02	4.14	4.06	810	9	4.79	11.50	0.35	87.19	1.94	32.86
Wang et al., (2016)[19]	Northen Tibet (E)	5123-2	34.43	89.13	65.04	1.11	4.78	3.65	226	498	22.43	152.80	1.56	0.45	22.18	98.01
Wang et al., (2016)[19]	Northen Tibet (E)	5124-1	34.42	89.19	65.39	0.98	5.10	3.22	250	439	22.78	182.50	1.53	0.57	19.29	119.28
Wang et al., (2016)[19]	Northen Tibet (E)	5124-2	34.42	89.19	65.54	1.07	4.89	3.41	242	528	23.86	182.30	1.60	0.46	22.14	113.72
Wang et al., (2016)[19]	Northen Tibet (E)	5126-1	34.40	89.24	65.64	1.01	4.99	3.74	275	506	23.77	198.50	1.63	0.54	21.30	121.63
Wang et al., (2016)[19]	Northen Tibet (E)	5127-3	34.39	89.26	65.73	0.97	5.13	3.41	240	445	23.48	182.60	1.56	0.54	18.96	116.75
Wang et al., (2016)[19]	Northen Tibet (E)	5133-1	34.39	89.28	65.67	1.04	5.16	3.31	252	495	22.87	199.10	1.51	0.51	21.64	131.51
Wang et al., (2016)[19]	Northen Tibet (E)	5125-1	34.41	89.21	66.05	1.00	5.14	3.17	243	474	18.20	144.50	1.29	0.51	26.03	112.19
Wang et al., (2016)[19]	Northen Tibet (E)	5127-1	34.39	89.26	66.25	1.01	5.26	3.18	250	442	18.88	150.70	1.37	0.57	23.43	109.84
Wang et al., (2016)[19]	Northen Tibet (E)	11WL59-2	34.53	90.55	62.98	1.37	4.52	3.47	172	639	24.26	150.00	1.59	0.27	26.34	94.40
Wang et al., (2016)[19]	Northen Tibet (E)	11WL59-3	34.53	90.55	63.07	1.47	4.62	3.51	170	633	22.14	137.90	1.43	0.27	28.57	96.43
Wang et al., (2016)[19]	Northen Tibet (E)	11WL59-4	34.53	90.55	63.02	1.58	4.58	3.50	179	656	25.21	147.80	1.51	0.27	26.01	97.75
Wang et al., (2016)[19]	Northen Tibet (E)	11WL61-3	34.54	90.55	63.26	1.41	4.63	3.51	173	648	22.00	131.20	1.47	0.27	29.47	89.01
Wang et al., (2016)[19]	Northen Tibet (E)	11WL61-4	34.54	90.55	63.00	1.46	4.65	3.52	173	643	22.38	136.20	1.39	0.27	28.74	98.34
Wang et al., (2016)[19]	Northen Tibet (E)	11WL60-3	34.54	90.55	63.10	1.51	4.63	3.51	174	658	23.62	144.00	1.48	0.26	27.86	97.30
Wang et al., (2016)[19]	Northen Tibet (E)	D5160-GS1	34.84	88.92	67.14	0.94	5.08	3.36	210	420	7.05	82.30	0.70	0.50	59.57	118.25
Wang et al., (2016)[19]	Northen Tibet (E)	D7535C1	34.84	88.92	68.05	0.87	5.08	3.38	179	586	8.78	74.39	1.06	0.31	66.71	70.38
Wang et al., (2016)[19]	Northen Tibet (E)	MB1-1	33.65	90.31	63.56	1.39	4.42	3.60	194	550	23.60	156.00	2.05	0.35	23.31	76.10
Wang et al., (2016)[19]	Northen Tibet (E)	MB1-4	33.65	90.31	63.44	1.30	4.48	3.43	199	560	22.20	136.00	2.01	0.36	25.23	67.66
Wang et al., (2016)[19]	Northen Tibet (E)	MB2	33.65	90.31	63.58	1.43	4.52	3.65	205	550	23.00	144.00	2.09	0.37	23.91	68.90
Wang et al., (2016)[19]	Northen Tibet (E)	MB4	33.65	90.31	63.16	1.34	4.55	3.54	192	620	20.90	138.00	1.81	0.31	29.67	76.24
Wang et al., (2016)[19]	Northen Tibet (E)	C1601-1	33.65	90.31	65.73	0.83	4.82	3.36	251	400	25.20	122.00	2.51	0.63	15.87	48.61
Wang et al., (2016)[19]	Northen Tibet (E)	C1603-1h	33.65	90.31	65.20	1.11	4.64	3.32	238	520	25.80	142.00	2.38	0.46	20.16	59.66
Wang et al., (2016)[19]	Northen Tibet (E)	2303	35.75	90.65	71.40	0.48	5.23	3.15	425	91	6.07	42.20	0.59	4.67	14.99	71.74
Wang et al., (2016)[19]	Northen Tibet (E)	2303a	35.75	90.65	70.67	0.51	5.42	3.28	418	113	6.75	48.49	0.58	3.70	16.75	83.46
Wang et al., (2016)[19]	Northen Tibet (E)	2011	35.83	90.48	75.50	0.24	4.13	3.13	454	31	5.50	11.53	0.39	14.74	5.60	29.48
Wang et al., (2016)[19]	Northen Tibet (E)	1P2JD7-1	35.79	90.42	72.64	0.25	3.95	4.17	923	7	4.02	10.04	0.32	136.73	1.68	30.99
Wang et al., (2016)[19]	Northen Tibet (E)	1P2JD7-1a	35.79	90.42	72.43	0.02	4.14	4.06	810	9	4.79	11.48	0.35	87.16	1.94	33.08
Wang et al., (2016)[19]	Northen Tibet (E)	2509	35.97	90.80	73.13	0.26	5.08	3.21	586	29	7.01	27.61	0.51	20.44	4.09	54.56
Wang et al., (2016)[19]	Northen Tibet (E)	2059a	35.97	90.80	71.86	0.24	5.03	3.26	545	43	6.83	31.90	0.46	12.76	6.25	68.90
Wang et al., (2016)[19]	Northen Tibet (E)	2511-1	35.95	90.78	69.24	0.57	5.47	3.04	476	72	9.84	79.78	0.62	6.65	7.27	128.16

Source	Location	Sample	Latitude	Longitude	SiO2(wt.%)	MgO(wt.%)	K2O(wt.%)	Na2O(wt.%)	Rb(ppm)	Sr(ppm)	Y(ppm)	La(ppm)	Yb(ppm)	Rb/Sr	Sr/Y	La/Yb
Wang et al., (2016)[19]	Northen Tibet (M)	MV1B	36.46	87.48	71.02	0.19	4.90	2.58	458	54	25.00	22.10	0.49	8.56	2.14	45.10
Wang et al., (2016)[19]	Northen Tibet (M)	UM1B	36.46	87.48	72.08	0.15	4.90	3.03	448	55	25.00	21.90	0.50	8.19	2.19	43.80
Wang et al., (2016)[19]	Northen Tibet (M)	MV2	36.46	87.48	73.42	0.10	4.92	2.81	479	51	26.00	22.50	0.48	9.39	1.96	46.88
Wang et al., (2016)[19]	Northen Tibet (M)	UM3V	36.46	87.48	71.72	0.12	4.86	2.71	468	57	27.00	24.00	0.53	8.28	2.09	45.28
Wang et al., (2016)[19]	Northen Tibet (M)	UMVU	36.46	87.48	72.92	0.14	4.95	2.75	510	54	27.00	22.80	0.50	9.43	2.00	45.60
Wang et al., (2016)[19]	Northen Tibet (M)	UMOP	36.46	87.48	72.05	0.08	8.96	0.86	838	57	44.00	19.60	0.35	14.75	1.29	56.00
Wang et al., (2016)[19]	Northen Tibet (M)	OID	36.46	87.48	74.43	0.15	4.63	5.01	555	64	26.00	6.96	0.43	8.63	2.47	16.19
Wang et al., (2016)[19]	Northen Tibet (M)	BKSP	36.46	87.48	72.62	0.03	5.27	2.00	453	49	24.00	20.20	0.44	9.17	2.06	45.91
Wang et al., (2016)[19]	Northen Tibet (M)	UBTG	36.46	87.48	74.67	0.10	4.55	3.20	463	55	26.00	18.40	0.43	8.37	2.13	42.79
Wang et al., (2016)[19]	Northen Tibet (M)	KSPO	36.46	87.48	74.84	0.16	2.55	4.76	370	54	7.00	14.10	0.37	6.85	7.71	38.11
Wang et al., (2016)[19]	Northen Tibet (M)	169	36.22	86.40	75.34	0.18	4.47	3.55	326	15	34.82	16.56	1.76	21.73	0.43	9.41
Wang et al., (2016)[19]	Northen Tibet (M)	10-1	36.69	86.07	60.62	2.20	4.01	2.92	159	821	20.64	154.30	1.32	0.19	39.78	116.89
Wang et al., (2016)[19]	Northen Tibet (M)	43-1	36.68	86.08	60.82	1.95	4.12	3.03	136	856	22.49	162.30	1.40	0.16	38.06	115.93
Wang et al., (2016)[19]	Northen Tibet (M)	46-2	36.65	86.08	60.17	2.10	4.05	3.05	166	882	23.63	179.10	1.45	0.19	37.33	123.52
Wang et al., (2016)[19]	Northen Tibet (M)	40	36.69	86.04	60.54	2.02	4.16	2.99	170	890	23.02	178.80	1.43	0.19	38.66	125.03
Wang et al., (2016)[19]	Northen Tibet (M)	7	36.69	86.10	57.88	2.06	3.70	3.09	152	975	21.98	169.40	1.31	0.16	44.36	129.31
Wang et al., (2016)[19]	Northen Tibet (M)	2	36.71	86.05	60.56	1.99	4.15	3.12	174	874	22.70	172.10	1.40	0.20	38.50	122.93
Wang et al., (2016)[19]	Northen Tibet (M)	not given	36.22	86.40	71.86	0.21	3.77	4.63	1283	10	1.32	4.40	0.12	128.30	7.58	36.67
Wang et al., (2016)[19]	Northen Tibet (M)	not given	36.92	85.95	61.76	1.98	4.27	3.53	257	864	14.06	58.96	1.03	0.30	61.45	57.24
Wang et al., (2016)[19]	Northen Tibet (M)	not given	36.92	85.95	61.01	2.67	4.32	3.41	276	812	15.30	57.72	1.09	0.34	53.07	52.95

Note: W - west part; M - middle part; E - east part.

Table S2. Data used to calculate Sr/Y for global young rock data subsets.

Source	Location	Sample	Latitude	Longitude	SiO2(wt.%)	MgO(wt.%)	K2O(wt.%)	Na2O(wt.%)	Rb(ppm)	Sr(ppm)	Y(ppm)	La(ppm)	Yb(ppm)	Sr/Y	Rb/Sr	La/Yb
GEOROC[1]	North Eastern Anatolia (W)	MK261	40.06	41.36	64.30	2.21	2.74	3.89	85	395	18.00	26.00		21.94	0.22	
GEOROC[1]	North Eastern Anatolia (W)	MK228	40.06	41.36	63.40	2.39	2.74	3.83	60	461	12.00	30.00		38.42	0.13	
GEOROC[1]	North Eastern Anatolia (W)	MK251	40.06	41.36	65.10	2.17	2.35	4.21	59	454	14.00	33.10	1.04	32.43	0.13	31.83
GEOROC[1]	North Eastern Anatolia (W)	MK36	40.06	41.36	63.80	2.07	2.51	4.00	72	443	14.00	28.00		31.64	0.16	
GEOROC[1]	North Eastern Anatolia (W)	MK49	40.06	41.36	66.30	1.89	2.85	3.88	83	318	16.00	33.90	1.36	19.88	0.26	24.93
GEOROC[1]	North Eastern Anatolia (W)	MK52A	40.06	41.36	63.80	2.29	2.70	3.80	71	434	13.00	39.00		33.38	0.16	
GEOROC[1]	North Eastern Anatolia (W)	MK93	40.06	41.36	63.50	2.43	2.76	3.58	72	443	15.00	38.50	1.35	29.53	0.16	28.52
GEOROC[1]	North Eastern Anatolia (W)	MK265	40.12	41.58	63.90	3.65	2.73	3.53	70	453	16.00	31.40	1.14	28.31	0.15	27.54
GEOROC[1]	North Eastern Anatolia (W)	MK277	40.12	41.58	60.60	4.08	2.18	4.11	62	489	16.00	28.10	1.33	30.56	0.13	21.13
GEOROC[1]	North Eastern Anatolia (W)	MK268	40.12	41.58	65.50	2.03	3.27	3.92	91	350	15.00	29.20	1.37	23.33	0.26	21.31
GEOROC[1]	North Eastern Anatolia (W)	MK281	40.12	41.58	57.00	2.23	2.00	4.68	52	417	31.00	24.70	2.67	13.45	0.12	9.25
GEOROC[1]	North Eastern Anatolia (W)	MK101	39.98	41.68	57.00	4.56	2.00	3.48	54	434	21.00	30.20	2.22	20.67	0.12	13.60
GEOROC[1]	North Eastern Anatolia (W)	MK112	39.98	41.68	66.80	1.24	3.26	3.68	78	350	18.00	31.60	1.55	19.44	0.22	20.39
GEOROC[1]	North Eastern Anatolia (W)	MK118	39.98	41.68	66.40	1.24	2.58	3.95	63	413	11.00	19.00		37.55	0.15	
GEOROC[1]	North Eastern Anatolia (W)	MK337	39.98	41.68	66.60	1.23	3.35	3.57	76	367	17.00	21.00		21.59	0.21	
GEOROC[1]	North Eastern Anatolia (W)	MK117	39.98	41.68	62.10	1.99	2.34	4.03	59	369	21.00	20.00	2.00	17.57	0.16	10.00
GEOROC[1]	North Eastern Anatolia (W)	MK106	39.98	41.68	67.50	0.70	4.30	5.01	111	175	33.00	45.00		5.30	0.63	
GEOROC[1]	North Eastern Anatolia (W)	MK318	39.98	41.68	65.10	1.13	4.02	4.94	100	244	37.00	49.00		6.59	0.41	
GEOROC[1]	North Eastern Anatolia (W)	2	39.07	41.37	56.90	2.88	1.75	4.53	46	425	34.20	28.00	2.50	12.43	0.11	11.20
GEOROC[1]	North Eastern Anatolia (W)	1	39.07	41.37	56.35	3.36	1.95	4.40	54	492	31.60	44.00	3.00	15.57	0.11	14.67
GEOROC[1]	North Eastern Anatolia (M)	MK131	40.05	42.17	56.30	3.86	1.47	3.91	27	399	27.00	16.00		14.78	0.07	
GEOROC[1]	North Eastern Anatolia (M)	MK134	40.05	42.17	64.00	2.12	2.45	3.86	61	308	21.00	22.00		14.67	0.20	
GEOROC[1]	North Eastern Anatolia (M)	YOZ 92	38.62	42.19	60.31	1.28	3.40	5.00	101	329	49.40	47.20	4.86	6.67	0.31	9.71
GEOROC[1]	North Eastern Anatolia (M)	N-136	38.67	42.20	59.82	0.91	4.35	6.53	135	211	54.93	51.99	6.37	3.84	0.64	8.17
GEOROC[1]	North Eastern Anatolia (M)	N-036	38.67	42.20	60.25	0.96	3.72	4.81	109	180	58.76	50.36	6.23	3.06	0.61	8.08
GEOROC[1]	North Eastern Anatolia (M)	N-037	38.67	42.20	61.55	0.56	4.53	6.05	94	138	33.77	34.33	3.80	4.08	0.68	9.05
GEOROC[1]	North Eastern Anatolia (M)	N-013	38.67	42.20	56.44	3.97	2.17	4.52	84	244	60.62	37.19	6.39	4.03	0.34	5.82
GEOROC[1]	North Eastern Anatolia (M)	N-050	38.67	42.20	57.12	2.26	2.02	5.30	44	337	41.16	28.01	3.96	8.20	0.13	7.08
GEOROC[1]	North Eastern Anatolia (M)	N-256	38.67	42.20	59.51	3.45	2.44	4.53	100	203	67.06	42.35	7.08	3.02	0.49	5.98
GEOROC[1]	North Eastern Anatolia (M)	N-272	38.67	42.20	60.63	0.63	3.70	5.77	53	186	34.70	26.95	3.57	5.37	0.29	7.55
GEOROC[1]	North Eastern Anatolia (M)	N-014	38.67	42.20	67.35	1.62	3.56	4.86	158	100	90.73	61.08	9.57	1.11	1.57	6.38
GEOROC[1]	North Eastern Anatolia (M)	NR-14	38.69	42.22	64.23	2.44	3.10	4.92	123	164	70.40	49.50	8.80	2.33	0.75	5.63
GEOROC[1]	North Eastern Anatolia (M)	NR-11	38.68	42.23	56.33	4.61	1.95	4.31	71	269	52.10	34.10	6.40	5.16	0.27	5.33
GEOROC[1]	North Eastern Anatolia (M)	NR-24	38.56	42.24	61.40	0.99	3.56	5.22	98	217	47.00	44.90	5.60	4.62	0.45	8.02
GEOROC[1]	North Eastern Anatolia (M)	2009-07-16-03	38.56	42.25	61.68	0.81	3.74	5.26	105	205	49.00	41.00		4.18	0.51	
GEOROC[1]	North Eastern Anatolia (M)	CU-1	38.67	42.27	61.16	0.73	3.59	5.28	95	236	54.40	42.00	5.50	4.33	0.40	7.64
GEOROC[1]	North Eastern Anatolia (M)	Z-12	38.55	42.35	58.58	1.72	3.75	5.40	91	340	55.10	48.10	5.94	6.17	0.27	8.10
GEOROC[1]	North Eastern Anatolia (M)	MK156	40.18	42.82	62.80	2.64	2.42	3.74	67	73	27.00	23.00		2.70	0.92	
GEOROC[1]	North Eastern Anatolia (M)	MK145	40.18	42.82	60.30	3.95	2.03	3.56	59	44	19.00	19.00		2.32	1.34	
GEOROC[1]	North Eastern Anatolia (M)	MK147	40.18	42.82	62.40	3.43	2.40	3.46	64	270	26.00	24.00		10.38	0.24	
GEOROC[1]	North Eastern Anatolia (M)	MK148	40.18	42.82	61.90	2.94	2.54	3.62	66	285	27.00	27.00		10.56	0.23	
GEOROC[1]	North Eastern Anatolia (M)	MK149	40.18	42.82	62.80	3.38	2.49	3.47	65	268	24.00	21.00		11.17	0.24	

Source	Location	Sample	Latitude	Longitude	SiO2(wt.%)	MgO(wt.%)	K2O(wt.%)	Na2O(wt.%)	Rb(ppm)	Sr(ppm)	Y(ppm)	La(ppm)	Yb(ppm)	Sr/Y	Rb/Sr	La/Yb
GEOROC[1]	North Eastern Anatolia (M)	MK159	40.18	42.82	60.30	1.83	2.05	4.12	55	286	32.00	23.60	2.97	8.94	0.19	7.95
GEOROC[1]	North Eastern Anatolia (M)	MK160	40.18	42.82	61.80	2.86	2.43	3.87	62	292	33.00	28.00		8.85	0.21	
GEOROC[1]	North Eastern Anatolia (M)	MK165	40.18	42.82	63.50	2.50	2.71	3.64	68	265	24.00	23.00		11.04	0.26	
GEOROC[1]	North Eastern Anatolia (M)	MK166A	40.18	42.82	63.60	2.69	2.49	3.55	69	267	25.00	24.00		10.68	0.26	
GEOROC[1]	North Eastern Anatolia (M)	MK166B	40.18	42.82	63.50	2.70	2.46	3.60	70	268	25.00	21.00		10.72	0.26	
GEOROC[1]	North Eastern Anatolia (M)	MK168	40.18	42.82	60.50	3.39	1.77	3.95	46	313	26.00	17.00		12.04	0.15	
GEOROC[1]	North Eastern Anatolia (M)	MK158	40.18	42.82	61.70	1.41	2.79	4.52	71	368	35.00	34.20	3.41	10.51	0.19	10.03
GEOROC[1]	North Eastern Anatolia (M)	MK162	40.16	43.14	55.30	4.79	1.26	3.47	30	420	23.00	20.20	1.90	18.26	0.07	10.63
GEOROC[1]	North Eastern Anatolia (E)	AR01	39.75	44.40	63.83	1.64	1.85	5.03	41	389	23.00	26.00	2.06	16.91	0.11	12.62
GEOROC[1]	North Eastern Anatolia (E)	AR02	39.75	44.40	62.64	2.33	2.15	4.92	56	381	26.00	27.50	2.48	14.65	0.15	11.09
GEOROC[1]	North Eastern Anatolia (E)	AR11	39.75	44.40	58.73	3.36	1.81	4.41	37	446	33.00	24.40	2.91	13.52	0.08	8.38
GEOROC[1]	North Eastern Anatolia (E)	109	39.75	44.40	61.50	2.00	1.90	4.60	58	485	31.00			15.65	0.12	
GEOROC[1]	North Eastern Anatolia (E)	112	39.75	44.40	61.80	2.00	1.90	4.70	60	492	27.00			18.22	0.12	
GEOROC[1]	North Eastern Anatolia (E)	108A	39.75	44.40	62.50	1.60	1.80	4.80	53	453	25.00			18.12	0.12	
GEOROC[1]	North Eastern Anatolia (E)	93	39.75	44.40	62.70	1.70	2.00	4.70	62	473	25.00			18.92	0.13	
GEOROC[1]	North Eastern Anatolia (E)	108B	39.75	44.40	62.80	1.50	1.80	4.90	55	447	24.00			18.63	0.12	
GEOROC[1]	North Eastern Anatolia (E)	90	39.75	44.40	63.10	1.80	2.10	4.60	66	463	26.00			17.81	0.14	
GEOROC[1]	North Eastern Anatolia (E)	118	39.75	44.40	63.10	1.80	1.70	4.80	49	469	24.00			19.54	0.10	
GEOROC[1]	North Eastern Anatolia (E)	94	39.75	44.40	66.60	1.10	2.10	4.40	69	406	23.00			17.65	0.17	
GEOROC[1]	North Eastern Anatolia (E)	88	39.75	44.40	66.70	1.10	2.20	4.40	62	423	25.00			16.92	0.15	
GEOROC[1]	North Eastern Anatolia (E)	89	39.75	44.40	67.20	1.20	2.20	4.60	63	416	23.00			18.09	0.15	
GEOROC[1]	North Eastern Anatolia (E)	95	39.75	44.40	68.30	1.10	2.10	4.40	67	312	20.00			15.60	0.21	
GEOROC[1]	North Eastern Anatolia (E)	101	39.75	44.40	69.30	0.70	2.20	4.70	68	323	21.00			15.38	0.21	
GEOROC[1]	North Eastern Anatolia (E)	100	39.75	44.40	69.60	0.70	2.30	4.60	72	311	22.00			14.14	0.23	
GEOROC[1]	North Eastern Anatolia (E)	102	39.75	44.40	70.70	0.60	2.60	4.60	74	307	22.00			13.95	0.24	
GEOROC[1]	North Eastern Anatolia (E)	MU 8.13	39.75	44.40	55.43	5.93	1.12	4.27	28	376	23.00	17.00		16.35	0.07	
GEOROC[1]	Northeastern Central Anatolia	KT4	39.60	39.50	62.80	2.22	3.11	3.91	95	294	19.20	37.20	1.52	15.31	0.32	24.47
GEOROC[1]	Northeastern Central Anatolia	YT2	39.60	39.50	62.69	2.44	3.23	3.83	83	301	18.20	35.00	1.78	16.53	0.28	19.66
GEOROC[1]	Northeastern Central Anatolia	IR3	39.60	39.50	62.90	2.34	2.88	3.96	91	282	18.10	35.30	1.40	15.55	0.32	25.21
GEOROC[1]	Northeastern Central Anatolia	IR5	39.60	39.50	62.79	2.35	3.09	3.95	80	273	17.40	32.90	1.61	15.70	0.29	20.43
GEOROC[1]	Northeastern Central Anatolia	IR8	39.60	39.50	62.46	2.41	3.09	4.02	83	281	18.90	33.50	1.68	14.85	0.30	19.94
GEOROC[1]	Northeastern Central Anatolia	IR9	39.60	39.50	62.96	2.30	3.09	4.02	83	288	17.80	34.60	1.69	16.16	0.29	20.47
GEOROC[1]	Northeastern Central Anatolia	IR10	39.60	39.50	62.81	2.33	3.01	3.95	84	288	17.80	33.70	1.90	16.17	0.29	17.74
GEOROC[1]	Northeastern Central Anatolia	IR4A	39.60	39.50	63.70	2.26	3.24	3.84	89	274	17.00	34.60	1.60	16.14	0.32	21.63
GEOROC[1]	Northeastern Central Anatolia	IR7	39.60	39.50	63.60	2.41	3.04	4.00	85	294	18.50	35.20	1.90	15.90	0.29	18.53
GEOROC[1]	Northeastern Central Anatolia	IR11	39.60	39.50	63.20	2.32	3.00	4.01	81	281	17.90	34.00	1.74	15.68	0.29	19.54
GEOROC[1]	Northeastern Central Anatolia	YT3	39.60	39.50	63.90	1.87	3.20	4.11	89	276	18.00	36.70	1.82	15.33	0.32	20.16
GEOROC[1]	Southwestern Central Anatolia	KO-92-10	38.17	32.50	67.66	1.36	3.83	3.11	157	460	20.40			22.54	0.34	
GEOROC[1]	Southwestern Central Anatolia	KO-92-13	38.17	32.50	66.47	1.44	3.32	3.87	97	828	18.10			45.74	0.12	
GEOROC[1]	Southwestern Central Anatolia	KO-94-6	38.17	32.50	66.70	1.45	3.65	3.20	146	434	20.10			21.61	0.34	
GEOROC[1]	Southwestern Central Anatolia	KO-94-7	38.17	32.50	63.55	1.66	3.27	3.23	122	557	25.60			21.75	0.22	

Source	Location	Sample	Latitude	Longitude	SiO2(wt.%)	MgO(wt.%)	K2O(wt.%)	Na2O(wt.%)	Rb(ppm)	Sr(ppm)	Y(ppm)	La(ppm)	Yb(ppm)	Sr/Y	Rb/Sr	La/Yb
GEOROC[1]	Southwestern Central Anatolia	KO-94-10	38.17	32.50	65.16	1.03	3.21	4.08	101	916	17.30			52.97	0.11	
GEOROC[1]	Southwestern Central Anatolia	KO94-11	38.17	32.50	58.04	2.85	2.59	3.40	91	629	24.90			25.28	0.14	
GEOROC[1]	Southwestern Central Anatolia	KO94-21	38.17	32.50	62.44	2.29	3.11	3.28	119	472	29.10			16.20	0.25	
GEOROC[1]	Southwestern Central Anatolia	KO-94-24	38.17	32.50	62.48	2.24	2.85	3.99	81	1041	20.80			50.04	0.08	
GEOROC[1]	Southwestern Central Anatolia	KO-92-4	38.17	32.50	66.48	1.64	3.70	3.66	108	788	18.60			42.38	0.14	
GEOROC[1]	Southwestern Central Anatolia	KO-92-5	38.17	32.50	56.97	5.66	3.36	3.29	86	593	24.30			24.39	0.15	
GEOROC[1]	Southwestern Central Anatolia	KO-92-6	38.17	32.50	59.96	2.32	2.59	3.79	82	635	26.20			24.25	0.13	
GEOROC[1]	Southwestern Central Anatolia	KO-92-7	38.17	32.50	57.80	3.22	2.51	3.51	73	679	26.10			26.01	0.11	
GEOROC[1]	Southwestern Central Anatolia	KO-92-18	38.17	32.50	58.36	3.66	1.64	3.60	45	580	23.50			24.67	0.08	
GEOROC[1]	Southwestern Central Anatolia	KO-92-19	38.17	32.50	60.41	3.37	2.52	3.55	77	887	19.00			46.66	0.09	
GEOROC[1]	Southwestern Central Anatolia	KO-92-128	38.17	32.50	59.17	2.96	2.48	3.44	77	906	20.00			45.31	0.08	
GEOROC[1]	Southwestern Central Anatolia	KO-93-129	38.17	32.50	59.02	2.59	2.07	3.15	79	629	25.70			24.47	0.12	
GEOROC[1]	Southwestern Central Anatolia	KO-93-136	38.17	32.50	63.47	1.84	3.17	3.68	114	630	21.10			29.85	0.18	
GEOROC[1]	Southwestern Central Anatolia	KO-94-14	38.17	32.50	57.68	3.68	2.74	3.17	88	1047	23.00			45.53	0.08	
GEOROC[1]	Southwestern Central Anatolia	KO-94-15	38.17	32.50	59.39	2.49	3.07	3.61	92	1334	22.30			59.83	0.07	
GEOROC[1]	Southwestern Central Anatolia	KO-94-16	38.17	32.50	60.49	2.47	3.06	3.64	98	1149	21.20			54.17	0.09	
GEOROC[1]	Southwestern Central Anatolia	KO-94-17	38.17	32.50	56.73	3.76	2.08	3.42	49	957	19.20			49.86	0.05	
GEOROC[1]	Southwestern Central Anatolia	KO-94-18	38.17	32.50	62.24	1.98	3.41	3.34	121	829	22.30			37.17	0.15	
GEOROC[1]	Southwestern Central Anatolia	KO-94-53	38.17	32.50	58.00	3.40	2.48	3.31	76	934	20.70			45.11	0.08	
GEOROC[1]	Southwestern Central Anatolia	KO-92-1	38.17	32.50	62.12	2.32	2.70	3.90	2	1294	22.30			58.02	0.00	
GEOROC[1]	Southwestern Central Anatolia	KO-92-15	38.17	32.50	68.41	1.45	3.68	3.53	90	634	12.70			49.95	0.14	
GEOROC[1]	Southwestern Central Anatolia	KO-92-16	38.17	32.50	62.76	3.01	3.05	4.14	78	1057	19.40			54.48	0.07	
GEOROC[1]	Southwestern Central Anatolia	KO-92-17	38.17	32.50	61.68	2.80	2.55	4.05	72	701	21.50			32.59	0.10	
GEOROC[1]	Southwestern Central Anatolia	KO-94-29	38.17	32.50	66.96	1.21	3.27	3.55	115	840	17.40			48.28	0.14	
GEOROC[1]	Southwestern Central Anatolia	KO-94-36	38.17	32.50	63.46	1.89	2.79	3.74	98	602	21.60			27.85	0.16	
GEOROC[1]	Southwestern Central Anatolia	KO-94-37	38.17	32.50	66.06	1.22	3.01	3.12	108	521	19.80			26.29	0.21	
GEOROC[1]	South Central Anatolia (W)	88-24	38.13	34.18	62.33	2.74	1.87	3.97	62	364	20.00			18.20	0.17	
GEOROC[1]	South Central Anatolia (W)	90-62	38.13	34.18	67.19	1.18	2.94	3.86	96	243	18.00	30.50	1.43	13.50	0.40	21.33
GEOROC[1]	South Central Anatolia (W)	90-36	38.13	34.18	64.04	2.75	1.97	3.83	66	350	21.00	31.30	2.03	16.67	0.19	15.42
GEOROC[1]	South Central Anatolia (W)	90-12	38.13	34.18	71.60	0.65	3.18	4.20	104	199	16.00			12.44	0.52	
GEOROC[1]	South Central Anatolia (W)	90-64	38.13	34.18	71.78	0.63	3.30	4.04	91	264	17.00			15.53	0.34	
GEOROC[1]	South Central Anatolia (W)	90-40	38.13	34.18	64.86	2.29	2.24	3.79	70	332	20.00			16.60	0.21	
GEOROC[1]	South Central Anatolia (W)	90-45	38.13	34.18	64.68	2.13	2.22	3.54	74	312	19.00			16.42	0.24	
GEOROC[1]	South Central Anatolia (W)	90-52	38.13	34.18	63.79	2.74	1.87	3.92	66	369	19.00			19.42	0.18	
GEOROC[1]	South Central Anatolia (W)	89-38	38.13	34.18	67.67	1.21	3.03	4.18	98	258	16.00			16.13	0.38	
GEOROC[1]	South Central Anatolia (W)	89-24	38.13	34.18	66.00	2.13	2.37	4.30	79	332	19.00			17.47	0.24	
GEOROC[1]	South Central Anatolia (W)	K102P	38.12	34.28	67.80	1.13	3.01	4.03	107	270	14.40	28.60	1.43	18.75	0.40	20.00
GEOROC[1]	South Central Anatolia (W)	K-84	38.11	34.29	71.80	0.66	3.17	4.40	93	229	14.10	29.30	1.42	16.24	0.41	20.63
GEOROC[1]	South Central Anatolia (W)	K110	38.11	34.30	70.70	0.64	3.26	4.12	107	221	13.90	28.20	1.51	15.90	0.48	18.68
GEOROC[1]	South Central Anatolia (W)	KEC-2	38.18	34.42	69.50	0.77	3.45	3.77	112	217	13.70	24.20	1.42	15.84	0.52	17.04
GEOROC[1]	South Central Anatolia (W)	C94-31	38.42	34.63	57.64	3.51	1.72	3.25	33	325	16.83	24.12	1.93	19.28	0.10	12.50
GEOROC[1]	South Central Anatolia (E)	ERC96-195	38.53	35.45	62.10	2.73	2.14	3.88	77	334	29.70			11.26	0.23	

Source	Location	Sample	Latitude	Longitude	SiO2(wt.%)	MgO(wt.%)	K2O(wt.%)	Na2O(wt.%)	Rb(ppm)	Sr(ppm)	Y(ppm)	La(ppm)	Yb(ppm)	Sr/Y	Rb/Sr	La/Yb
GEOROC[1]	South Central Anatolia (E)	ERC95-92	38.53	35.45	63.98	2.52	2.03	3.71	62	307	22.00			13.97	0.20	
GEOROC[1]	South Central Anatolia (E)	ERC96-129	38.53	35.45	62.99	2.61	2.22	3.90	70	308	27.10			11.35	0.23	
GEOROC[1]	South Central Anatolia (E)	ERC91-29	38.53	35.45	57.11	3.66	1.34	4.21	30	422	29.00	27.90	2.58	14.54	0.07	10.81
GEOROC[1]	South Central Anatolia (E)	ERC95-99	38.53	35.45	67.03	1.92	2.69	3.83	96	242	22.10			10.97	0.39	
GEOROC[1]	South Central Anatolia (E)	ERC95-100	38.53	35.45	69.92	1.14	3.04	3.81	116	213	22.70			9.37	0.55	
GEOROC[1]	South Central Anatolia (E)	ERC91-9	38.53	35.45	65.71	2.15	1.99	3.91	78	275	26.10			10.55	0.28	
GEOROC[1]	South Central Anatolia (E)	ERC95-83	38.53	35.45	67.06	1.69	2.27	3.68	83	258	23.80	20.30	1.84	10.82	0.32	11.03
GEOROC[1]	South Central Anatolia (E)	ERC95-90	38.53	35.45	70.55	0.79	2.83	3.92	113	201	22.00			9.13	0.56	
GEOROC[1]	South Central Anatolia (E)	ERC96-109	38.53	35.45	62.07	3.07	1.37	3.37	37	335	21.00			15.97	0.11	
GEOROC[1]	South Central Anatolia (E)	ERC96-112	38.53	35.45	59.44	2.70	1.73	3.78	27	376	30.80			12.22	0.07	
GEOROC[1]	South Central Anatolia (E)	ERC96-113	38.53	35.45	62.04	2.11	2.04	4.39	73	343	33.80			10.16	0.21	
GEOROC[1]	South Central Anatolia (E)	C88-89	38.53	35.45	71.24	0.93	2.85	4.10	111	202	19.80			10.19	0.55	
GEOROC[1]	South Central Anatolia (E)	ERC91-21	38.53	35.45	65.84	1.98	2.09	3.95	85	295	27.30	25.90	2.10	10.81	0.29	12.33
GEOROC[1]	South Central Anatolia (E)	ERC91-28	38.53	35.45	70.50	0.96	2.79	4.00	134	210	25.00			8.40	0.64	
GEOROC[1]	South Central Anatolia (E)	ERC96-186	38.53	35.45	65.26	2.01	2.27	3.46	87	264	24.40			10.83	0.33	
GEOROC[1]	South Central Anatolia (E)	A2	38.53	35.45	60.48	3.08	1.89	3.87	51	348	25.30	25.10	2.54	13.77	0.15	9.88
GEOROC[1]	South Central Anatolia (E)	A4	38.53	35.45	61.42	3.30	1.83	3.71	54	327	22.70	24.20	2.32	14.42	0.16	10.43
GEOROC[1]	South Central Anatolia (E)	CM2G	38.53	35.45	64.35	2.40	2.22	3.86	77	321	23.50	29.30	2.35	13.68	0.24	12.47
GEOROC[1]	South Central Anatolia (E)	CM8	38.53	35.45	64.91	2.29	2.26	3.91	81	308	22.70	28.50	2.21	13.55	0.26	12.90
GEOROC[1]	South Central Anatolia (E)	CM1E	38.53	35.45	64.75	2.41	2.24	3.90	77	298	22.60	26.70	2.07	13.17	0.26	12.90
GEOROC[1]	South Central Anatolia (E)	CM5	38.53	35.45	61.92	2.60	1.99	3.82	67	329	25.10	27.30	2.27	13.11	0.20	12.03
GEOROC[1]	South Central Anatolia (E)	CM5K	38.53	35.45	63.61	2.42	2.13	3.82	76	304	22.70	26.80	2.27	13.40	0.25	11.81
GEOROC[1]	South Central Anatolia (E)	ERC95-128	38.53	35.45	61.02	3.03	1.85	3.72	61	323	26.90			12.02	0.19	
GEOROC[1]	South Central Anatolia (E)	ERC95-9	38.53	35.45	63.34	2.19	2.37	4.10	80	286	27.70			10.32	0.28	
GEOROC[1]	South Central Anatolia (E)	ERC96-71	38.53	35.45	64.72	2.02	2.03	4.54	69	265	20.40	26.20	2.21	12.97	0.26	11.86
GEOROC[1]	South Central Anatolia (E)	ERC91-27	38.53	35.45	68.87	1.28	2.82	3.85	114	210	25.60			8.21	0.54	
GEOROC[1]	South Central Anatolia (E)	ERC95-111	38.53	35.45	65.84	1.60	2.26	3.60	91	249	24.30			10.24	0.36	
GEOROC[1]	South Central Anatolia (E)	ERC96-55	38.53	35.45	63.51	2.04	1.79	3.55	65	281	22.10			12.71	0.23	
GEOROC[1]	South Central Anatolia (E)	ERC95-14	38.53	35.45	62.25	1.55	3.01	4.20	88	298	36.40			8.19	0.30	
GEOROC[1]	South Central Anatolia (E)	ERC95-75	38.53	35.45	63.72	1.23	2.52	4.60	87	297	38.70			7.67	0.29	
GEOROC[1]	South Central Anatolia (E)	ERC91-1	38.53	35.45	63.24	2.78	1.60	3.80	55	315	25.20			12.50	0.18	
GEOROC[1]	South Central Anatolia (E)	ERC92-1	38.53	35.45	65.06	2.21	2.15	3.78	92	275	24.30			11.33	0.33	
GEOROC[1]	South Central Anatolia (E)	ERC92-3	38.53	35.45	70.10	0.92	2.96	3.90	147	195	23.80	28.50	2.10	8.17	0.75	13.57
GEOROC[1]	South Central Anatolia (E)	ER11	38.53	35.45	62.02	2.93	1.63	3.65	45	324	22.00	23.80	2.15	14.73	0.14	11.07
GEOROC[1]	South Central Anatolia (E)	ER13	38.53	35.45	66.44	1.40	2.99	4.54	106	229	27.00	30.10	2.70	8.48	0.46	11.15
GEOROC[1]	South Central Anatolia (E)	ER14	38.53	35.45	63.76	2.24	2.45	4.17	84	286	27.00	30.20	2.67	10.59	0.29	11.31
GEOROC[1]	South Central Anatolia (E)	ER15	38.53	35.45	68.97	1.15	2.79	3.96	106	217	21.00	27.40	2.14	10.33	0.49	12.80
GEOROC[1]	South Central Anatolia (E)	ER16	38.53	35.45	69.14	0.78	2.85	3.89	117	207	21.00	30.00	2.18	9.86	0.57	13.76
GEOROC[1]	South Central Anatolia (E)	ER17	38.53	35.45	64.24	2.08	2.36	3.77	81	290	27.00	36.10	2.51	10.74	0.28	14.38
GEOROC[1]	South Central Anatolia (E)	ER20	38.53	35.45	65.69	2.19	2.32	3.75	79	281	25.00	33.20	2.37	11.24	0.28	14.01
GEOROC[1]	South Central Anatolia (E)	ER21	38.53	35.45	63.18	2.62	1.81	3.84	64	318	23.00	24.90	2.23	13.83	0.20	11.17
GEOROC[1]	South Central Anatolia (E)	ER22	38.53	35.45	66.20	2.20	2.55	4.41	95	259	22.00			11.77	0.37	
GEOROC[1]	South Central Anatolia (E)	ER23	38.53	35.45	63.48	2.91	2.23	3.49	70	315	22.00	23.50	1.76	14.32	0.22	13.35
GEOROC[1]	South Central Anatolia (E)	ER25	38.53	35.45	64.33	2.43	1.78	3.87	48	303	21.00	23.20	1.95	14.43	0.16	11.90

Source	Location	Sample	Latitude	Longitude	SiO2(wt.%)	MgO(wt.%)	K2O(wt.%)	Na2O(wt.%)	Rb(ppm)	Sr(ppm)	Y(ppm)	La(ppm)	Yb(ppm)	Sr/Y	Rb/Sr	La/Yb
GEOROC[1]	South Central Anatolia (E)	ER26	38.53	35.45	57.90	3.90	1.50	3.96	26	394	27.00	27.80	2.71	14.59	0.07	10.26
GEOROC[1]	South Central Anatolia (E)	IEY-001	38.53	35.45	68.92	1.58	1.93	3.83	89	273	19.90	23.40	1.86	13.72	0.33	12.58
GEOROC[1]	South Central Anatolia (E)	IEY-003	38.53	35.45	67.93	1.75	2.22	3.79	98	269	23.40	26.90	2.26	11.50	0.36	11.90
GEOROC[1]	South Central Anatolia (E)	IEY-005	38.53	35.45	69.10	1.77	2.16	3.80	93	262	22.10	27.60	2.00	11.86	0.35	13.80
GEOROC[1]	South Central Anatolia (E)	IEY-0017	38.53	35.45	67.54	1.91	2.08	3.82	90	256	22.10	26.20	2.21	11.58	0.35	11.86
GEOROC[1]	South Central Anatolia (E)	IY-033	38.53	35.45	67.50	1.77	2.34	3.96	84	258	22.20	25.00	2.01	11.62	0.33	12.44
GEOROC[1]	South Central Anatolia (E)	IY-0317	38.53	35.45	67.50	1.82	2.42	3.82	83	256	22.80	25.00	1.99	11.23	0.32	12.56
GEOROC[1]	South Central Anatolia (E)	EY-0012	38.53	35.45	65.48	2.15	2.31	4.21	72	323	21.20	29.70	2.18	15.24	0.22	13.62
GEOROC[1]	South Central Anatolia (E)	EY-0016	38.53	35.45	65.96	2.43	2.56	3.81	87	248	22.00	28.40	2.31	11.27	0.35	12.29
GEOROC[1]	South Central Anatolia (E)	IEY-007	38.53	35.45	66.85	2.67	2.91	3.42	99	245	19.30	25.00	2.03	12.69	0.40	12.32
GEOROC[1]	South Central Anatolia (E)	IEY-009	38.53	35.45	69.60	1.65	2.94	3.74	89	264	22.40	28.40	2.23	11.79	0.34	12.74
GEOROC[1]	South Central Anatolia (E)	IEY-0013	38.53	35.45	66.53	2.05	2.22	3.74	86	269	23.50	26.70	2.32	11.45	0.32	11.51
GEOROC[1]	South Central Anatolia (E)	IEY-00X2	38.53	35.45	65.77	2.69	2.39	3.34	81	300	21.00	28.90	2.02	14.29	0.27	14.31
GEOROC[1]	South Central Anatolia (E)	IEY-00X4	38.53	35.45	65.23	2.71	1.99	3.31	71	316	21.00	30.00	2.01	15.05	0.22	14.93
GEOROC[1]	South Central Anatolia (E)	IEY-00X12	38.53	35.45	66.66	2.04	2.14	3.73	89	280	24.50	27.40	2.40	11.43	0.32	11.42
GEOROC[1]	South Central Anatolia (E)	IEY-00X14	38.53	35.45	66.70	2.23	1.97	3.68	81	280	23.20	26.30	2.21	12.07	0.29	11.90
GEOROC[1]	South Central Anatolia (E)	IEY-00X18	38.53	35.45	68.65	1.27	2.99	3.71	126	232	25.20	30.80	2.56	9.21	0.54	12.03
GEOROC[1]	South Central Anatolia (E)	IEY-00X20	38.53	35.45	68.85	1.47	2.74	3.68	85	257	20.40	27.90	2.03	12.60	0.33	13.74
GEOROC[1]	South Central Anatolia (E)	IEY-00X22	38.53	35.45	69.90	1.26	3.05	3.73	94	230	20.60	30.20	2.09	11.17	0.41	14.45
GEOROC[1]	South Central Anatolia (E)	IEY-00X24	38.53	35.45	70.00	1.50	2.48	3.48	86	229	20.00	28.50	2.06	11.45	0.38	13.83
GEOROC[1]	South Central Anatolia (E)	IEY-00X26	38.53	35.45	68.43	1.75	2.40	3.58	86	250	21.70	27.50	2.22	11.52	0.34	12.39
GEOROC[1]	South Central Anatolia (E)	IEY-00X30	38.53	35.45	69.12	1.50	2.92	3.67	99	254	22.20	29.50	2.22	11.44	0.39	13.29
GEOROC[1]	South Central Anatolia (E)	IEY-0011	38.53	35.45	71.11	1.28	3.11	3.92	97	233	20.90	30.10	1.95	11.15	0.42	15.44
GEOROC[1]	South Central Anatolia (E)	IEY-0015A	38.53	35.45	70.51	1.03	3.10	3.84	114	213	21.40	29.50	2.28	9.95	0.54	12.94
GEOROC[1]	South Central Anatolia (E)	IEY-0015B	38.53	35.45	70.62	1.02	3.11	3.85	116	225	21.70	30.80	2.11	10.37	0.52	14.60
GEOROC[1]	South Central Anatolia (E)	IEY-00X28	38.53	35.45	70.94	0.75	3.03	3.83	57	113	10.10	15.50	1.05	11.19	0.50	14.76
GEOROC[1]	South Central Anatolia (E)	OU-0319	38.53	35.45	67.82	2.10	1.82	3.89	56	265	20.20	23.70	1.93	13.12	0.21	12.28
GEOROC[1]	South Central Anatolia (E)	OU-0322-5B	38.53	35.45	65.28	2.46	1.82	3.90	57	300	22.40	25.60	2.03	13.39	0.19	12.61
GEOROC[1]	South Central Anatolia (E)	OU-0322-6	38.53	35.45	65.88	2.42	1.85	3.83	58	284	20.90	23.00	1.93	13.59	0.20	11.92
GEOROC[1]	South Central Anatolia (E)	OU-0322-9	38.53	35.45	65.46	2.17	2.54	3.82	79	295	20.80	29.60	2.18	14.18	0.27	13.58
GEOROC[1]	South Central Anatolia (E)	OU-0326-1	38.53	35.45	67.03	1.74	2.27	3.89	81	265	21.10	24.80	1.93	12.56	0.31	12.85
GEOROC[1]	South Central Anatolia (E)	OU-0328B	38.53	35.45	66.95	1.88	2.30	3.90	81	265	20.10	21.30	1.97	13.18	0.31	10.81
GEOROC[1]	South Central Anatolia (E)	E-103	38.55	35.54	62.19	2.04	2.94	3.77	98	300	32.20	29.41	2.70	9.33	0.33	10.89
GEOROC[1]	South Central Anatolia (E)	E-86	38.67	35.55	64.08	1.91	2.03	3.85	65	316	19.60	23.36	1.98	16.10	0.21	11.80
GEOROC[1]	South Central Anatolia (E)	E-88	38.66	35.56	62.19	2.01	1.98	3.74	69	295	20.20	22.63	1.99	14.62	0.24	11.37
GEOROC[1]	South Central Anatolia (E)	E-169	38.32	35.64	64.08	2.17	2.47	3.76	96	279	21.30	26.72	2.01	13.10	0.34	13.29
GEOROC[1]	South Central Anatolia (E)	E-141	38.34	35.65	61.39	2.71	2.07	4.06	71	318	19.20	20.97	1.93	16.55	0.22	10.87
GEOROC[1]	South Central Anatolia (E)	E-102-B	38.57	35.65	58.61	3.12	2.16	4.01	87	330	28.30	33.99	2.51	11.66	0.26	13.54
GEOROC[1]	South Central Anatolia (E)	ZD03-04	38.60	35.90	66.34	1.78	2.24	3.96	85	261	22.50	23.80	2.14	11.60	0.33	11.12
GEOROC[1]	South Central Anatolia (E)	ZD03-08A	38.60	35.90	66.44	1.76	2.34	3.93	86	254	22.00	24.10	2.11	11.55	0.34	11.42
GEOROC[1]	South Central Anatolia (E)	ZD03-08B	38.60	35.90	66.49	1.77	2.34	3.93	86	256	21.80	24.00	2.05	11.74	0.34	11.71
GEOROC[1]	South Central Anatolia (E)	ZD03-40	38.60	35.90	66.91	1.82	2.27	3.87	83	253	23.00	25.50	2.14	11.00	0.33	11.92
GEOROC[1]	South Central Anatolia (E)	ZD03-12	38.60	35.90	66.00	1.73	2.31	3.94	87	256	22.30	24.90	1.96	11.48	0.34	12.70
GEOROC[1]	South Central Anatolia (E)	ZD03-16	38.60	35.90	66.35	1.74	2.24	3.92	85	248	21.20	24.30	2.10	11.70	0.34	11.57

Source	Location	Sample	Latitude	Longitude	SiO2(wt.%)	MgO(wt.%)	K2O(wt.%)	Na2O(wt.%)	Rb(ppm)	Sr(ppm)	Y(ppm)	La(ppm)	Yb(ppm)	Sr/Y	Rb/Sr	La/Yb
GEOROC[1]	South Central Anatolia (E)	ZD03-26	38.60	35.90	66.73	1.87	2.27	3.91	86	258	21.80	24.60	1.92	11.83	0.33	12.81
GEOROC[1]	South Central Anatolia (E)	ZD03-34	38.60	35.90	66.60	1.72	2.29	3.93	88	260	22.20	25.60	2.04	11.71	0.34	12.55
GEOROC[1]	South Central Anatolia (E)	ZD03-11	38.60	35.90	65.20	1.99	2.21	3.76	79	258	21.90	24.50	2.11	11.78	0.31	11.61
GEOROC[1]	South Central Anatolia (E)	ZD03-15	38.60	35.90	66.03	2.05	2.20	3.86	77	254	21.50	22.90	2.16	11.81	0.30	10.60
GEOROC[1]	South Central Anatolia (E)	ZD03-19	38.60	35.90	65.92	2.03	2.18	3.82	84	263	22.60	26.60	2.28	11.64	0.32	11.67
GEOROC[1]	South Central Anatolia (E)	ZD-03-23	38.60	35.90	66.48	1.84	2.30	3.87	82	261	22.40	24.70	2.09	11.65	0.31	11.82
GEOROC[1]	South Central Anatolia (E)	ZD03-01	38.60	35.90	66.76	1.56	2.30	3.92	87	251	21.90	25.30	2.13	11.46	0.35	11.88
GEOROC[1]	South Central Anatolia (E)	ZD03-03	38.60	35.90	66.14	1.69	2.22	3.87	85	264	21.70	23.20	1.99	12.17	0.32	11.66
GEOROC[1]	South Central Anatolia (E)	ZD03-05	38.60	35.90	66.18	1.68	2.30	3.82	88	250	21.50	23.60	2.11	11.63	0.35	11.18
GEOROC[1]	Northwestern Iran	L-1	38.62	45.51	65.10	0.98	3.77	4.30	143	972	18.00	42.60	1.88	54.00	0.15	22.66
GEOROC[1]	Northwestern Iran	BA2	38.55	45.53	57.50	3.50	2.76	5.27	56	1030	16.80	64.90	1.51	61.31	0.05	42.98
GEOROC[1]	Northwestern Iran	DA14	38.56	45.54	62.40	2.21	2.87	5.31	69	1045	12.70	61.40	1.10	82.28	0.07	55.82
GEOROC[1]	Northern Iran	DMV12	35.95	52.11	60.68	2.72	4.42	4.75	125	1704	13.00	95.20	1.10	131.08	0.07	86.55
GEOROC[1]	Northern Iran	DMV13	35.95	52.11	62.60	2.15	4.61	4.56	163	1209	13.00	96.10	1.10	93.00	0.13	87.36
GEOROC[1]	Northern Iran	DMV19G	35.95	52.11	59.18	2.59	4.41	4.84	135	1492	11.00	79.00		135.64	0.09	
GEOROC[1]	Northern Iran	DMV19B	35.95	52.11	59.30	2.73	4.37	4.74	125	1632	12.00	100.40	1.00	136.00	0.08	100.40
GEOROC[1]	Northern Iran	DMV34	35.95	52.11	60.63	2.76	4.36	4.78	117	1406	13.00	89.40	1.30	108.15	0.08	68.77
GEOROC[1]	Northern Iran	DMV147	35.95	52.11	61.20	2.22	4.32	4.87	128	1220	14.00	80.00		87.14	0.10	
GEOROC[1]	Northern Iran	DMV07	35.95	52.11	60.24	2.53	4.39	4.82	126	1621	12.00	95.50	1.10	135.08	0.08	86.82
GEOROC[1]	Northern Iran	DMV116	35.95	52.11	62.04	2.69	4.43	4.73	141	1300	13.00	86.00		100.00	0.11	
GEOROC[1]	Northern Iran	DMV39	35.95	52.11	59.39	2.75	4.43	4.80	121	1430	11.00	92.30	1.00	130.00	0.08	92.30
GEOROC[1]	Northern Iran	DMV132A	35.95	52.11	61.32	2.61	4.39	4.79	130	1340	14.00	84.00		95.71	0.10	
GEOROC[1]	Northern Iran	DMV114	35.95	52.11	62.31	2.37	4.31	4.69	126	1210	15.00	68.00		80.67	0.10	
GEOROC[1]	Northern Iran	DMV108	35.95	52.11	60.13	3.02	4.26	4.71	117	1340	14.00	86.00		95.71	0.09	
GEOROC[1]	Northern Iran	DA5400	35.95	52.11	59.61	1.27	4.30	4.55	125	1340	12.40	84.20	0.77	108.06	0.09	109.35
GEOROC[1]	Northern Iran	DA P2	35.95	52.11	59.98	2.61	5.00	5.04	127	1580	12.80	98.00	1.06	123.44	0.08	92.45
GEOROC[1]	Northern Iran	DA4800	35.95	52.11	61.32	2.46	4.36	4.86	131	1370	12.77	91.60	1.04	107.28	0.10	88.08
GEOROC[1]	Eastern Carpathian	C14	47.28	25.22	63.52	2.54	2.40	3.11	154	323	27.80	24.00		11.62	0.48	
GEOROC[1]	Eastern Carpathian	C26	47.22	25.20	59.43	3.32	1.61	3.25	63	337	29.30	21.00		11.50	0.19	
GEOROC[1]	Eastern Carpathian	C18	47.13	25.42	55.83	3.85	0.99	3.11	32	264	26.10	11.00		10.11	0.12	
GEOROC[1]	Eastern Carpathian	C21	47.10	25.40	60.74	1.67	1.91	3.33	67	364	33.30	35.00		10.93	0.18	
GEOROC[1]	Eastern Carpathian	C13	47.10	25.22	63.57	3.89	2.24	3.65	80	366	16.70	26.00		21.92	0.22	
GEOROC[1]	Eastern Carpathian	C5	47.08	25.35	61.76	3.08	3.14	3.48	133	303	25.60	35.00		11.84	0.44	
GEOROC[1]	Eastern Carpathian	C7	47.05	25.27	62.35	1.89	1.21	3.80	50	289	21.20	14.00		13.63	0.17	
GEOROC[1]	Eastern Carpathian	C8	47.05	25.30	64.11	1.12	0.92	3.95	32	283	16.00	11.00		17.69	0.11	
GEOROC[1]	Eastern Carpathian	C2	47.05	25.35	66.36	0.82	1.00	4.03	26	290	13.40	14.00		21.64	0.09	
GEOROC[1]	Eastern Carpathian	C3	47.05	25.37	68.60	0.71	1.29	4.43	44	296	14.90	16.00		19.87	0.15	
GEOROC[1]	Eastern Carpathian	C10	46.97	25.18	58.03	2.57	1.81	3.37	72	250	30.50	20.00		8.20	0.29	
GEOROC[1]	Eastern Carpathian	G9	46.73	25.37	61.23	3.11	1.80	3.50	60	244	21.90	24.00		11.14	0.25	
GEOROC[1]	Eastern Carpathian	G7	46.67	25.32	57.86	3.38	1.33	3.55	40	301	25.10	22.00		11.99	0.13	
GEOROC[1]	Eastern Carpathian	G1	46.62	25.55	56.83	3.86	1.14	3.35	34	281	23.00	17.00		12.22	0.12	

Source	Location	Sample	Latitude	Longitude	SiO2(wt.%)	MgO(wt.%)	K2O(wt.%)	Na2O(wt.%)	Rb(ppm)	Sr(ppm)	Y(ppm)	La(ppm)	Yb(ppm)	Sr/Y	Rb/Sr	La/Yb
GEOROC[1]	Eastern Carpathian	G32	46.62	25.18	57.87	3.62	1.44	3.63	46	322	23.20	27.00		13.88	0.14	
GEOROC[1]	Eastern Carpathian	G3	46.62	25.43	59.38	3.19	1.38	3.49	41	290	22.30	17.00		13.00	0.14	
GEOROC[1]	Eastern Carpathian	G6	46.60	25.40	66.82	3.32	1.20	3.53	37	274	29.00	19.00		9.45	0.13	
GEOROC[1]	Eastern Carpathian	G39	46.58	25.48	58.78	3.01	1.57	3.53	50	316	21.30	22.00		14.84	0.16	
GEOROC[1]	Eastern Carpathian	G40	46.57	25.42	59.03	3.42	1.19	3.72	37	234	23.40	14.00		10.00	0.16	
GEOROC[1]	Eastern Carpathian	H30	46.57	25.58	60.94	3.07	2.07	3.23	76	376	20.60	24.00		18.25	0.20	
GEOROC[1]	Eastern Carpathian	H27	46.45	25.67	65.88	0.79	2.26	4.68	81	274	24.80	35.00		11.05	0.30	
GEOROC[1]	Eastern Carpathian	H24	46.42	25.68	63.15	2.91	1.98	3.46	78	317	21.00	26.00		15.10	0.25	
GEOROC[1]	Eastern Carpathian	H23	46.38	25.65	60.18	3.40	1.74	3.56	63	324	22.00	20.00		14.73	0.19	
GEOROC[1]	Eastern Carpathian	H21	46.37	25.67	57.90	3.20	1.42	3.50	42	469	23.70	26.00		19.79	0.09	
GEOROC[1]	Eastern Carpathian	H18	46.37	25.70	62.31	2.73	1.94	3.11	78	317	21.20	25.00		14.95	0.25	
GEOROC[1]	Eastern Carpathian	H8	46.35	25.85	61.85	3.12	2.11	3.05	80	308	21.50	25.00		14.33	0.26	
GEOROC[1]	Eastern Carpathian	H20	46.33	25.68	60.60	3.64	2.17	2.82	87	374	26.10	30.00		14.33	0.23	
GEOROC[1]	Eastern Carpathian	H16	46.30	25.80	65.95	2.81	2.55	2.88	102	223	20.30	27.00		10.99	0.46	
GEOROC[1]	Eastern Carpathian	H17	46.13	25.73	59.06	3.32	1.81	3.47	63	337	26.90	25.00		12.53	0.19	
GEOROC[1]	Eastern Carpathian	H51	46.10	25.67	56.02	3.94	1.22	3.13	42	372	21.70	17.00		17.14	0.11	
GEOROC[1]	Eastern Carpathian	G19	46.82	25.20	57.86	3.26	1.34	3.65	43	236	22.80	14.00		10.35	0.18	
GEOROC[1]	Greater Caucasus (W)	50-8	43.53	42.84	57.81	3.64	2.43	3.90	61	603	20.00			30.15	0.10	
GEOROC[1]	Greater Caucasus (W)	50-3	43.53	42.84	55.93	4.37	2.55	4.02	59	738	20.00			36.90	0.08	
GEOROC[1]	Greater Caucasus (W)	E-37	43.52	42.78	64.26	2.13	3.14	4.03	99	348	20.00			17.40	0.28	
GEOROC[1]	Greater Caucasus (W)	E-38	43.52	42.78	65.12	1.92	3.20	4.00	122	347	17.00			20.41	0.35	
GEOROC[1]	Greater Caucasus (W)	E-42	43.52	42.78	64.39	2.29	2.98	3.97	113	353	18.00			19.61	0.32	
Chernyshev et al., (2014)[2]	Greater Caucasus (W)	MI-40/97	43.35	42.44	71.61	0.76	3.68	3.27	202	231	8.00			28.88	0.87	
Chernyshev et al., (2014)[2]	Greater Caucasus (W)	218-3/0	43.35	42.44	71.47	1.23	3.63	3.73	165	225	21.00			10.71	0.73	
Chernyshev et al., (2014)[2]	Greater Caucasus (W)	221-1/0	43.35	42.44	71.14	0.87	3.97	3.47	180	202	22.00			9.18	0.89	
Chernyshev et al., (2014)[2]	Greater Caucasus (W)	236-1/0	43.35	42.44	70.82	1.57	3.99	2.89	191	254	24.00			10.58	0.75	
Chernyshev et al., (2014)[2]	Greater Caucasus (W)	Bt-15/97	43.35	42.44	70.91	1.13	3.47	3.68	142	226	18.00			12.56	0.63	
Chernyshev et al., (2014)[2]	Greater Caucasus (W)	Bt-5/97	43.35	42.44	67.26	1.54	3.00	4.27	121	263	21.00			12.52	0.46	
Chernyshev et al., (2014)[2]	Greater Caucasus (W)	209/0	43.35	42.44	69.38	1.29	3.35	3.87	153	270	20.00			13.50	0.57	
Chernyshev et al., (2014)[2]	Greater Caucasus (W)	217-1/0	43.35	42.44	69.83	1.09	3.41	4.16	159	243	21.00			11.57	0.65	
Chernyshev et al., (2014)[2]	Greater Caucasus (W)	Bt-8/97	43.35	42.44	69.40	0.99	3.42	4.08	158	273	22.00			12.41	0.58	
Chernyshev et al., (2014)[2]	Greater Caucasus (W)	Bt-13/97	43.35	42.44	68.68	1.14	3.72	4.17	191	287	7.00			41.00	0.67	
Chernyshev et al., (2014)[2]	Greater Caucasus (W)	Bt-2/97	43.35	42.44	69.01	1.16	3.65	4.03	183	287	6.00			47.83	0.64	
Chernyshev et al., (2014)[2]	Greater Caucasus (W)	211/0	43.35	42.44	70.11	1.39	3.54	3.77	153	251	18.00			13.94	0.61	
GEOROC[1]	Greater Caucasus (W)	E-20	43.33	42.68	68.43	1.36	3.55	3.91	161	234	16.00			14.63	0.69	
GEOROC[1]	Greater Caucasus (W)	E-23	43.33	42.68	65.60	1.85	3.21	4.13	131	311	24.00			12.96	0.42	
GEOROC[1]	Greater Caucasus (W)	E-34	43.33	42.68	67.89	1.44	3.77	3.87	167	247	17.00			14.53	0.68	
GEOROC[1]	Greater Caucasus (W)	Bt-6	43.30	42.47	68.08	0.91	3.19	3.89	135	368	11.00			33.45	0.37	
GEOROC[1]	Greater Caucasus (W)	Bt-17	43.30	42.47	70.11	0.54	3.51	3.91	142	378	11.00			34.36	0.38	
GEOROC[1]	Greater Caucasus (W)	MI-36	43.30	42.47	68.00	0.85	3.39	4.00	159	389	6.00			64.83	0.41	
GEOROC[1]	Greater Caucasus (W)	MI-37	43.30	42.47	68.45	0.88	3.35	3.77	160	359	8.00			44.88	0.45	
GEOROC[1]	Greater Caucasus (W)	MI-41	43.30	42.47	67.62	1.02	3.24	4.02	148	392	8.00			49.00	0.38	
GEOROC[1]	Greater Caucasus (W)	MI-43	43.30	42.47	68.20	0.86	3.41	3.92	158	365	15.00			24.33	0.43	

Source	Location	Sample	Latitude	Longitude	SiO2(wt.%)	MgO(wt.%)	K2O(wt.%)	Na2O(wt.%)	Rb(ppm)	Sr(ppm)	Y(ppm)	La(ppm)	Yb(ppm)	Sr/Y	Rb/Sr	La/Yb
GEOROC[1]	Greater Caucasus (W)	Ea-21	43.30	42.47	65.64	1.84	2.77	3.77	114	433	8.00			54.13	0.26	
GEOROC[1]	Greater Caucasus (W)	Ea-22	43.30	42.47	66.27	1.82	2.72	4.27	126	345	9.00			38.33	0.37	
GEOROC[1]	Greater Caucasus (W)	Ea-23	43.30	42.47	65.18	2.10	2.84	3.99	114	355	8.00			44.38	0.32	
GEOROC[1]	Greater Caucasus (W)	Ea-25	43.30	42.47	68.11	1.60	3.90	3.99	177	283	7.00			40.43	0.63	
GEOROC[1]	Greater Caucasus (W)	Ea-26	43.30	42.47	67.33	1.77	3.20	3.27	139	375	9.00			41.67	0.37	
GEOROC[1]	Greater Caucasus (W)	Ea-28	43.30	42.47	66.42	1.82	3.13	4.00	138	392	6.00			65.33	0.35	
GEOROC[1]	Greater Caucasus (W)	Ea-29	43.30	42.47	67.22	1.89	3.24	3.63	144	375	9.00			41.67	0.38	
GEOROC[1]	Greater Caucasus (W)	Ea-30	43.30	42.47	66.58	2.25	3.18	3.27	140	398	7.00			56.86	0.35	
GEOROC[1]	Greater Caucasus (W)	Ea-31	43.30	42.47	66.59	1.92	3.40	3.53	143	390	7.00			55.71	0.37	
GEOROC[1]	Greater Caucasus (W)	E-1	43.30	42.47	70.10	0.93	3.54	3.63	152	298	21.00			14.19	0.51	
GEOROC[1]	Greater Caucasus (W)	E-2	43.30	42.47	67.87	1.05	3.15	3.91	132	384	21.00			18.29	0.34	
GEOROC[1]	Greater Caucasus (W)	E-3	43.30	42.47	68.25	0.85	3.26	3.87	139	373	21.00			17.76	0.37	
GEOROC[1]	Greater Caucasus (W)	E-4	43.30	42.47	68.35	1.19	3.00	3.90	125	335	20.00			16.75	0.37	
GEOROC[1]	Greater Caucasus (W)	E-5	43.30	42.47	69.14	1.08	3.16	3.88	128	316	20.00			15.80	0.41	
GEOROC[1]	Greater Caucasus (W)	E-6	43.30	42.47	69.38	1.09	3.59	3.89	162	296	21.00			14.10	0.55	
GEOROC[1]	Greater Caucasus (W)	E-7	43.30	42.47	70.41	1.18	3.72	3.56	166	279	19.00			14.68	0.59	
GEOROC[1]	Greater Caucasus (W)	E-8	43.30	42.47	69.78	1.09	3.36	3.87	147	288	20.00			14.40	0.51	
GEOROC[1]	Greater Caucasus (W)	20	43.35	42.44	68.70	1.46	4.00	3.76	142	317	9.00	48.90	1.40	35.22	0.45	34.93
GEOROC[1]	Greater Caucasus (W)	27	43.35	42.44	67.20	1.49	2.93	4.06	174	51	14.00	48.10	1.60	3.64	3.41	30.06
GEOROC[1]	Greater Caucasus (W)	43	43.35	42.44	67.27	1.06	3.22	3.74	172	604	14.00	55.00	1.70	43.14	0.28	32.35
GEOROC[1]	Greater Caucasus (W)	32	43.35	42.44	67.20	1.54	3.39	4.21	146	389	11.00	46.90	1.50	35.36	0.38	31.27
GEOROC[1]	Greater Caucasus (W)	45	43.35	42.44	66.46	1.05	3.23	3.60	168	256	13.00	54.70	1.60	19.69	0.66	34.19
GEOROC[1]	Greater Caucasus (W)	48	43.35	42.44	66.40	1.44	3.31	4.31	154	278	13.00	56.60	1.80	21.38	0.55	31.44
GEOROC[1]	Greater Caucasus (W)	22	43.35	42.44	67.70	0.82	3.37	4.31	183	378	11.00	40.20	1.30	34.36	0.48	30.92
GEOROC[1]	Greater Caucasus (W)	23	43.35	42.44	67.50	1.45	3.37	4.32	152	207	12.00	43.00	1.50	17.25	0.73	28.67
GEOROC[1]	Greater Caucasus (W)	7	43.35	42.44	66.80	0.82	3.49	4.20	115	318	9.00	36.10	1.20	35.33	0.36	30.08
GEOROC[1]	Greater Caucasus (W)	6	43.35	42.44	66.79	0.92	3.70	3.34	140	295	14.00	40.20	1.40	21.07	0.47	28.71
GEOROC[1]	Greater Caucasus (W)	396	43.35	42.44	66.80	1.40	3.47	4.23	166	320	25.00	46.30	1.31	12.80	0.52	35.34
GEOROC[1]	Greater Caucasus (W)	338	43.35	42.44	67.40	1.57	3.66	4.00	158	332	28.00	48.40	1.43	11.86	0.48	33.85
GEOROC[1]	Greater Caucasus (W)	10	43.35	42.44	67.50	1.86	3.26	4.31	92	342	11.00	40.30	1.52	31.09	0.27	26.51
GEOROC[1]	Greater Caucasus (W)	340	43.35	42.44	64.40	1.64	3.22	4.54	118	308	29.00	39.80	1.55	10.62	0.38	25.68
Lebedev et al., (2009)[3]	Greater Caucasus (E)	SU-III	42.70	44.43	60.64	5.24	2.16	4.83	59	712	20.00			35.60	0.08	
Lebedev et al., (2009)[3]	Greater Caucasus (E)	SU-II	42.70	44.43	60.09	4.15	1.72	5.20	45	598	20.00			29.90	0.08	
Lebedev et al., (2009)[3]	Greater Caucasus (E)	SU-I	42.70	44.43	58.45	4.56	1.90	5.11	55	608	25.00			24.32	0.09	
Lebedev et al., (2009)[3]	Greater Caucasus (E)	SU-24	42.70	44.43	60.92	4.34	1.62	5.31	45	541	25.00			21.64	0.08	
Lebedev et al., (2009)[3]	Greater Caucasus (E)	SU-23	42.70	44.43	58.22	4.79	1.79	5.13	40	691	24.00			28.79	0.06	
Lebedev et al., (2009)[3]	Greater Caucasus (E)	SU-22	42.70	44.43	65.29	3.15	1.65	5.40	45	570	18.00			31.67	0.08	
Lebedev et al., (2009)[3]	Greater Caucasus (E)	SU-21	42.69	44.43	63.87	2.90	1.86	5.07	55	479	19.00			25.21	0.11	
Lebedev et al., (2009)[3]	Greater Caucasus (E)	SU-20	42.69	44.43	64.24	3.75	1.78	5.37	50	564	19.00			29.68	0.09	
Lebedev et al., (2009)[3]	Greater Caucasus (E)	GZ-71	42.69	44.43	66.86	3.50	0.48	4.43	16	521	21.00			24.81	0.03	
Lebedev et al., (2009)[3]	Greater Caucasus (E)	GZ-70*	42.69	44.43	64.23	3.62	2.15	3.81	63	523	20.00			26.15	0.12	
Lebedev et al., (2009)[3]	Greater Caucasus (E)	SU-8	42.69	44.43	60.99	4.42	1.87	3.81	86	535	26.00			20.58	0.16	
Lebedev et al., (2009)[3]	Greater Caucasus (E)	SU-6	42.69	44.43	65.90	3.19	0.33	5.07	13	620	35.00			17.71	0.02	

Source	Location	Sample	Latitude	Longitude	SiO2(wt.%)	MgO(wt.%)	K2O(wt.%)	Na2O(wt.%)	Rb(ppm)	Sr(ppm)	Y(ppm)	La(ppm)	Yb(ppm)	Sr/Y	Rb/Sr	La/Yb
GEOROC[1]	Southeastern Lesser Caucasus	120	39.86	46.02	55.67	4.66	2.60	4.22	55	730	21.00	52.00	2.00	34.76	0.08	26.00
GEOROC[1]	Southeastern Lesser Caucasus	180	39.86	46.02	55.21	2.50	3.11	5.04	43	1190	27.00	69.00	2.20	44.07	0.04	31.36
GEOROC[1]	Southeastern Lesser Caucasus	13	39.86	46.02	57.66	3.18	3.01	3.85	55	1360	24.00	60.00	1.80	56.67	0.04	33.33
GEOROC[1]	Southeastern Lesser Caucasus	25	39.86	46.02	58.52	3.23	2.80	4.00	49	1275	32.00	60.00	1.90	39.84	0.04	31.58
GEOROC[1]	Southeastern Lesser Caucasus	33	39.86	46.02	59.85	2.67	3.11	4.38	66	1615	32.00	70.00	2.00	50.47	0.04	35.00
GEOROC[1]	Southeastern Lesser Caucasus	160	39.86	46.02	59.28	2.79	3.46	4.65	56	1360	19.00	67.00	2.10	71.58	0.04	31.90
GEOROC[1]	Southeastern Lesser Caucasus	185	39.86	46.02	57.85	2.77	2.89	4.53	48	790	15.00	48.00	1.30	52.67	0.06	36.92
GEOROC[1]	Northern Lesser Caucasus	S21.1	41.13	43.69	63.94	2.63	2.84	4.07	58	395	21.47	38.74	1.92	18.37	0.15	20.17
GEOROC[1]	Northern Lesser Caucasus	S1.1	41.05	43.82	57.88	3.05	1.98	4.00	44	516	27.21	31.01	2.49	18.97	0.08	12.43
GEOROC[1]	Northern Lesser Caucasus	S1.2	41.05	43.82	57.84	3.11	1.98	4.07	46	529	27.86	30.28	2.50	18.97	0.09	12.12
GEOROC[1]	Northern Lesser Caucasus	S11.1	41.01	43.94	60.03	2.95	1.97	3.71	48	538	22.02	28.37	1.92	24.42	0.09	14.76
GEOROC[1]	Northern Lesser Caucasus	S12.1	41.07	44.10	65.45	1.51	2.33	3.95	69	477	11.17	27.78	0.96	42.71	0.14	29.07
GEOROC[1]	Northern Lesser Caucasus	S15.1	41.02	43.91	59.31	4.06	1.96	4.07	41	504	18.84	33.89	1.82	26.75	0.08	18.64
GEOROC[1]	Northern Lesser Caucasus	S16.1	41.02	43.89	58.50	3.26	1.76	4.16	39	527	26.80	31.04	2.49	19.66	0.07	12.48
GEOROC[1]	Northern Lesser Caucasus	S17.1	41.01	43.86	58.86	3.23	1.72	4.06	37	519	24.73	29.87	2.45	21.00	0.07	12.19
GEOROC[1]	Northern Lesser Caucasus	S18.1	41.08	43.80	57.56	4.66	1.59	3.95	26	492	24.01	32.14	2.55	20.51	0.05	12.61
GEOROC[1]	Northern Lesser Caucasus	S2.1	41.04	43.82	58.27	2.87	2.00	3.94	44	503	25.72	28.53	2.32	19.54	0.09	12.31
GEOROC[1]	Northern Lesser Caucasus	S20.1	41.09	43.66	61.95	3.00	2.47	4.15	49	453	18.82	37.60	1.84	24.05	0.11	20.41
GEOROC[1]	Northern Lesser Caucasus	S20.2	41.09	43.66	61.84	3.06	2.57	4.11	51	458	18.58	36.38	1.81	24.67	0.11	20.12
GEOROC[1]	Northern Lesser Caucasus	S22.1	41.08	43.61	62.27	3.20	2.28	3.78	55	392	19.70	29.19	1.87	19.89	0.14	15.57
GEOROC[1]	Northern Lesser Caucasus	S23.1	41.09	43.59	62.39	3.30	2.23	4.18	58	405	19.15	26.71	1.84	21.16	0.14	14.50
GEOROC[1]	Northern Lesser Caucasus	S24.1	41.10	43.56	62.70	2.63	2.48	4.01	64	435	17.77	26.65	1.63	24.49	0.15	16.40
GEOROC[1]	Northern Lesser Caucasus	S3.2	41.07	43.94	58.28	3.18	1.91	4.12	44	523	28.75	31.33	2.52	18.20	0.08	12.41
GEOROC[1]	Northern Lesser Caucasus	S30.1	41.12	43.90	58.82	3.55	1.84	4.33	45	584	20.20	27.53	1.83	28.92	0.08	15.05
GEOROC[1]	Northern Lesser Caucasus	S30.2	41.11	43.92	58.97	3.46	1.90	3.98	47	603	20.42	27.83	1.87	29.53	0.08	14.86
GEOROC[1]	Northern Lesser Caucasus	S30.3	41.11	43.92	68.24	1.55	2.34	3.79	74	433	12.45	26.11	1.11	34.76	0.17	23.44
GEOROC[1]	Northern Lesser Caucasus	S4.1	41.08	43.95	59.73	3.44	1.90	3.96	46	531	21.54	27.34	1.86	24.66	0.09	14.72
GEOROC[1]	Northern Lesser Caucasus	S4.2	41.08	43.95	57.67	3.36	1.83	3.77	42	562	27.22	31.76	2.36	20.64	0.07	13.45
GEOROC[1]	Northern Lesser Caucasus	S5.1	41.09	43.94	61.57	2.95	2.04	3.85	48	635	16.12	33.31	1.38	39.38	0.08	24.22
GEOROC[1]	Northern Lesser Caucasus	S6.1	41.09	43.94	62.18	2.75	2.18	3.82	52	662	16.22	36.64	1.35	40.79	0.08	27.17
GEOROC[1]	Northern Lesser Caucasus	S7.1	41.05	43.67	60.67	3.14	2.26	4.27	53	676	19.49	39.07	1.63	34.67	0.08	23.92
GEOROC[1]	Northern Lesser Caucasus	S7.2	41.05	43.67	60.43	3.40	2.28	4.23	52	690	19.38	39.10	1.63	35.60	0.08	23.95
GEOROC[1]	Northern Lesser Caucasus	S7.3	41.05	43.67	60.86	3.39	2.24	3.87	53	696	19.19	38.31	1.60	36.27	0.08	23.93
GEOROC[1]	Northern Lesser Caucasus	S7.4	41.05	43.67	60.15	3.75	2.13	4.27	50	701	19.03	38.16	1.64	36.85	0.07	23.23
GEOROC[1]	Northern Lesser Caucasus	S7.5	41.05	43.67	60.30	3.66	2.13	4.14	50	705	19.31	38.12	1.62	36.50	0.07	23.51
GEOROC[1]	Northern Lesser Caucasus	S8.1	41.01	43.94	60.22	2.71	1.98	4.18	48	539	22.60	28.40	1.93	23.85	0.09	14.69
GEOROC[1]	Northern Lesser Caucasus	S9.1	41.07	44.00	64.15	1.95	2.04	4.34	45	558	11.28	26.00	0.98	49.47	0.08	26.54
GEOROC[1]	Northern Lesser Caucasus	S9.2	41.07	43.99	65.80	1.77	2.21	4.12	57	540	11.65	27.38	1.01	46.39	0.11	27.23
GEOROC[1]	Northwestern Lesser Caucasus	YUG-151	41.81	43.45	62.63	2.45	2.17	4.18	33	621	15.00			41.40	0.05	
GEOROC[1]	Northwestern Lesser Caucasus	YUG-150	41.84	43.41	62.50	2.53	2.07	4.21	36	607	13.00			46.69	0.06	
GEOROC[1]	Northwestern Lesser Caucasus	YUG-154	41.74	43.52	61.71	2.40	2.10	4.23	39	575	16.00			35.94	0.07	
GEOROC[1]	Northwestern Lesser Caucasus	YUG-79	41.73	43.46	59.47	2.56	1.94	4.27	25	634	26.00			24.38	0.04	

Source	Location	Sample	Latitude	Longitude	SiO2(wt.%)	MgO(wt.%)	K2O(wt.%)	Na2O(wt.%)	Rb(ppm)	Sr(ppm)	Y(ppm)	La(ppm)	Yb(ppm)	Sr/Y	Rb/Sr	La/Yb
GEOROC[1]	Northwestern Lesser Caucasus	YUG-155	41.74	43.46	59.47	2.50	1.77	4.36	17	643	22.00			29.23	0.03	
GEOROC[1]	Northwestern Lesser Caucasus	YUG-152	41.80	43.49	59.46	2.58	1.99	4.58	29	632	22.00			28.73	0.05	
GEOROC[1]	Northwestern Lesser Caucasus	AR-1/07	41.36	44.46	58.45	5.28	1.99	3.41	60	480	20.00			24.00	0.13	
GEOROC[1]	Northwestern Lesser Caucasus	YUG-153	41.79	43.58	58.19	2.79	2.04	5.03	27	611	23.00			26.57	0.04	
Lebedev et al., (2008a)[4]	Northwestern Lesser Caucasus	YUG-17	41.42	43.48	64.97	2.13	2.31	3.31	60	402	21.00			19.14	0.15	
Lebedev et al., (2008a)[4]	Northwestern Lesser Caucasus	YUG-32	41.36	43.28	64.20	1.37	2.33	3.82	56	386	29.00			13.31	0.15	
Lebedev et al., (2008a)[4]	Northwestern Lesser Caucasus	YUG-51	41.49	43.38	56.07	3.32	0.83	2.52	12	467	30.00			15.57	0.03	
Lebedev et al., (2008a)[4]	Northwestern Lesser Caucasus	YUG-61	41.32	43.43	56.03	3.41	1.36	3.72	23	458	25.00			18.32	0.05	
Lebedev et al., (2008a)[4]	Northwestern Lesser Caucasus	YUG-50	41.49	43.38	55.91	3.92	0.89	2.98	14	457	27.00			16.93	0.03	
Lebedev et al., (2008a)[4]	Northwestern Lesser Caucasus	YUG-9	41.43	43.47	55.86	4.49	1.23	3.35	16	544	26.00			20.92	0.03	
Lebedev et al., (2008a)[4]	Northwestern Lesser Caucasus	YUG-174	41.59	44.13	55.85	4.86	1.53	3.74	21	533	24.00			22.21	0.04	
Lebedev et al., (2008a)[4]	Northwestern Lesser Caucasus	YUG-60	41.32	43.43	55.82	3.59	1.54	3.73	29	469	27.00			17.37	0.06	
Lebedev et al., (2008a)[4]	Northwestern Lesser Caucasus	YUG-8	41.43	43.47	55.51	4.38	1.17	3.24	17	562	26.00			21.62	0.03	
Lebedev et al., (2008a)[4]	Northwestern Lesser Caucasus	YUG-52	41.49	43.38	55.01	5.36	0.85	2.89	11	458	27.00			16.96	0.02	
Lebedev et al., (2008b)[5]	Northwestern Lesser Caucasus	YUG-118	41.50	44.11	69.32	0.65	2.37	3.63	68	615	13.00			47.31	0.11	
Lebedev et al., (2008b)[5]	Northwestern Lesser Caucasus	YUG-113	41.37	44.12	64.27	1.32	1.90	3.51	42	527	18.00			29.28	0.08	
Lebedev et al., (2008b)[5]	Northwestern Lesser Caucasus	YUG-112	41.37	44.13	63.77	1.30	1.80	3.52	42	543	17.00			31.94	0.08	
Lebedev et al., (2008b)[5]	Northwestern Lesser Caucasus	YUG-122	41.60	44.09	58.90	2.55	1.83	3.55	29	533	26.00			20.50	0.05	
Lebedev et al., (2008b)[5]	Northwestern Lesser Caucasus	YUG-115	41.42	44.10	58.42	2.90	2.04	3.47	34	547	28.00			19.54	0.06	
Lebedev et al., (2008b)[5]	Northwestern Lesser Caucasus	YUG-125	41.51	43.87	56.67	3.66	1.63	3.86	25	538	27.00			19.93	0.05	
Lebedev et al., (2008b)[5]	Northwestern Lesser Caucasus	YUG-121	41.60	44.08	56.67	4.07	1.75	3.67	27	582	25.00			23.28	0.05	
Lebedev et al., (2008b)[5]	Northwestern Lesser Caucasus	YUG-116	41.43	44.11	56.61	2.89	1.95	3.97	27	644	25.00			25.76	0.04	
Lebedev et al., (2008b)[5]	Northwestern Lesser Caucasus	YUG-123	41.58	43.97	56.47	3.06	1.82	3.83	27	604	30.00			20.13	0.04	
Lebedev et al., (2008b)[5]	Northwestern Lesser Caucasus	YUG-119	41.60	44.11	56.41	4.79	1.77	3.50	29	598	25.00			23.92	0.05	
Lebedev et al., (2008b)[5]	Northwestern Lesser Caucasus	YUG-120	41.60	44.08	56.27	3.67	1.36	3.58	19	539	23.00			23.43	0.04	
Nomade et al., (2016)[6]	Northwestern Lesser Caucasus	TS-10-08	41.53	44.12	57.20	4.05	1.97	3.81	39	555	24.60	34.40	2.20	22.56	0.07	15.64
Nomade et al., (2016)[6]	Northwestern Lesser Caucasus	TS-05-08	41.65	44.11	56.10	4.79	1.74	3.99	32	618	24.40	34.80	2.10	25.34	0.05	16.57
Nomade et al., (2016)[6]	Northwestern Lesser Caucasus	TS-08-08	41.57	43.90	55.60	4.86	1.64	4.08	26	572	27.20	34.20	2.40	21.03	0.05	14.25
Nomade et al., (2016)[6]	Northwestern Lesser Caucasus	GODO-10-01	41.66	43.67	65.70	2.01	2.06	4.21	46	539	12.80	28.90	1.10	42.09	0.09	26.27
Nomade et al., (2016)[6]	Northwestern Lesser Caucasus	TS-07-08	41.65	43.84	64.90	2.55	1.94	4.04	42	526	11.70	28.70	0.90	44.97	0.08	31.89
Nomade et al., (2016)[6]	Northwestern Lesser Caucasus	SAM-94	41.65	43.73	64.80	1.95	2.07	4.23	45	544	12.40	29.10	1.10	43.85	0.08	26.45
Nomade et al., (2016)[6]	Northwestern Lesser Caucasus	MAR10-01	41.37	44.12	63.50	2.78	1.78	4.03	41	518	12.60	24.60	1.10	41.10	0.08	22.36
Nomade et al., (2016)[6]	Northwestern Lesser Caucasus	SAM-97	41.45	43.77	61.80	2.27	2.11	4.31	36	650	16.80	36.40	1.40	38.66	0.06	26.00
Nomade et al., (2016)[6]	Northwestern Lesser Caucasus	SAM-10-01	41.55	43.75	61.70	3.50	1.97	3.93	46	575	20.00	33.80	1.80	28.77	0.08	18.78
GEOROC[1]	Central Lesser Caucasus	32G/01	40.46	45.02	59.28	3.56	2.88	4.03	60	946	18.00			52.56	0.06	
GEOROC[1]	Central Lesser Caucasus	G-73/03	40.45	44.79	68.77	1.53	3.90	4.25	65	388	22.00			17.64	0.17	
GEOROC[1]	Central Lesser Caucasus	31G/01	40.39	45.21	57.04	3.78	2.47	3.98	54	756	26.00			29.08	0.07	
GEOROC[1]	Central Lesser Caucasus	30G/01	40.39	45.20	56.94	4.62	2.33	3.86	52	744	18.00			41.33	0.07	
GEOROC[1]	Central Lesser Caucasus	G-5/03	40.38	45.13	56.60	4.04	2.49	4.11	43	736	25.00			29.44	0.06	
GEOROC[1]	Central Lesser Caucasus	19G/01	40.32	44.69	70.86	0.59	3.61	5.04	99	332	21.00			15.81	0.30	
GEOROC[1]	Central Lesser Caucasus	8G/01	40.31	44.91	57.84	4.36	2.51	3.83	62	655	24.00			27.29	0.09	
GEOROC[1]	Central Lesser Caucasus	5bG/01	40.30	44.93	56.87	3.42	2.72	4.66	53	937	23.00			40.74	0.06	
GEOROC[1]	Central Lesser Caucasus	7G/01	40.30	44.90	57.43	3.93	2.27	3.62	57	693	23.00			30.13	0.08	

Source	Location	Sample	Latitude	Longitude	SiO2(wt.%)	MgO(wt.%)	K2O(wt.%)	Na2O(wt.%)	Rb(ppm)	Sr(ppm)	Y(ppm)	La(ppm)	Yb(ppm)	Sr/Y	Rb/Sr	La/Yb
GEOROC[1]	Central Lesser Caucasus	6G/01	40.30	44.92	58.99	3.74	2.56	4.20	65	659	25.00			26.36	0.10	
GEOROC[1]	Central Lesser Caucasus	G-47/03	40.30	45.00	57.60	4.56	2.51	3.82	45	765	25.00			30.60	0.06	
GEOROC[1]	Central Lesser Caucasus	4G/01	40.29	44.92	56.16	3.21	2.42	4.52	57	1097	24.00			45.71	0.05	
GEOROC[1]	Central Lesser Caucasus	G-110/03	40.28	44.67	71.35	0.64	3.69	4.84	92	354	25.00			14.16	0.26	
GEOROC[1]	Central Lesser Caucasus	G-108/03	40.24	44.60	64.93	1.34	4.28	4.51	86	344	36.00			9.56	0.25	
GEOROC[1]	Central Lesser Caucasus	33G/01	40.19	44.48	57.79	4.17	2.73	4.42	46	982	22.00			44.64	0.05	
GEOROC[1]	Central Lesser Caucasus	G-39/03	40.08	45.21	59.46	2.84	2.90	4.52	57	673	26.00			25.88	0.08	
GEOROC[1]	Central Lesser Caucasus	AR-9/07	40.40	44.46	68.25	1.12	2.74	4.47	120	320	20.00			16.00	0.38	
GEOROC[1]	Central Lesser Caucasus	AR-7/07	40.40	44.47	67.81	1.40	2.93	3.97	140	290	20.00			14.50	0.48	
GEOROC[1]	Central Lesser Caucasus	AR-6/07	40.40	44.49	67.79	1.35	3.05	3.91	130	280	20.00			14.00	0.46	
GEOROC[1]	Central Lesser Caucasus	AR-17/07	40.40	44.45	67.95	1.30	2.74	4.21	120	280	20.00			14.00	0.43	
GEOROC[1]	Central Lesser Caucasus	AR-3/07	40.40	44.46	57.89	4.87	1.94	3.61	70	530	20.00			26.50	0.13	
GEOROC[1]	Central Lesser Caucasus	AR-15/07	40.39	44.46	69.93	1.15	3.30	3.89	150	250	20.00			12.50	0.60	
GEOROC[1]	Central Lesser Caucasus	AR-13/07	40.39	44.46	68.15	1.38	3.39	3.87	130	280	10.00			28.00	0.46	
GEOROC[1]	Central Lesser Caucasus	AR-11/07	40.38	44.47	67.87	1.20	3.06	4.26	130	290	20.00			14.50	0.45	
GEOROC[1]	Central Lesser Caucasus	Y3.1	40.32	44.55	56.42	3.80	2.13	4.16	44	882	24.00	50.00	2.20	36.75	0.05	22.73
GEOROC[1]	Central Lesser Caucasus	Y4.1	40.32	44.58	57.24	4.39	2.80	4.49	45	1015	23.00	59.00	2.20	44.13	0.04	26.82
GEOROC[1]	Central Lesser Caucasus	Y9.2	40.17	44.38	58.75	3.97	1.62	3.82	36	519	18.90	26.00	1.80	27.46	0.07	14.44
Gao et al., (2015)[7]	Southeastern Tibet	TC-44	25.17	98.53	66.11	1.37	3.92	3.26	189	279	21.30	95.20	1.81	13.10	0.68	52.60
Gao et al., (2015)[7]	Southeastern Tibet	TC-45	25.17	98.53	66.47	1.42	3.74	3.14	192	286	17.60	64.90	1.43	16.25	0.67	45.38
Gao et al., (2015)[7]	Southeastern Tibet	TC-46	25.17	98.53	65.83	0.95	3.45	3.71	159	336	21.60	62.30	1.72	15.56	0.47	36.22
Gao et al., (2015)[7]	Southeastern Tibet	TC-60C	25.17	98.53	65.24	1.33	4.11	2.81	153	315	29.10	97.90	2.81	10.82	0.49	34.84
Gao et al., (2015)[7]	Southeastern Tibet	TC-61	25.17	98.53	65.12	0.60	3.64	3.23	156	292	20.10	71.40	1.94	14.53	0.54	36.80
Gao et al., (2015)[7]	Southeastern Tibet	TC-62	25.17	98.53	66.59	1.15	3.59	3.34	150	326	19.50	67.80	1.94	16.72	0.46	34.95
Gao et al., (2015)[7]	Southeastern Tibet	TC-63	25.17	98.53	66.10	1.00	3.60	3.39	156	328	25.00	85.90	2.32	13.12	0.48	37.03
Gao et al., (2015)[7]	Southeastern Tibet	TC-64	25.17	98.53	66.64	1.31	3.70	3.56	151	339	19.40	64.20	1.90	17.47	0.44	33.79
Gao et al., (2015)[7]	Southeastern Tibet	TC-47	25.17	98.53	62.16	1.57	3.23	2.36	146	180	19.20	58.30	1.52	9.38	0.81	38.36
Gao et al., (2015)[7]	Southeastern Tibet	TC-48	25.17	98.53	63.49	1.56	3.29	2.65	161	206	18.60	55.70	1.57	11.08	0.78	35.48
Gao et al., (2015)[7]	Southeastern Tibet	TC-49	25.17	98.53	61.54	1.61	3.40	2.30	148	160	18.40	56.20	1.62	8.70	0.93	34.69
Gao et al., (2015)[7]	Southeastern Tibet	TC-50	25.17	98.53	62.12	1.57	3.45	3.05	158	189	17.90	60.10	1.67	10.56	0.84	35.99
Gao et al., (2015)[7]	Southeastern Tibet	TC-51	25.17	98.53	59.13	1.47	3.45	2.09	160	124	17.20	58.80	1.81	7.21	1.29	32.49
Gao et al., (2015)[7]	Southeastern Tibet	TC-52	25.17	98.53	63.35	1.57	3.44	3.29	150	210	17.50	58.50	1.65	12.00	0.71	35.45
Gao et al., (2015)[7]	Southeastern Tibet	TC-53	25.17	98.53	62.97	1.61	3.45	2.42	157	186	19.30	58.30	1.60	9.64	0.84	36.44
Gao et al., (2015)[7]	Southeastern Tibet	TC-55	25.17	98.53	63.73	0.99	3.32	2.66	168	245	26.60	88.50	1.94	9.21	0.69	45.62
Zhou et al., (2012)[8]	Southeastern Tibet	TC-31 1	25.22	98.55	55.53	4.52	2.97	3.79	86	360	25.50	47.40	2.54	14.12	0.24	18.66
Zhou et al., (2012)[8]	Southeastern Tibet	TC-9	25.22	98.50	55.80	4.43	2.96	3.53	95	452	25.40	43.40	2.20	17.80	0.21	19.73
Zhou et al., (2012)[8]	Southeastern Tibet	TC-11	25.22	98.50	55.02	4.67	3.00	4.32	86	459	25.40	41.90	2.25	18.07	0.19	18.62
Zhou et al., (2012)[8]	Southeastern Tibet	TC-12	25.22	98.50	58.75	3.48	3.51	4.24	119	523	26.70	72.90	2.46	19.59	0.23	29.63
Zhou et al., (2012)[8]	Southeastern Tibet	TC-15	25.22	98.50	57.13	3.79	3.25	3.58	96	550	25.90	73.60	2.52	21.24	0.18	29.21
Zhou et al., (2012)[8]	Southeastern Tibet	TC-16	25.22	98.50	56.89	3.78	3.26	3.93	96	554	26.30	73.00	2.50	21.06	0.17	29.20
Zhou et al., (2012)[8]	Southeastern Tibet	TC-19	25.22	98.50	60.47	3.02	3.79	3.73	138	467	26.40	71.00	2.27	17.69	0.30	31.28
Zhou et al., (2012)[8]	Southeastern Tibet	TC-21	25.22	98.50	58.35	3.70	3.42	3.80	112	553	27.60	69.50	2.17	20.04	0.20	32.03
Zhou et al., (2012)[8]	Southeastern Tibet	TC-22	25.22	98.50	58.69	3.55	3.45	3.54	112	555	27.00	70.80	2.10	20.56	0.20	33.71

Source	Location	Sample	Latitude	Longitude	SiO2(wt.%)	MgO(wt.%)	K2O(wt.%)	Na2O(wt.%)	Rb(ppm)	Sr(ppm)	Y(ppm)	La(ppm)	Yb(ppm)	Sr/Y	Rb/Sr	La/Yb
Zhou et al., (2012)[8]	Southeastern Tibet	TC-23	25.22	98.50	57.33	3.76	3.35	3.95	99	551	26.30	70.50	2.36	20.95	0.18	29.87
Zhou et al., (2012)[8]	Southeastern Tibet	TC-26	25.22	98.50	57.34	3.86	3.33	3.85	97	550	25.50	73.70	2.46	21.57	0.18	29.96
Tucker et al., (2013)[9]	Southeastern Tibet	DA09026	25.17	98.01	61.50	2.43	4.02	3.60	137	457	30.10	86.80	2.63	15.18	0.30	33.00
Zhang et al., (2012)[10]	Southeastern Tibet	DY08-1	25.13	98.47	62.04	2.51	3.99	3.63	132	444	30.33	83.31	2.65	14.62	0.30	31.44
Zhang et al., (2012)[10]	Southeastern Tibet	GD08-1	25.31	98.48	55.38	5.98	2.42	3.43	80	450	23.15	45.08	2.31	19.45	0.18	19.52
Zhang et al., (2012)[10]	Southeastern Tibet	MA08-2	25.02	98.43	59.05	3.61	3.25	3.88	94	492	25.28	65.45	2.49	19.46	0.19	26.29
Zhang et al., (2012)[10]	Southeastern Tibet	MA08-3	25.02	98.43	58.73	3.55	3.36	3.83	99	514	26.81	65.10	2.61	19.15	0.19	24.94
Zhang et al., (2012)[10]	Southeastern Tibet	XC08-1	25.03	98.40	58.55	3.71	3.17	3.82	98	551	27.28	67.47	2.69	20.18	0.18	25.08
Zhang et al., (2012)[10]	Southeastern Tibet	HS08-1	25.28	98.48	58.81	3.38	3.11	3.30	110	484	24.68	75.99	2.19	19.60	0.23	34.70
Zhang et al., (2012)[10]	Southeastern Tibet	JS08-2	25.23	98.48	55.06	4.88	2.58	3.73	69	423	24.98	42.74	2.37	16.92	0.16	18.03
Zhang et al., (2012)[10]	Southeastern Tibet	JS08-3	25.23	98.48	55.39	4.79	2.60	3.78	69	437	25.33	43.67	2.47	17.24	0.16	17.68
Zhang et al., (2012)[10]	Southeastern Tibet	TS08-1	25.23	98.47	58.93	4.06	3.25	3.63	99	518	25.97	71.41	2.44	19.94	0.19	29.27
Zhang et al., (2012)[10]	Southeastern Tibet	BH08-1	25.11	98.55	66.92	1.79	3.61	3.47	157	314	19.95	67.23	1.96	15.74	0.50	34.30
Zhang et al., (2012)[10]	Southeastern Tibet	LW08-3	25.05	98.55	66.69	0.94	3.66	3.27	157	320	21.84	72.53	2.08	14.66	0.49	34.87
Zhang et al., (2012)[10]	Southeastern Tibet	TT08-2	24.75	98.63	62.05	2.76	3.25	3.62	95	564	25.38	76.97	2.45	22.21	0.17	31.42
Zhang et al., (2012)[10]	Southeastern Tibet	TT08-4	24.74	98.64	61.24	2.92	3.43	3.35	88	582	25.48	81.40	2.47	22.83	0.15	32.96
Zhang et al., (2012)[10]	Southeastern Tibet	DP08-1	25.13	98.47	61.25	2.68	3.78	3.71	128	484	28.95	80.86	2.62	16.71	0.26	30.86
Zhang et al., (2012)[10]	Southeastern Tibet	HY08-1	25.22	98.58	56.04	4.90	2.84	3.54	84	355	25.97	49.32	2.60	13.67	0.24	18.97
Shi et al., (2012)[11]	Southeastern Tibet	SD018-1	24.72	98.65	62.93	1.88	3.64	3.54	118	521	31.50	84.20	2.58	16.52	0.23	32.64
Shi et al., (2012)[11]	Southeastern Tibet	SD018-2	24.72	98.65	62.85	1.77	3.62	3.53	113	502	33.80	88.90	2.83	14.85	0.22	31.41
Shi et al., (2012)[11]	Southeastern Tibet	SD024-2-1	24.72	98.64	60.84	2.36	3.33	3.62	102	651	26.00	75.30	2.33	25.03	0.16	32.32
Shi et al., (2012)[11]	Southeastern Tibet	SD024-2-2	24.72	98.64	60.77	2.32	3.34	3.66	100	643	25.50	75.70	2.42	25.21	0.16	31.28
Li et al., (2015)[12]	Southeastern Tibet	TC1	25.20	98.58	62.02	1.97	3.39	3.19	119	445	20.70	66.10	1.85	21.50	0.27	35.73
Li et al., (2015)[12]	Southeastern Tibet	TC7	25.20	98.58	66.90	0.67	3.58	3.20	145	309	17.40	63.00	1.71	17.76	0.47	36.84
Li et al., (2015)[12]	Southeastern Tibet	TC16	25.20	98.58	56.42	3.61	2.95	3.48	79	486	24.50	59.20	2.24	19.84	0.16	26.43
Xu et al., (2012)[13]	Southeastern Tibet	TC-01	25.21	98.49	55.61	3.53	2.20	3.80	46	471	22.65	36.10	2.58	20.79	0.10	13.99
Xu et al., (2012)[13]	Southeastern Tibet	TC-02	25.21	98.49	56.07	3.77	1.85	3.44	47	424	23.75	34.87	2.55	17.87	0.11	13.67
Xu et al., (2012)[13]	Southeastern Tibet	TC-04	25.21	98.49	55.69	3.87	2.16	4.21	46	463	22.78	37.20	2.47	20.34	0.10	15.06
Xu et al., (2012)[13]	Southeastern Tibet	TC-05	25.21	98.49	58.45	3.07	2.57	3.67	72	474	24.18	44.02	2.63	19.59	0.15	16.74
Xu et al., (2012)[13]	Southeastern Tibet	TC-06	25.21	98.49	55.54	3.29	2.30	3.79	52	374	22.19	38.48	2.53	16.85	0.14	15.21
Xu et al., (2012)[13]	Southeastern Tibet	TC-09	25.26	98.50	56.43	3.22	2.35	3.71	53	284	23.62	40.29	2.59	12.02	0.19	15.56
Xu et al., (2012)[13]	Southeastern Tibet	TC-11	25.26	98.50	61.13	3.03	3.18	3.71	96	279	24.80	72.69	2.59	11.25	0.34	28.07
Li and Liu, (2012)[14]	Southeastern Tibet	HSK01	25.17	98.50	58.78	4.05	3.38	3.60	103	542	26.51	74.22	2.42	20.45	0.19	30.67
Li and Liu, (2012)[14]	Southeastern Tibet	HSK02	25.17	98.50	59.07	3.35	3.03	3.29	124	478	23.74	76.28	2.19	20.13	0.26	34.83
Li and Liu, (2012)[14]	Southeastern Tibet	HSK03	25.17	98.50	59.69	3.47	3.64	3.55	118	483	25.51	68.64	2.47	18.93	0.24	27.79
Li and Liu, (2012)[14]	Southeastern Tibet	HSK04	25.17	98.50	59.86	3.11	3.19	3.35	113	390	20.35	62.27	1.87	19.16	0.29	33.30
Li and Liu, (2012)[14]	Southeastern Tibet	DYS01	25.17	98.50	61.39	2.44	3.98	3.55	130	481	30.37	86.84	2.84	15.84	0.27	30.58
Li and Liu, (2012)[14]	Southeastern Tibet	DYS02	25.17	98.50	60.54	2.53	3.88	3.61	129	448	27.35	77.32	2.64	16.38	0.29	29.29
Li and Liu, (2012)[14]	Southeastern Tibet	DYS03	25.17	98.50	67.68	1.69	3.75	3.25	187	294	20.21	68.13	1.83	14.55	0.64	37.23
Li and Liu, (2012)[14]	Southeastern Tibet	DYS04	25.17	98.50	64.64	1.91	3.94	2.91	153	273	19.34	61.75	1.81	14.12	0.56	34.12
Li and Liu, (2012)[14]	Southeastern Tibet	DYS05	25.17	98.50	58.58	2.68	3.68	3.52	114	489	28.85	83.51	2.88	16.95	0.23	29.00
Li and Liu, (2012)[14]	Southeastern Tibet	MAS05	25.17	98.50	57.44	3.62	3.26	3.72	95	590	27.33	68.36	2.66	21.59	0.16	25.70
Li and Liu, (2012)[14]	Southeastern Tibet	MAS07	25.17	98.50	58.45	3.65	3.36	3.71	109	566	27.53	69.70	2.68	20.56	0.19	26.01

Source	Location	Sample	Latitude	Longitude	SiO2(wt.%)	MgO(wt.%)	K2O(wt.%)	Na2O(wt.%)	Rb(ppm)	Sr(ppm)	Y(ppm)	La(ppm)	Yb(ppm)	Sr/Y	Rb/Sr	La/Yb
Zhang et al., (2008)[15]	Northen Tibet (W)	PL-9	36.28	81.48	55.18	3.97	4.14	3.64	119	1060	24.59	144.00	2.05	43.11	0.11	70.24
Wang and Zhang, (2011)[16]	Northen Tibet (W)	CY2-6	36.28	81.48	55.12	3.80	3.96	3.40	107	1215	28.70	129.00	1.89	42.33	0.09	68.25
Ning et al., (2009)[17]	Northen Tibet (W)	B33062	35.48	82.80	59.39	2.19	4.22	3.13	190	854	32.92	182.90	2.24	25.94	0.22	81.65
Ning et al., (2009)[17]	Northen Tibet (W)	B33065	35.48	82.80	60.70	1.97	4.30	3.44	218	858	30.96	201.60	1.93	27.71	0.25	104.46
Wang et al., (2016)[19]	Northen Tibet (E)	5123-2	34.43	89.13	65.04	1.11	4.78	3.65	226	498	22.43	152.80	1.56	22.18	0.45	98.01
Wang et al., (2016)[19]	Northen Tibet (E)	5124-1	34.42	89.19	65.39	0.98	5.10	3.22	250	439	22.78	182.50	1.53	19.29	0.57	119.28
Wang et al., (2016)[19]	Northen Tibet (E)	5124-2	34.42	89.19	65.54	1.07	4.89	3.41	242	528	23.86	182.30	1.60	22.14	0.46	113.72
Wang et al., (2016)[19]	Northen Tibet (E)	5126-1	34.40	89.24	65.64	1.01	4.99	3.74	275	506	23.77	198.50	1.63	21.30	0.54	121.63
Wang et al., (2016)[19]	Northen Tibet (E)	5127-3	34.39	89.26	65.73	0.97	5.13	3.41	240	445	23.48	182.60	1.56	18.96	0.54	116.75
Wang et al., (2016)[19]	Northen Tibet (E)	5133-1	34.39	89.28	65.67	1.04	5.16	3.31	252	495	22.87	199.10	1.51	21.64	0.51	131.51
Wang et al., (2016)[19]	Northen Tibet (E)	5125-1	34.41	89.21	66.05	1.00	5.14	3.17	243	474	18.20	144.50	1.29	26.03	0.51	112.19
Wang et al., (2016)[19]	Northen Tibet (E)	5127-1	34.39	89.26	66.25	1.01	5.26	3.18	250	442	18.88	150.70	1.37	23.43	0.57	109.84
Wang et al., (2016)[19]	Northen Tibet (E)	11WL59-2	34.53	90.55	62.98	1.37	4.52	3.47	172	639	24.26	150.00	1.59	26.34	0.27	94.40
Wang et al., (2016)[19]	Northen Tibet (E)	11WL59-3	34.53	90.55	63.07	1.47	4.62	3.51	170	633	22.14	137.90	1.43	28.57	0.27	96.43
Wang et al., (2016)[19]	Northen Tibet (E)	11WL59-4	34.53	90.55	63.02	1.58	4.58	3.50	179	656	25.21	147.80	1.51	26.01	0.27	97.75
Wang et al., (2016)[19]	Northen Tibet (E)	11WL61-3	34.54	90.55	63.26	1.41	4.63	3.51	173	648	22.00	131.20	1.47	29.47	0.27	89.01
Wang et al., (2016)[19]	Northen Tibet (E)	11WL61-4	34.54	90.55	63.00	1.46	4.65	3.52	173	643	22.38	136.20	1.39	28.74	0.27	98.34
Wang et al., (2016)[19]	Northen Tibet (E)	11WL60-3	34.54	90.55	63.10	1.51	4.63	3.51	174	658	23.62	144.00	1.48	27.86	0.26	97.30
Wang et al., (2016)[19]	Northen Tibet (E)	MB1-1	33.65	90.31	63.56	1.39	4.42	3.60	194	550	23.60	156.00	2.05	23.31	0.35	76.10
Wang et al., (2016)[19]	Northen Tibet (E)	MB1-4	33.65	90.31	63.44	1.30	4.48	3.43	199	560	22.20	136.00	2.01	25.23	0.36	67.66
Wang et al., (2016)[19]	Northen Tibet (E)	MB2	33.65	90.31	63.58	1.43	4.52	3.65	205	550	23.00	144.00	2.09	23.91	0.37	68.90
Wang et al., (2016)[19]	Northen Tibet (E)	MB4	33.65	90.31	63.16	1.34	4.55	3.54	192	620	20.90	138.00	1.81	29.67	0.31	76.24
Wang et al., (2016)[19]	Northen Tibet (E)	C1601-1	33.65	90.31	65.73	0.83	4.82	3.36	251	400	25.20	122.00	2.51	15.87	0.63	48.61
Wang et al., (2016)[19]	Northen Tibet (E)	C1603-1h	33.65	90.31	65.20	1.11	4.64	3.32	238	520	25.80	142.00	2.38	20.16	0.46	59.66
Wang et al., (2016)[19]	Northen Tibet (E)	2303a	35.75	90.65	70.67	0.51	5.42	3.28	418	113	6.75	48.49	0.58	16.75	3.70	83.46
Wang et al., (2016)[19]	Northen Tibet (M)	10-1	36.69	86.07	60.62	2.20	4.01	2.92	159	821	20.64	154.30	1.32	39.78	0.19	116.89
Wang et al., (2016)[19]	Northen Tibet (M)	43-1	36.68	86.08	60.82	1.95	4.12	3.03	136	856	22.49	162.30	1.40	38.06	0.16	115.93
Wang et al., (2016)[19]	Northen Tibet (M)	46-2	36.65	86.08	60.17	2.10	4.05	3.05	166	882	23.63	179.10	1.45	37.33	0.19	123.52
Wang et al., (2016)[19]	Northen Tibet (M)	40	36.69	86.04	60.54	2.02	4.16	2.99	170	890	23.02	178.80	1.43	38.66	0.19	125.03
Wang et al., (2016)[19]	Northen Tibet (M)	2	36.71	86.05	60.56	1.99	4.15	3.12	174	874	22.70	172.10	1.40	38.50	0.20	122.93

Note: W - west part; M - middle part; E - east part.

Table S3. Data subsets used to plot correlation between Moho depth and Sr/Y.

Location	Moho(km)	uncertainty	Average Rb/Sr(*)	Median Sr/Y(*)	std(*)	Average Rb/Sr(**)	Median Sr/Y(**)	std(**)
North Eastern Anatolia (W)	43.12	1.22	0.20	21.77	9.60	0.17	22.64	8.06
North Eastern Anatolia (E)	47.21	2.99	0.15	16.92	1.84	0.14	16.92	1.83
Northeastern Central Anatolia	42.65	1.02	0.30	15.70	0.49	0.30	15.70	0.49
South Central Anatolia (W)	36.24	0.44	0.32	16.42	1.94	0.32	16.42	1.94
South Central Anatolia (E)	36.61	0.71	0.32	11.64	1.84	0.32	11.64	1.84
Eastern Carpathian	37.67	2.09	0.21	13.00	3.63	0.21	13.00	3.63
Greater Caucasus (E)	45.14	1.80	0.08	25.68	5.06	0.08	25.68	5.06
Northern Lesser Caucasus	44.49	1.58	0.09	24.66	9.16	0.09	24.66	9.16
Northwestern Lesser Caucasus	43.80	2.03	0.06	23.96	9.64	0.06	23.96	9.64
Central Lesser Caucasus	47.55	2.17				0.24	26.43	8.65
Southeastern Tibet	39.48	2.25	0.34	17.36	4.04			
Northen Tibet (W)	52.20	2.59	0.17	35.02	9.21	0.19	38.50	0.90
Northen Tibet (M)	52.38	1.25	0.19	38.50	0.90			
North Eastern Anatolia (M)	42.98	1.40	0.42	6.42	4.37	0.44	10.45	4.41
Southwestern Central Anatolia	37.39	0.61	0.13	39.77	13.27	0.13	39.77	13.27
Northwestern Iran	44.62	0.87	0.09	61.31	14.68			
Northern Iran	42.91	2.78	0.09	108.06	19.20			
Greater Caucasus (W)	43.48	0.47	0.52	20.41	15.72	0.54	19.65	14.21
Southeastern Lesser Caucasus	46.66	2.00	0.05	50.47	12.16	0.04	48.26	11.90
Central Lesser Caucasus	47.55	2.17	0.20	27.29	12.08			
Southeastern Tibet	39.48	2.25				0.38	16.71	4.34
Northen Tibet (E)	66.92	1.83	0.57	23.43	4.09	0.57	23.67	4.03

Note: W - west part; M - middle part; E - east part; std - standard deviation.

The subsets with value in red are discarded due to their higher value of Rb/Sr or std.

* represents value calculated from all samples.

** represents value calculated from sub-alkaline samples.

Table S4. Data used to calculate La/Ybn for global young rock data subsets.

Source	Location	Sample	Latitude	Longitude	SiO2(wt.%)	MgO(wt.%)	Na2O(wt.%)	K2O(wt.%)	Rb(ppm)	Sr(ppm)	Y(ppm)	La(ppm)	Yb(ppm)	Th(ppm)	Rb/Sr	Sr/Y	La/Ybn
GEOROC[1]	North Eastern Anatolia (W)	MK251	40.06	41.36	65.10	2.17	4.21	2.35	59	454	14.00	33.10	1.04	8.35	0.13	32.43	21.62
GEOROC[1]	North Eastern Anatolia (W)	MK49	40.06	41.36	66.30	1.89	3.88	2.85	83	318	16.00	33.90	1.36	11.47	0.26	19.88	16.93
GEOROC[1]	North Eastern Anatolia (W)	MK93	40.06	41.36	63.50	2.43	3.58	2.76	72	443	15.00	38.50	1.35	9.94	0.16	29.53	19.37
GEOROC[1]	North Eastern Anatolia (W)	MK265	40.12	41.58	63.90	3.65	3.53	2.73	70	453	16.00	31.40	1.14	8.04	0.15	28.31	18.71
GEOROC[1]	North Eastern Anatolia (W)	MK277	40.12	41.58	60.60	4.08	4.11	2.18	62	489	16.00	28.10	1.33	7.50	0.13	30.56	14.35
GEOROC[1]	North Eastern Anatolia (W)	MK268	40.12	41.58	65.50	2.03	3.92	3.27	91	350	15.00	29.20	1.37	10.59	0.26	23.33	14.48
GEOROC[1]	North Eastern Anatolia (W)	MK281	40.12	41.58	57.00	2.23	4.68	2.00	52	417	31.00	24.70	2.67	6.55	0.12	13.45	6.28
GEOROC[1]	North Eastern Anatolia (W)	MK101	39.98	41.68	57.00	4.56	3.48	2.00	54	434	21.00	30.20	2.22	9.88	0.12	20.67	9.24
GEOROC[1]	North Eastern Anatolia (W)	MK112	39.98	41.68	66.80	1.24	3.68	3.26	78	350	18.00	31.60	1.55	9.51	0.22	19.44	13.85
GEOROC[1]	North Eastern Anatolia (W)	MK117	39.98	41.68	62.10	1.99	4.03	2.34	59	369	21.00	20.00	2.00	6.67	0.16	17.57	6.79
GEOROC[1]	North Eastern Anatolia (W)	2	39.07	41.37	56.90	2.88	4.53	1.75	46	425	34.20	28.00	2.50	5.20	0.11	12.43	7.61
GEOROC[1]	North Eastern Anatolia (W)	1	39.07	41.37	56.35	3.36	4.40	1.95	54	492	31.60	44.00	3.00	7.50	0.11	15.57	9.96
GEOROC[1]	North Eastern Anatolia (M)	YOZ 92	38.62	42.19	60.31	1.28	5.00	3.40	101	329	49.40	47.20	4.86	12.80	0.31	6.67	6.60
GEOROC[1]	North Eastern Anatolia (M)	N-136	38.67	42.20	59.82	0.91	6.53	4.35	135	211	54.93	51.99	6.37	17.00	0.64	3.84	5.55
GEOROC[1]	North Eastern Anatolia (M)	N-036	38.67	42.20	60.25	0.96	4.81	3.72	109	180	58.76	50.36	6.23	13.50	0.61	3.06	5.49
GEOROC[1]	North Eastern Anatolia (M)	N-037	38.67	42.20	61.55	0.56	6.05	4.53	94	138	33.77	34.33	3.80	10.02	0.68	4.08	6.15
GEOROC[1]	North Eastern Anatolia (M)	N-013	38.67	42.20	56.44	3.97	4.52	2.17	84	244	60.62	37.19	6.39	11.94	0.34	4.03	3.95
GEOROC[1]	North Eastern Anatolia (M)	N-050	38.67	42.20	57.12	2.26	5.30	2.02	44	337	41.16	28.01	3.96	5.63	0.13	8.20	4.81
GEOROC[1]	North Eastern Anatolia (M)	N-256	38.67	42.20	59.51	3.45	4.53	2.44	100	203	67.06	42.35	7.08	14.00	0.49	3.02	4.06
GEOROC[1]	North Eastern Anatolia (M)	N-272	38.67	42.20	60.63	0.63	5.77	3.70	53	186	34.70	26.95	3.57	6.02	0.29	5.37	5.13
GEOROC[1]	North Eastern Anatolia (M)	N-014	38.67	42.20	67.35	1.62	4.86	3.56	158	100	90.73	61.08	9.57	21.60	1.57	1.11	4.33
GEOROC[1]	North Eastern Anatolia (M)	NR-14	38.69	42.22	64.23	2.44	4.92	3.10	123	164	70.40	49.50	8.80	17.10	0.75	2.33	3.82
GEOROC[1]	North Eastern Anatolia (M)	NR-11	38.68	42.23	56.33	4.61	4.31	1.95	71	269	52.10	34.10	6.40	10.30	0.27	5.16	3.62
GEOROC[1]	North Eastern Anatolia (M)	NR-24	38.56	42.24	61.40	0.99	5.22	3.56	98	217	47.00	44.90	5.60	12.30	0.45	4.62	5.45
GEOROC[1]	North Eastern Anatolia (M)	CU-1	38.67	42.27	61.16	0.73	5.28	3.59	95	236	54.40	42.00	5.50	11.80	0.40	4.33	5.19
GEOROC[1]	North Eastern Anatolia (M)	Z-12	38.55	42.35	58.58	1.72	5.40	3.75	91	340	55.10	48.10	5.94	12.70	0.27	6.17	5.50
GEOROC[1]	North Eastern Anatolia (M)	MK159	40.18	42.82	60.30	1.83	4.12	2.05	55	286	32.00	23.60	2.97	8.63	0.19	8.94	5.40
GEOROC[1]	North Eastern Anatolia (M)	MK158	40.18	42.82	61.70	1.41	4.52	2.79	71	368	35.00	34.20	3.41	10.76	0.19	10.51	6.81
GEOROC[1]	North Eastern Anatolia (M)	MK162	40.16	43.14	55.30	4.79	3.47	1.26	30	420	23.00	20.20	1.90	4.55	0.07	18.26	7.22
GEOROC[1]	North Eastern Anatolia (M)	MK174	40.16	43.14	66.50	0.81	4.70	3.16	77	236	37.00	28.40	2.87	10.57	0.33	6.38	6.72
GEOROC[1]	North Eastern Anatolia (E)	TE01	39.20	44.00	58.53	1.45	6.90	3.71	99	291	55.00	54.50	5.86	13.20	0.34	5.29	6.32
GEOROC[1]	North Eastern Anatolia (E)	AR01	39.75	44.40	63.83	1.64	5.03	1.85	41	389	23.00	26.00	2.06	6.20	0.11	16.91	8.57
GEOROC[1]	North Eastern Anatolia (E)	AR02	39.75	44.40	62.64	2.33	4.92	2.15	56	381	26.00	27.50	2.48	7.60	0.15	14.65	7.53
GEOROC[1]	North Eastern Anatolia (E)	AR11	39.75	44.40	58.73	3.36	4.41	1.81	37	446	33.00	24.40	2.91	5.40	0.08	13.52	5.70
GEOROC[1]	Northeastern Central Anatolia	M22	39.40	38.00	55.07	4.85	3.70	1.20	33	352	25.70	19.30	2.60	6.60	0.01	13.70	5.04
GEOROC[1]	Northeastern Central Anatolia	IL2	39.60	39.50	56.70	2.63	4.65	3.21	59	504	27.90	41.30	2.77	6.30	0.12	18.06	10.13
GEOROC[1]	Northeastern Central Anatolia	IL3	39.60	39.50	56.77	2.65	4.65	2.66	56	542	27.20	41.90	2.67	10.50	0.10	19.91	10.66
GEOROC[1]	Northeastern Central Anatolia	IL6	39.60	39.50	56.80	2.70	4.80	2.81	51	525	26.10	37.50	2.68	7.30	0.10	20.13	9.51
GEOROC[1]	Northeastern Central Anatolia	KT3	39.60	39.50	62.60	2.70	3.98	3.05	74	341	17.90	33.40	1.68	13.00	0.22	19.06	13.51
GEOROC[1]	Northeastern Central Anatolia	KT4	39.60	39.50	62.80	2.22	3.91	3.11	95	294	19.20	37.20	1.52	19.20	0.32	15.31	16.63
GEOROC[1]	Northeastern Central Anatolia	YT2	39.60	39.50	62.69	2.44	3.83	3.23	83	301	18.20	35.00	1.78	14.20	0.28	16.53	13.36

Source	Location	Sample	Latitude	Longitude	SiO2(wt.%)	MgO(wt.%)	Na2O(wt.%)	K2O(wt.%)	Rb(ppm)	Sr(ppm)	Y(ppm)	La(ppm)	Yb(ppm)	Th(ppm)	Rb/Sr	Sr/Y	La/Ybn
GEOROC[1]	Northeastern Central Anatolia	IR2	39.60	39.50	62.99	2.33	3.89	2.93	91	268	19.00	37.40	1.56	15.00	0.34	14.13	16.29
GEOROC[1]	Northeastern Central Anatolia	IR3	39.60	39.50	62.90	2.34	3.96	2.88	91	282	18.10	35.30	1.40	15.50	0.32	15.55	17.13
GEOROC[1]	Northeastern Central Anatolia	IR5	39.60	39.50	62.79	2.35	3.95	3.09	80	273	17.40	32.90	1.61	13.30	0.29	15.70	13.88
GEOROC[1]	Northeastern Central Anatolia	IR8	39.60	39.50	62.46	2.41	4.02	3.09	83	281	18.90	33.50	1.68	13.00	0.30	14.85	13.55
GEOROC[1]	Northeastern Central Anatolia	IR9	39.60	39.50	62.96	2.30	4.02	3.09	83	288	17.80	34.60	1.69	14.60	0.29	16.16	13.91
GEOROC[1]	Northeastern Central Anatolia	IR10	39.60	39.50	62.81	2.33	3.95	3.01	84	288	17.80	33.70	1.90	14.70	0.29	16.17	12.05
GEOROC[1]	Northeastern Central Anatolia	IR4A	39.60	39.50	63.70	2.26	3.84	3.24	89	274	17.00	34.60	1.60	16.00	0.32	16.14	14.69
GEOROC[1]	Northeastern Central Anatolia	IR7	39.60	39.50	63.60	2.41	4.00	3.04	85	294	18.50	35.20	1.90	14.20	0.29	15.90	12.59
GEOROC[1]	Northeastern Central Anatolia	IR11	39.60	39.50	63.20	2.32	4.01	3.00	81	281	17.90	34.00	1.74	13.70	0.29	15.68	13.27
GEOROC[1]	Northeastern Central Anatolia	YT3	39.60	39.50	63.90	1.87	4.11	3.20	89	276	18.00	36.70	1.82	15.20	0.32	15.33	13.70
GEOROC[1]	Northeastern Central Anatolia	PT1	39.60	39.50	69.40	0.58	4.02	3.87	113	262	12.70	35.70	1.00	21.60	0.43	20.66	24.25
GEOROC[1]	Northeastern Central Anatolia	AT2	39.60	39.50	69.77	0.56	4.17	3.87	97	283	9.50	36.20	0.98	20.20	0.34	29.77	25.09
GEOROC[1]	Northeastern Central Anatolia	AT3	39.60	39.50	70.76	0.55	4.10	3.52	93	265	9.10	31.40	0.91	15.00	0.35	29.12	23.44
GEOROC[1]	Northeastern Central Anatolia	T1	39.60	39.50	70.74	0.57	4.03	3.59	109	283	9.70	34.00	0.89	17.70	0.39	29.13	25.95
GEOROC[1]	Northeastern Central Anatolia	T3	39.60	39.50	70.69	0.57	4.04	3.60	111	291	9.90	35.20	0.89	19.90	0.38	29.41	26.87
GEOROC[1]	Northeastern Central Anatolia	PT2	39.60	39.50	70.56	0.59	3.98	3.70	105	277	11.50	34.00	1.11	18.30	0.38	24.09	20.81
GEOROC[1]	South Central Anatolia (W)	90-62	38.13	34.18	67.19	1.18	3.86	2.94	96	243	18.00	30.50	1.43		0.40	13.50	14.49
GEOROC[1]	South Central Anatolia (W)	90-5	38.13	34.18	60.30	3.13	3.60	1.90	58	443	18.00	24.80	1.78	7.00	0.13	24.61	9.46
GEOROC[1]	South Central Anatolia (W)	90-36	38.13	34.18	64.04	2.75	3.83	1.97	66	350	21.00	31.30	2.03	9.00	0.19	16.67	10.47
GEOROC[1]	South Central Anatolia (W)	C94-15 (1)	37.98	34.25	55.75	3.73	3.50	2.12	41	473	18.13	28.30	2.08	10.88	0.09	26.07	9.24
GEOROC[1]	South Central Anatolia (W)	K102P	38.12	34.28	67.80	1.13	4.03	3.01	107	270	14.40	28.60	1.43	14.00	0.40	18.75	13.59
GEOROC[1]	South Central Anatolia (W)	K-84	38.11	34.29	71.80	0.66	4.40	3.17	93	229	14.10	29.30	1.42	14.50	0.41	16.24	14.02
GEOROC[1]	South Central Anatolia (W)	K110	38.11	34.30	70.70	0.64	4.12	3.26	107	221	13.90	28.20	1.51	14.20	0.48	15.90	12.69
GEOROC[1]	South Central Anatolia (W)	KEC-2	38.18	34.42	69.50	0.77	3.77	3.45	112	217	13.70	24.20	1.42	13.60	0.52	15.84	11.58
GEOROC[1]	South Central Anatolia (W)	C94-31	38.42	34.63	57.64	3.51	3.25	1.72	33	325	16.83	24.12	1.93	6.68	0.10	19.28	8.49
GEOROC[1]	South Central Anatolia (E)	E-188	38.46	35.23	60.40	3.08	3.95	1.83	63	378	18.40	19.46	1.80	10.20	0.17	20.53	7.34
GEOROC[1]	South Central Anatolia (E)	E-185	38.49	35.26	60.00	3.15	3.61	1.69	46	352	18.20	20.64	1.88	6.60	0.13	19.36	7.46
GEOROC[1]	South Central Anatolia (E)	ERC91-29	38.53	35.45	57.11	3.66	4.21	1.34	30	422	29.00	27.90	2.58		0.07	14.54	7.35
GEOROC[1]	South Central Anatolia (E)	ERC95-83	38.53	35.45	67.06	1.69	3.68	2.27	83	258	23.80	20.30	1.84		0.32	10.82	7.49
GEOROC[1]	South Central Anatolia (E)	ERC91-21	38.53	35.45	65.84	1.98	3.95	2.09	85	295	27.30	25.90	2.10		0.29	10.81	8.38
GEOROC[1]	South Central Anatolia (E)	C2	38.53	35.45	55.83	5.23	3.52	1.21	29	494	22.10	24.20	2.22	5.70	0.06	22.36	7.41
GEOROC[1]	South Central Anatolia (E)	C3C	38.53	35.45	55.54	5.29	3.46	1.17	29	512	23.20	24.40	2.26	4.70	0.06	22.07	7.33
GEOROC[1]	South Central Anatolia (E)	C6	38.53	35.45	55.30	5.07	3.41	1.19	30	502	22.10	23.70	2.07	5.80	0.06	22.72	7.78
GEOROC[1]	South Central Anatolia (E)	C21	38.53	35.45	55.27	5.02	3.43	1.18	27	515	23.00	24.80	2.11	5.80	0.05	22.41	7.98
GEOROC[1]	South Central Anatolia (E)	C23	38.53	35.45	55.68	4.95	3.63	1.18	28	494	21.20	23.80	1.95	5.00	0.06	23.29	8.29
GEOROC[1]	South Central Anatolia (E)	2C13	38.53	35.45	55.64	4.91	3.48	1.17	31	510	22.80	24.60	2.03	5.50	0.06	22.35	8.23
GEOROC[1]	South Central Anatolia (E)	C11C	38.53	35.45	55.81	4.96	3.52	1.18	27	498	21.90	23.30	1.90	4.40	0.05	22.75	8.33
GEOROC[1]	South Central Anatolia (E)	C22	38.53	35.45	55.39	5.06	3.48	1.18	28	525	21.30	23.60	2.04	5.30	0.05	24.63	7.86
GEOROC[1]	South Central Anatolia (E)	C14	38.53	35.45	55.45	5.04	3.44	1.15	27	508	21.60	23.30	2.19	3.60	0.05	23.51	7.23
GEOROC[1]	South Central Anatolia (E)	2C1	38.53	35.45	55.45	5.26	3.51	1.19	28	501	21.40	24.00	2.05	6.70	0.06	23.42	7.95
GEOROC[1]	South Central Anatolia (E)	2C6	38.53	35.45	55.66	5.06	3.48	1.19	29	508	22.20	24.20	2.04	4.60	0.06	22.87	8.06
GEOROC[1]	South Central Anatolia (E)	A2	38.53	35.45	60.48	3.08	3.87	1.89	51	348	25.30	25.10	2.54	9.10	0.15	13.77	6.71
GEOROC[1]	South Central Anatolia (E)	A4	38.53	35.45	61.42	3.30	3.71	1.83	54	327	22.70	24.20	2.32	9.10	0.16	14.42	7.09

Source	Location	Sample	Latitude	Longitude	SiO2(wt.%)	MgO(wt.%)	Na2O(wt.%)	K2O(wt.%)	Rb(ppm)	Sr(ppm)	Y(ppm)	La(ppm)	Yb(ppm)	Th(ppm)	Rb/Sr	Sr/Y	La/Ybn
GEOROC[1]	South Central Anatolia (E)	CM2G	38.53	35.45	64.35	2.40	3.86	2.22	77	321	23.50	29.30	2.35	13.20	0.24	13.68	8.47
GEOROC[1]	South Central Anatolia (E)	CM8	38.53	35.45	64.91	2.29	3.91	2.26	81	308	22.70	28.50	2.21	13.00	0.26	13.55	8.76
GEOROC[1]	South Central Anatolia (E)	CM1E	38.53	35.45	64.75	2.41	3.90	2.24	77	298	22.60	26.70	2.07	11.90	0.26	13.17	8.76
GEOROC[1]	South Central Anatolia (E)	CM5	38.53	35.45	61.92	2.60	3.82	1.99	67	329	25.10	27.30	2.27	10.20	0.20	13.11	8.17
GEOROC[1]	South Central Anatolia (E)	CM5K	38.53	35.45	63.61	2.42	3.82	2.13	76	304	22.70	26.80	2.27	12.30	0.25	13.40	8.02
GEOROC[1]	South Central Anatolia (E)	ERC98-3	38.53	35.45	62.52	3.37	3.83	1.76	59	291	16.20	19.50	1.83	10.70	0.20	17.96	7.24
GEOROC[1]	South Central Anatolia (E)	ERC96-71	38.53	35.45	64.72	2.02	4.54	2.03	69	265	20.40	26.20	2.21	12.20	0.26	12.97	8.05
GEOROC[1]	South Central Anatolia (E)	ERC92-3	38.53	35.45	70.10	0.92	3.90	2.96	147	195	23.80	28.50	2.10		0.75	8.17	9.22
GEOROC[1]	South Central Anatolia (E)	ER11	38.53	35.45	62.02	2.93	3.65	1.63	45	324	22.00	23.80	2.15	6.60	0.14	14.73	7.52
GEOROC[1]	South Central Anatolia (E)	ER13	38.53	35.45	66.44	1.40	4.54	2.99	106	229	27.00	30.10	2.70	16.80	0.46	8.48	7.57
GEOROC[1]	South Central Anatolia (E)	ER14	38.53	35.45	63.76	2.24	4.17	2.45	84	286	27.00	30.20	2.67	13.70	0.29	10.59	7.68
GEOROC[1]	South Central Anatolia (E)	ER15	38.53	35.45	68.97	1.15	3.96	2.79	106	217	21.00	27.40	2.14	14.70	0.49	10.33	8.70
GEOROC[1]	South Central Anatolia (E)	ER16	38.53	35.45	69.14	0.78	3.89	2.85	117	207	21.00	30.00	2.18	17.80	0.57	9.86	9.35
GEOROC[1]	South Central Anatolia (E)	ER17	38.53	35.45	64.24	2.08	3.77	2.36	81	290	27.00	36.10	2.51	14.10	0.28	10.74	9.77
GEOROC[1]	South Central Anatolia (E)	ER20	38.53	35.45	65.69	2.19	3.75	2.32	79	281	25.00	33.20	2.37	12.40	0.28	11.24	9.52
GEOROC[1]	South Central Anatolia (E)	ER21	38.53	35.45	63.18	2.62	3.84	1.81	64	318	23.00	24.90	2.23	10.50	0.20	13.83	7.59
GEOROC[1]	South Central Anatolia (E)	ER23	38.53	35.45	63.48	2.91	3.49	2.23	70	315	22.00	23.50	1.76	11.00	0.22	14.32	9.07
GEOROC[1]	South Central Anatolia (E)	ER25	38.53	35.45	64.33	2.43	3.87	1.78	48	303	21.00	23.20	1.95	8.50	0.16	14.43	8.08
GEOROC[1]	South Central Anatolia (E)	ER26	38.53	35.45	57.90	3.90	3.96	1.50	26	394	27.00	27.80	2.71	4.70	0.07	14.59	6.97
GEOROC[1]	South Central Anatolia (E)	IEY-001	38.53	35.45	68.92	1.58	3.83	1.93	89	273	19.90	23.40	1.86	11.50	0.33	13.72	8.55
GEOROC[1]	South Central Anatolia (E)	IEY-003	38.53	35.45	67.93	1.75	3.79	2.22	98	269	23.40	26.90	2.26	12.70	0.36	11.50	8.09
GEOROC[1]	South Central Anatolia (E)	IEY-005	38.53	35.45	69.10	1.77	3.80	2.16	93	262	22.10	27.60	2.00	12.50	0.35	11.86	9.37
GEOROC[1]	South Central Anatolia (E)	IEY-0017	38.53	35.45	67.54	1.91	3.82	2.08	90	256	22.10	26.20	2.21	10.60	0.35	11.58	8.05
GEOROC[1]	South Central Anatolia (E)	IY-033	38.53	35.45	67.50	1.77	3.96	2.34	84	258	22.20	25.00	2.01	12.20	0.33	11.62	8.45
GEOROC[1]	South Central Anatolia (E)	IY-0317	38.53	35.45	67.50	1.82	3.82	2.42	83	256	22.80	25.00	1.99	12.20	0.32	11.23	8.53
GEOROC[1]	South Central Anatolia (E)	EY-005	38.53	35.45	66.96	2.50	3.64	2.53	74	313	16.80	23.80	2.15	12.80	0.24	18.63	7.52
GEOROC[1]	South Central Anatolia (E)	EY-0012	38.53	35.45	65.48	2.15	4.21	2.31	72	323	21.20	29.70	2.18	11.70	0.22	15.24	9.26
GEOROC[1]	South Central Anatolia (E)	EY-0016	38.53	35.45	65.96	2.43	3.81	2.56	87	248	22.00	28.40	2.31	13.20	0.35	11.27	8.35
GEOROC[1]	South Central Anatolia (E)	IEY-00X6	38.53	35.45	59.57	3.92	3.29	1.53	49	438	21.40	27.50	1.84	7.30	0.11	20.47	10.15
GEOROC[1]	South Central Anatolia (E)	IEY-00X16	38.53	35.45	62.74	2.80	3.65	1.76	60	342	20.00	25.30	2.04	9.70	0.18	17.10	8.42
GEOROC[1]	South Central Anatolia (E)	IEY-007	38.53	35.45	66.85	2.67	3.42	2.91	99	245	19.30	25.00	2.03	14.20	0.40	12.69	8.37
GEOROC[1]	South Central Anatolia (E)	IEY-009	38.53	35.45	69.60	1.65	3.74	2.94	89	264	22.40	28.40	2.23	14.60	0.34	11.79	8.65
GEOROC[1]	South Central Anatolia (E)	IEY-0013	38.53	35.45	66.53	2.05	3.74	2.22	86	269	23.50	26.70	2.32	11.10	0.32	11.45	7.82
GEOROC[1]	South Central Anatolia (E)	IEY-00X2	38.53	35.45	65.77	2.69	3.34	2.39	81	300	21.00	28.90	2.02	13.50	0.27	14.29	9.72
GEOROC[1]	South Central Anatolia (E)	IEY-00X4	38.53	35.45	65.23	2.71	3.31	1.99	71	316	21.00	30.00	2.01	14.10	0.22	15.05	10.14
GEOROC[1]	South Central Anatolia (E)	IEY-00X8	38.53	35.45	65.83	3.10	3.25	2.05	70	313	18.00	24.10	1.92	11.50	0.22	17.39	8.53
GEOROC[1]	South Central Anatolia (E)	IEY-00X12	38.53	35.45	66.66	2.04	3.73	2.14	89	280	24.50	27.40	2.40	12.70	0.32	11.43	7.76
GEOROC[1]	South Central Anatolia (E)	IEY-00X14	38.53	35.45	66.70	2.23	3.68	1.97	81	280	23.20	26.30	2.21	10.50	0.29	12.07	8.08
GEOROC[1]	South Central Anatolia (E)	IEY-00X18	38.53	35.45	68.65	1.27	3.71	2.99	126	232	25.20	30.80	2.56	17.30	0.54	9.21	8.17
GEOROC[1]	South Central Anatolia (E)	IEY-00X20	38.53	35.45	68.85	1.47	3.68	2.74	85	257	20.40	27.90	2.03	12.70	0.33	12.60	9.34
GEOROC[1]	South Central Anatolia (E)	IEY-00X22	38.53	35.45	69.90	1.26	3.73	3.05	94	230	20.60	30.20	2.09	14.30	0.41	11.17	9.82
GEOROC[1]	South Central Anatolia (E)	IEY-00X24	38.53	35.45	70.00	1.50	3.48	2.48	86	229	20.00	28.50	2.06	14.10	0.38	11.45	9.40
GEOROC[1]	South Central Anatolia (E)	IEY-00X26	38.53	35.45	68.43	1.75	3.58	2.40	86	250	21.70	27.50	2.22	12.00	0.34	11.52	8.42
GEOROC[1]	South Central Anatolia (E)	IEY-00X30	38.53	35.45	69.12	1.50	3.67	2.92	99	254	22.20	29.50	2.22	14.30	0.39	11.44	9.03
GEOROC[1]	South Central Anatolia (E)	IEY-0015A	38.53	35.45	70.51	1.03	3.84	3.10	114	213	21.40	29.50	2.28	16.40	0.54	9.95	8.79

Source	Location	Sample	Latitude	Longitude	SiO2(wt.%)	MgO(wt.%)	Na2O(wt.%)	K2O(wt.%)	Rb(ppm)	Sr(ppm)	Y(ppm)	La(ppm)	Yb(ppm)	Th(ppm)	Rb/Sr	Sr/Y	La/Ybn
GEOROC[1]	South Central Anatolia (E)	IEY-0015B	38.53	35.45	70.62	1.02	3.85	3.11	116	225	21.70	30.80	2.11	18.30	0.52	10.37	9.92
GEOROC[1]	South Central Anatolia (E)	IEY-00X28	38.53	35.45	70.94	0.75	3.83	3.03	57	113	10.10	15.50	1.05	8.10	0.50	11.19	10.03
GEOROC[1]	South Central Anatolia (E)	OU-034	38.53	35.45	55.19	5.76	3.41	1.18	23	467	22.00	23.20	2.08	3.90	0.05	21.23	7.58
GEOROC[1]	South Central Anatolia (E)	OU-0313A	38.53	35.45	55.81	5.38	3.68	1.04	21	452	20.40	23.30	1.78	3.80	0.05	22.16	8.89
GEOROC[1]	South Central Anatolia (E)	OU-0321B	38.53	35.45	55.52	4.63	4.02	1.48	33	517	27.00	34.80	2.54	6.30	0.06	19.15	9.31
GEOROC[1]	South Central Anatolia (E)	OU-0322-7	38.53	35.45	55.01	5.14	3.86	1.38	26	503	27.30	30.20	2.43	4.70	0.05	18.42	8.44
GEOROC[1]	South Central Anatolia (E)	OU-0322-8	38.53	35.45	55.43	4.58	3.95	1.36	25	476	25.80	28.80	2.30	3.50	0.05	18.45	8.51
GEOROC[1]	South Central Anatolia (E)	OU-0319	38.53	35.45	67.82	2.10	3.89	1.82	56	265	20.20	23.70	1.93	8.50	0.21	13.12	8.34
GEOROC[1]	South Central Anatolia (E)	OU-0322-5B	38.53	35.45	65.28	2.46	3.90	1.82	57	300	22.40	25.60	2.03	8.70	0.19	13.39	8.57
GEOROC[1]	South Central Anatolia (E)	OU-0322-6	38.53	35.45	65.88	2.42	3.83	1.85	58	284	20.90	23.00	1.93	8.90	0.20	13.59	8.10
GEOROC[1]	South Central Anatolia (E)	OU-0322-9	38.53	35.45	65.46	2.17	3.82	2.54	79	295	20.80	29.60	2.18	16.10	0.27	14.18	9.22
GEOROC[1]	South Central Anatolia (E)	OU-0326-1	38.53	35.45	67.03	1.74	3.89	2.27	81	265	21.10	24.80	1.93	11.90	0.31	12.56	8.73
GEOROC[1]	South Central Anatolia (E)	OU-0328B	38.53	35.45	66.95	1.88	3.90	2.30	81	265	20.10	21.30	1.97	11.30	0.31	13.18	7.34
GEOROC[1]	South Central Anatolia (E)	E-103	38.55	35.54	62.19	2.04	3.77	2.94	98	300	32.20	29.41	2.70	14.89	0.33	9.33	7.40
GEOROC[1]	South Central Anatolia (E)	E-86	38.67	35.55	64.08	1.91	3.85	2.03	65	316	19.60	23.36	1.98	10.71	0.21	16.10	8.01
GEOROC[1]	South Central Anatolia (E)	E-108	38.56	35.55	58.71	3.46	3.43	1.96	61	376	20.70	22.93	1.91	9.64	0.16	18.15	8.16
GEOROC[1]	South Central Anatolia (E)	E-88	38.66	35.56	62.19	2.01	3.74	1.98	69	295	20.20	22.63	1.99	10.82	0.24	14.62	7.73
GEOROC[1]	South Central Anatolia (E)	E-133	38.43	35.58	57.31	4.34	3.36	1.41	44	396	18.60	19.86	1.79	7.98	0.11	21.30	7.54
GEOROC[1]	South Central Anatolia (E)	E-169	38.32	35.64	64.08	2.17	3.76	2.47	96	279	21.30	26.72	2.01	14.81	0.34	13.10	9.03
GEOROC[1]	South Central Anatolia (E)	E-141	38.34	35.65	61.39	2.71	4.06	2.07	71	318	19.20	20.97	1.93	11.18	0.22	16.55	7.38
GEOROC[1]	South Central Anatolia (E)	E-102-B	38.57	35.65	58.61	3.12	4.01	2.16	87	330	28.30	33.99	2.51	15.76	0.26	11.66	9.20
GEOROC[1]	South Central Anatolia (E)	ZD03-04	38.60	35.90	66.34	1.78	3.96	2.24	85	261	22.50	23.80	2.14	12.80	0.33	11.60	7.56
GEOROC[1]	South Central Anatolia (E)	ZD03-08A	38.60	35.90	66.44	1.76	3.93	2.34	86	254	22.00	24.10	2.11	11.50	0.34	11.55	7.76
GEOROC[1]	South Central Anatolia (E)	ZD03-08B	38.60	35.90	66.49	1.77	3.93	2.34	86	256	21.80	24.00	2.05	11.70	0.34	11.74	7.95
GEOROC[1]	South Central Anatolia (E)	ZD03-40	38.60	35.90	66.91	1.82	3.87	2.27	83	253	23.00	25.50	2.14	11.70	0.33	11.00	8.09
GEOROC[1]	South Central Anatolia (E)	ZD03-12	38.60	35.90	66.00	1.73	3.94	2.31	87	256	22.30	24.90	1.96	10.70	0.34	11.48	8.63
GEOROC[1]	South Central Anatolia (E)	ZD03-16	38.60	35.90	66.35	1.74	3.92	2.24	85	248	21.20	24.30	2.10	10.30	0.34	11.70	7.86
GEOROC[1]	South Central Anatolia (E)	ZD03-26	38.60	35.90	66.73	1.87	3.91	2.27	86	258	21.80	24.60	1.92	11.30	0.33	11.83	8.70
GEOROC[1]	South Central Anatolia (E)	ZD03-34	38.60	35.90	66.60	1.72	3.93	2.29	88	260	22.20	25.60	2.04	11.60	0.34	11.71	8.52
GEOROC[1]	South Central Anatolia (E)	ZD03-11	38.60	35.90	65.20	1.99	3.76	2.21	79	258	21.90	24.50	2.11	11.70	0.31	11.78	7.89
GEOROC[1]	South Central Anatolia (E)	ZD03-15	38.60	35.90	66.03	2.05	3.86	2.20	77	254	21.50	22.90	2.16	10.50	0.30	11.81	7.20
GEOROC[1]	South Central Anatolia (E)	ZD03-19	38.60	35.90	65.92	2.03	3.82	2.18	84	263	22.60	26.60	2.28	10.70	0.32	11.64	7.93
GEOROC[1]	South Central Anatolia (E)	ZD-03-23	38.60	35.90	66.48	1.84	3.87	2.30	82	261	22.40	24.70	2.09	11.70	0.31	11.65	8.03
GEOROC[1]	South Central Anatolia (E)	ZD03-01	38.60	35.90	66.76	1.56	3.92	2.30	87	251	21.90	25.30	2.13	11.80	0.35	11.46	8.07
GEOROC[1]	South Central Anatolia (E)	ZD03-03	38.60	35.90	66.14	1.69	3.87	2.22	85	264	21.70	23.20	1.99	11.50	0.32	12.17	7.92
GEOROC[1]	South Central Anatolia (E)	ZD03-05	38.60	35.90	66.18	1.68	3.82	2.30	88	250	21.50	23.60	2.11	12.50	0.35	11.63	7.60
GEOROC[1]	Northwestern Iran	BA2	38.55	45.53	57.50	3.50	5.27	2.76	56	1030	16.80	64.90	1.51	17.35	0.05	61.31	29.20
GEOROC[1]	Northwestern Iran	DA14	38.56	45.54	62.40	2.21	5.31	2.87	69	1045	12.70	61.40	1.10	21.60	0.07	82.28	37.92
GEOROC[1]	Northern Iran	DMV12	35.95	52.11	60.68	2.72	4.75	4.42	125	1704	13.00	95.20	1.10		0.07	131.08	58.79
GEOROC[1]	Northern Iran	DMV13	35.95	52.11	62.60	2.15	4.56	4.61	163	1209	13.00	96.10	1.10		0.13	93.00	59.35
GEOROC[1]	Northern Iran	DMV07	35.95	52.11	60.24	2.53	4.82	4.39	126	1621	12.00	95.50	1.10		0.08	135.08	58.98
GEOROC[1]	Northern Iran	DMV39	35.95	52.11	59.39	2.75	4.80	4.43	121	1430	11.00	92.30	1.00		0.08	130.00	62.70
GEOROC[1]	Northern Iran	DA P2	35.95	52.11	59.98	2.61	5.04	5.00	127	1580	12.80	98.00	1.06	27.20	0.08	123.44	62.81

Source	Location	Sample	Latitude	Longitude	SiO2(wt.%)	MgO(wt.%)	Na2O(wt.%)	K2O(wt.%)	Rb(ppm)	Sr(ppm)	Y(ppm)	La(ppm)	Yb(ppm)	Th(ppm)	Rb/Sr	Sr/Y	La/Ybn
GEOROC[1]	Northern Iran	DA4800	35.95	52.11	61.32	2.46	4.86	4.36	131	1370	12.77	91.60	1.04	27.60	0.10	107.28	59.83
GEOROC[1]	Greater Caucasus (W)	20.00	43.35	42.44	68.70	1.46	3.76	4.00	142	317	9.00	48.90	1.40	25.50	0.45	35.22	23.73
GEOROC[1]	Greater Caucasus (W)	27.00	43.35	42.44	67.20	1.49	4.06	2.93	174	51	14.00	48.10	1.60	23.80	3.41	3.64	20.42
GEOROC[1]	Greater Caucasus (W)	43.00	43.35	42.44	67.27	1.06	3.74	3.22	172	604	14.00	55.00	1.70	24.60	0.28	43.14	21.98
GEOROC[1]	Greater Caucasus (W)	32.00	43.35	42.44	67.20	1.54	4.21	3.39	146	389	11.00	46.90	1.50	27.20	0.38	35.36	21.24
GEOROC[1]	Greater Caucasus (W)	45.00	43.35	42.44	66.46	1.05	3.60	3.23	168	256	13.00	54.70	1.60	23.90	0.66	19.69	23.22
GEOROC[1]	Greater Caucasus (W)	48.00	43.35	42.44	66.40	1.44	4.31	3.31	154	278	13.00	56.60	1.80	25.00	0.55	21.38	21.36
GEOROC[1]	Greater Caucasus (W)	22.00	43.35	42.44	67.70	0.82	4.31	3.37	183	378	11.00	40.20	1.30	19.80	0.48	34.36	21.01
GEOROC[1]	Greater Caucasus (W)	23.00	43.35	42.44	67.50	1.45	4.32	3.37	152	207	12.00	43.00	1.50	21.70	0.73	17.25	19.47
GEOROC[1]	Greater Caucasus (W)	7.00	43.35	42.44	66.80	0.82	4.20	3.49	115	318	9.00	36.10	1.20	18.70	0.36	35.33	20.44
GEOROC[1]	Greater Caucasus (W)	6.00	43.35	42.44	66.79	0.92	3.34	3.70	140	295	14.00	40.20	1.40	21.10	0.47	21.07	19.51
GEOROC[1]	Greater Caucasus (W)	396.00	43.35	42.44	66.80	1.40	4.23	3.47	166	320	25.00	46.30	1.31	24.60	0.52	12.80	24.01
GEOROC[1]	Greater Caucasus (W)	338.00	43.35	42.44	67.40	1.57	4.00	3.66	158	332	28.00	48.40	1.43	22.00	0.48	11.86	22.99
GEOROC[1]	Greater Caucasus (W)	10.00	43.35	42.44	67.50	1.86	4.31	3.26	92	342	11.00	40.30	1.52	19.00	0.27	31.09	18.01
GEOROC[1]	Greater Caucasus (W)	9.00	43.35	42.44	67.40	1.44	4.15	4.00	110	370	5.00	47.90	1.44	21.00	0.30	74.00	22.60
GEOROC[1]	Greater Caucasus (W)	340.00	43.35	42.44	64.40	1.64	4.54	3.22	118	308	29.00	39.80	1.55	17.00	0.38	10.62	17.44
GEOROC[1]	Southeastern Lesser Caucasus	120.00	39.86	46.02	55.67	4.66	4.22	2.60	55	730	21.00	52.00	2.00	5.60	0.08	34.76	17.66
GEOROC[1]	Southeastern Lesser Caucasus	180.00	39.86	46.02	55.21	2.50	5.04	3.11	43	1190	27.00	69.00	2.20	7.50	0.04	44.07	21.31
GEOROC[1]	Southeastern Lesser Caucasus	13.00	39.86	46.02	57.66	3.18	3.85	3.01	55	1360	24.00	60.00	1.80	3.60	0.04	56.67	22.64
GEOROC[1]	Southeastern Lesser Caucasus	25.00	39.86	46.02	58.52	3.23	4.00	2.80	49	1275	32.00	60.00	1.90	6.30	0.04	39.84	21.45
GEOROC[1]	Southeastern Lesser Caucasus	33.00	39.86	46.02	59.85	2.67	4.38	3.11	66	1615	32.00	70.00	2.00	4.00	0.04	50.47	23.78
GEOROC[1]	Southeastern Lesser Caucasus	143.00	39.86	46.02	57.08	2.29	4.53	2.87	40	1647	16.00	59.00	2.20	4.00	0.02	102.94	18.22
GEOROC[1]	Southeastern Lesser Caucasus	160.00	39.86	46.02	59.28	2.79	4.65	3.46	56	1360	19.00	67.00	2.10	4.00	0.04	71.58	21.67
GEOROC[1]	Southeastern Lesser Caucasus	185.00	39.86	46.02	57.85	2.77	4.53	2.89	48	790	15.00	48.00	1.30	4.00	0.06	52.67	25.08
GEOROC[1]	Southeastern Lesser Caucasus	73/P	39.86	46.02	67.80	1.10	5.50	4.00	70	1356	10.00	72.00	2.10	12.20	0.05	135.60	23.29
GEOROC[1]	Northern Lesser Caucasus	S21.1	41.13	43.69	63.94	2.63	4.07	2.84	58	395	21.47	38.74	1.92	11.36	0.15	18.37	13.70
GEOROC[1]	Northern Lesser Caucasus	S1.1	41.05	43.82	57.88	3.05	4.00	1.98	44	516	27.21	31.01	2.49	5.51	0.08	18.97	8.45
GEOROC[1]	Northern Lesser Caucasus	S1.2	41.05	43.82	57.84	3.11	4.07	1.98	46	529	27.86	30.28	2.50	5.53	0.09	18.97	8.23
GEOROC[1]	Northern Lesser Caucasus	S11.1	41.01	43.94	60.03	2.95	3.71	1.97	48	538	22.02	28.37	1.92	6.16	0.09	24.42	10.03
GEOROC[1]	Northern Lesser Caucasus	S12.1	41.07	44.10	65.45	1.51	3.95	2.33	69	477	11.17	27.78	0.96	8.21	0.14	42.71	19.75
GEOROC[1]	Northern Lesser Caucasus	S15.1	41.02	43.91	59.31	4.06	4.07	1.96	41	504	18.84	33.89	1.82	6.11	0.08	26.75	12.66
GEOROC[1]	Northern Lesser Caucasus	S16.1	41.02	43.89	58.50	3.26	4.16	1.76	39	527	26.80	31.04	2.49	5.30	0.07	19.66	8.48
GEOROC[1]	Northern Lesser Caucasus	S17.1	41.01	43.86	58.86	3.23	4.06	1.72	37	519	24.73	29.87	2.45	5.23	0.07	21.00	8.28
GEOROC[1]	Northern Lesser Caucasus	S18.1	41.08	43.80	57.56	4.66	3.95	1.59	26	492	24.01	32.14	2.55	5.46	0.05	20.51	8.56
GEOROC[1]	Northern Lesser Caucasus	S2.1	41.04	43.82	58.27	2.87	3.94	2.00	44	503	25.72	28.53	2.32	5.49	0.09	19.54	8.36
GEOROC[1]	Northern Lesser Caucasus	S20.1	41.09	43.66	61.95	3.00	4.15	2.47	49	453	18.82	37.60	1.84	9.40	0.11	24.05	13.87
GEOROC[1]	Northern Lesser Caucasus	S20.2	41.09	43.66	61.84	3.06	4.11	2.57	51	458	18.58	36.38	1.81	9.28	0.11	24.67	13.67
GEOROC[1]	Northern Lesser Caucasus	S22.1	41.08	43.61	62.27	3.20	3.78	2.28	55	392	19.70	29.19	1.87	8.77	0.14	19.89	10.58
GEOROC[1]	Northern Lesser Caucasus	S23.1	41.09	43.59	62.39	3.30	4.18	2.23	58	405	19.15	26.71	1.84	8.71	0.14	21.16	9.85
GEOROC[1]	Northern Lesser Caucasus	S24.1	41.10	43.56	62.70	2.63	4.01	2.48	64	435	17.77	26.65	1.63	8.46	0.15	24.49	11.14
GEOROC[1]	Northern Lesser Caucasus	S3.2	41.07	43.94	58.28	3.18	4.12	1.91	44	523	28.75	31.33	2.52	5.52	0.08	18.20	8.43
GEOROC[1]	Northern Lesser Caucasus	S30.1	41.12	43.90	58.82	3.55	4.33	1.84	45	584	20.20	27.53	1.83	4.76	0.08	28.92	10.23

Source	Location	Sample	Latitude	Longitude	SiO2(wt.%)	MgO(wt.%)	Na2O(wt.%)	K2O(wt.%)	Rb(ppm)	Sr(ppm)	Y(ppm)	La(ppm)	Yb(ppm)	Th(ppm)	Rb/Sr	Sr/Y	La/Ybn
GEOROC[1]	Northern Lesser Caucasus	S30.2	41.11	43.92	58.97	3.46	3.98	1.90	47	603	20.42	27.83	1.87	5.03	0.08	29.53	10.10
GEOROC[1]	Northern Lesser Caucasus	S30.3	41.11	43.92	68.24	1.55	3.79	2.34	74	433	12.45	26.11	1.11	8.25	0.17	34.76	15.92
GEOROC[1]	Northern Lesser Caucasus	S4.1	41.08	43.95	59.73	3.44	3.96	1.90	46	531	21.54	27.34	1.86	5.50	0.09	24.66	10.00
GEOROC[1]	Northern Lesser Caucasus	S4.2	41.08	43.95	57.67	3.36	3.77	1.83	42	562	27.22	31.76	2.36	4.90	0.07	20.64	9.14
GEOROC[1]	Northern Lesser Caucasus	S5.1	41.09	43.94	61.57	2.95	3.85	2.04	48	635	16.12	33.31	1.38	5.71	0.08	39.38	16.45
GEOROC[1]	Northern Lesser Caucasus	S6.1	41.09	43.94	62.18	2.75	3.82	2.18	52	662	16.22	36.64	1.35	5.88	0.08	40.79	18.46
GEOROC[1]	Northern Lesser Caucasus	S7.1	41.05	43.67	60.67	3.14	4.27	2.26	53	676	19.49	39.07	1.63	8.38	0.08	34.67	16.25
GEOROC[1]	Northern Lesser Caucasus	S7.2	41.05	43.67	60.43	3.40	4.23	2.28	52	690	19.38	39.10	1.63	8.08	0.08	35.60	16.27
GEOROC[1]	Northern Lesser Caucasus	S7.3	41.05	43.67	60.86	3.39	3.87	2.24	53	696	19.19	38.31	1.60	8.01	0.08	36.27	16.26
GEOROC[1]	Northern Lesser Caucasus	S7.4	41.05	43.67	60.15	3.75	4.27	2.13	50	701	19.03	38.16	1.64	7.45	0.07	36.85	15.78
GEOROC[1]	Northern Lesser Caucasus	S7.5	41.05	43.67	60.30	3.66	4.14	2.13	50	705	19.31	38.12	1.62	7.56	0.07	36.50	15.97
GEOROC[1]	Northern Lesser Caucasus	S8.1	41.01	43.94	60.22	2.71	4.18	1.98	48	539	22.60	28.40	1.93	6.05	0.09	23.85	9.98
GEOROC[1]	Northern Lesser Caucasus	S9.1	41.07	44.00	64.15	1.95	4.34	2.04	45	558	11.28	26.00	0.98	6.86	0.08	49.47	18.03
GEOROC[1]	Northern Lesser Caucasus	S9.2	41.07	43.99	65.80	1.77	4.12	2.21	57	540	11.65	27.38	1.01	7.50	0.11	46.39	18.50
Nomade et al., (2016)[6]	Northwestern Lesser Caucasus	TS-10-08	41.53	44.12	57.20	4.05	3.81	1.97	39	555	24.60	34.40	2.20	5.60	0.07	22.56	10.62
Nomade et al., (2016)[6]	Northwestern Lesser Caucasus	TS-05-08	41.65	44.11	56.10	4.79	3.99	1.74	32	618	24.40	34.80	2.10	5.20	0.05	25.34	11.26
Nomade et al., (2016)[6]	Northwestern Lesser Caucasus	TS-08-08	41.57	43.90	55.60	4.86	4.08	1.64	26	572	27.20	34.20	2.40	4.70	0.05	21.03	9.68
Nomade et al., (2016)[6]	Northwestern Lesser Caucasus	BORD-C-1	41.53	43.72	70.70	1.02	4.39	2.46	69	451	7.80	20.00	0.60	4.60	0.15	57.83	22.64
Nomade et al., (2016)[6]	Northwestern Lesser Caucasus	GODO-10-01	41.66	43.67	65.70	2.01	4.21	2.06	46	539	12.80	28.90	1.10	5.50	0.09	42.09	17.85
Nomade et al., (2016)[6]	Northwestern Lesser Caucasus	TS-07-08	41.65	43.84	64.90	2.55	4.04	1.94	42	526	11.70	28.70	0.90	5.20	0.08	44.97	21.66
Nomade et al., (2016)[6]	Northwestern Lesser Caucasus	SAM-94	41.65	43.73	64.80	1.95	4.23	2.07	45	544	12.40	29.10	1.10	5.50	0.08	43.85	17.97
Nomade et al., (2016)[6]	Northwestern Lesser Caucasus	MAR10-01	41.37	44.12	63.50	2.78	4.03	1.78	41	518	12.60	24.60	1.10	5.50	0.08	41.10	15.19
Nomade et al., (2016)[6]	Northwestern Lesser Caucasus	SAM-92	41.48	43.75	63.10	1.84	4.25	1.56	31	640	9.10	22.70	0.80	4.50	0.05	70.30	19.28
Nomade et al., (2016)[6]	Northwestern Lesser Caucasus	SAM-97	41.45	43.77	61.80	2.27	4.31	2.11	36	650	16.80	36.40	1.40	4.60	0.06	38.66	17.66
Nomade et al., (2016)[6]	Northwestern Lesser Caucasus	SAM-10-01	41.55	43.75	61.70	3.50	3.93	1.97	46	575	20.00	33.80	1.80	5.40	0.08	28.77	12.76
Nomade et al., (2016)[6]	Northwestern Lesser Caucasus	SAM-96	41.66	43.76	61.30	2.15	4.30	1.48	29	715	10.80	25.40	0.90	3.90	0.04	66.16	19.17
GEOROC[1]	Central Lesser Caucasus	Y3.1	40.32	44.55	56.42	3.80	4.16	2.13	44	882	24.00	50.00	2.20	6.00	0.05	36.75	15.44
GEOROC[1]	Central Lesser Caucasus	Y4.1	40.32	44.58	57.24	4.39	4.49	2.80	45	1015	23.00	59.00	2.20	6.60	0.04	44.13	18.22
Gao et al., (2015)[7]	Southeastern Tibet	TC-45	25.17	98.53	66.47	1.42	3.14	3.74	192	286	17.60	64.90	1.43	29.40	0.67	16.25	30.83
Gao et al., (2015)[7]	Southeastern Tibet	TC-46	25.17	98.53	65.83	0.95	3.71	3.45	159	336	21.60	62.30	1.72	25.60	0.47	15.56	24.61
Gao et al., (2015)[7]	Southeastern Tibet	TC-60C	25.17	98.53	65.24	1.33	2.81	4.11	153	315	29.10	97.90	2.81	41.80	0.49	10.82	23.67
Gao et al., (2015)[7]	Southeastern Tibet	TC-61	25.17	98.53	65.12	0.60	3.23	3.64	156	292	20.10	71.40	1.94	34.90	0.54	14.53	25.00
Gao et al., (2015)[7]	Southeastern Tibet	TC-62	25.17	98.53	66.59	1.15	3.34	3.59	150	326	19.50	67.80	1.94	34.00	0.46	16.72	23.74
Gao et al., (2015)[7]	Southeastern Tibet	TC-63	25.17	98.53	66.10	1.00	3.39	3.60	156	328	25.00	85.90	2.32	35.30	0.48	13.12	25.15
Gao et al., (2015)[7]	Southeastern Tibet	TC-64	25.17	98.53	66.64	1.31	3.56	3.70	151	339	19.40	64.20	1.90	33.80	0.44	17.47	22.95
Gao et al., (2015)[7]	Southeastern Tibet	TC-47	25.17	98.53	62.16	1.57	2.36	3.23	146	180	19.20	58.30	1.52	26.30	0.81	9.38	26.06
Gao et al., (2015)[7]	Southeastern Tibet	TC-48	25.17	98.53	63.49	1.56	2.65	3.29	161	206	18.60	55.70	1.57	26.10	0.78	11.08	24.10
Gao et al., (2015)[7]	Southeastern Tibet	TC-49	25.17	98.53	61.54	1.61	2.30	3.40	148	160	18.40	56.20	1.62	34.00	0.93	8.70	23.57
Gao et al., (2015)[7]	Southeastern Tibet	TC-50	25.17	98.53	62.12	1.57	3.05	3.45	158	189	17.90	60.10	1.67	38.20	0.84	10.56	24.45
Gao et al., (2015)[7]	Southeastern Tibet	TC-51	25.17	98.53	59.13	1.47	2.09	3.45	160	124	17.20	58.80	1.81	42.50	1.29	7.21	22.07
Gao et al., (2015)[7]	Southeastern Tibet	TC-52	25.17	98.53	63.35	1.57	3.29	3.44	150	210	17.50	58.50	1.65	38.30	0.71	12.00	24.09
Gao et al., (2015)[7]	Southeastern Tibet	TC-53	25.17	98.53	62.97	1.61	2.42	3.45	157	186	19.30	58.30	1.60	28.00	0.84	9.64	24.75

Source	Location	Sample	Latitude	Longitude	SiO2(wt.%)	MgO(wt.%)	Na2O(wt.%)	K2O(wt.%)	Rb(ppm)	Sr(ppm)	Y(ppm)	La(ppm)	Yb(ppm)	Th(ppm)	Rb/Sr	Sr/Y	La/Ybn
Gao et al., (2015)[7]	Southeastern Tibet	TC-54	25.17	98.53	59.04	1.78	2.22	3.20	119	129	21.80	62.70	1.75	28.80	0.92	5.92	24.34
Gao et al., (2015)[7]	Southeastern Tibet	TC-55	25.17	98.53	63.73	0.99	2.66	3.32	168	245	26.60	88.50	1.94	26.60	0.69	9.21	30.99
Zhou et al., (2012)[8]	Southeastern Tibet	TC-31 1	25.22	98.55	55.53	4.52	3.79	2.97	86	360	25.50	47.40	2.54	25.70	0.24	14.12	12.68
Zhou et al., (2012)[8]	Southeastern Tibet	TC-9	25.22	98.50	55.80	4.43	3.53	2.96	95	452	25.40	43.40	2.20	14.40	0.21	17.80	13.40
Zhou et al., (2012)[8]	Southeastern Tibet	TC-11	25.22	98.50	55.02	4.67	4.32	3.00	86	459	25.40	41.90	2.25	14.20	0.19	18.07	12.65
Zhou et al., (2012)[8]	Southeastern Tibet	TC-12	25.22	98.50	58.75	3.48	4.24	3.51	119	523	26.70	72.90	2.46	25.50	0.23	19.59	20.13
Zhou et al., (2012)[8]	Southeastern Tibet	TC-15	25.22	98.50	57.13	3.79	3.58	3.25	96	550	25.90	73.60	2.52	24.20	0.18	21.24	19.84
Zhou et al., (2012)[8]	Southeastern Tibet	TC-16	25.22	98.50	56.89	3.78	3.93	3.26	96	554	26.30	73.00	2.50	23.10	0.17	21.06	19.84
Zhou et al., (2012)[8]	Southeastern Tibet	TC-19	25.22	98.50	60.47	3.02	3.73	3.79	138	467	26.40	71.00	2.27	22.80	0.30	17.69	21.25
Zhou et al., (2012)[8]	Southeastern Tibet	TC-21	25.22	98.50	58.35	3.70	3.80	3.42	112	553	27.60	69.50	2.17	16.50	0.20	20.04	21.76
Zhou et al., (2012)[8]	Southeastern Tibet	TC-22	25.22	98.50	58.69	3.55	3.54	3.45	112	555	27.00	70.80	2.10	16.40	0.20	20.56	22.90
Zhou et al., (2012)[8]	Southeastern Tibet	TC-23	25.22	98.50	57.33	3.76	3.95	3.35	99	551	26.30	70.50	2.36	19.30	0.18	20.95	20.29
Zhou et al., (2012)[8]	Southeastern Tibet	TC-26	25.22	98.50	57.34	3.86	3.85	3.33	97	550	25.50	73.70	2.46	25.60	0.18	21.57	20.35
Tucker et al., (2013)[9]	Southeastern Tibet	DA09026	25.17	98.01	61.50	2.43	3.60	4.02	137	457	30.10	86.80	2.63	33.50	0.30	15.18	22.42
Zhang et al., (2012)[10]	Southeastern Tibet	DY08-1	25.13	98.47	62.04	2.51	3.63	3.99	132	444	30.33	83.31	2.65	32.77	0.30	14.62	21.36
Zhang et al., (2012)[10]	Southeastern Tibet	GD08-1	25.31	98.48	55.38	5.98	3.43	2.42	80	450	23.15	45.08	2.31	20.67	0.18	19.45	13.26
Zhang et al., (2012)[10]	Southeastern Tibet	MA08-2	25.02	98.43	59.05	3.61	3.88	3.25	94	492	25.28	65.45	2.49	23.16	0.19	19.46	17.86
Zhang et al., (2012)[10]	Southeastern Tibet	MA08-3	25.02	98.43	58.73	3.55	3.83	3.36	99	514	26.81	65.10	2.61	22.97	0.19	19.15	16.94
Zhang et al., (2012)[10]	Southeastern Tibet	XC08-1	25.03	98.40	58.55	3.71	3.82	3.17	98	551	27.28	67.47	2.69	24.09	0.18	20.18	17.04
Zhang et al., (2012)[10]	Southeastern Tibet	HS08-1	25.28	98.48	58.81	3.38	3.30	3.11	110	484	24.68	75.99	2.19	34.16	0.23	19.60	23.57
Zhang et al., (2012)[10]	Southeastern Tibet	JS08-2	25.23	98.48	55.06	4.88	3.73	2.58	69	423	24.98	42.74	2.37	18.70	0.16	16.92	12.25
Zhang et al., (2012)[10]	Southeastern Tibet	JS08-3	25.23	98.48	55.39	4.79	3.78	2.60	69	437	25.33	43.67	2.47	19.47	0.16	17.24	12.01
Zhang et al., (2012)[10]	Southeastern Tibet	TS08-1	25.23	98.47	58.93	4.06	3.63	3.25	99	518	25.97	71.41	2.44	23.28	0.19	19.94	19.88
Zhang et al., (2012)[10]	Southeastern Tibet	BH08-1	25.11	98.55	66.92	1.79	3.47	3.61	157	314	19.95	67.23	1.96	42.78	0.50	15.74	23.30
Zhang et al., (2012)[10]	Southeastern Tibet	LW08-3	25.05	98.55	66.69	0.94	3.27	3.66	157	320	21.84	72.53	2.08	35.62	0.49	14.66	23.69
Zhang et al., (2012)[10]	Southeastern Tibet	TT08-2	24.75	98.63	62.05	2.76	3.62	3.25	95	564	25.38	76.97	2.45	22.81	0.17	22.21	21.34
Zhang et al., (2012)[10]	Southeastern Tibet	TT08-4	24.74	98.64	61.24	2.92	3.35	3.43	88	582	25.48	81.40	2.47	22.84	0.15	22.83	22.39
Zhang et al., (2012)[10]	Southeastern Tibet	YW08-2	25.42	98.50	62.12	1.92	3.91	4.13	177	114	28.21	51.60	3.22	19.71	1.55	4.03	10.89
Zhang et al., (2012)[10]	Southeastern Tibet	DP08-1	25.13	98.47	61.25	2.68	3.71	3.78	128	484	28.95	80.86	2.62	34.30	0.26	16.71	20.97
Zhang et al., (2012)[10]	Southeastern Tibet	HY08-1	25.22	98.58	56.04	4.90	3.54	2.84	84	355	25.97	49.32	2.60	23.03	0.24	13.67	12.89
Shi et al., (2012)[11]	Southeastern Tibet	SD018-1	24.72	98.65	62.93	1.88	3.54	3.64	118	521	31.50	84.20	2.58	25.20	0.23	16.52	22.17
Shi et al., (2012)[11]	Southeastern Tibet	SD018-2	24.72	98.65	62.85	1.77	3.53	3.62	113	502	33.80	88.90	2.83	24.80	0.22	14.85	21.34
Shi et al., (2012)[11]	Southeastern Tibet	SD024-2-1	24.72	98.64	60.84	2.36	3.62	3.33	102	651	26.00	75.30	2.33	22.00	0.16	25.03	21.95
Shi et al., (2012)[11]	Southeastern Tibet	SD024-2-2	24.72	98.64	60.77	2.32	3.66	3.34	100	643	25.50	75.70	2.42	22.80	0.16	25.21	21.25
Li et al., (2015)[12]	Southeastern Tibet	TC1	25.20	98.58	62.02	1.97	3.19	3.39	119	445	20.70	66.10	1.85	35.40	0.27	21.50	24.27
Li et al., (2015)[12]	Southeastern Tibet	TC7	25.20	98.58	66.90	0.67	3.20	3.58	145	309	17.40	63.00	1.71	32.60	0.47	17.76	25.03
Li et al., (2015)[12]	Southeastern Tibet	TC16	25.20	98.58	56.42	3.61	3.48	2.95	79	486	24.50	59.20	2.24	16.30	0.16	19.84	17.95
Xu et al., (2012)[13]	Southeastern Tibet	TC-01	25.21	98.49	55.61	3.53	3.80	2.20	46	471	22.65	36.10	2.58	13.30	0.10	20.79	9.51
Xu et al., (2012)[13]	Southeastern Tibet	TC-02	25.21	98.49	56.07	3.77	3.44	1.85	47	424	23.75	34.87	2.55	15.47	0.11	17.87	9.29
Xu et al., (2012)[13]	Southeastern Tibet	TC-04	25.21	98.49	55.69	3.87	4.21	2.16	46	463	22.78	37.20	2.47	14.64	0.10	20.34	10.23
Xu et al., (2012)[13]	Southeastern Tibet	TC-05	25.21	98.49	58.45	3.07	3.67	2.57	72	474	24.18	44.02	2.63	19.65	0.15	19.59	11.37
Xu et al., (2012)[13]	Southeastern Tibet	TC-06	25.21	98.49	55.54	3.29	3.79	2.30	52	374	22.19	38.48	2.53	15.56	0.14	16.85	10.33
Xu et al., (2012)[13]	Southeastern Tibet	TC-09	25.26	98.50	56.43	3.22	3.71	2.35	53	284	23.62	40.29	2.59	16.24	0.19	12.02	10.57
Xu et al., (2012)[13]	Southeastern Tibet	TC-11	25.26	98.50	61.13	3.03	3.71	3.18	96	279	24.80	72.69	2.59	23.62	0.34	11.25	19.07
Li and Liu, (2012)[14]	Southeastern Tibet	HSK01	25.17	98.50	58.78	4.05	3.60	3.38	103	542	26.51	74.22	2.42	22.47	0.19	20.45	20.83

Source	Location	Sample	Latitude	Longitude	SiO2(wt.%)	MgO(wt.%)	Na2O(wt.%)	K2O(wt.%)	Rb(ppm)	Sr(ppm)	Y(ppm)	La(ppm)	Yb(ppm)	Th(ppm)	Rb/Sr	Sr/Y	La/Ybn
Li and Liu, (2012)[14]	Southeastern Tibet	HSK02	25.17	98.50	59.07	3.35	3.29	3.03	124	478	23.74	76.28	2.19	35.16	0.26	20.13	23.66
Li and Liu, (2012)[14]	Southeastern Tibet	HSK03	25.17	98.50	59.69	3.47	3.55	3.64	118	483	25.51	68.64	2.47	28.74	0.24	18.93	18.88
Li and Liu, (2012)[14]	Southeastern Tibet	HSK04	25.17	98.50	59.86	3.11	3.35	3.19	113	390	20.35	62.27	1.87	29.57	0.29	19.16	22.62
Li and Liu, (2012)[14]	Southeastern Tibet	DYS01	25.17	98.50	61.39	2.44	3.55	3.98	130	481	30.37	86.84	2.84	32.75	0.27	15.84	20.77
Li and Liu, (2012)[14]	Southeastern Tibet	DYS02	25.17	98.50	60.54	2.53	3.61	3.88	129	448	27.35	77.32	2.64	34.00	0.29	16.38	19.90
Li and Liu, (2012)[14]	Southeastern Tibet	DYS03	25.17	98.50	67.68	1.69	3.25	3.75	187	294	20.21	68.13	1.83	40.61	0.64	14.55	25.29
Li and Liu, (2012)[14]	Southeastern Tibet	DYS04	25.17	98.50	64.64	1.91	2.91	3.94	153	273	19.34	61.75	1.81	40.03	0.56	14.12	23.18
Li and Liu, (2012)[14]	Southeastern Tibet	DYS05	25.17	98.50	58.58	2.68	3.52	3.68	114	489	28.85	83.51	2.88	32.95	0.23	16.95	19.70
Li and Liu, (2012)[14]	Southeastern Tibet	MAS05	25.17	98.50	57.44	3.62	3.72	3.26	95	590	27.33	68.36	2.66	22.42	0.16	21.59	17.46
Li and Liu, (2012)[14]	Southeastern Tibet	MAS07	25.17	98.50	58.45	3.65	3.71	3.36	109	566	27.53	69.70	2.68	24.78	0.19	20.56	17.67
Zhang et al., (2008)[15]	Northen Tibet (W)	PL-9	36.28	81.48	55.18	3.97	3.64	4.14	119	1060	24.59	144.00	2.05	25.00	0.11	43.11	47.72
Wang and Zhang, (2011)[16]	Northen Tibet (W)	CY2-6	36.28	81.48	55.12	3.80	3.40	3.96	107	1215	28.70	129.00	1.89	17.40	0.09	42.33	46.37
Wang et al., (2012)[18]	Northen Tibet (E)	2511-1*	35.97	90.79	69.24	0.57	3.04	5.47	476	72	9.84	79.80	0.62	103.00	6.66	7.27	87.44
Wang et al., (2016)[19]	Northen Tibet (E)	5123-2	34.43	89.13	65.04	1.11	3.65	4.78	226	498	22.43	152.80	1.56	5.41	0.45	22.18	66.58
Wang et al., (2016)[19]	Northen Tibet (E)	5124-1	34.42	89.19	65.39	0.98	3.22	5.10	250	439	22.78	182.50	1.53	5.81	0.57	19.29	81.03
Wang et al., (2016)[19]	Northen Tibet (E)	5124-2	34.42	89.19	65.54	1.07	3.41	4.89	242	528	23.86	182.30	1.60	5.66	0.46	22.14	77.26
Wang et al., (2016)[19]	Northen Tibet (E)	5126-1	34.40	89.24	65.64	1.01	3.74	4.99	275	506	23.77	198.50	1.63	6.14	0.54	21.30	82.63
Wang et al., (2016)[19]	Northen Tibet (E)	5127-3	34.39	89.26	65.73	0.97	3.41	5.13	240	445	23.48	182.60	1.56	5.72	0.54	18.96	79.31
Wang et al., (2016)[19]	Northen Tibet (E)	5133-1	34.39	89.28	65.67	1.04	3.31	5.16	252	495	22.87	199.10	1.51	5.82	0.51	21.64	89.34
Wang et al., (2016)[19]	Northen Tibet (E)	5125-1	34.41	89.21	66.05	1.00	3.17	5.14	243	474	18.20	144.50	1.29	5.52	0.51	26.03	76.21
Wang et al., (2016)[19]	Northen Tibet (E)	5127-1	34.39	89.26	66.25	1.01	3.18	5.26	250	442	18.88	150.70	1.37	6.22	0.57	23.43	74.62
Wang et al., (2016)[19]	Northen Tibet (E)	11WL59-2	34.53	90.55	62.98	1.37	3.47	4.52	172	639	24.26	150.00	1.59	34.01	0.27	26.34	64.13
Wang et al., (2016)[19]	Northen Tibet (E)	11WL59-3	34.53	90.55	63.07	1.47	3.51	4.62	170	633	22.14	137.90	1.43	33.16	0.27	28.57	65.51
Wang et al., (2016)[19]	Northen Tibet (E)	11WL59-4	34.53	90.55	63.02	1.58	3.50	4.58	179	656	25.21	147.80	1.51	36.39	0.27	26.01	66.40
Wang et al., (2016)[19]	Northen Tibet (E)	11WL61-3	34.54	90.55	63.26	1.41	3.51	4.63	173	648	22.00	131.20	1.47	33.92	0.27	29.47	60.47
Wang et al., (2016)[19]	Northen Tibet (E)	11WL61-4	34.54	90.55	63.00	1.46	3.52	4.65	173	643	22.38	136.20	1.39	33.56	0.27	28.74	66.80
Wang et al., (2016)[19]	Northen Tibet (E)	11WL60-3	34.54	90.55	63.10	1.51	3.51	4.63	174	658	23.62	144.00	1.48	34.28	0.26	27.86	66.10
Wang et al., (2016)[19]	Northen Tibet (E)	D5160-GS1	34.84	88.92	67.14	0.94	3.36	5.08	210	420	7.05	82.30	0.70	30.80	0.50	59.57	80.33
Wang et al., (2016)[19]	Northen Tibet (E)	D7535C1	34.84	88.92	68.05	0.87	3.38	5.08	179	586	8.78	74.39	1.06	27.60	0.31	66.71	47.81
Wang et al., (2016)[19]	Northen Tibet (E)	MB1-1	33.65	90.31	63.56	1.39	3.60	4.42	194	550	23.60	156.00	2.05	32.50	0.35	23.31	51.69
Wang et al., (2016)[19]	Northen Tibet (E)	MB1-4	33.65	90.31	63.44	1.30	3.43	4.48	199	560	22.20	136.00	2.01	32.80	0.36	25.23	45.96
Wang et al., (2016)[19]	Northen Tibet (E)	MB2	33.65	90.31	63.58	1.43	3.65	4.52	205	550	23.00	144.00	2.09	30.50	0.37	23.91	46.81
Wang et al., (2016)[19]	Northen Tibet (E)	MB4	33.65	90.31	63.16	1.34	3.54	4.55	192	620	20.90	138.00	1.81	29.80	0.31	29.67	51.79
Wang et al., (2016)[19]	Northen Tibet (E)	C1601-1	33.65	90.31	65.73	0.83	3.36	4.82	251	400	25.20	122.00	2.51	49.80	0.63	15.87	33.02
Wang et al., (2016)[19]	Northen Tibet (E)	C1603-1h	33.65	90.31	65.20	1.11	3.32	4.64	238	520	25.80	142.00	2.38	40.10	0.46	20.16	40.53
Wang et al., (2016)[19]	Northen Tibet (E)	2303a	35.75	90.65	70.67	0.51	3.28	5.42	418	113	6.75	48.49	0.58	47.15	3.70	16.75	56.70
Wang et al., (2016)[19]	Northen Tibet (E)	2511-1	35.95	90.78	69.24	0.57	3.04	5.47	476	72	9.84	79.78	0.62	103.49	6.65	7.27	87.06
Wang et al., (2016)[19]	Northen Tibet (M)	10-1	36.69	86.07	60.62	2.20	2.92	4.01	159	821	20.64	154.30	1.32	40.50	0.19	39.78	79.41
Wang et al., (2016)[19]	Northen Tibet (M)	43-1	36.68	86.08	60.82	1.95	3.03	4.12	136	856	22.49	162.30	1.40	43.40	0.16	38.06	78.75
Wang et al., (2016)[19]	Northen Tibet (M)	46-2	36.65	86.08	60.17	2.10	3.05	4.05	166	882	23.63	179.10	1.45	43.60	0.19	37.33	83.91
Wang et al., (2016)[19]	Northen Tibet (M)	40	36.69	86.04	60.54	2.02	2.99	4.16	170	890	23.02	178.80	1.43	42.80	0.19	38.66	84.94
Wang et al., (2016)[19]	Northen Tibet (M)	7	36.69	86.10	57.88	2.06	3.09	3.70	152	975	21.98	169.40	1.31	30.50	0.16	44.36	87.85

Source	Location	Sample	Latitude	Longitude	SiO2(wt.%)	MgO(wt.%)	Na2O(wt.%)	K2O(wt.%)	Rb(ppm)	Sr(ppm)	Y(ppm)	La(ppm)	Yb(ppm)	Th(ppm)	Rb/Sr	Sr/Y	La/Ybn
Wang et al., (2016)[19]	Northen Tibet (M)	2.00	36.71	86.05	60.56	1.99	3.12	4.15	174	874	22.70	172.10	1.40	39.10	0.20	38.50	83.51
Gao et al., (2010)[20]	Southwestern Tibet	PRDZ1	30.90	81.70	65.61	1.63	3.93	3.82	149	1025	12.00	42.20	1.03	16.50	0.15	85.42	27.78
Gao et al., (2010)[20]	Southwestern Tibet	PRDZ2	30.90	81.70	65.97	1.75	3.83	3.84	143	988	12.10	50.80	1.00	17.50	0.14	81.61	34.37
Gao et al., (2010)[20]	Southwestern Tibet	PRDZ3	30.90	81.70	65.73	1.76	3.78	3.79	134	994	11.20	41.50	0.96	16.00	0.13	88.75	29.46
Gao et al., (2010)[20]	Southwestern Tibet	PRDZ4	30.90	81.70	65.02	1.72	3.82	3.85	149	950	11.60	46.70	1.00	17.60	0.16	81.90	31.60
Gao et al., (2010)[20]	Southwestern Tibet	PRDZ5	30.90	81.70	65.29	1.70	3.77	3.80	155	995	12.90	49.70	1.10	17.90	0.16	77.13	30.81
Gao et al., (2010)[20]	Southwestern Tibet	07BB-02	31.00	80.60	63.38	1.65	3.19	3.60	127	1030	13.02	42.53	1.07	18.78	0.12	79.11	27.00
Gao et al., (2010)[20]	Southwestern Tibet	07BB-03	31.00	80.60	62.34	2.30	3.29	3.84	142	1239	17.47	48.62	1.39	21.34	0.11	70.92	23.76
Gao et al., (2010)[20]	Southwestern Tibet	07BB-04	31.00	80.60	62.28	2.26	3.17	3.71	147	1283	15.51	53.62	1.28	21.35	0.11	82.72	28.46
Chung et al., (2003)[21]	Southern Tibet (E)	ET023	29.61	91.60	65.27	1.53	4.19	2.95	86	1048	8.20	21.90	0.65	8.83	0.08	127.80	22.89
Yang et al., (2015)[22]	Southern Tibet (E)	1602-515	29.62	91.60	58.80	4.17	3.34	3.55	167	838	9.69	38.40	0.85	33.60	0.20	86.48	30.69
Yang et al., (2015)[22]	Southern Tibet (E)	1602-516	29.62	91.60	59.70	4.35	3.71	3.59	169	942	9.89	36.15	0.86	39.60	0.18	95.25	28.56
Yang et al., (2015)[22]	Southern Tibet (E)	1602-517	29.62	91.60	56.20	5.06	3.56	3.28	162	874	10.60	38.90	0.93	38.70	0.19	82.45	28.41
Yang et al., (2015)[22]	Southern Tibet (E)	1602-519	29.62	91.60	58.30	5.03	3.70	3.44	174	1175	10.60	41.00	0.92	36.40	0.15	110.85	30.27
Yang et al., (2015)[22]	Southern Tibet (E)	1602-544	29.62	91.60	60.40	4.23	3.55	3.18	135	793	10.50	36.70	0.80	32.40	0.17	75.52	31.16
Yang et al., (2015)[22]	Southern Tibet (E)	1602-546	29.62	91.60	60.40	4.63	4.01	3.31	154	980	10.20	39.70	0.90	39.00	0.16	96.08	29.97
Yang et al., (2015)[22]	Southern Tibet (E)	1602-551	29.62	91.60	60.70	4.31	3.34	3.35	129	749	9.89	37.50	0.86	48.30	0.17	75.73	29.62
Yang et al., (2015)[22]	Southern Tibet (E)	2005-379	29.62	91.60	59.60	5.10	3.90	3.20	170	954	12.70	44.40	1.03	37.80	0.18	75.12	29.28
Chen et al., (2011)[23]	Southern Tibet (E)	LKE03-02	29.53	91.40	69.95	1.73	3.71	3.86	98	715	3.77	11.03	0.49	9.34	0.14	189.66	15.29
Chung et al., (2003)[21]	Southern Tibet (E)	ET025E	29.69	91.75	66.76	0.93	3.63	3.43	159	689	6.90	24.40	0.53	14.20	0.23	99.86	31.27
Chung et al., (2003)[21]	Southern Tibet (M)	ET026C	29.48	90.87	65.41	1.29	4.25	2.78	67	902	6.40	21.30	0.44	7.49	0.07	140.94	32.89
Chung et al., (2003)[21]	Southern Tibet (M)	ET026D	29.48	90.87	63.62	1.04	4.61	2.71	61	1051	5.60	21.60	0.38	8.86	0.06	187.68	38.61
Xu et al., (2010)[24]	Southern Tibet (M)	T379	29.58	90.00	67.63	1.37	4.03	3.67	153	766	8.42	32.90	0.59	31.90	0.20	90.97	37.88
Xu et al., (2010)[24]	Southern Tibet (M)	T380	29.58	90.00	68.44	1.11	4.18	3.86	158	814	6.20	24.60	0.48	22.60	0.19	131.29	34.82
Xu et al., (2010)[24]	Southern Tibet (M)	T358	29.58	90.00	66.09	1.00	4.11	3.59	145	673	6.69	28.40	0.50	21.60	0.22	100.60	38.59
Xu et al., (2010)[24]	Southern Tibet (M)	T381	29.58	90.00	68.61	1.15	4.03	3.72	162	724	7.17	29.60	0.54	29.10	0.22	100.98	37.24
Williams et al., (2004)[25]	Southern Tibet (M)	JPT3	29.83	89.00	64.70	1.30	3.20	4.60	116	803	11.00	30.90	0.80	19.30	0.14	72.99	26.24
Williams et al., (2004)[25]	Southern Tibet (M)	JPT4	29.83	89.00	70.30	0.80	3.40	4.10	147	651	4.80	22.80	0.40	16.40	0.23	135.67	38.72
Chung et al., (2003)[21]	Southern Tibet (M)	T065C	29.74	89.88	57.47	2.39	3.60	2.71	81	1121	9.00	37.60	0.61	17.90	0.07	124.56	41.87
Gao et al., (2010)[20]	Southern Tibet (W)	ZM-13	29.66	87.47	65.99	1.54	4.13	3.71	40	825	8.69	20.60	0.87	2.95	0.05	94.94	16.09
Gao et al., (2010)[20]	Southern Tibet (W)	ZM-3	29.66	87.47	65.37	1.84	4.02	3.25	141	884	17.50	33.36	1.00	24.60	0.16	50.51	22.66
Gao et al., (2010)[20]	Southern Tibet (W)	ZM-4	29.66	87.47	67.00	1.60	3.92	3.49	158	752	11.00	37.80	0.96	24.50	0.21	68.36	26.75
Gao et al., (2010)[20]	Southern Tibet (W)	ZM-6	29.66	87.47	65.10	1.94	4.00	3.19	142	878	11.20	38.70	0.91	21.70	0.16	78.39	28.89
Gao et al., (2010)[20]	Southern Tibet (W)	ZM-7	29.66	87.47	63.86	2.35	4.27	3.41	153	807	9.57	33.60	0.83	24.60	0.19	84.33	27.50
Chung et al., (2003)[21]	Southern Tibet (W)	T041D	29.36	88.81	56.73	3.31	4.11	1.72	31	911	9.50	17.80	0.74	3.27	0.03	95.89	16.34

Note: W - west part; M - middle part; E - east part.

n denotes the value is normalized to chondritic values of McDonough and Sun (1995)[26].

The data in blue are rocks formed during middle Miocene.

Table S5. Data subsets used to plot correlation between Moho depth and La/Ybn.

Location	Moho(km)	uncertainty	Average Rb/Sr(*)	Average La(ppm)(*)	Median La/Ybn(*)	std(*)	Average Rb/Sr(**)	Average La(ppm)(**)	Median La/Ybn(**)	std(**)
North Eastern Anatolia (W)	43.12	1.22	0.16	31.06	14.10	5.25	0.16	31.06	14.10	5.25
North Eastern Anatolia (E)	47.21	2.99	0.17	33.10	6.93	1.28	0.15	25.30	7.22	1.06
Northeastern Central Anatolia	42.65	1.02	0.28	34.78	13.88	5.81	0.31	33.97	14.30	5.74
South Central Anatolia (W)	36.24	0.44	0.30	27.70	11.58	2.25	0.28	29.01	11.58	2.25
South Central Anatolia (E)	36.61	0.71	0.11	25.88	8.16	0.77	0.26	25.88	8.16	0.77
Northern Lesser Caucasus	44.49	1.58	0.09	31.76	11.14	3.75	0.09	31.76	11.14	3.75
Northwestern Lesser Caucasus	43.80	2.03	0.07	29.42	17.76	4.35	0.07	29.42	17.76	4.35
Central Lesser Caucasus	47.55	2.17	0.05	54.50	16.83	1.97	0.06	38.00	12.63	3.98
Southwestern Tibet	57.95	0.82	0.14	46.96	28.96	3.21	0.14	46.96	28.96	3.21
Southern Tibet (E)	66.00	2.75	0.17	33.64	29.62	4.79	0.17	36.75	29.97	1.01
Southern Tibet (M)	68.42	1.19	0.16	27.74	37.88	4.55	0.16	27.74	37.88	4.55
Southern Tibet (W)	64.07	3.18	0.13	30.31	24.71	5.68	0.13	30.31	24.71	5.68
North Eastern Anatolia (M)	42.98	1.40	0.44	39.14	5.42	1.09	0.77	46.76	3.94	0.31
Northwestern Iran	44.62	0.87	0.06	63.15	33.56	6.17				
Northern Iran	42.91	2.78	0.09	94.78	59.59	1.85				
Greater Caucasus (W)	43.48	0.47	0.65	46.16	21.24	1.99	0.65	46.16	21.24	1.99
Southeastern Lesser Caucasus	46.66	2.00	0.05	61.89	21.67	2.44	0.04	60.00	22.05	0.84
Southeastern Tibet	39.48	2.25	0.37	65.50	21.25	5.07	0.41	70.77	22.62	2.39
Northen Tibet (W)	52.20	2.59	0.10	136.50	47.04	0.96		169.33	83.71	3.44
Northen Tibet (E)	66.92	1.83	1.04	137.71	66.40	15.81	1.10	135.14	66.10	16.10
Northen Tibet (M)	52.38	1.25	0.18	169.33	83.71	3.44				

Note: Note: W - west part; M - middle part; E - east part; std - standard deviation.

n denotes the value is normalized to chondritic values of McDonough and Sun (1995)[26].

The data in blue are rocks formed during middle Miocene.

The subsets with value in red are discarded due to their higher value of Rb/Sr or std.

* represents value calculated from all samples.

** represents value calculated from sub-alkaline samples.

Table S6. Data used to construct Figures 3 and 4.

Location	Moho(km)	std	Sr(ppm)	std	Y(ppm)	std	SiO2(wt.%)	std	La(ppm)	std	Yb(ppm)	std	Th(ppm)	std
Aegean	28.20	0.60	374.9	46.4	26.9	3.4	58.6	2.0	23.0	4.4	2.8	0.6	6.7	2.8
Aeolian	24.90	1.00	617.6	113.4	24.5	5.0	58.1	2.3	33.3	11.2	2.2	0.3	11.3	5.9
NE Aleutian	40.00	3.00	523.5	26.0	18.1	1.7	60.1	1.2	11.9	0.3	1.7	0.0	1.4	0.2
Aleutian	18.90	4.40	341.8	66.1	30.4	5.4	58.9	2.6	11.5	2.1	2.8	0.5	3.1	1.0
NVZ	54.40	4.70	531.5	103.8	12.1	3.0	61.4	2.2	14.5	5.6	1.1	0.4	4.2	2.6
N_CVZ	65.00	0.70	811.0	188.4	16.1	3.2	59.3	2.0	36.2	13.1	1.3	0.5	8.0	3.5
S_CVZ	65.00	0.70	740.3	143.9	15.8	4.1	60.1	2.4	37.8	8.7	1.2	0.4	7.6	3.3
SVZ	40.00	5.00	502.0	359.0	28.4	22.3	58.3	2.4	19.0	6.1	2.4	1.0	5.9	2.6
New Britain	27.10	4.30	319.9	62.4	32.7	5.8	60.2	2.6	10.4	2.6	3.6	0.8	1.5	0.8
Cascades	38.00	1.90	488.5	118.0	22.8	7.4	60.8	2.4	16.9	5.7	1.7	0.6	4.1	1.6
C. America	28.00	7.00	436.0	64.0	27.7	5.2	58.5	1.7	21.3	10.5	3.0	0.7	4.4	2.6
Guatamala	44.00	3.00	503.9	43.7	16.3	2.8	61.0	1.7	17.0	1.6	1.7	0.2	3.8	0.5
G. Antilles	25.00	5.00	276.7	90.1	21.4	6.1	60.0	3.7	10.7	5.5	2.7	1.5	1.8	1.1
Honshu	30.20	3.60	361.3	71.2	30.7	8.5	57.8	2.1	10.8	2.7	2.6	0.5	3.5	1.3
Izu-Bonin	20.50	2.70	225.0	72.7	26.7	8.3	58.1	1.9	5.1	2.4	3.1	1.1	1.0	0.7
Marianas	14.50	1.00	339.2	40.2	35.8	4.2	59.6	1.6	12.8	2.2	3.9	0.7	1.9	0.5
Kamchatka	34.60	4.80	380.0	58.9	28.6	9.5	58.4	2.6	13.1	4.9	3.0	1.0	1.9	0.8
Kurile	18.30	0.90	293.2	49.4	30.2	5.6	58.8	2.2	10.0	4.0	3.5	0.9	2.8	0.9
L. Antilles	24.70	0.70	267.7	36.0	22.4	3.1	59.5	2.1	10.1	1.3	2.3	0.3	2.8	0.7
Liguria	30.00	3.00	391.3	141.9	25.5	10.6	56.8	1.9	20.9	4.5	2.7	0.5	4.9	1.8
Luzon	30.80	1.40	504.5	158.7	22.6	8.7	58.8	2.6	17.2	7.5	2.1	1.0	6.7	3.5
Mexican	37.20	9.00	478.3	66.2	22.7	4.7	60.6	2.7	21.5	6.8	1.9	0.6	4.2	1.3
New Hebrides	25.00	3.00	527.4	126.8	28.5	6.3	57.6	2.1	14.3	3.3	3.3	1.1	2.2	0.7
Ryukyu	24.50	3.40	461.5	83.8	25.6	5.0	58.2	2.2	12.6	3.1	3.2	0.5	2.7	0.7
S. Sandwich	11.80	0.10	136.7	15.4	30.2	4.3	56.8	1.0	7.7	3.2	3.6	0.7	1.4	0.5
S. Shetland	26.20	1.20	448.7	83.8	25.8	5.0	59.0	2.4	17.6	4.7	2.6	0.9	5.4	2.0
Sulawesi	27.40	2.20	335.6	48.7	29.2	8.3	58.8	1.9	8.7	3.8	2.5	0.7	2.1	1.6
Sunda	27.80	1.80	408.4	89.3	26.0	5.2	57.7	2.1	16.5	5.1	2.7	0.9	5.5	1.8
Tonga	20.00	3.00	231.9	103.5	19.4	8.3	59.3	2.3	4.5	1.0	2.8	0.9	0.4	0.1

Location	Moho(km)	std	Sr(ppm)	std	Y(ppm)	std	SiO2(wt.%)	std	La(ppm)	std	Yb(ppm)	std	Th(ppm)	std
North Eastern Anatolia (W)	43.12	1.22	396.3	79.9	20.2	8.3	63.1	3.6	31.1	6.2	1.8	0.7	8.4	1.9
North Eastern Anatolia (E)	47.21	2.99	409.6	62.9	24.6	3.3	64.3	4.0	33.1	14.3	3.3	1.7	8.1	3.5
Northeastern Central Anatolia	42.65	1.02	284.6	9.0	18.1	0.6	63.1	0.5	34.8	4.2	1.7	0.6	14.6	4.1
South Central Anatolia (W)	36.24	0.44	285.6	57.7	17.2	2.5	66.8	4.0	27.7	2.7	1.7	0.3	11.2	3.3
South Central Anatolia (E)	36.61	0.71	274.8	44.7	23.3	3.7	65.7	3.0	25.9	3.4	2.1	0.2	10.7	3.5
Eastern Carpathian	37.67	2.09	308.2	51.7	23.1	4.4	61.1	3.3						
Greater Caucasus (E)	45.14	1.80	580.2	69.6	22.7	4.8	62.5	2.9						
Northern Lesser Caucasus	44.49	1.58	541.3	92.7	20.3	4.8	60.9	2.6	31.8	4.5	1.8	0.5	6.9	1.7
Northwestern Lesser Caucasus	43.80	2.03	550.2	67.0	22.1	5.6	59.5	3.8	29.4	5.4	1.4	0.6	5.0	0.5
Central Lesser Caucasus	47.55	2.17					56.8	0.6	54.5	6.4	2.2	0.0	6.3	0.4
Southeastern Tibet	39.48	2.25	416.5	127.5	24.2	3.8	60.5	3.7						
Northen Tibet (W)	52.20	2.59	996.8	174.4	29.3	3.6	57.6	2.9						
Northen Tibet (M)	52.38	1.25	864.6	27.4	22.5	1.1	60.5	0.2						
Southwestern Tibet	57.95	0.82					64.5	1.5	47.0	4.5	1.1	0.2	18.4	2.0
Southern Tibet (E)	66.00	2.75					61.5	4.1	33.6	10.1	0.8	0.2	30.7	13.5
Southern Tibet (M)	68.42	1.19					65.8	3.8	27.7	5.6	0.5	0.1	19.5	8.1
Southern Tibet (W)	64.07	3.18					64.0	3.7	30.3	8.9	0.9	0.1	16.9	10.8

Note: W - west part; M - middle part; E - east part; std - standard deviation.

The data subsets in red are rocks formed at subduction-related magmatic arcs (Chapman et al., 2015[27]; Profeta et al., 2015[28]).

The data subsets in blue are rocks formed during middle Miocene.

References for supplementary tables

1. Sarbas, B., Nohl, U., Busch, U., Kalbskopf, B. & Youssa, C. Geochemistry of Rocks of the Oceans and Continents. *GEOROC* http://georoc.mpch-mainz.gwdg.de/georoc/Start.asp (2016).
2. Chernyshev, I. V. et al. Two stages of explosive volcanism of the Elbrus area: Geochronology, petrochemical and isotopic-geochemical characteristics of volcanic rocks, and their role in the neogene-quaternary evolution of the Greater Caucasus. *Stratigr. Geol. Correl.* **22**, 96–121 (2014).
3. Lebedev, V. A. et al. Geochronology and genesis of the young (Pliocene) granitoids of the Greater Caucasus: Dzhimara multiphase Massif of the Kazbek neovolcanic area. *Geochem. Int.* **47**, 550–567 (2009).
4. Lebedev, V. A., Bubnov, S. N., Dudauri, O. Z. & Vashakidze, G. T. Geochronology of Pliocene volcanism in the Dzhavakheti Highland (the Lesser Caucasus). Part 1: Western part of the Dzhavakheti Highland. *Stratigr. Geol. Correl.* **16**, 204–224 (2008).
5. Lebedev, V. A., Bubnov, S. N., Dudauri, O. Z. & Vashakidze, G. T. Geochronology of Pliocene volcanism in the Dzhavakheti Highland (the Lesser Caucasus). Part 2: Eastern part of the Dzhavakheti Highland. Regional geological correlation. *Stratigr. Geol. Correl.* **16**, 553–574 (2008).
6. Nomade, S. et al. New $^{40}Ar/^{39}Ar$, unspiked K/Ar and geochemical constraints on the Pleistocene magmatism of the Samtskhe-Javakheti highlands (Republic of Georgia). *Quatern. Int.* **395**, 45–59 (2016).
7. Gao, J.-F. et al. Magma mixing recorded by Sr isotopes of plagioclase from dacites of the Quaternary Tengchong volcanic field, SE Tibetan Plateau. *J. Asian. Earth. Sci.* 98, 1–17 (2015).
8. Zhou, M.-F. et al. Heterogeneous mantle source and magma differentiation of quaternary arc-like volcanic rocks from Tengchong, SE margin of the Tibetan Plateau. *Contrib. Mineral. Petrol.* **163**, 841–860 (2012).
9. Tucker, R. T., Zou, H., Fan, Q. & Schmitt, A. K. Ion microprobe dating of zircons from active Dayingshan volcano, Tengchong, SE Tibetan Plateau: Time scales and

nature of magma chamber storage. *Lithos* **172–173**, 214–221 (2013).

10. Zhang, Y., Liu, J. & Meng, F. Geochemistry of Cenozoic volcanic rocks in Tengchong, SW China: relationship with the uplift of the Tibetan Plateau. *Isl. Arc.* **21**, 255–269 (2012).
11. Shi, Y. R., Wu, Z. H., Fan, T. Y., Tong, Y. B. & Yang, Z. Y. SHRIMP zircon U–Pb dating and geochemical analysis of the Pliocene volcanic rocks from Longchuanjiang valley, Tengchong area, western Yunnan Province. *Geol. Bull. China.* **31**, 241–249 (2012).
12. Li, L. L., Wang, S. B., Liu, J. H. & Shi, Y. R. Age and origin of Mid-Pleistocene volcanic rocks from Qushi in Tengchong area, western Yunnan Province: SHRIMP zircon U–Pb dating and constrain from Hf-in-zircon isotopes. *Acta. Petrol. Sinica* **31**, 2609–2619 (2015).
13. Xu, C. L., Zhao, G. T., He, Y. Y. & Li, D. P. Geochemistry of Cenozoic volcanic rocks from Tengchong, western Yunnan. *Mar. Geol. Quatern. Geol.* **32**, 65–75 (2012).
14. Li, X. & Liu, J. Q. A study on the geochemical characteristics and petrogenesis of Holocene volcanic rocks in the Tengchong volcanic eruption field, Yunnan Province, SW China. *Acta. Petrol. Sinica* **28**, 1507–1516 (2012).
15. Zhang, Z., Xiao, X., Wang, J., Wang, Y. & Kusky, T. M. Post-collisional Plio-Pleistocene shoshonitic volcanism in the western Kunlun Mountains, NW China: Geochemical constraints on mantle source characteristics and petrogenesis. *J. Asian. Earth. Sci.* **31**, 379–403 (2008).
16. Wang, H. Y. & Zhang, C. L. Age and geochemical characteristics of Quaternary volcanic rocks in the northwestern margin of Tibetan Plateau and their significance. *Geol. Bull. China.* **30**, 1171–1181 (2011).
17. Ning, W. K, Chi, X. G., Liu, J. F., Zhao, Z. & Li, C. Genesis of Heishibeihu Cenozoic potassic volcanic rocks from northern Qinghai–Tibet Plateau, China. *Geol. Bull. China.* **28**, 1355–1360 (2009).
18. Wang, Q. et al. Crustal melting and flow beneath Northern Tibet: evidence from Mid-Miocene to Quaternary strongly peraluminous rhyolites in the Southern Kunlun Range. *J. Petrol.* **53**, 2523–2566 (2012).

19. Wang, Q. et al. Pliocene-Quaternary crustal melting in central and northern Tibet and insights into crustal flow. *Nat. Commun.* **7**, doi:10.1038/ncomms11888 (2016).
20. Gao, Y. et al. Adakitic rocks from slab melt-modified mantle sources in the continental collision zone of southern Tibet. *Lithos* **119**, 651–663 (2010)
21. Chung, S.-L. et al. Adakites from continental collision zones: Melting of thickened lower crust beneath southern Tibet. *Geology* **31**, 1021–1024 (2003).
22. Yang, Z.-M., Lu, Y.-J., Hou, Z.-Q. & Chang, Z.-S. High-Mg Diorite from Qulong in Southern Tibet: Implications for the Genesis of Adakite-like Intrusions and Associated Porphyry Cu Deposits in Collisional Orogens. *J. Petrol.* **56**, 227–254 (2015).
23. Chen, J.-L. et al. Geochemical variations in Miocene adakitic rocks from the western and eastern Lhasa terrane: Implications for lower crustal flow beneath the Southern Tibetan Plateau. *Lithos* **125**, 928–939 (2011).
24. Xu, W.-C., Zhang, H.-F., Guo, L. & Yuan, H.-L. Miocene high Sr/Y magmatism, south Tibet: Product of partial melting of subducted Indian continental crust and its tectonic implication. *Lithos* **114**, 293–306 (2010).
25. Williams, H. M., Turner, S. P., Pearce, J. A., Kelley, S. P. & Harris, N. B. W. Nature of the Source Regions for Post-collisional, Potassic Magmatism in Southern and Northern Tibet from Geochemical Variations and Inverse Trace Element Modelling. *J. Petrol.* **45**, 555–607 (2004).
26. McDonough, W. F. & Sun, S-S. The composition of the Earth. *Chem. Geol.* **120**, 223–253 (1995).
27. Chapman, J. B., Ducea, M. N., Profeta, L. & DeCelles, P. G. Tracking changes in crustal thickness during orogenic evolution with Sr/Y; an example from the Western U.S. Cordillera. *Geology* **43**, 919–923 (2015).
28. Profeta, L., et al. Quantifying crustal thickness over time in magmatic arcs: *Sci. Rep.* **5**, doi:10.1038/srep17786 (2015).

PART II
(Qinling Data Compilation)

Supplementary Figures

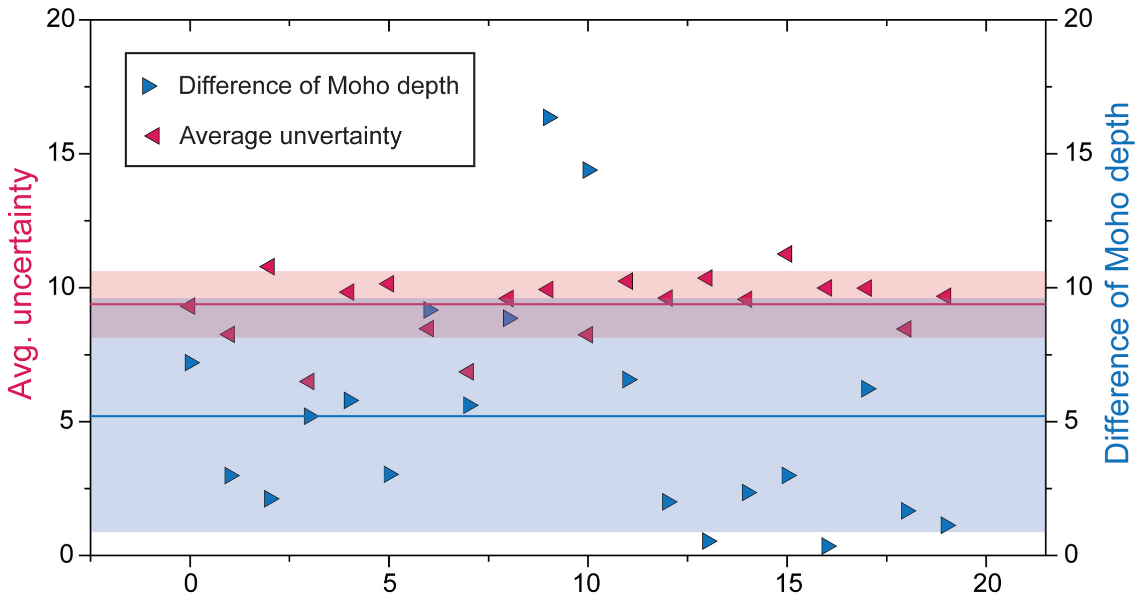

Figure S4. Plot of average uncertainty and difference between Moho depth calculated from Sr/Y and $(La/Yb)_N$ in Qinling Orogenic Belt. Red triangles are average uncertainty of Moho depth of data subsets. Blue triangles are difference of Moho depth of data subsets. Each are displayed the mean and 2 S.D. confident level.

Supplementary Tables

Table S7. Data used to plot histogram of age and probability distribution of intrusions from Qinling Orogenic Belt.

Location	Intrusion	Sample	Longitude	Latitude	Age (Ma)	1 σ	Reference
SQB	Shahewan	S7	109.70	33.75	210	1	Wang et al., (2011)[1]
SQB	Shahewan	14QL36-1	109.68	33.77	214	1	Hu et al., (2016)[2]
SQB	Caoping	14QL34-2	109.43	33.75	215	1	Hu et al., (2016)[2]
SQB	Caoping	CP-1	109.50	33.78	224	1	Gong et al., (2009)[3]
SQB	Caoping	HYS-2	109.50	33.78	220	1	Jiang et al., (2010)[4]
SQB	Zhashui	14QL39-1	109.17	33.7	200	1	Hu et al., (2017)[5]
SQB	Zhashui	ZS-1	109.25	33.63	225	1	Gong et al., (2009)[3]
SQB	Dongjiangkou	14QL38-1	108.77	33.63	217	1	Hu et al., (2017)[5]
SQB	Dongjiangkou	ZS03-01	109.03	33.63	211	2	Yang et al., (2009)[6]
SQB	Dongjiangkou	DJK-YW-1	108.98	33.75	221	1	Qin et al., (2010)[7]
SQB	Yanzhiba	08JK14-1	108.50	33.42	222	1	Yang et al., (2012)[8]
SQB	Yanzhiba	08XY1-13	108.50	33.42	209	1	Yang et al., (2012)[8]
SQB	Yanzhiba	10ns-07	108.43	33.31	201	1	Dong et al., (2012)[9]
SQB	Laocheng	08NS03-1	108.35	33.50	221	1	Yang et al., (2011)[10]
SQB	Laocheng	08NS09-1	108.35	33.50	216	1	Yang et al., (2011)[10]
SQB	Laocheng	08NS05-1	108.35	33.50	210	1	Yang et al., (2011)[10]
SQB	Wulong	WL-22-01	108.25	33.50	233	1	Qin et al., (2013)[11]
SQB	Wulong	WL-14-04	108.25	33.50	227	1	Qin et al., (2013)[11]
SQB	Wulong	WL-4-02	108.25	33.50	218	1	Qin et al., (2013)[11]
SQB	Wulong	WL-16-08	108.25	33.50	207	1	Qin et al., (2013)[11]
SQB	Longcaoping	NFP01-3	107.98	33.75	217	1	Yang et al., (2012)[8]
SQB	Longcaoping	NFP-07	107.96	33.67	189	2	Yang et al., (2012)[8]
SQB	Longcaoping	FP-05	107.97	33.67	207	1	Yang et al., (2012)[8]
SQB	Xichahe	XH-03	107.75	33.47	212	1	Qin et al., (2008)[12]
SQB	Huayang	HYG01-2	107.38	33.55	214	1	unpublished data
SQB	Huayang	HYG02-1	107.38	33.55	198	1	unpublished data
SQB	Huayang	HYG03-1	107.38	33.55	194	1	unpublished data
SQB	Huayang	16HY02-1	107.55	33.56	210	1	unpublished data
SQB	Huayang	16HY04-1	107.53	33.6	206	1	unpublished data

Location	Intrusion	Sample	Longitude	Latitude	Age (Ma)	1 σ	Reference
SQB	Huayang	14HY02-3	107.35	33.67	202	1	unpublished data
SQB	Xiba	XB01-2	107.20	33.67	219	1	Zhang et al., (2012)[13]
SQB	Taoyuanpu	13LB04-8	106.82	33.69	216	1	unpblished data
SQB	Liuba	13LB02-5	106.93	33.61	211	1	unpblished data
SQB	Huoshaodian	13QL19-6	106.93	33.53	215	1	Deng et al., (2016)[14]
SQB	Erdaohexiang	13MX10-7	106.85	33.29	202	1	unpblished data
SQB	Guangtoushan	13QL14-1	106.67	33.43	224	1	Deng et al., (2016)[14]
SQB	Guangtoushan	13QL14-3	106.67	33.43	218	1	Deng et al., (2016)[14]
SQB	Jiangjiaping	13QL07-1	106.37	33.48	215	1	Deng et al., (2016)[14]
SQB	Zhangjiaba	13QL10-2	106.34	33.45	230	1	Deng et al., (2016)[14]
SQB	Xinyuan	13QL21-2	106.23	33.52	229	1	Deng et al., (2016)[14]
SQB	Miba	518-3489	105.83	33.50	211	1	Sun et al., (2002)[15]
SQB	Mishuling	MSL01	105.75	34.03	215	1	Li et al., (2013)[16]
SQB	Huangzhuguan	H-03	105.65	33.95	214	1	Wang et al., (2011)[17]
SQB	Wenquan	A5	105.25	34.60	225	2	Cai et al., (2011)[18]
SQB	Wenquan	W23-5	105.25	34.60	216	1	Zhu et al., (2011)[19]
SQB	Wuchaba/Zhongchuan	DPC12–01	105.05	34.28	219	1	Kong et al., (2017)[20]
SQB	Zhongchuan	QS-5	105.00	34.33	236	1	Zhu et al., (2013)[21]
SQB	Luchuba	SEB12–01	105.00	34.08	211	1	Kong et al., (2017)[20]
SQB	Lüjing	LJ02/1B	104.57	34.54	221	1	Wang et al., (2016)[22]
SQB	Meiwu	SLG-2	103.12	35.04	243	2	Luo et al., (2015)[23]
SQB	Daerzang	D49	102.85	35.17	238	2	Jin et al., (2005)[24]
SQB	Xiahe	XH11-11GD	102.67	35.22	244	1	Wei et al., (2013)[25]
SQB	Xiahe	XH11-18GD	102.67	35.22	248	1	Wei et al., (2013)[25]
SQB	Xiekeng	XK-8	102.45	35.56	242	1	Luo et al., (2012)[26]
SQB	Shuangpenxi	SPX-6	102.33	35.56	242	2	Luo et al., (2012)[26]
SQB	Tongren	TR2-3	101.88	35.45	241	1	Li et al., (2015)[27]
SQB	Maixiu	ZK10-1	101.83	35.27	234	2	Li et al., (2013)[28]
SQB	Xiaguanfang	XGF-14	109.85	33.63	143	1	Wu et al., (2013)[29]
SQB	Yuanzijie	YZJ-20	109.80	33.62	142	1	Wu et al., (2013)[29]
SQB	Xiaohekou	XHK-3	109.63	33.59	150	1	Wu et al., (2013)[29]
SQB	Wagou	WG-1	109.63	33.59	145	1	Wu et al., (2013)[29]

Location	Intrusion	Sample	Longitude	Latitude	Age (Ma)	1 σ	Reference
SQB	Yuanjiagou	YJG-1	109.63	33.59	145	1	Wu et al., (2013)[29]
SQB	Baishagou	BSG-10	109.61	33.50	143	1	Wu et al., (2013)[29]
SQB	Chigou	CGV-1	109.61	33.50	144	1	Wu et al., (2013)[29]
SQB	Tudigou	TDG-3	109.61	33.50	145	1	Wu et al., (2013)[29]
SQB	Lengshuigou	LSG-10	109.62	33.43	142	1	Wu et al., (2013)[29]
NQB	Cuihuashan	FYK-1	108.85	33.95	227	2	Jiang et al., (2010)[4]
NQB	Qinlingliang	QS2	107.20	34.25	210	1	Wang et al., (2011)[1]
NQB	Laojunshan	LS1	107.67	34.15	214	3	Wang et al., (2011)[1]
NQB	Baoji	08BJ04-1	107.22	34.23	210	1	unpublished data
NQB	Baoji	08BJ10-1	107.22	34.23	216	1	unpublished data
NQB	Taibai	TB04/01	107.75	33.88	214	1	Lü et al., (2014)[30]
NQB	Mangling	ML-37/1B	110.58	33.85	157	1	Yang et al., (2014)[31]
NQB	Mangling	ML-28/1B	110.58	33.85	148	1	Yang et al., (2014)[31]
NQB	Mangling	ML-32/1B	110.58	33.85	144	1	Yang et al., (2014)[31]
NQB	Mangling	09CL264	110.30	33.8	161	1	Qin et al., (2012)[32]
NQB	Muhuguan	MHG30-1	109.50	34.00	149	1	Liu et al., (2014)[33]
NQB	Taibai	ZG237	107.75	34.08	153	1	Zhang et al., (2014)[34]

Notes: SQB - South Qinling Belt; NQB - North Qinling Belt.

Table S8. Data used to calcuate Sr/Y and La/Ybn for granitoids data subsets of Qinling Orogenic Belt.

Location	Intrusion	Sample	Longitude	Latitude	Age (Ma)	error	Reference	SiO2(wt.%)	MgO(wt.%)	Na2O(wt.%)	K2O(wt.%)	Rb(ppm)	Sr(ppm)	Y(ppm)	La(ppm)	Yb(ppm)	Rb/Sr(*)	Sr/Y	Rb/Sr(**)	La/Ybn
SQB	Shahewan	S7	109.70	33.75	210	2	Wang et al., (2011)[1]	64.20	2.20	3.84	4.18	110	675	13.50	34.80	1.26	0.16	50.00	0.16	18.76
SQB	Shahewan	B11	109.70	33.75	210	2	Wang et al., (2011)[1]	64.00	2.20	4.50	5.00	113	675	11.90	35.10	1.08	0.17	56.72	0.17	22.08
SQB	Shahewan	96S1	109.70	33.75	210	2	Wang et al., (2011)[1]	67.00	2.04	4.13	4.08	88	360	11.40	49.40	1.29	0.24	31.58	0.24	26.01
SQB	Shahewan	96S5	109.70	33.75	210	2	Wang et al., (2011)[1]	65.38	2.15	4.24	4.14	100	430	14.30	50.40	1.36	0.23	30.07	0.23	25.17
SQB	Shahewan	96S7	109.70	33.75	210	2	Wang et al., (2011)[1]	64.18	2.16	4.18	4.51	101	460	13.80	42.30	1.25	0.22	33.33	0.22	22.99
SQB	Shahewan	B32	109.70	33.75	210	2	Wang et al., (2011)[1]	65.40	2.37	4.70	4.45	101	660	13.90	35.70	1.26	0.15	47.48	0.15	19.25
SQB	Shahewan	B23	109.70	33.75	210	2	Wang et al., (2011)[1]	70.00	1.00	5.00	4.62	126	594	10.20	25.10	0.90	0.21	58.24	0.21	18.95
SQB	Shahewan	08SL1-4	109.83	33.77	215	1	Hu et al., (2016)[2]	66.33	2.20	5.08	3.54	80	642	11.00	31.00	1.34	0.12	58.36	0.12	15.72
SQB	Shahewan	08SL1-6	109.80	33.75	215	1	Hu et al., (2016)[2]	64.23	3.53	4.23	4.28	120	706	13.50	38.00	1.30	0.17	52.30	0.17	19.86
SQB	Shahewan	08SL1-7	109.80	33.75	215	1	Hu et al., (2016)[2]	63.76	3.58	4.43	4.16	107	686	14.30	38.00	1.45	0.16	47.97	0.16	17.80
SQB	Shahewan	08SL1-8	109.80	33.74	215	1	Hu et al., (2016)[2]	63.84	3.12	4.71	4.02	96	787	15.70	37.00	1.71	0.12	50.13	0.12	14.70
SQB	Shahewan	12SHW01-1	109.72	33.78	214	1	Hu et al., (2016)[2]	69.58	1.53	4.55	2.86	149	612	11.00	32.00	0.92	0.24	55.64	0.24	23.63
SQB	Shahewan	12SHW03-1	109.72	33.78	214	1	Hu et al., (2016)[2]	68.19	1.81	4.93	3.28	127	691	13.00	34.00	1.11	0.18	53.15	0.18	20.81
SQB	Shahewan	12SHW04-1	109.71	33.78	214	1	Hu et al., (2016)[2]	68.00	2.30	3.89	2.88	129	713	15.50	38.00	1.29	0.18	46.00	0.18	20.01
SQB	Shahewan	12SHW07-1	109.70	33.78	214	1	Hu et al., (2016)[2]	67.44	2.52	3.35	3.77	98	562	12.40	30.00	1.05	0.17	45.32	0.17	19.41
SQB	Shahewan	12SHW09-1	109.68	33.75	214	1	Hu et al., (2016)[2]	66.60	2.67	3.17	2.94	130	823	11.80	32.00	0.98			0.16	22.18
SQB	Shahewan	12SHW11-1	109.68	33.75	214	1	Hu et al., (2016)[2]	67.71	2.18	3.03	3.23	131	849	15.50	41.00	1.27	0.15	54.77	0.15	21.93
SQB	Shahewan	12SHW14-1	109.67	33.73	214	1	Hu et al., (2016)[2]	68.04	2.52	3.10	2.67	137	645	14.10	39.00	1.16	0.21	45.74	0.21	22.84
SQB	Shahewan	14QL35-3	109.67	33.73	214	1	Hu et al., (2016)[2]	68.12	2.06	3.72	4.98	146	571	14.80	38.00	1.38	0.26	38.58	0.26	18.71
SQB	Shahewan	14QL35-4	109.67	33.76	214	1	Hu et al., (2016)[2]	66.37	2.64	3.38	4.78	137	725	19.20	46.00	1.62	0.19	37.76	0.19	19.29
SQB	Shahewan	14QL35-5	109.68	33.77	214	1	Hu et al., (2016)[2]	66.06	2.50	4.05	4.58	128	822	16.60	41.00	1.43	0.16	49.52	0.16	19.48
SQB	Shahewan	14QL35-7	109.68	33.77	214	1	Hu et al., (2016)[2]	66.78	2.73	3.76	4.27	119	724	19.60	47.00	1.68	0.16	36.94	0.16	19.00
SQB	Shahewan	14QL36-1	109.68	33.77	214	1	Hu et al., (2016)[2]	66.06	2.67	4.29	3.72	125	659	17.60	45.00	1.47	0.19	37.44	0.19	20.80
SQB	Shahewan	14QL36-2	109.68	33.77	214	1	Hu et al., (2016)[2]	64.81	3.07	4.08	3.59	114	790	23.00	49.00	1.96	0.14	34.35	0.14	16.98
SQB	Shahewan	SHW-1	109.70	33.75	212	1	Gong et al., (2009)[3]	66.94	2.23	3.95	4.27	122	767	15.73	37.39	1.23	0.16	48.76	0.16	20.65
SQB	Shahewan	SHW-2	109.70	33.75	212	1	Gong et al., (2009)[3]	65.96	2.35	3.84	4.32	122	748	17.26	37.98	1.42	0.16	43.34	0.16	18.17
SQB	Shahewan	SHW-3	109.70	33.75	212	1	Gong et al., (2009)[3]	66.51	2.46	3.68	4.07	115	634	18.50	39.98	1.45	0.18	34.27	0.18	18.73
SQB	Shahewan	SHW-4	109.70	33.75	212	1	Gong et al., (2009)[3]	65.82	2.41	3.79	4.40	113	755	20.03	41.88	1.63	0.15	37.69	0.15	17.45
SQB	Shahewan	SHW-5	109.70	33.75	212	1	Gong et al., (2009)[3]	65.77	2.39	3.78	4.38	113	766	19.44	41.06	1.56	0.15	39.40	0.15	17.88
SQB	Caoping	08SL1-23	109.55	33.80	215	1	Hu et al., (2016)[2]	62.49	3.95	4.21	3.67	89	872	16.10	31.00	1.89			0.10	11.14
SQB	Caoping	08SL1-24	109.55	33.80	215	1	Hu et al., (2016)[2]	61.62	4.13	3.96	3.70	96	615	20.00	40.00	2.06	0.16	30.75	0.16	13.19
SQB	Caoping	08SL1-25	109.55	33.80	215	1	Hu et al., (2016)[2]	61.14	4.09	4.30	3.93	97	665	21.00	39.00	2.02	0.15	31.67	0.15	13.12
SQB	Caoping	08SL1-26	109.55	33.80	215	1	Hu et al., (2016)[2]	60.11	4.88	4.45	3.87	96	679	19.40	35.00	1.91	0.14	35.00	0.14	12.45
SQB	Caoping	08SL1-27	109.55	33.80	215	1	Hu et al., (2016)[2]	61.81	4.22	4.06	3.85	90	666	18.00	35.00	1.83	0.14	37.00	0.14	12.99
SQB	Caoping	08SL1-28	109.55	33.80	215	1	Hu et al., (2016)[2]	66.68	2.22	4.37	4.28	124	531	12.80	30.00	1.52	0.23	41.48	0.23	13.41
SQB	Caoping	08SL1-29	109.55	33.80	215	1	Hu et al., (2016)[2]	66.50	2.45	4.52	3.91	109	518	12.90	31.00	1.45	0.21	40.16	0.21	14.52

Location	Intrusion	Sample	Longitude	Latitude	Age (Ma)	error	Reference	SiO2(wt.%)	MgO(wt.%)	Na2O(wt.%)	K2O(wt.%)	Rb(ppm)	Sr(ppm)	Y(ppm)	La(ppm)	Yb(ppm)	Rb/Sr(*)	Sr/Y	Rb/Sr(**)	La/Ybn
SQB	Caoping	14QL34-2	109.43	33.75	215	1	Hu et al., (2016)[2]	68.45	1.94	3.67	4.07	120	528	18.50	36.00	1.80	0.23	28.54	0.23	13.59
SQB	Caoping	14QL34-4	109.43	33.75	215	1	Hu et al., (2016)[2]	68.54	1.92	3.90	3.97	121	528	18.50	38.00	1.82	0.23	28.54	0.23	14.18
SQB	Caoping	08SL1-30	109.54	33.75	216	1	Hu et al., (2016)[2]	70.78	0.70	4.61	4.97	184	346	10.50	41.00	0.89	0.53	32.95		
SQB	Caoping	08SL1-31	109.54	33.75	216	1	Hu et al., (2016)[2]	71.88	0.83	4.45	4.78	182	619	8.78	28.00	0.90			0.29	21.13
SQB	Caoping	12CP14-1	109.54	33.77	216	1	Hu et al., (2016)[2]	71.37	0.64	5.73	4.82	158	290	7.57	18.00	0.58	0.54	38.31	0.54	21.08
SQB	Caoping	CP-1	109.50	33.78	224	1	Gong et al., (2009)[3]	64.47	2.72	3.70	3.73	99	638	23.17	40.64	2.08	0.16	27.54	0.16	13.27
SQB	Caoping	CP-2	109.50	33.78	224	1	Gong et al., (2009)[3]	64.09	3.00	3.62	3.55	100	614	25.50	41.22	2.30	0.16	24.08	0.16	12.17
SQB	Caoping	CP-3	109.50	33.78	224	1	Gong et al., (2009)[3]	68.85	1.76	3.80	3.93	120	533	15.23	31.68	1.44	0.23	35.00	0.23	14.95
SQB	Caoping	HYS-1	109.50	33.78	220	2	Jiang et al., (2010)[4]	64.82	2.78	3.72	3.80	103	569	17.10	34.30	1.72	0.18	33.27	0.18	13.55
SQB	Caoping	HYS-2	109.50	33.78	220	2	Jiang et al., (2010)[4]	67.96	2.00	3.83	4.01	111	509	16.20	33.40	1.77	0.22	31.42	0.22	12.82
SQB	Caoping	HYS-4	109.50	33.78	220	2	Jiang et al., (2010)[4]	68.93	1.75	3.75	3.94	116	470	13.90	30.90	1.61	0.25	33.81	0.25	13.04
SQB	Caoping	HYS-5	109.50	33.78	220	2	Jiang et al., (2010)[4]	66.06	2.69	3.26	4.32	111	578	17.20	32.40	1.87	0.19	33.60	0.19	11.77
SQB	Zhashui	ZS-1	109.25	33.63	225	1	Gong et al., (2009)[3]	69.61	0.98	4.12	3.69	127	454	14.14	27.37	1.28	0.28	32.11	0.28	14.53
SQB	Zhashui	ZS-2	109.25	33.63	225	1	Gong et al., (2009)[3]	71.56	0.54	3.75	4.84	180	407	9.51	26.27	0.81			0.44	22.03
SQB	Zhashui	ZS-3	109.25	33.63	225	1	Gong et al., (2009)[3]	70.47	0.78	4.12	3.72	140	426	16.97	32.39	1.59	0.33	25.10	0.33	13.84
SQB	Zhashui	14QL39-1	109.17	33.7	200	1	Hu et al., (2017)[5]	70.70	0.82	4.12	3.92	138	404	19.20	37.30	1.71	0.34	21.04	0.34	14.82
SQB	Zhashui	14QL39-2	109.17	33.68	200	1	Hu et al., (2017)[5]	70.92	0.87	4.03	3.86	130	373	16.30	34.40	1.44	0.35	22.88	0.35	16.23
SQB	Zhashui	14QL39-3	109.17	33.68	200	1	Hu et al., (2017)[5]	71.36	0.96	3.90	3.49	139	393	16.30	32.20	1.56	0.35	24.11	0.35	14.02
SQB	Zhashui	15QL12-1	109.33	33.58	201	1	Hu et al., (2017)[5]	69.65	1.42	3.55	4.37	122	364	12.50	35.70	1.15	0.34	29.12	0.34	21.09
SQB	Zhashui	15QL12-2	109.33	33.58	201	1	Hu et al., (2017)[5]	70.11	0.93	3.20	5.60	122	447	11.00	33.40	0.98	0.27	40.64	0.27	23.15
SQB	Zhashui	15QL12-3	109.33	33.58	201	1	Hu et al., (2017)[5]	69.54	1.41	3.66	4.32	125	363	15.70	43.40	1.48	0.34	23.12	0.34	19.92
SQB	Zhashui	15QL12-5	109.33	33.58	201	1	Hu et al., (2017)[5]	69.32	1.29	3.45	4.49	143	354	12.20	33.50	1.12	0.40	29.02	0.40	20.32
SQB	Zhashui	15QL12-7	109.33	33.58	201	1	Hu et al., (2017)[5]	71.44	0.62	3.58	4.64	102	331	9.42	26.10	0.84	0.31	35.14	0.31	21.11
SQB	Zhashui	ZS01-01	109.13	33.65	199	2	Hu et al., (2017)[5]	68.33	1.35	4.04	3.40	126	592	16.00	29.20	1.34	0.21	37.00	0.21	14.80
SQB	Zhashui	ZS02-01	109.12	33.65	199	2	Hu et al., (2017)[5]	70.24	1.21	3.77	3.44	138	564	16.30	28.00	1.39	0.24	34.60	0.24	13.68
SQB	Zhashui	ZS--3	109.25	33.63	203	1	Liu et al., (2013)[35]	70.38	1.02	4.28	4.11	146	350	16.45	39.67	1.84	0.42	21.29	0.42	14.65
SQB	Zhashui	ZS--4	109.25	33.63	203	1	Liu et al., (2013)[35]	69.30	0.95	4.01	4.04	153	418	29.42	49.39	2.31	0.37	14.20	0.37	14.52
SQB	Zhashui	ZS--5	109.25	33.63	203	1	Liu et al., (2013)[35]	71.80	0.65	4.04	4.24	163	299	24.10	38.54	2.62	0.55	12.39	0.55	9.99
SQB	Dongjiangkou	DJK-1-6	108.98	33.75	220	2	Qin et al., (2010)[7]	64.88	3.35	3.50	3.75	149	734	16.70	27.80	1.63	0.20	43.95	0.20	11.59
SQB	Dongjiangkou	DJK-1-4	108.98	33.75	220	2	Qin et al., (2010)[7]	64.16	3.76	3.59	3.73	159	728	17.40	37.60	1.67	0.22	41.84	0.22	15.29
SQB	Dongjiangkou	DJK-Y-1	108.98	33.75	220	2	Qin et al., (2010)[7]	65.08	3.39	3.64	3.37	138	680	16.80	31.20	1.61	0.20	40.48	0.20	13.16
SQB	Dongjiangkou	DJK-Y-6	108.98	33.75	220	2	Qin et al., (2010)[7]	65.22	3.13	3.60	3.72	136	695	17.40	33.90	1.71	0.20	39.94	0.20	13.47
SQB	Dongjiangkou	DJK-YW-1	108.98	33.75	221	2	Qin et al., (2010)[7]	68.11	2.36	3.77	3.95	129	708	11.00	46.50	1.09	0.18	64.36	0.18	28.98
SQB	Dongjiangkou	DJK-YW-22	108.98	33.75	221	2	Qin et al., (2010)[7]	65.02	3.03	4.35	2.99	98	875	12.60	35.00	1.18	0.11	69.44	0.11	20.15

Location	Intrusion	Sample	Longitude	Latitude	Age (Ma)	error	Reference	SiO2(wt.%)	MgO(wt.%)	Na2O(wt.%)	K2O(wt.%)	Rb(ppm)	Sr(ppm)	Y(ppm)	La(ppm)	Yb(ppm)	Rb/Sr(*)	Sr/Y	Rb/Sr(**)	La/Ybn
SQB	Dongjiangkou	DJK-YW-25	108.98	33.75	221	2	Qin et al., (2010)[7]	66.73	2.35	4.34	3.10	96	929	12.30	32.00	1.18	0.10	75.53	0.10	18.42
SQB	Dongjiangkou	DJK-YW-27	108.98	33.75	221	2	Qin et al., (2010)[7]	65.41	2.54	3.96	3.73	101	900	12.50	25.10	1.19	0.11	72.00	0.11	14.33
SQB	Dongjiangkou	DJK-YW-34	108.98	33.75	221	2	Qin et al., (2010)[7]	65.52	2.96	4.14	3.10	101	927	15.70	27.10	1.46	0.11	59.04	0.11	12.61
SQB	Dongjiangkou	DJK-M-1	108.98	33.75	220	2	Qin et al., (2010)[7]	67.29	2.13	4.37	2.96	35	805	7.01	12.90	0.73			0.04	12.00
SQB	Dongjiangkou	DJK-M-12	108.98	33.75	220	2	Qin et al., (2010)[7]	65.15	2.48	4.61	3.07	82	938	10.50	28.20	0.88	0.09	89.33	0.09	21.77
SQB	Dongjiangkou	DJK-M-10	108.98	33.75	220	2	Qin et al., (2010)[7]	65.85	2.45	4.01	3.11	77	869	12.70	26.40	1.10	0.09	68.43	0.09	16.30
SQB	Dongjiangkou	DJK-M-33	108.98	33.75	220	2	Qin et al., (2010)[7]	64.32	3.09	4.08	2.96	74	913	12.50	27.10	1.10	0.08	73.04	0.08	16.74
SQB	Dongjiangkou	DJK-M-34	108.98	33.75	220	2	Qin et al., (2010)[7]	64.48	2.92	4.54	2.62	75	1002	10.90	27.60	0.97	0.07	91.93	0.07	19.33
SQB	Dongjiangkou	DJK-M-4	108.98	33.75	220	2	Qin et al., (2010)[7]	63.45	3.32	4.11	3.17	84	936	12.90	17.00	1.12	0.09	72.56	0.09	10.31
SQB	Dongjiangkou	JK-2	108.98	33.75	219	2	Jiang et al., (2010)[4]	62.83	3.17	4.23	2.41	64	939	11.70	28.80	1.21	0.07	80.26	0.07	16.17
SQB	Dongjiangkou	JK-3	108.98	33.75	219	2	Jiang et al., (2010)[4]	64.56	3.38	4.23	3.12	73	954	13.90	24.20	1.31	0.08	68.63	0.08	12.55
SQB	Dongjiangkou	JK-4	108.98	33.75	219	2	Jiang et al., (2010)[4]	64.25	3.43	4.13	3.26	84	1071	12.80	31.30	1.31	0.08	83.67	0.08	16.23
SQB	Dongjiangkou	JK-5	108.98	33.75	219	2	Jiang et al., (2010)[4]	66.44	2.98	3.70	3.81	101	814	11.90	36.00	1.34	0.12	68.40	0.12	18.25
SQB	Dongjiangkou	JK-6	108.98	33.75	219	2	Jiang et al., (2010)[4]	66.31	2.96	3.97	3.31	94	889	11.40	33.10	1.16	0.11	77.98	0.11	19.38
SQB	Dongjiangkou	JK-7	108.98	33.75	219	2	Jiang et al., (2010)[4]	67.44	2.30	3.68	3.91	129	486	13.00	40.70	1.51	0.27	37.38	0.27	18.31
SQB	Dongjiangkou	JK-8	108.98	33.75	219	2	Jiang et al., (2010)[4]	65.32	3.49	3.55	3.92	125	584	14.10	40.20	1.66	0.21	41.42	0.21	16.45
SQB	Dongjiangkou	JK-9	108.98	33.75	219	2	Jiang et al., (2010)[4]	65.08	3.78	3.67	3.91	128	590	15.70	29.40	1.71	0.22	37.58	0.22	11.68
SQB	Dongjiangkou	JK-10	108.98	33.75	219	2	Jiang et al., (2010)[4]	68.37	2.38	4.07	3.40	96	696	10.90	25.30	1.20	0.14	63.85	0.14	14.32
SQB	Dongjiangkou	14QL27-1	108.87	33.69	217	1	Hu et al., (2017)[5]	66.74	3.20	3.31	3.23	97	849	12.40	35.50	1.20	0.11	68.47	0.11	20.10
SQB	Dongjiangkou	14QL27-4	108.93	33.70	217	1	Hu et al., (2017)[5]	68.21	2.67	3.69	3.37	122	682	14.60	35.60	1.40	0.18	46.71	0.18	17.27
SQB	Dongjiangkou	14QL27-5	108.97	33.73	217	1	Hu et al., (2017)[5]	69.42	2.19	3.39	4.08	132	602	13.40	37.00	1.34	0.22	44.93	0.22	18.76
SQB	Dongjiangkou	14QL27-7	108.97	33.73	217	1	Hu et al., (2017)[5]	68.05	2.73	3.58	3.54	119	766	14.40	36.10	1.39	0.16	53.19	0.16	17.64
SQB	Dongjiangkou	14QL27-9	109.00	33.75	217	1	Hu et al., (2017)[5]	66.31	3.39	3.78	3.30	102	771	17.50	37.30	1.66	0.13	44.06	0.13	15.26
SQB	Dongjiangkou	14QL28-3	109.00	33.76	217	1	Hu et al., (2017)[5]	68.10	2.67	3.40	4.07	133	715	13.60	32.90	1.31	0.19	52.57	0.19	17.06
SQB	Dongjiangkou	14QL38-1	108.77	33.63	217	1	Hu et al., (2017)[5]	67.14	2.91	3.74	3.29	111	782	17.10	29.30	1.53	0.14	45.73	0.14	13.01
SQB	Dongjiangkou	14QL38-3	108.77	33.63	217	1	Hu et al., (2017)[5]	66.17	2.98	3.78	3.04	97	892	11.30	32.00	1.07	0.11	78.94	0.11	20.32
SQB	Dongjiangkou	14QL38-4	108.77	33.63	217	1	Hu et al., (2017)[5]	67.38	2.83	3.49	3.27	102	847	15.70	31.50	1.50	0.12	53.95	0.12	14.27
SQB	Dongjiangkou	14QL38-5	108.76	33.62	217	1	Hu et al., (2017)[5]	66.83	3.27	3.43	3.61	118	773	11.30	31.00	1.08	0.15	68.41	0.15	19.50
SQB	Dongjiangkou	14QL38-7	108.76	33.62	217	1	Hu et al., (2017)[5]	65.96	3.69	3.23	3.91	146	703	14.20	23.40	1.35	0.21	49.51	0.21	11.77
SQB	Dongjiangkou	ZS04-01	109.03	33.77	217	1	Hu et al., (2017)[5]	64.05	3.38	3.43	3.53	121	708	18.40	36.30	1.54	0.17	38.48	0.17	16.01
SQB	Dongjiangkou	ZS05-01	109.03	33.77	217	1	Hu et al., (2017)[5]	66.58	2.32	3.79	3.29	109	906	14.90	28.00	1.20	0.12	60.81	0.12	15.85
SQB	Dongjiangkou	ZS06-01	109.03	33.77	217	1	Hu et al., (2017)[5]	63.99	3.03	3.58	3.77	151	1070	18.50	39.30	1.55	0.14	57.84	0.14	17.22
SQB	Dongjiangkou	DJK-S-01	108.98	33.75	214	2	Qin et al., (2010)[7]	66.21	2.39	4.21	3.46	107	803	11.70	25.20	1.20	0.13	68.63	0.13	14.27
SQB	Dongjiangkou	DJK-S-02	108.98	33.75	214	2	Qin et al., (2010)[7]	65.56	2.42	4.24	3.53	107	837	12.80	24.50	1.31	0.13	65.39	0.13	12.70
SQB	Dongjiangkou	DJK-S-03	108.98	33.75	214	2	Qin et al., (2010)[7]	67.96	1.93	4.26	3.51	107	755	10.10	25.70	1.04	0.14	74.75	0.14	16.79
SQB	Dongjiangkou	DJK-S-04	108.98	33.75	214	2	Qin et al., (2010)[7]	68.24	2.07	3.94	3.81	122	736	10.50	24.00	1.02	0.17	70.10	0.17	15.98

Location	Intrusion	Sample	Longitude	Latitude	Age (Ma)	error	Reference	SiO2(wt.%)	MgO(wt.%)	Na2O(wt.%)	K2O(wt.%)	Rb(ppm)	Sr(ppm)	Y(ppm)	La(ppm)	Yb(ppm)	Rb/Sr(*)	Sr/Y	Rb/Sr(**)	La/Ybn
SQB	Yanzhiba	08JK12-1	108.50	33.42	222	1	Yang et al., (2012)[8]	65.30	2.22	4.24	3.22	80	714	8.72	25.00	0.74	0.11	81.88	0.11	22.95
SQB	Yanzhiba	08JK14-1	108.50	33.42	222	1	Yang et al., (2012)[8]	68.70	1.30	4.21	3.48	106	658	8.53	26.00	0.77	0.16	77.14	0.16	22.94
SQB	Yanzhiba	08JK16-1	108.50	33.42	222	1	Yang et al., (2012)[8]	69.60	1.09	4.45	3.93	122	700	7.73	24.00	0.65	0.17	90.56	0.17	25.08
SQB	Yanzhiba	08JK17-1	108.50	33.42	222	1	Yang et al., (2012)[8]	69.40	0.70	4.14	3.11	124	523	8.40	21.00	0.90	0.24	62.26	0.24	15.85
SQB	Laocheng	08NS01-1	108.35	33.50	221	2	Yang et al., (2012)[8]	57.40	2.97	3.46	3.99	187	814	11.70	30.00	1.04	0.23	69.57	0.23	19.60
SQB	Laocheng	08NS03-1	108.35	33.50	221	2	Yang et al., (2012)[8]	69.30	1.45	4.27	3.72	75	856	6.07	20.00	0.55	0.09	141.02	0.09	24.70
SQB	Laocheng	08NS07-1	108.35	33.50	221	2	Yang et al., (2012)[8]	65.40	1.97	4.71	2.81	78	1019	11.20	31.00	0.90	0.08	90.98	0.08	23.40
SQB	Laocheng	08NS08-1	108.35	33.50	221	2	Yang et al., (2012)[8]	62.20	3.05	4.25	3.10	74	884	11.20	33.00	0.90	0.08	78.93	0.08	24.91
SQB	Laocheng	NS-4	108.35	33.50	218	3	Jiang et al., (2010)[4]	64.64	2.42	3.94	3.49	104	738	11.20	31.90	1.09	0.14	65.89	0.14	19.88
SQB	Laocheng	NS-5	108.35	33.50	218	3	Jiang et al., (2010)[4]	67.16	1.55	4.15	3.97	128	682	9.80	30.00	0.95	0.19	69.59	0.19	21.45
SQB	Laocheng	NS-6	108.35	33.50	218	3	Jiang et al., (2010)[4]	70.38	0.62	4.42	3.69	99	600	5.50	32.10	0.47	0.17	109.09	0.17	46.40
SQB	Laocheng	NS-7	108.35	33.50	218	3	Jiang et al., (2010)[4]	67.86	1.61	4.64	3.10	103	789	9.50	29.50	0.91	0.13	83.05	0.13	22.02
SQB	Laocheng	NS-9	108.35	33.50	218	3	Jiang et al., (2010)[4]	66.72	1.19	4.65	3.90	100	895	7.20	39.10	0.71	0.11	124.31	0.11	37.41
SQB	Laocheng	NS-10	108.35	33.50	218	3	Jiang et al., (2010)[4]	68.55	1.43	3.20	3.90	86	671	4.30	32.90	0.56	0.13	156.05	0.13	39.91
SQB	Laocheng	NS-12	108.35	33.50	218	3	Jiang et al., (2010)[4]	68.53	1.04	4.25	2.95	127	679	6.70	35.80	0.64	0.19	101.34	0.19	38.00
SQB	Laocheng	NS-13	108.35	33.50	218	3	Jiang et al., (2010)[4]	70.18	0.89	3.94	3.00	98	656	6.40	29.70	0.47	0.15	102.50	0.15	42.93
SQB	Laocheng	08NS09-1	108.35	33.50	216	1	Yang et al., (2012)[8]	62.10	4.30	3.74	3.85	109	887	15.60	32.00	1.39	0.12	56.86	0.12	15.64
SQB	Laocheng	08NS05-1	108.35	33.50	210	2	Yang et al., (2012)[8]	68.40	0.82	4.84	3.31	79	709	4.87	31.00	0.37	0.11	145.59		
SQB	Laocheng	08NS06-1	108.35	33.50	210	2	Yang et al., (2012)[8]	68.70	0.84	4.96	2.43	69	725	4.89	25.00	0.39	0.10	148.26	0.10	43.55
SQB	Laocheng	08NS06-2	108.35	33.50	211	2	Yang et al., (2012)[8]	70.70	0.60	4.15	2.62	65	579	7.23	31.00	0.63			0.11	33.43
SQB	Wulong	WL-22-01	108.25	33.50	233	2	Qin et al., (2013)[11]	63.20	2.74	4.09	3.11	96	878	14.10	54.10	1.48	0.11	62.27	0.11	24.83
SQB	Wulong	WL-22-07	108.25	33.50	233	2	Qin et al., (2013)[11]	60.65	3.18	4.21	2.90	111	868	16.70	28.60	1.67			0.13	11.63
SQB	Wulong	WL-22-08	108.25	33.50	233	2	Qin et al., (2013)[11]	61.84	2.74	4.36	3.08	91	967	14.60	46.40	1.45	0.09	66.23	0.09	21.74
SQB	Wulong	WL-22-09	108.25	33.50	233	2	Qin et al., (2013)[11]	61.98	2.98	4.12	3.24	98	922	15.60	33.50	1.55	0.11	59.10	0.11	14.68
SQB	Wulong	WL-22-10	108.25	33.50	233	2	Qin et al., (2013)[11]	61.63	2.97	4.08	3.24	78	947	14.30	38.40	1.43	0.08	66.22	0.08	18.24
SQB	Wulong	WL-13-03	108.25	33.50	231	2	Qin et al., (2013)[11]	67.97	1.93	4.13	3.38	86	850	9.17	23.30	0.97			0.10	16.32
SQB	Wulong	WL-13-05	108.25	33.50	231	2	Qin et al., (2013)[11]	67.91	2.19	4.56	2.01	70	896	8.77	28.80	0.95			0.08	20.59
SQB	Wulong	WL-12-01	108.25	33.50	227	2	Qin et al., (2013)[11]	63.16	2.76	4.27	3.15	90	866	11.20	19.60	0.99	0.10	77.32	0.10	13.45
SQB	Wulong	WL-12-02	108.25	33.50	227	2	Qin et al., (2013)[11]	63.75	2.83	4.18	3.34	96	851	10.50	22.60	0.95	0.11	81.05	0.11	16.16
SQB	Wulong	WL-12-03	108.25	33.50	227	2	Qin et al., (2013)[11]	63.98	2.83	4.26	3.05	86	833	10.90	48.40	1.01	0.10	76.42		
SQB	Wulong	WL-12-09	108.25	33.50	227	2	Qin et al., (2013)[11]	62.78	2.99	4.25	2.96	74	792	15.80	57.80	1.45	0.09	50.13		
SQB	Wulong	WL-12-12	108.25	33.50	227	2	Qin et al., (2013)[11]	63.87	2.84	4.49	3.17	91	785	11.50	28.70	1.13	0.12	68.26	0.12	17.25
SQB	Wulong	WL-12-16	108.25	33.50	227	2	Qin et al., (2013)[11]	63.31	2.79	4.32	3.05	91	807	12.70	28.40	1.24	0.11	63.54	0.11	15.56
SQB	Wulong	WL-14-04	108.25	33.50	227	2	Qin et al., (2013)[11]	62.28	3.87	3.70	3.71	145	973	16.00	31.40	1.69	0.15	60.81	0.15	12.62

Location	Intrusion	Sample	Longitude	Latitude	Age (Ma)	error	Reference	SiO2(wt.%)	MgO(wt.%)	Na2O(wt.%)	K2O(wt.%)	Rb(ppm)	Sr(ppm)	Y(ppm)	La(ppm)	Yb(ppm)	Rb/Sr(*)	Sr/Y	Rb/Sr(**)	La/Ybn
SQB	Wulong	WL-4-02	108.25	33.50	218	2	Qin et al., (2013)[11]	70.47	0.72	3.64	4.21	160	479	11.40	23.80	1.09	0.33	42.02	0.33	14.83
SQB	Wulong	WL-4-04	108.25	33.50	218	2	Qin et al., (2013)[11]	70.65	0.70	4.00	3.58	153	456	12.20	32.80	1.20	0.34	37.38	0.34	18.57
SQB	Wulong	WL-5-03	108.25	33.50	218	2	Qin et al., (2013)[11]	70.65	0.66	3.89	3.89	165	486	11.30	32.70	0.95	0.34	43.01	0.34	23.38
SQB	Wulong	WL-5-02	108.25	33.50	218	2	Qin et al., (2013)[11]	69.16	0.81	3.94	4.20	143	567	8.83	33.90	0.75	0.25	64.21	0.25	30.71
SQB	Wulong	WL-21-01	108.25	33.50	218	2	Qin et al., (2013)[11]	71.05	0.64	4.32	3.55	106	572	7.15	24.10	0.67	0.19	80.00	0.19	24.44
SQB	Wulong	WL-21-04	108.25	33.50	218	2	Qin et al., (2013)[11]	70.43	0.65	4.88	3.24	107	560	6.80	22.90	0.65	0.19	82.35	0.19	23.93
SQB	Wulong	WL-21-05	108.25	33.50	218	2	Qin et al., (2013)[11]	69.85	0.71	4.60	3.17	108	591	7.48	26.90	0.71	0.18	79.01	0.18	25.74
SQB	Wulong	WL-21-06	108.25	33.50	218	2	Qin et al., (2013)[11]	70.82	0.68	4.46	3.30	103	573	7.98	22.50	0.74	0.18	71.80	0.18	20.66
SQB	Wulong	WL-16-08	108.25	33.50	207	2	Qin et al., (2013)[11]	70.29	0.69	4.34	3.61	111	596	6.11	30.60	0.56	0.19	97.55	0.19	37.12
SQB	Wulong	WL-16-02	108.25	33.50	207	2	Qin et al., (2013)[11]	69.38	0.64	4.27	3.96	118	669	5.10	23.50	0.43	0.18	131.18	0.18	37.13
SQB	Wulong	WL-16-05	108.25	33.50	207	2	Qin et al., (2013)[11]	69.37	0.69	4.50	3.79	121	660	5.92	30.30	0.49	0.18	111.49	0.18	42.01
SQB	Wulong	WL-30-01	108.25	33.50	207	2	Qin et al., (2013)[11]	68.82	0.85	4.71	3.14	94	650	4.62	34.90	0.34	0.14	140.69	0.14	69.73
SQB	Wulong	WL-29-17	108.25	33.50	207	2	Qin et al., (2013)[11]	68.70	1.10	4.01	3.56	119	736	7.61	56.70	0.73	0.16	96.71	0.16	52.76
SQB	Wulong	WL-30-02	108.25	33.50	207	2	Qin et al., (2013)[11]	68.01	0.98	5.20	2.28	87	748	4.94	31.70	0.37	0.12	151.42	0.12	58.20
SQB	Wulong	WL-30-03	108.25	33.50	207	2	Qin et al., (2013)[11]	67.68	1.03	4.89	2.60	97	716	5.33	29.20	0.39	0.14	134.33	0.14	50.86
SQB	Wulong	WL-30-05	108.25	33.50	207	2	Qin et al., (2013)[11]	66.91	0.96	5.01	2.95	100	744	5.17	36.00	0.36	0.13	143.91	0.13	67.93
SQB	Wulong	WL-30-06	108.25	33.50	207	2	Qin et al., (2013)[11]	68.65	0.84	5.11	2.29	97	688	6.11	29.10	0.42	0.14	112.60	0.14	47.07
SQB	Wulong	WL-30-04	108.25	33.50	207	2	Qin et al., (2013)[11]	67.95	1.10	5.01	2.25	109	802	8.17	52.20	0.70	0.14	98.16	0.14	50.66
SQB	Wulong	WL-08-13	108.25	33.50	207	2	Qin et al., (2013)[11]	69.33	0.96	4.43	2.94	116	659	7.66	32.60	0.76	0.18	86.03	0.18	29.14
SQB	Wulong	WL-08-14	108.25	33.50	207	2	Qin et al., (2013)[11]	69.09	0.94	4.40	2.84	112	648	7.32	32.60	0.74	0.17	88.52	0.17	29.93
SQB	Wulong	WL-08-16	108.25	33.50	207	2	Qin et al., (2013)[11]	69.52	0.94	4.33	3.01	113	644	7.32	30.20	0.74	0.18	87.98	0.18	27.72
SQB	Wulong	WL-08-03	108.25	33.50	207	2	Qin et al., (2013)[11]	68.56	0.87	4.70	3.23	101	791	5.34	35.80	0.49	0.13	148.13	0.13	49.63
SQB	Wulong	WL-09-03	108.25	33.50	207	2	Qin et al., (2013)[11]	68.32	1.10	4.61	3.26	111	825	9.58	43.70	0.96	0.13	86.12	0.13	30.92
SQB	Wulong	WL-02	108.25	33.50	208	2	Qin et al., (2008)[12]	68.44	0.71	4.50	2.51	100	763	4.77	32.20	0.33	0.13	159.96	0.13	66.29
SQB	Wulong	WL-04	108.25	33.50	208	2	Qin et al., (2008)[12]	65.29	1.31	5.00	2.30	86	1003	10.60	30.60	0.96	0.09	94.62	0.09	21.65
SQB	Wulong	WL-05	108.25	33.50	208	2	Qin et al., (2008)[12]	68.92	0.85	4.30	3.90	115	663	7.27	24.70	0.63	0.17	91.20	0.17	26.63
SQB	Wulong	WL-10	108.25	33.50	208	2	Qin et al., (2008)[12]	71.30	0.76	4.30	3.30	99	643	6.98	20.80	0.52	0.15	92.12	0.15	27.17
SQB	Wulong	WL-13	108.25	33.50	208	2	Qin et al., (2008)[12]	70.89	0.86	4.30	3.10	97	715	7.79	29.00	0.62	0.14	91.78	0.14	31.77
SQB	Wulong	WL-15	108.25	33.50	208	2	Qin et al., (2008)[12]	70.54	0.85	4.00	3.30	97	647	11.20	26.20	0.82	0.15	57.77	0.15	21.71
SQB	Wulong	WL-18	108.25	33.50	208	2	Qin et al., (2008)[12]	66.04	1.24	4.80	2.30	81	1115	8.36	49.90	0.63	0.07	133.37	0.07	53.81
SQB	Wulong	WL-21	108.25	33.50	208	2	Qin et al., (2008)[12]	63.84	1.26	4.70	2.60	55	1066	8.39	39.30	0.65	0.05	127.06	0.05	41.07
SQB	Longcaoping	NFP01-1	107.98	33.75	217	2	Dong et al., (2012)[9]	56.64	3.68	3.51	2.40	78	608	25.80	28.30	2.20	0.13	23.57	0.13	8.74
SQB	Longcaoping	NFP01-2	107.98	33.75	217	2	Dong et al., (2012)[9]	56.06	3.36	3.70	2.55	79	647	31.00	33.90	2.49	0.12	20.87	0.12	9.25
SQB	Longcaoping	NFP01-3	107.98	33.75	217	2	Dong et al., (2012)[9]	55.20	3.08	3.65	2.53	72	822	28.90	37.70	2.63	0.09	28.44	0.09	9.74
SQB	Longcaoping	NFP01-4	107.98	33.75	217	2	Dong et al., (2012)[9]	56.55	3.79	3.39	2.17	60	649	33.30	44.40	2.99	0.09	19.49	0.09	10.09
SQB	Longcaoping	NFP01-5	107.98	33.75	217	2	Dong et al., (2012)[9]	57.10	3.53	3.53	2.27	60	594	32.10	43.00	3.05	0.10	18.50	0.10	9.58

Location	Intrusion	Sample	Longitude	Latitude	Age (Ma)	error	Reference	SiO2(wt.%)	MgO(wt.%)	Na2O(wt.%)	K2O(wt.%)	Rb(ppm)	Sr(ppm)	Y(ppm)	La(ppm)	Yb(ppm)	Rb/Sr(*)	Sr/Y	Rb/Sr(**)	La/Ybn
SQB	Longcaoping	NFP01-6	107.98	33.75	217	2	Dong et al., (2012)[9]	57.69	2.80	3.68	2.54	75	682	31.30	26.70	2.85	0.11	21.79	0.11	6.36
SQB	Longcaoping	NFP-07	107.96	33.67	189	3	Dong et al., (2012)[9]	69.45	1.10	4.51	2.62	142	569	23.70	29.70	1.93	0.25	24.01	0.25	10.45
SQB	Longcaoping	NFP-08	107.96	33.67	189	3	Dong et al., (2012)[9]	69.50	1.11	5.18	2.63	168	515	25.30	42.60	2.11	0.33	20.36	0.33	13.72
SQB	Longcaoping	NFP-09	107.96	33.67	189	3	Dong et al., (2012)[9]	70.33	1.00	4.78	2.28	165	475	19.70	37.40	1.61	0.35	24.11	0.35	15.78
SQB	Longcaoping	NFP-10	107.96	33.67	189	3	Dong et al., (2012)[9]	69.81	1.08	4.69	2.43	147	525	18.90	29.50	1.67	0.28	27.78	0.28	12.00
SQB	Longcaoping	NFP-11	107.96	33.67	189	3	Dong et al., (2012)[9]	68.09	1.28	4.16	2.95	115	732	17.30	34.70	1.37			0.16	17.21
SQB	Longcaoping	NFP-12	107.96	33.67	189	3	Dong et al., (2012)[9]	67.07	1.42	4.19	2.90	89	769	19.20	35.50	1.49			0.12	16.19
SQB	Longcaoping	NFP-13	107.96	33.67	189	3	Dong et al., (2012)[9]	71.48	0.63	3.95	3.84	157	395	16.60	45.60	1.28	0.40	23.80	0.40	24.20
SQB	Xichahe	XH-03	107.75	33.47	212	2	Qin et al., (2008)[12]	62.30	3.15	4.00	2.20	90	907	13.40	30.80	1.04	0.10	67.69	0.10	20.12
SQB	Xichahe	XH-04	107.75	33.47	212	2	Qin et al., (2008)[12]	61.75	3.05	4.40	2.21	86	900	10.60	24.80	0.79	0.10	84.91	0.10	21.33
SQB	Xichahe	XH-05	107.75	33.47	212	2	Qin et al., (2008)[12]	64.40	2.55	4.00	2.70	84	903	10.30	24.20	0.83	0.09	87.67	0.09	19.81
SQB	Xichahe	XH-06	107.75	33.47	212	2	Qin et al., (2008)[12]	60.91	3.99	4.20	2.60	104	837	16.10	21.60	1.27	0.12	51.99	0.12	11.55
SQB	Xichahe	XH-07	107.75	33.47	212	2	Qin et al., (2008)[12]	60.03	3.42	4.10	2.70	104	865	14.20	17.10	1.17	0.12	60.92	0.12	9.93
SQB	Xichahe	XH-10	107.75	33.47	212	2	Qin et al., (2008)[12]	60.83	4.18	4.00	2.90	140	821	18.30	31.50	1.49	0.17	44.86	0.17	14.36
SQB	Xichahe	XH-12	107.75	33.47	212	2	Qin et al., (2008)[12]	60.23	5.99	3.40	2.00	79	1029	22.10	20.10	2.15	0.08	46.56	0.08	6.35
SQB	Xichahe	XH-14	107.75	33.47	212	2	Qin et al., (2008)[12]	58.65	6.00	3.80	2.00	67	1253	22.70	27.40	2.13	0.05	55.20	0.05	8.74
SQB	Xichahe	XH-15	107.75	33.47	212	2	Qin et al., (2008)[12]	59.89	4.02	3.10	3.20	132	942	16.60	38.90	1.51	0.14	56.75	0.14	17.50
SQB	Huayang	HYG01-2	107.38	33.55	214	1	unpublished data	62.60	2.86	3.32	3.79	119	443	12.80	32.40	1.46	0.27	34.61	0.27	15.08
SQB	Huayang	HYG05-1	107.38	33.55	214	1	unpublished data	62.30	2.43	5.29	3.44	214	195	35.10	52.40	3.94	1.10	5.56	1.10	9.03
SQB	Huayang	HYG10-1	107.38	33.55	209	3	unpublished data	67.10	0.93	4.97	3.06	99	596	6.85	22.90	0.69	0.17	87.01	0.17	
SQB	Huayang	14HY02-1	107.35	33.67	210	2	unpublished data	61.27	1.85	4.31	2.63	85	949	7.08	42.70	0.63	0.09	134.04	0.09	
SQB	Huayang	15HY03-2	107.67	33.53	207	2	unpublished data	68.28	1.16	4.27	4.22	124	269	10.80	30.20	0.65	0.46	24.91	0.46	31.56
SQB	Huayang	16HY02-1	107.55	33.56	207	2	unpublished data	68.95	0.74	4.00	4.49	133	385	12.10	55.50	0.89	0.35	31.82		
SQB	Huayang	16HY06-3	107.38	33.62	207	2	unpublished data	64.44	1.40	4.84	2.71	128	655	9.20	30.80	0.73	0.20	71.20	0.20	28.66
SQB	Huayang	HYG10-2	107.38	33.55	207	1	unpublished data	69.30	0.70	4.23	3.98	110	454	8.67	42.40	0.90	0.24	52.36	0.24	32.00
SQB	Huayang	HYG03-1	107.38	33.55	194	1	unpublished data	64.30	1.55	4.92	1.84	75	719	25.50	56.70	2.56	0.10	28.20	0.10	15.05
SQB	Huayang	HYG09-1	107.38	33.55	196	1	unpublished data	66.80	1.10	4.50	2.03	127	412	9.42	21.20	1.13	0.31	43.74	0.31	12.74
SQB	Xiba	XB01-1	107.20	33.67	219	1	Zhang et al., (2012)[13]	61.55	3.22	3.37	3.10	146	592	19.90	43.10	1.63	0.25	29.75	0.25	17.96
SQB	Xiba	XB01-2	107.20	33.67	219	1	Zhang et al., (2012)[13]	65.14	2.44	3.30	3.70	142	506	15.50	34.40	1.38	0.28	32.65	0.28	16.93
SQB	Xiba	XB01-3	107.20	33.67	219	1	Zhang et al., (2012)[13]	56.10	4.89	3.45	2.17	121	718	21.80	27.50	1.79	0.17	32.94	0.17	10.44
SQB	Xiba	XB02-1	107.20	33.67	219	1	Zhang et al., (2012)[13]	64.68	2.33	3.53	3.14	130	642	21.80	40.90	1.69	0.20	29.45	0.20	16.44
SQB	Xiba	XB03-1	107.20	33.67	219	1	Zhang et al., (2012)[13]	69.62	0.97	3.78	4.58	188	404	15.50	45.00	1.22	0.47	26.06	0.47	25.06

Location	Intrusion	Sample	Longitude	Latitude	Age (Ma)	error	Reference	SiO2(wt.%)	MgO(wt.%)	Na2O(wt.%)	K2O(wt.%)	Rb(ppm)	Sr(ppm)	Y(ppm)	La(ppm)	Yb(ppm)	Rb/Sr(*)	Sr/Y	Rb/Sr(**)	La/Ybn
SQB	Xiba	XB04-1	107.20	33.67	219	1	Zhang et al., (2012)[13]	69.86	0.88	3.67	4.83	179	364	14.50	43.00	1.17	0.49	25.10	0.49	24.97
SQB	Xiba	XB05-1	107.20	33.67	219	1	Zhang et al., (2012)[13]	64.44	2.16	4.44	2.70	101	702	13.10	27.30	1.15	0.14	53.59	0.14	16.13
SQB	Xiba	XB06-1	107.20	33.67	218	1	Zhang et al., (2012)[13]	65.60	2.08	4.07	2.77	121	740	13.90	27.80	1.18	0.16	53.24	0.16	16.00
SQB	Xiba	XB07-1	107.20	33.67	218	1	Zhang et al., (2012)[13]	71.10	1.22	3.12	4.52	195	410	8.88	28.50	0.85	0.48	46.17	0.48	22.78
SQB	Xiba	XB08-1	107.20	33.67	218	1	Zhang et al., (2012)[13]	64.04	2.45	3.81	2.94	116	746	16.10	27.70	1.34	0.16	46.34	0.16	14.04
SQB	Xiba	XB09-2	107.20	33.67	218	1	Zhang et al., (2012)[13]	62.29	2.84	3.31	3.04	134	654	18.30	31.00	1.36	0.20	35.74	0.20	15.48
SQB	Xiba	XB10-1	107.20	33.67	218	1	Zhang et al., (2012)[13]	64.35	2.39	4.92	3.28	117	674	12.60	31.00	1.10	0.17	53.49	0.17	19.14
SQB	Xiba	XB11-1	107.20	33.67	218	1	Zhang et al., (2012)[13]	64.12	2.43	4.52	3.55	138	770	11.60	24.90	0.98	0.18	66.38	0.18	17.26
SQB	Xiba	XB12-1	107.20	33.67	218	1	Zhang et al., (2012)[13]	65.57	2.34	3.56	3.18	116	858	11.90	23.50	1.11	0.14	72.10	0.14	14.38
SQB	Taoyuanpu	13LB04-5	106.82	33.69	216	1	unpblished data	67.69	1.80	3.73	3.16	96	539	10.24	22.72	1.15	0.18	52.62	0.18	13.47
SQB	Taoyuanpu	13LB04-7	106.82	33.69	216	1	unpblished data	68.63	1.64	3.63	4.06	95	554	7.74	24.82	0.83	0.17	71.51	0.17	20.22
SQB	Taoyuanpu	13LB04-8	106.82	33.69	216	1	unpblished data	64.69	2.48	3.86	2.79	93	634	13.45	32.78	1.49	0.15	47.13	0.15	14.99
SQB	Taoyuanpu	13LB04-10	106.83	33.68	216	1	unpblished data	62.73	4.52	3.05	2.69	94	502	13.33	22.54	1.47	0.19	37.64	0.19	10.43
SQB	Taoyuanpu	13LB04-11	106.85	33.69	216	1	unpblished data	61.97	5.25	2.93	2.70	95	467	15.47	26.08	1.71	0.20	30.18	0.20	10.34
SQB	Liuba	13LB02-5	106.93	33.61	211	1	unpblished data	63.59	2.54	7.53	3.04	92	767	12.94	19.40	1.36	0.12	59.30	0.12	9.68
SQB	Liuba	13LB02-6	106.93	33.58	211	1	unpblished data	59.82	5.98	3.07	1.99	55	679	19.09	32.22	2.11	0.08	35.59	0.08	10.39
SQB	Huoshaodian	13QL19-3	106.92	33.54	215	1	Deng et al., (2016)[14]	65.46	2.88	3.08	3.47	100	545	11.42	37.26	1.23	0.18	47.76	0.18	20.51
SQB	Huoshaodian	13QL19-4	106.93	33.53	215	1	Deng et al., (2016)[14]	60.16	4.65	3.44	2.60	66	701	12.12	31.16	1.31	0.09	57.85	0.09	16.21
SQB	Huoshaodian	13QL19-5	106.93	33.53	215	1	Deng et al., (2016)[14]	58.37	5.77	2.90	2.26	63	621	14.68	38.00	1.68	0.10	42.29	0.10	15.38
SQB	Huoshaodian	13QL19-6	106.93	33.53	215	1	Deng et al., (2016)[14]	62.42	4.11	3.35	2.72	78	639	12.53	31.82	1.40	0.12	50.99	0.12	15.44
SQB	Huoshaodian	13QL19-7	106.93	33.53	215	1	Deng et al., (2016)[14]	60.34	4.77	3.30	1.85	74	681	11.94	25.48	1.33	0.11	56.99	0.11	12.99
SQB	Guangtoushan	13QL13-1	106.68	33.45	224	1	Deng et al., (2016)[14]	71.84	0.57	4.17	3.19	95	784	5.81	15.76	0.54	0.12	134.87	0.12	19.97
SQB	Guangtoushan	13QL14-1	106.67	33.43	224	1	Deng et al., (2016)[14]	71.35	0.85	4.07	2.46	108	716	8.16	25.34	0.72	0.15	87.70	0.15	23.98
SQB	Guangtoushan	13QL15-3	106.63	33.33	224	1	Deng et al., (2016)[14]	71.83	0.52	4.24	2.46	85	758	6.35	16.69	0.77	0.11	119.30	0.11	14.80
SQB	Guangtoushan	13MX02-6	106.57	33.38	224	1	Deng et al., (2016)[14]	71.55	0.65	5.02	4.30	136	403	18.51	39.24	1.81	0.34	21.78	0.34	14.69
SQB	Guangtoushan	13MX02-7	106.63	33.32	224	1	Deng et al., (2016)[14]	68.23	1.21	4.76	2.10	114	876	10.16	49.22	0.58	0.13	86.22		
SQB	Guangtoushan	13MX06-2	106.62	33.25	224	1	Deng et al., (2016)[14]	69.20	0.73	5.38	2.48	106	1175	6.75	17.83	0.79	0.09	173.94	0.09	15.25
SQB	Guangtoushan	13QL13-2	106.68	33.45	218	1	Deng et al., (2016)[14]	71.99	0.54	3.62	3.23	104	794	6.82	30.92	0.59	0.13	116.36	0.13	35.36
SQB	Guangtoushan	13QL14-5	106.68	33.43	218	1	Deng et al., (2016)[14]	70.66	0.71	3.86	1.95	72	824	6.02	36.64	0.41	0.09	136.93		
SQB	Guangtoushan	13QL14-6	106.67	33.42	218	1	Deng et al., (2016)[14]	70.36	0.59	6.23	3.19	102	641	5.36	25.36	0.56	0.16	119.56	0.16	30.99
SQB	Guangtoushan	13QL14-8	106.65	33.37	218	1	Deng et al., (2016)[14]	68.98	0.63	7.35	3.26	108	693	5.09	21.82	0.59	0.16	136.15	0.16	25.21
SQB	Guangtoushan	13MX07-1	106.68	33.38	218	1	Deng et al., (2016)[14]	71.71	0.60	4.30	3.51	132	622	6.99	18.09	0.66	0.21	89.04	0.21	18.51
SQB	Guangtoushan	13MX07-5	106.68	33.38	218	1	Deng et al., (2016)[14]	71.57	0.79	4.54	2.72	130	523	14.56	18.37	1.50	0.25	35.89	0.25	8.33

Location	Intrusion	Sample	Longitude	Latitude	Age (Ma)	error	Reference	SiO2(wt.%)	MgO(wt.%)	Na2O(wt.%)	K2O(wt.%)	Rb(ppm)	Sr(ppm)	Y(ppm)	La(ppm)	Yb(ppm)	Rb/Sr(*)	Sr/Y	Rb/Sr(**)	La/Ybn
SQB	Guangtoushan	13MX07-6	106.68	33.38	218	1	Deng et al., (2016)[14]	70.98	0.67	4.09	4.34	186	516	18.36	23.60	1.96	0.36	28.12	0.36	8.19
SQB	Guangtoushan	13MX08-2	106.73	33.29	218	1	Deng et al., (2016)[14]	69.23	0.90	4.63	3.29	107	875	9.94	32.28	0.91	0.12	88.03	0.12	24.15
SQB	Zhangjiaba	13QL10-1	106.34	33.45	230	1	Deng et al., (2016)[14]	63.83	2.75	3.85	2.76	99	545	15.72	48.32	1.67	0.18	34.67	0.18	19.61
SQB	Zhangjiaba	13QL10-3	106.34	33.44	230	1	Deng et al., (2016)[14]	60.90	3.59	3.03	2.59	81	610	17.55	30.32	1.92	0.13	34.74	0.13	10.74
SQB	Zhangjiaba	13QL10-4	106.33	33.43	230	1	Deng et al., (2016)[14]	63.72	3.22	2.60	2.62	77	644	16.67	39.02	1.74	0.12	38.65	0.12	15.25
SQB	Xinyuan	13QL21-2	106.23	33.52	229	1	Deng et al., (2016)[14]	65.89	1.88	3.49	2.83	80	590	14.28	16.83	1.74	0.14	41.30	0.14	6.56
SQB	Xinyuan	13QL21-3	106.22	33.50	229	1	Deng et al., (2016)[14]	65.61	2.22	3.69	2.24	80	531	15.12	21.90	1.96	0.15	35.10	0.15	7.60
SQB	Xinyuan	13QL21-4	106.22	33.50	229	1	Deng et al., (2016)[14]	65.58	1.65	3.44	3.93	108	505	13.82	17.65	1.57	0.21	36.51	0.21	7.66
SQB	Xinyuan	13QL21-5	106.20	33.50	229	1	Deng et al., (2016)[14]	66.57	1.55	3.61	3.28	77	573	11.30	18.85	1.29	0.13	50.71	0.13	9.90
SQB	Xinyuan	13QL23-1	106.18	33.47	228	1	Deng et al., (2016)[14]	69.57	0.87	3.74	2.76	56	786	9.94	30.96	1.02	0.07	79.09		
SQB	Xinyuan	13QL23-2	106.18	33.47	228	1	Deng et al., (2016)[14]	67.60	0.96	4.24	2.51	50	696	11.40	20.90	1.31	0.07	61.04	0.07	10.81
SQB	Xinyuan	13QL23-3	106.18	33.45	228	1	Deng et al., (2016)[14]	65.75	1.39	3.69	3.19	59	802	12.23	40.68	1.16	0.07	65.58		
SQB	Miba	MB-02	105.83	33.50	211	2	Li et al., (2004)[36]	66.07	2.40	4.19	3.84	133	545	18.80	48.10	1.66	0.24	28.99	0.24	19.68
SQB	Miba	MB-05	105.83	33.50	211	2	Li et al., (2004)[36]	58.19	4.37	4.10	2.26	80	761	17.20	30.30	1.38	0.10	44.24	0.10	14.92
SQB	Miba	MB-15	105.83	33.50	211	2	Li et al., (2004)[36]	68.85	0.94	4.24	3.10	84	873	11.60	34.80	1.01			0.10	23.41
SQB	Miba	MB-03	105.83	33.50	211	2	Li et al., (2004)[36]	62.16	3.53	3.84	3.80	120	797	18.30	47.60	1.55	0.15	43.55	0.15	20.86
SQB	Miba	MB-06	105.83	33.50	211	2	Li et al., (2004)[36]	63.31	3.35	3.97	3.46	105	665	16.40	41.10	1.46	0.16	40.55	0.16	19.12
SQB	Miba	MB-14	105.83	33.50	211	2	Li et al., (2004)[36]	62.82	3.74	3.56	3.50	108	710	19.10	43.90	1.63	0.15	37.17	0.15	18.30
SQB	Miba	MB-09	105.83	33.50	211	2	Li et al., (2004)[36]	56.27	5.68	4.51	2.93	82	596	22.30	43.10	1.86	0.14	26.73	0.14	15.74
SQB	Miba	MB-11	105.83	33.50	211	2	Li et al., (2004)[36]	58.06	5.00	3.81	3.35	110	775	22.20	45.10	1.85	0.14	34.91	0.14	16.56
SQB	Miba	MB-18b	105.83	33.50	211	2	Li et al., (2004)[36]	56.78	5.28	3.74	2.49	130	599	16.60	32.80	1.38	0.22	36.08	0.22	16.15
SQB	Mishuling	MSL01	105.75	34.03	215	1	Li et al., (2013)[16]	60.96	2.20	3.65	2.62	87	351	23.87	46.63	2.38	0.25	14.70	0.25	13.31
SQB	Mishuling	MSL02	105.75	34.03	215	1	Li et al., (2013)[16]	63.10	2.07	3.34	3.64	137	375	23.83	50.72	2.12	0.36	15.72	0.36	16.25
SQB	Mishuling	MSL04	105.75	34.03	215	1	Li et al., (2013)[16]	61.94	2.31	3.39	4.05	166	434	24.94	51.16	2.11	0.38	17.38	0.38	16.47
SQB	Mishuling	MSL06	105.75	34.03	215	1	Li et al., (2013)[16]	62.70	2.42	3.38	3.74	162	444	30.41	54.68	2.74	0.36	14.58	0.36	13.56
SQB	Mishuling	MSL07	105.75	34.03	215	1	Li et al., (2013)[16]	62.99	2.10	3.31	4.38	149	444	29.03	54.71	1.99	0.34	15.30	0.34	18.68
SQB	Mishuling	MSL08	105.75	34.03	215	1	Li et al., (2013)[16]	63.21	2.22	3.69	3.41	157	431	29.54	55.28	2.32	0.36	14.59	0.36	16.19
SQB	Mishuling	MSL10	105.75	34.03	215	1	Li et al., (2013)[16]	64.68	2.03	3.41	4.05	136	378	24.02	46.12	1.87	0.36	15.72	0.36	16.75
SQB	Mishuling	MSL11	105.75	34.03	215	1	Li et al., (2013)[16]	65.12	1.78	3.56	4.00	155	326	25.50	54.02	2.11	0.48	12.77	0.48	17.39
SQB	Mishuling	MG-02	105.75	34.03	213	3	Qin et al., (2009)[37]	63.67	2.28	3.44	3.51	154	374	27.27	1.23	31.42	0.41	13.71	0.41	9.64
SQB	Mishuling	MG-08	105.75	34.03	213	3	Qin et al., (2009)[37]	63.66	2.24	3.64	2.65	139	380	19.61	1.15	13.13	0.37	19.39	0.37	15.94
SQB	Mishuling	MG-13	105.75	34.03	213	3	Qin et al., (2009)[37]	63.15	2.23	3.47	3.69	156	397	26.54	1.36	18.05	0.39	14.96	0.39	13.00
SQB	Mishuling	MG-2-16	105.75	34.03	213	3	Qin et al., (2009)[37]	63.94	2.04	3.46	3.95	172	354	27.32	1.25	15.93	0.49	12.95	0.49	11.07
SQB	Mishuling	WQ-21	105.75	34.03	213	3	Qin et al., (2009)[37]	64.88	1.85	3.15	3.99	186	357	23.90	1.18	21.60	0.52	14.95	0.52	15.05

Location	Intrusion	Sample	Longitude	Latitude	Age (Ma)	error	Reference	SiO2(wt.%)	MgO(wt.%)	Na2O(wt.%)	K2O(wt.%)	Rb(ppm)	Sr(ppm)	Y(ppm)	La(ppm)	Yb(ppm)	Rb/Sr(*)	Sr/Y	Rb/Sr(**)	La/Ybn
SQB	Mishuling	WQ-23	105.75	34.03	213	3	Qin et al., (2009)[37]	64.58	1.88	3.18	4.08	157	349	23.99	1.29	17.15	0.45	14.56	0.45	17.89
SQB	Mishuling	WQ-24	105.75	34.03	213	3	Qin et al., (2009)[37]	63.63	2.09	3.12	3.93	132	335	21.15	1.14	17.02	0.39	15.84	0.39	18.44
SQB	Mishuling	WQ-28	105.75	34.03	213	3	Qin et al., (2009)[37]	62.28	2.48	3.06	3.77	169	382	30.52	1.38	16.04	0.44	12.51	0.44	11.83
SQB	Mishuling	WQ21	105.75	34.03	213	3	Zhang et al., (2007)[38]	64.88	1.85	3.15	3.99	186	357	23.90	49.86	2.25	0.52	14.95	0.52	15.05
SQB	Mishuling	WQ23	105.75	34.03	213	3	Zhang et al., (2007)[38]	64.58	1.88	3.18	4.08	157	349	23.99	50.30	1.91	0.45	14.56	0.45	17.89
SQB	Mishuling	WQ24	105.75	34.03	213	3	Zhang et al., (2007)[38]	63.63	2.09	3.12	3.93	132	335	21.15	45.07	1.66	0.39	15.84	0.39	18.44
SQB	Mishuling	WQ28	105.75	34.03	213	3	Zhang et al., (2007)[38]	62.28	2.48	3.06	3.77	169	382	30.52	44.07	2.53	0.44	12.51	0.44	11.83
SQB	Huangzhuguan	H-03	105.65	33.95	214	1	Wang et al., (2011)[17]	63.77	3.16	3.79	2.76	82	589	16.20	25.60	1.54	0.14	36.36	0.14	11.29
SQB	Huangzhuguan	H-05	105.65	33.95	214	1	Wang et al., (2011)[17]	62.44	3.70	3.77	2.62	58	557	14.60	25.00	1.36	0.10	38.15	0.10	12.49
SQB	Huangzhuguan	H-06	105.65	33.95	214	1	Wang et al., (2011)[17]	68.38	1.59	3.97	3.70	70	450	11.20	19.50	1.09	0.16	40.18	0.16	12.15
SQB	Wenquan	1358/1	105.25	34.60	225	3	Cai et al., (2011)[18]	65.21	1.74	3.60	4.49	156	670	17.60	61.70	1.84	0.23	38.07	0.23	22.78
SQB	Wenquan	1027/1	105.25	34.60	225	3	Cai et al., (2011)[18]	69.44	0.83	4.19	5.15	216	290	12.70	49.10	1.16	0.74	22.83	0.74	28.75
SQB	Wenquan	1025/1	105.25	34.60	225	3	Cai et al., (2011)[18]	70.78	0.84	4.04	4.82	206	260	12.10	39.80	1.16	0.79	21.49	0.79	23.31
SQB	Wenquan	1043/1	105.25	34.60	225	3	Cai et al., (2011)[18]	71.32	0.73	4.29	4.52	206	220	12.60	26.90	1.17	0.94	17.46	0.94	15.62
SQB	Wenquan	1372/1	105.25	34.60	225	3	Cai et al., (2011)[18]	66.58	1.61	3.60	4.67	174	600	17.30	60.40	1.77	0.29	34.68	0.29	23.18
SQB	Wenquan	1044/1	105.25	34.60	225	3	Cai et al., (2011)[18]	71.94	0.67	4.21	4.66	239	210	10.70	29.80	1.13	1.14	19.63	1.14	17.91
SQB	Wenquan	1026/2b	105.25	34.60	225	3	Cai et al., (2011)[18]	70.52	0.79	4.34	5.04	209	270	12.70	36.90	1.35	0.77	21.26	0.77	18.57
SQB	Wenquan	WQ66	105.25	34.60	223	7	Zhang et al., (2007)[38]	66.74	1.72	3.12	4.89	229	188	24.00	26.69	2.40	1.22	7.83		
SQB	Wenquan	WQ66-1	105.25	34.60	223	7	Zhang et al., (2007)[38]	67.17	1.38	3.28	4.73	207	429	17.10	43.67	1.49	0.48	25.09	0.48	19.91
SQB	Wenquan	WQ66-2	105.25	34.60	223	7	Zhang et al., (2007)[38]	67.18	1.53	3.41	4.40	188	488	20.50	42.35	1.87	0.38	23.80	0.38	15.38
SQB	Wenquan	WQ66-3	105.25	34.60	223	7	Zhang et al., (2007)[38]	67.34	1.52	3.16	4.66	152	447	15.90	37.73	1.42	0.34	28.12	0.34	18.05
SQB	Wenquan	W8-1	105.25	34.60	216	2	Zhu et al., (2011)[19]	69.67	1.08	3.58	5.18	178	299	14.30	23.60	1.27	0.60	20.91	0.60	12.62
SQB	Wenquan	W8-2	105.25	34.60	216	2	Zhu et al., (2011)[19]	70.04	1.35	3.71	4.00	147	256	16.80	30.90	1.52	0.57	15.24	0.57	13.81
SQB	Wenquan	W8-3	105.25	34.60	216	2	Zhu et al., (2011)[19]	69.91	1.43	3.90	3.52	139	288	18.20	33.40	1.65	0.48	15.82	0.48	13.75
SQB	Wenquan	W17-2	105.25	34.60	217	2	Zhu et al., (2011)[19]	71.42	0.65	3.97	4.99	162	273	20.00	19.30	1.91	0.59	13.65	0.59	6.86
SQB	Wenquan	W17-3	105.25	34.60	217	2	Zhu et al., (2011)[19]	71.46	0.57	3.83	5.00	167	250	18.40	25.30	1.76	0.67	13.59	0.67	9.77
SQB	Wenquan	W23-4	105.25	34.60	217	2	Zhu et al., (2011)[19]	71.74	1.27	3.01	4.92	189	148	17.10	39.80	1.60	1.28	8.65	1.28	16.90
SQB	Wenquan	W23-5	105.25	34.60	217	2	Zhu et al., (2011)[19]	70.48	1.57	2.98	4.90	267	148	15.30	35.70	1.46	1.80	9.67	1.80	16.61
SQB	Wenquan	W25-1	105.25	34.60	217	2	Zhu et al., (2011)[19]	71.90	0.77	3.63	4.36	174	203	12.40	35.70	1.18	0.86	16.37	0.86	20.55
SQB	Wenquan	W25-3	105.25	34.60	217	2	Zhu et al., (2011)[19]	70.99	0.71	3.71	5.00	188	204	13.50	32.60	1.05	0.92	15.11	0.92	21.09
SQB	Wenquan	W26-3	105.25	34.60	217	2	Zhu et al., (2011)[19]	71.57	1.00	3.19	4.67	166	255	12.80	35.30	1.28	0.65	19.92	0.65	18.73
SQB	Wenquan	YX-9	105.25	34.60	217	2	Zhu et al., (2011)[19]	71.10	1.00	3.17	4.69	161	212	16.50	33.60	1.57	0.76	12.85	0.76	14.54
SQB	Luchuba	SEB12–01	105.00	34.08	211	1	Kong et al., (2017)[20]	65.20	2.64	3.32	3.51	167	407	17.30	35.30	1.58	0.41	23.53	0.41	15.18
SQB	Luchuba	SEB12–02	105.00	34.08	211	1	Kong et al., (2017)[20]	64.15	2.77	3.44	3.52	144	383	17.70	35.50	1.61	0.38	21.64	0.38	14.98

Location	Intrusion	Sample	Longitude	Latitude	Age (Ma)	error	Reference	SiO2(wt.%)	MgO(wt.%)	Na2O(wt.%)	K2O(wt.%)	Rb(ppm)	Sr(ppm)	Y(ppm)	La(ppm)	Yb(ppm)	Rb/Sr(*)	Sr/Y	Rb/Sr(**)	La/Ybn
SQB	Luchuba	BSB12–01	105.00	34.08	211	1	Kong et al., (2017)[20]	66.63	1.66	3.56	4.61	173	372	14.90	24.40	1.30	0.47	24.97	0.47	12.75
SQB	Luchuba	YDB12–03	105.00	34.08	211	1	Kong et al., (2017)[20]	66.75	2.58	3.19	3.89	165	381	15.70	35.70	1.48	0.43	24.27	0.43	16.39
SQB	Luchuba	YDB12–05	105.00	34.08	211	1	Kong et al., (2017)[20]	67.18	1.70	3.35	4.39	208	416	17.10	39.00	1.48	0.50	24.33	0.50	17.90
SQB	Luchuba	NSC12–01	105.00	34.08	211	1	Kong et al., (2017)[20]	71.29	0.77	3.62	4.35	184	224	10.90	29.10	0.89	0.82	20.55	0.82	22.21
SQB	Zhongchuan	TJZ12–01	105.05	34.28	219	1	Kong et al., (2017)[20]	69.94	0.88	3.59	4.39	213	279	13.50	25.30	1.17	0.76	20.67	0.76	14.69
SQB	Zhongchuan	LTB12–01	105.05	34.28	219	1	Kong et al., (2017)[20]	70.51	0.99	3.44	4.28	215	287	12.80	28.90	1.12	0.75	22.42	0.75	17.53
SQB	Zhongchuan	DBQ12–01	105.05	34.28	219	1	Kong et al., (2017)[20]	69.92	0.82	3.15	4.96	276	374	20.10	35.50	1.86	0.74	18.61	0.74	12.97
SQB	Zhongchuan	DBQ12–03	105.05	34.28	219	1	Kong et al., (2017)[20]	70.03	0.92	3.04	4.12	257	289	20.80	44.90	1.79	0.89	13.89	0.89	17.04
SQB	Zhongchuan	ZTC12–01	105.05	34.28	219	1	Kong et al., (2017)[20]	68.95	0.86	3.26	4.84	234	377	16.10	30.90	1.36	0.62	23.42	0.62	15.43
SQB	Zhongchuan	MDG12–01	105.05	34.28	219	1	Kong et al., (2017)[20]	71.28	0.73	3.42	4.18	257	313	16.90	31.80	1.43	0.82	18.52	0.82	15.11
SQB	Zhongchuan	DPC12–01	105.05	34.28	219	1	Kong et al., (2017)[20]	70.64	0.54	3.69	4.43	283	215	15.00	35.60	1.25	1.32	14.33	1.32	19.35
SQB	Zhongchuan	DBL12–01	105.05	34.28	219	1	Kong et al., (2017)[20]	70.56	0.87	3.46	3.84	215	303	16.10	26.20	1.37	0.71	18.82	0.71	12.99
SQB	Zhongchuan	MZG12–02	105.05	34.28	219	1	Kong et al., (2017)[20]	64.61	1.05	3.19	7.27	341	364	22.40	63.80	1.87	0.94	16.25	0.94	23.18
SQB	Zhongchuan	MK12–02	105.05	34.28	219	1	Kong et al., (2017)[20]	68.35	1.08	3.43	3.99	261	329	26.80	36.90	2.45	0.79	12.28	0.79	10.23
SQB	Zhongchuan	ZC-01	105.00	34.33	220	1	Yang et al., (2017)[39]	63.59	1.14	3.81	6.10	232	493	20.81	46.86	1.68	0.47	23.69	0.47	18.95
SQB	Zhongchuan	ZC-05	105.00	34.33	220	1	Yang et al., (2017)[39]	67.41	1.10	3.30	4.97	213	345	23.50	60.80	2.41	0.62	14.68	0.62	17.14
SQB	Zhongchuan	QS-1	105.00	34.33	236	2	Zhu et al., (2013)[21]	69.71	1.02	3.57	4.11	180	323	23.00	56.30	1.96	0.56	14.04	0.56	19.51
SQB	Zhongchuan	QS-2	105.00	34.33	236	2	Zhu et al., (2013)[21]	70.47	1.13	3.54	3.21	166	304	27.50	45.50	2.37	0.55	11.05	0.55	13.04
SQB	Zhongchuan	QS-4	105.00	34.33	236	2	Zhu et al., (2013)[21]	69.94	0.93	3.28	4.98	197	338	18.60	49.90	1.53	0.58	18.17	0.58	22.16
SQB	Zhongchuan	QS-7	105.00	34.33	236	2	Zhu et al., (2013)[21]	69.17	1.02	3.54	4.45	189	347	24.00	42.20	2.09	0.54	14.46	0.54	13.72
SQB	Zhongchuan	QS-9	105.00	34.33	236	2	Zhu et al., (2013)[21]	68.61	0.85	3.17	5.89	219	344	15.50	42.80	1.29	0.64	22.19	0.64	22.54
SQB	Zhongchuan	QS-10	105.00	34.33	236	2	Zhu et al., (2013)[21]	70.06	1.10	3.52	3.86	179	321	18.10	45.40	1.57	0.56	17.73	0.56	19.64
SQB	Zhongchuan	QSL-2	105.00	34.33	233	2	Zhu et al., (2013)[21]	70.25	0.96	3.27	4.77	191	329	18.00	40.90	1.53	0.58	18.28	0.58	18.16
SQB	Zhongchuan	QSL-3	105.00	34.33	233	2	Zhu et al., (2013)[21]	68.50	0.83	3.61	5.25	201	328	18.10	49.00	1.49	0.61	18.12	0.61	22.34
SQB	Zhongchuan	QSL-4	105.00	34.33	233	2	Zhu et al., (2013)[21]	67.96	0.87	3.67	5.15	202	356	17.00	31.70	1.41	0.57	20.94	0.57	15.27
SQB	Zhongchuan	QSL-5	105.00	34.33	233	2	Zhu et al., (2013)[21]	69.89	1.01	3.48	4.47	211	294	21.20	35.00	1.71	0.72	13.87	0.72	13.90
SQB	Zhongchuan	QSL-6	105.00	34.33	233	2	Zhu et al., (2013)[21]	69.05	0.84	3.66	4.98	204	329	19.50	41.60	1.56	0.62	16.87	0.62	18.12
SQB	Zhongchuan	QSL-7	105.00	34.33	233	2	Zhu et al., (2013)[21]	71.00	0.92	3.31	4.41	220	257	28.00	39.50	2.44	0.86	9.18	0.86	11.00
SQB	Zhongchuan	QSL-8	105.00	34.33	233	2	Zhu et al., (2013)[21]	67.61	1.15	3.45	4.83	192	382	20.60	43.00	1.77	0.50	18.54	0.50	16.50
SQB	Lüjing	LJ01/1B	104.57	34.57	221	1	Wang et al., (2016)[22]	71.53	0.64	3.44	5.07	189	205	10.10	15.10	1.09	0.92	20.30		
SQB	Lüjing	LJ02/1B	104.57	34.54	221	1	Wang et al., (2016)[22]	70.98	0.65	3.34	5.39	195	216	11.00	25.90	1.11	0.90	19.60	0.90	15.85
SQB	Lüjing	LJ02/2B	104.57	34.50	221	1	Wang et al., (2016)[22]	71.70	0.83	3.23	4.42	171	226	14.00	50.10	1.44	0.76	16.14	0.76	23.63
SQB	Lüjing	LJ10/1B	104.59	34.28	221	1	Wang et al., (2016)[22]	69.71	0.85	3.48	5.08	217	270	14.90	49.60	1.42	0.81	18.09	0.81	23.73
SQB	Lüjing	LJ13/1B	104.60	34.28	221	1	Wang et al., (2016)[22]	70.66	0.86	3.30	4.86	185	256	21.70	52.50	1.86	0.72	11.79	0.72	19.17
SQB	Lüjing	LJ13/5B	104.60	34.28	221	1	Wang et al., (2016)[22]	66.92	1.32	3.43	4.97	189	341	22.30	64.00	2.19	0.55	15.31	0.55	19.85

Location	Intrusion	Sample	Longitude	Latitude	Age (Ma)	error	Reference	SiO2(wt.%)	MgO(wt.%)	Na2O(wt.%)	K2O(wt.%)	Rb(ppm)	Sr(ppm)	Y(ppm)	La(ppm)	Yb(ppm)	Rb/Sr(*)	Sr/Y	Rb/Sr(**)	La/Ybn
SQB	Meiwu	WQ113	103.25	34.88	242	2	Zhang et al., (2007)[38]	65.86	3.24	2.30	4.25	242	281	27.21	49.86	2.43	0.86	10.34	0.86	13.94
SQB	Meiwu	WQ114	103.25	34.88	242	2	Zhang et al., (2007)[38]	68.20	1.77	2.79	3.93	195	361	20.70	39.95	1.88	0.54	17.45	0.54	14.44
SQB	Meiwu	09104	103.5	35.02	241	3	Luo et al., (2015)[23]	60.45	5.42	2.87	2.27	95	418	15.90	25.00	1.36	0.23	26.29	0.23	12.49
SQB	Meiwu	09107	103.62	34.95	241	3	Luo et al., (2015)[23]	59.51	4.58	3.32	0.76	34	494	18.30	19.40	1.51	0.07	26.99	0.07	8.73
SQB	Meiwu	09108	103.62	34.95	240	3	Luo et al., (2015)[23]	62.55	4.74	3.06	2.52	94	364	19.10	25.20	1.75	0.26	19.06	0.26	9.78
SQB	Meiwu	D36	103.62	34.95	245	6	Jin et al., (2005)[24]	60.08	4.95	3.00	2.72	122	420	20.40	29.30	1.73	0.29	20.59	0.29	11.51
SQB	Meiwu	0907	103.12	35.02	242	2	Luo et al., (2015)[23]	69.56	1.47	3.57	3.69	161	548	13.40	32.60	1.17	0.29	40.90	0.29	18.93
SQB	Meiwu	0910	103.12	35.00	242	2	Luo et al., (2015)[23]	68.31	2.01	3.36	3.58	157	501	16.70	24.30	1.52	0.31	30.00	0.31	10.86
SQB	Meiwu	SLG-2	103.12	35.04	243	3	Luo et al., (2015)[23]	69.93	1.48	3.67	3.64	166	512	12.40	28.70	1.09	0.32	41.29	0.32	17.89
SQB	Meiwu	MR-1	103.17	35.05	242	2	Luo et al., (2015)[23]	66.72	2.22	3.48	3.56	185	553	17.70	41.00	1.63	0.33	31.24	0.33	17.09
SQB	Meiwu	09109	103.61	34.95	242	2	Luo et al., (2015)[23]	65.18	2.75	3.36	3.44	140	442	17.40	38.40	1.41	0.32	25.40	0.32	18.50
SQB	Meiwu	09111	103.19	35.04	242	2	Luo et al., (2015)[23]	65.55	2.45	3.38	3.46	153	463	15.00	42.90	1.30	0.33	30.87	0.33	22.42
SQB	Meiwu	09112	103.16	35.05	242	2	Luo et al., (2015)[23]	67.84	2.02	3.59	3.51	130	536	12.90	23.30	1.05	0.24	41.55	0.24	15.07
SQB	Xiahe	WQ118	102.85	35.17	244	1	Zhang et al., (2007)[38]	59.86	4.03	2.71	1.90	67	397	15.56	26.77	1.78	0.17	25.53	0.17	10.22
SQB	Xiahe	WQ119	102.85	35.17	244	1	Zhang et al., (2007)[38]	64.81	2.82	2.71	2.82	109	336	16.61	34.92	1.69	0.33	20.23	0.33	14.04
SQB	Xiahe	WQ120	102.85	35.17	244	1	Zhang et al., (2007)[38]	64.59	2.58	2.77	2.89	112	323	15.77	33.35	1.52	0.35	20.47	0.35	14.90
SQB	Xiahe	XH11-02MG	102.67	35.22	248	1	Wei et al., (2013)[25]	69.18	1.34	3.20	4.49	171	563	14.98	28.16	1.61	0.30	37.61	0.30	11.88
SQB	Xiahe	XH11-03MG	102.67	35.22	248	1	Wei et al., (2013)[25]	68.09	1.52	3.26	4.29	163	564	13.43	29.54	1.27	0.29	42.00	0.29	15.80
SQB	Xiahe	XH11-07MG	102.67	35.22	248	1	Wei et al., (2013)[25]	66.07	1.56	3.09	4.46	186	598	18.46	32.78	1.77	0.31	32.41	0.31	12.58
SQB	Xiahe	XH11-11GD	102.67	35.22	244	1	Wei et al., (2013)[25]	62.10	2.38	2.55	2.76	124	540	21.66	35.54	1.91	0.23	24.94	0.23	12.64
SQB	Xiahe	XH11-18GD	102.67	35.22	244	1	Wei et al., (2013)[25]	60.18	3.87	2.86	1.87	74	434	17.70	27.61	1.68	0.17	24.54	0.17	11.16
SQB	Xiahe	XH11-21MG	102.67	35.22	248	1	Wei et al., (2013)[25]	67.01	1.44	3.17	4.01	153	645	19.30	25.35	1.91	0.24	33.41	0.24	9.02
SQB	Xiahe	XH11-23MG	102.67	35.22	248	1	Wei et al., (2013)[25]	66.89	1.58	2.94	4.28	172	603	18.00	34.46	1.76	0.29	33.48	0.29	13.30
SQB	Xiekeng	0936	102.45	35.56	244	2	Luo et al., (2012)[26]	55.28	2.50	3.68	1.20	49	434	22.60	16.60	2.20	0.11	19.20	0.11	5.13
SQB	Xiekeng	P4-05a	102.45	35.56	242	2	Luo et al., (2012)[26]	56.82	3.62	3.06	1.92	44	305	23.10	20.20	2.81	0.14	13.20	0.14	4.88
SQB	Xiekeng	0948	102.45	35.56	244	2	Luo et al., (2012)[26]	56.00	5.62	2.88	1.34	57	305	26.40	17.30	2.55	0.19	11.55	0.19	4.61
SQB	Xiekeng	0951	102.45	35.56	244	2	Luo et al., (2012)[26]	62.29	2.00	3.40	2.22	94	342	23.00	27.80	2.37	0.27	14.87	0.27	7.97
SQB	Xiekeng	XK-8	102.45	35.56	242	2	Luo et al., (2012)[26]	64.72	1.80	4.82	3.16	81	361	17.30	24.20	1.80	0.22	20.87	0.22	9.13
SQB	Shuangpenxi	0926	102.33	35.56	242	3	Luo et al., (2012)[26]	63.83	2.22	3.03	3.23	160	283	28.50	37.30	2.66	0.57	9.93	0.57	9.53
SQB	Shuangpenxi	0927	102.33	35.56	242	3	Luo et al., (2012)[26]	63.85	2.24	2.91	3.56	172	283	29.20	42.20	2.67	0.61	9.69	0.61	10.74
SQB	Shuangpenxi	SPX-6	102.33	35.56	242	3	Luo et al., (2012)[26]	63.05	2.52	2.92	3.28	149	298	30.60	52.10	2.73	0.50	9.74	0.50	12.96
SQB	Tongren	TR1-2	101.88	35.45	241	1	Li et al., (2015)[27]	66.73	1.53	3.27	4.73	233	244	14.60	35.00	1.46	0.95	16.71	0.95	16.29
SQB	Tongren	TR1-3	101.88	35.45	241	1	Li et al., (2015)[27]	66.57	2.03	2.96	3.98	223	278	17.50	27.50	1.88	0.80	15.89	0.80	9.94
SQB	Tongren	TR1-4	101.88	35.45	241	1	Li et al., (2015)[27]	65.78	1.63	2.88	4.18	230	196	16.40	36.30	1.71	1.17	11.95	1.17	14.42

Location	Intrusion	Sample	Longitude	Latitude	Age (Ma)	error	Reference	SiO2(wt.%)	MgO(wt.%)	Na2O(wt.%)	K2O(wt.%)	Rb(ppm)	Sr(ppm)	Y(ppm)	La(ppm)	Yb(ppm)	Rb/Sr(*)	Sr/Y	Rb/Sr(**)	La/Ybn
SQB	Tongren	TR1-5	101.88	35.45	241	1	Li et al., (2015)[27]	66.62	1.89	2.79	4.05	186	254	19.70	27.30	2.20	0.73	12.89	0.73	8.43
SQB	Tongren	TR1-6	101.88	35.45	241	1	Li et al., (2015)[27]	65.09	2.03	2.63	4.91	245	305	20.40	30.20	2.09	0.80	14.95	0.80	9.82
SQB	Tongren	TR1-7	101.88	35.45	241	1	Li et al., (2015)[27]	66.14	1.98	3.03	4.64	222	304	19.10	28.80	2.15	0.73	15.92	0.73	9.10
SQB	Tongren	TR1-8	101.88	35.45	241	1	Li et al., (2015)[27]	66.09	1.80	3.07	4.24	232	303	16.70	32.20	1.77	0.77	18.14	0.77	12.36
SQB	Tongren	TR2-1	101.88	35.45	241	2	Li et al., (2015)[27]	63.75	2.40	3.09	3.62	170	275	23.00	24.40	2.50	0.62	11.96	0.62	6.63
SQB	Tongren	TR2-2	101.88	35.45	241	2	Li et al., (2015)[27]	64.58	2.24	2.91	3.69	166	255	18.00	40.80	1.96	0.65	14.17	0.65	14.14
SQB	Tongren	TR2-3	101.88	35.45	241	2	Li et al., (2015)[27]	67.04	1.29	3.53	3.71	188	252	23.80	33.20	2.71	0.75	10.59	0.75	8.32
SQB	Maixiu	ZK10-2	101.83	35.27	234	3	Li et al., (2013)[28]	56.96	2.89	2.60	1.90	33	252	22.70	23.90	2.09	0.13	11.10	0.13	7.77
SQB	Maixiu	ZK10-3	101.83	35.27	234	3	Li et al., (2013)[28]	56.22	4.64	2.53	1.72	124	144	16.70	21.70	1.66	0.86	8.62	0.86	8.88
SQB	Maixiu	ZK10-36	101.83	35.27	234	3	Li et al., (2013)[28]	57.29	3.01	2.44	1.19	25	240	19.00	15.00	1.97	0.11	12.63	0.11	5.17
SQB	Maixiu	ZK11-06	101.83	35.27	234	3	Li et al., (2013)[28]	55.08	4.53	2.29	1.83	68	201	15.00	15.50	1.53	0.34	13.40	0.34	6.88
SQB	Maixiu	ZK11-09	101.83	35.27	234	3	Li et al., (2013)[28]	55.64	3.59	2.34	1.69	63	251	16.20	15.50	1.61	0.25	15.49	0.25	6.54
SQB	Maixiu	ZK11-20	101.83	35.27	234	3	Li et al., (2013)[28]	55.58	2.78	2.45	1.24	45	341	14.70	15.80	1.50			0.13	7.16
SQB	Xiaguanfang	XGF-12	109.85	33.63	143	1	Wu et al., (2013)[29]	67.89	1.28	3.93	4.90	94	796	12.20	31.70	1.22	0.12	65.25	0.12	17.65
SQB	Xiaguanfang	XGF-13	109.85	33.63	143	1	Wu et al., (2013)[29]	68.31	1.38	3.65	5.47	96	850	9.37	27.10	1.00	0.11	90.72	0.11	18.41
SQB	Xiaguanfang	XGF-14	109.85	33.63	143	1	Wu et al., (2013)[29]	66.80	1.42	4.22	4.82	84	939	11.10	32.30	1.24	0.09	84.59	0.09	17.70
SQB	Xiaguanfang	XGF-7	109.85	33.63	143	1	Wu et al., (2013)[29]	60.92	2.95	4.43	3.07	70	1051	21.00	40.80	2.43	0.07	50.05	0.07	11.41
SQB	Xiaguanfang	XGF-8	109.85	33.63	143	1	Wu et al., (2013)[29]	62.21	2.67	4.32	3.10	62	1026	19.20	41.00	2.33	0.06	53.44	0.06	11.95
SQB	Yuanzijie	YZJ-18	109.80	33.62	142	1	Wu et al., (2013)[29]	67.03	2.31	3.94	3.68	83	792	9.15	38.10	0.95	0.11	86.56	0.11	27.24
SQB	Yuanzijie	YZJ-19	109.80	33.62	142	1	Wu et al., (2013)[29]	66.74	2.05	4.22	3.54	68	882	12.10	34.00	1.35	0.08	72.89	0.08	17.11
SQB	Yuanzijie	YZJ-20	109.80	33.62	142	1	Wu et al., (2013)[29]	67.27	1.92	4.14	3.35	68	795	11.50	29.10	1.27	0.09	69.13	0.09	15.57
SQB	Yuanzijie	YZJ-22	109.80	33.62	142	1	Wu et al., (2013)[29]	61.73	1.89	0.19	3.88	92	293	13.30	38.10	1.52	0.31	22.03	0.31	17.03
SQB	Yuanzijie	YZJ-25	109.80	33.62	142	1	Wu et al., (2013)[29]	63.25	1.62	0.22	3.98	92	269	11.30	33.60	1.44	0.34	23.81	0.34	15.85
SQB	Xiaohekou	XHK-1	109.63	33.59	150	1	Wu et al., (2013)[29]	66.24	1.68	4.16	3.33	75	1044	16.20	31.40	1.75	0.07	64.44	0.07	12.19
SQB	Xiaohekou	XHK-2	109.63	33.59	150	1	Wu et al., (2013)[29]	65.87	1.72	4.20	3.11	64	978	15.70	32.30	1.75	0.07	62.29	0.07	12.54
SQB	Xiaohekou	XHK-4	109.63	33.59	150	1	Wu et al., (2013)[29]	68.21	0.98	4.00	3.73	70	719	12.60	34.80	1.39	0.10	57.06	0.10	17.01
SQB	Xiaohekou	XHK-5	109.63	33.59	150	1	Wu et al., (2013)[29]	67.47	1.05	3.85	3.80	59	821	13.20	28.30	1.66	0.07	62.20	0.07	11.58
SQB	Xiaohekou	XH2	109.63	33.59	148	3	unpublished data	69.64	0.81	2.32	4.49	117	498	13.50	29.00	1.39	0.23	36.89	0.23	14.17
SQB	Xiaohekou	XH3	109.63	33.59	148	3	unpublished data	67.05	2.03	3.85	4.86	130	1004	22.10	28.00	2.09	0.13	45.43	0.13	9.10
SQB	Xiaohekou	XH5	109.63	33.59	148	3	unpublished data	65.54	2.57	5.46	0.69	168	2310	26.40	32.00	2.56	0.07	87.50	0.07	8.49
SQB	Wagou	WG-2	109.63	33.59	145	1	Wu et al., (2013)[29]	61.37	2.56	3.78	3.02	86	4833	25.50	41.80	3.00	0.02	189.53	0.02	9.47
SQB	Wagou	WG-3	109.63	33.59	145	1	Wu et al., (2013)[29]	63.23	2.09	4.23	2.22	84	1120	14.40	40.00	1.80	0.08	77.78	0.08	15.10
SQB	Wagou	WG-6	109.63	33.59	145	1	Wu et al., (2013)[29]	64.93	2.10	3.34	3.05	109	712	19.60	30.70	2.03	0.15	36.33	0.15	10.27
SQB	Wagou	WG-8	109.63	33.59	145	1	Wu et al., (2013)[29]	65.51	1.40	3.37	4.21	133	698	14.10	25.80	1.68	0.19	49.50	0.19	10.43
SQB	Yuanjiagou	YJG-1	109.63	33.59	145	1	Wu et al., (2013)[29]	69.86	1.02	4.16	4.13	56	665	9.39	19.20	0.94	0.08	70.82	0.08	13.88

Location	Intrusion	Sample	Longitude	Latitude	Age (Ma)	error	Reference	SiO2(wt.%)	MgO(wt.%)	Na2O(wt.%)	K2O(wt.%)	Rb(ppm)	Sr(ppm)	Y(ppm)	La(ppm)	Yb(ppm)	Rb/Sr(*)	Sr/Y	Rb/Sr(**)	La/Ybn
SQB	Yuanjiagou	YJG-3	109.63	33.59	145	1	Wu et al., (2013)[29]	69.86	1.21	4.24	4.11	64	766	10.50	26.50	1.24	0.08	72.95	0.08	14.52
SQB	Baishagou	BSG-9	109.61	33.50	143	1	Wu et al., (2013)[29]	64.37	1.60	5.04	3.59	89	1540	24.30	61.80	2.50	0.06	63.37	0.06	16.79
SQB	Baishagou	BSG-12	109.61	33.50	143	1	Wu et al., (2013)[29]	62.70	1.60	5.19	3.60	93	1620	23.50	61.90	2.58	0.06	68.94	0.06	16.30
SQB	Baishagou	BSG-2	109.61	33.50	143	1	Wu et al., (2013)[29]	56.61	3.74	4.94	2.52	62	1222	21.70	45.40	2.06	0.05	56.31	0.05	14.97
SQB	Baishagou	BSG-4	109.61	33.50	143	1	Wu et al., (2013)[29]	60.20	2.99	5.01	3.57	89	1335	21.40	49.60	2.02	0.07	62.38	0.07	16.68
SQB	Baishagou	BSG-7	109.61	33.50	143	1	Wu et al., (2013)[29]	59.98	2.53	4.47	3.17	88	1122	21.80	46.40	2.19	0.08	51.47	0.08	14.39
SQB	Chigou	CGII-1	109.61	33.50	146	1	Wu et al., (2013)[29]	65.98	1.32	4.57	3.96	94	1157	17.40	44.40	1.82	0.08	66.49	0.08	16.57
SQB	Chigou	CGII-2	109.61	33.50	146	1	Wu et al., (2013)[29]	64.21	1.93	4.18	4.00	91	1315	18.80	53.20	1.87	0.07	69.95	0.07	19.33
SQB	Chigou	CGIII-2	109.61	33.50	146	1	Wu et al., (2013)[29]	63.14	2.31	3.98	3.33	87	1288	21.90	46.10	2.39	0.07	58.81	0.07	13.10
SQB	Chigou	CGIII-3	109.61	33.50	146	1	Wu et al., (2013)[29]	62.60	2.44	4.37	3.51	83	1438	22.80	47.40	2.01	0.06	63.07	0.06	16.02
SQB	Chigou	CG-1	109.61	33.50	146	1	Wu et al., (2013)[29]	60.49	3.36	3.70	3.12	116	1207	21.30	52.50	2.09	0.10	56.67	0.10	17.06
SQB	Chigou	CG1301-122	109.61	33.50	146	1	Wu et al., (2013)[29]	60.21	3.02	3.84	3.25	112	1430	21.30	55.70	2.05	0.08	67.14	0.08	18.46
SQB	Chigou	CG1301-505	109.61	33.50	146	1	Wu et al., (2013)[29]	60.83	2.35	3.53	3.90	128	1352	18.70	46.90	1.79	0.09	72.30	0.09	17.80
SQB	Chigou	CG-4	109.61	33.50	142	1	Wu et al., (2013)[29]	61.71	3.22	3.48	3.34	97	1273	19.90	41.50	1.96	0.08	63.97	0.08	14.38
SQB	Chigou	CGV-2	109.61	33.50	144	1	Wu et al., (2013)[29]	55.73	5.52	3.56	4.21	128	938	22.20	47.10	2.21			0.14	14.48
SQB	Chigou	CGV-5	109.61	33.50	144	1	Wu et al., (2013)[29]	57.64	3.21	4.82	3.55	100	1149	21.90	46.50	2.28	0.09	52.47	0.09	13.85
SQB	Chigou	CGV03	109.61	33.50	144	1	Wu et al., (2013)[29]	62.10	2.24	4.81	3.73	103	1361	21.70	54.30	2.24	0.08	62.72	0.08	16.47
SQB	Chigou	CGVI-4	109.61	33.50	146	1	Wu et al., (2013)[29]	63.21	1.92	4.69	3.86	89	1415	20.80	53.20	2.11	0.06	68.03	0.06	17.13
SQB	Chigou	CG201-165	109.61	33.50	146	1	Wu et al., (2013)[29]	67.80	1.41	3.90	4.64	82	1116	18.40	52.60	1.70	0.07	60.65	0.07	21.02
SQB	Chigou	CG201-166	109.61	33.50	146	1	Wu et al., (2013)[29]	67.20	1.40	4.11	4.48	73	1035	13.60	52.20	1.39	0.07	76.10	0.07	25.51
SQB	Chigou	CG201-334	109.61	33.50	146	1	Wu et al., (2013)[29]	63.68	2.14	3.61	4.33	114	1213	19.70	57.00	2.06	0.09	61.57	0.09	18.80
SQB	Tudigou	TDG-8	109.61	33.50	145	1	Wu et al., (2013)[29]	64.11	1.34	2.15	6.25	125	274	15.20	34.40	1.69			0.46	13.83
SQB	Tudigou	TDG-9	109.61	33.50	145	1	Wu et al., (2013)[29]	64.18	1.34	1.94	6.40	129	256	16.20	34.00	1.93			0.50	11.97
SQB	Shuangyuangou	SYG-3	109.58	33.50	145	1	Wu et al., (2013)[29]	66.37	1.35	4.43	3.56	73	913	16.50	37.40	1.90	0.08	55.33	0.08	13.37
SQB	Shuangyuangou	SYG-2	109.58	33.50	145	1	Wu et al., (2013)[29]	65.02	1.46	4.34	3.00	63	1013	15.60	31.70	1.81	0.06	64.94	0.06	11.90
SQB	Shuangyuangou	SYG-4	109.58	33.50	145	1	Wu et al., (2013)[29]	67.18	1.17	4.35	3.71	56	991	14.80	22.90	1.60	0.06	66.96	0.06	9.72
SQB	Shuangyuangou	SYG-7	109.58	33.50	145	1	Wu et al., (2013)[29]	66.81	1.52	4.46	3.27	63	1028	18.10	32.30	2.01	0.06	56.80	0.06	10.92
SQB	Shuangyuangou	SYG-10	109.58	33.50	145	1	Wu et al., (2013)[29]	66.48	1.36	4.57	3.03	62	1204	13.90	34.00	1.48			0.05	15.61
SQB	Shuangyuangou	SYG-9	109.58	33.50	145	1	Wu et al., (2013)[29]	66.90	1.26	4.13	3.59	79	947	13.90	36.80	1.39	0.08	68.13	0.08	17.99
SQB	Lengshuigou	LSG-3	109.62	33.43	142	1	Wu et al., (2013)[29]	66.60	1.40	4.58	3.34	59	1128	13.00	19.50	1.45	0.05	86.77	0.05	9.14
SQB	Lengshuigou	LSG-9	109.62	33.43	142	1	Wu et al., (2013)[29]	63.99	1.97	1.71	3.42	63	1134	16.60	37.00	1.81	0.06	68.31	0.06	13.89
SQB	Lengshuigou	LSG-32	109.62	33.43	142	1	Wu et al., (2013)[29]	66.14	1.00	1.29	8.44	213	299	9.44	19.30	1.01	0.71	31.67	0.71	12.98
SQB	Lengshuigou	LSG-33	109.62	33.43	142	1	Wu et al., (2013)[29]	64.28	0.69	3.90	6.12	159	272	10.60	17.60	1.23	0.58	25.66	0.58	9.72
SQB	Lengshuigou	LS02	109.62	33.43	142	2	unpublished data	66.12	1.88	5.55	3.97	143	56	26.40	23.00	2.53	2.55	2.12	2.55	6.18
SQB	Lengshuigou	LS03	109.62	33.43	142	2	unpublished data	65.92	1.85	6.38	3.83	127	261	12.30	25.00	1.23	0.49	21.22	0.49	13.81
SQB	Lengshuigou	LS05	109.62	33.43	142	2	unpublished data	62.44	5.03	5.52	3.42	102	272	7.67	10.00	0.83	0.38	35.46	0.38	8.18

Location	Intrusion	Sample	Longitude	Latitude	Age (Ma)	error	Reference	SiO2(wt.%)	MgO(wt.%)	Na2O(wt.%)	K2O(wt.%)	Rb(ppm)	Sr(ppm)	Y(ppm)	La(ppm)	Yb(ppm)	Rb/Sr(*)	Sr/Y	Rb/Sr(**)	La/Ybn
SQB	Lengshuigou	LS06	109.62	33.43	142	2	unpublished data	59.62	4.32	5.67	6.00	447	114	66.60	51.00	8.10	3.92	1.71	3.92	4.28
SQB	Lengshuigou	LS15	109.62	33.43	142	2	unpublished data	61.77	5.07	5.97	3.24	97	243	8.61	23.00	0.88	0.40	28.22	0.40	17.76
SQB	Lengshuigou	LS16	109.62	33.43	142	2	unpublished data	61.69	5.27	5.64	3.35	99	267	13.00	29.00	1.24	0.37	20.54	0.37	15.89
SQB	Lengshuigou	LS18	109.62	33.43	142	2	unpublished data	64.82	2.35	4.85	3.75	83	768	14.50	27.00	1.57	0.11	52.97	0.11	11.68
SQB	Lengshuigou	LS19	109.62	33.43	142	2	unpublished data	66.51	1.95	4.99	3.38	135	526	9.90	19.00	1.10	0.26	53.13	0.26	11.73
NQB	Cuihuashan	FYK-2	108.85	33.95	227	4	Jiang et al., (2010)[4]	71.28	0.50	4.70	3.55	137	408	9.20	16.70	0.79	0.34	44.35	0.34	14.36
NQB	Cuihuashan	FYK-4	108.85	33.95	227	4	Jiang et al., (2010)[4]	70.76	0.66	4.07	4.19	142	396	12.90	45.10	1.41	0.36	30.70	0.36	21.73
NQB	Qinlingliang	QS2	107.20	34.25	210	2	Wang et al., (2011)[1]	64.80	2.16	4.20	4.32	130	617	18.30	47.40	1.83	0.21	33.72	0.21	17.60
NQB	Qinlingliang	QS2-1	107.20	34.25	210	2	Wang et al., (2011)[1]	65.00	2.17	4.20	4.05	89	588	17.30	43.90	1.75	0.15	33.99	0.15	17.04
NQB	Laojunshan	LS0	107.67	34.15	214	3	Wang et al., (2011)[1]	66.28	2.03	4.34	3.70	91	770	25.90	60.90	2.22	0.12	29.73	0.12	18.64
NQB	Laojunshan	LS1	107.67	34.15	214	3	Wang et al., (2011)[1]	65.00	1.60	5.00	2.40	77	594	15.70	45.80	1.59	0.13	37.83	0.13	19.57
NQB	Laojunshan	LS2	107.67	34.15	214	3	Wang et al., (2011)[1]	65.00	2.00	4.00	4.00	130	564	22.30	49.80	2.28	0.23	25.29	0.23	14.84
NQB	Laojunshan	LS3	107.67	34.15	214	3	Wang et al., (2011)[1]	65.00	2.00	4.15	4.18	119	693	15.00	40.70	1.49	0.17	46.20	0.17	18.56
NQB	Baoji	08BJ17-1	107.22	34.23	212	1	unpublished data	71.48	0.62	3.96	4.62	121	236	25.90	44.80	2.37			0.51	12.84
NQB	Baoji	08BJ12-1	107.22	34.23	212	1	unpublished data	69.73	0.52	5.02	4.50	110	320	12.60	42.20	1.36	0.34	25.40	0.34	21.08
NQB	Baoji	08BJ11-1	107.22	34.23	216	2	unpublished data	68.61	0.75	4.35	4.26	86	417	15.40	41.80	1.65	0.21	27.08	0.21	17.21
NQB	Baoji	08BJ12-2	107.22	34.23	212	1	unpublished data	69.73	0.59	4.16	5.08	101	277	9.29	30.20	0.94	0.36	29.82	0.36	21.83
NQB	Baoji	08BJ13-1	107.22	34.23	212	1	unpublished data	69.62	0.97	3.76	4.51	87	356	17.00	37.30	1.91	0.24	20.94	0.24	13.27
NQB	Baoji	08BJ07-2	107.22	34.23	212	1	unpublished data	57.15	5.37	1.95	7.16	451	1451	22.50	91.20	2.28			0.31	27.17
NQB	Mangling	ML34/IB	110.58	33.85	157	1	Yang et al., (2014)[31]	57.39	3.22	3.86	2.54	66	1321	21.38	49.06	2.09	0.05	61.77	0.05	15.95
NQB	Mangling	ML-35/1B	110.58	33.85	157	1	Yang et al., (2014)[31]	55.19	5.34	3.82	2.65	71	1036	19.09	44.38	1.74	0.07	54.27	0.07	17.33
NQB	Mangling	ML-37/1B	110.58	33.85	157	1	Yang et al., (2014)[31]	59.18	3.82	3.62	3.18	97	859	17.18	49.14	1.57	0.11	50.00	0.11	21.26
NQB	Mangling	ML-38/1B	110.58	33.85	157	1	Yang et al., (2014)[31]	55.22	4.67	3.82	2.90	88	1044	19.21	48.14	1.70	0.08	54.36	0.08	19.24
NQB	Mangling	ML-40/1B	110.58	33.85	157	1	Yang et al., (2014)[31]	58.57	3.59	3.42	2.36	70	761	20.96	35.26	2.07	0.09	36.33	0.09	11.57
NQB	Mangling	09CL257	110.30	33.8	158	2	Qin et al., (2012)[32]	67.31	0.79	3.85	4.84	160	729	16.87	48.66	1.84	0.22	43.20	0.22	17.97
NQB	Mangling	09CL260	110.30	33.8	161	1	Qin et al., (2012)[32]	69.35	1.14	3.22	5.03	125	549	12.21	41.56	1.36	0.23	45.00	0.23	20.76
NQB	Mangling	09CL261	110.30	33.8	161	1	Qin et al., (2012)[32]	70.80	0.52	3.54	5.30	190	327	10.48	35.51	1.19	0.58	31.24	0.58	20.27
NQB	Mangling	09CL263	110.30	33.8	161	1	Qin et al., (2012)[32]	70.53	0.60	3.51	4.72	158	541	15.65	47.60	1.54	0.29	34.56	0.29	21.00
NQB	Mangling	ML-17/1B	110.58	33.85	148	1	Yang et al., (2014)[31]	68.37	0.97	3.85	5.04	152	484	11.52	45.74	1.23	0.31	42.01	0.31	25.26
NQB	Mangling	ML-20/1B	110.58	33.85	148	1	Yang et al., (2014)[31]	67.34	1.01	3.73	4.80	183	526	16.94	64.90	1.83	0.35	31.03	0.35	24.09
NQB	Mangling	ML-21/1B	110.58	33.85	148	1	Yang et al., (2014)[31]	69.72	0.83	3.49	3.24	109	459	13.13	55.90	1.35	0.24	34.94	0.24	28.13
NQB	Mangling	ML-23/1B	110.58	33.85	148	1	Yang et al., (2014)[31]	70.97	1.15	3.79	4.09	120	327	18.34	62.14	2.08			0.37	20.29

Location	Intrusion	Sample	Longitude	Latitude	Age (Ma)	error	Reference	SiO2(wt.%)	MgO(wt.%)	Na2O(wt.%)	K2O(wt.%)	Rb(ppm)	Sr(ppm)	Y(ppm)	La(ppm)	Yb(ppm)	Rb/Sr(*)	Sr/Y	Rb/Sr(**)	La/Ybn
NQB	Mangling	ML-27/1B	110.58	33.85	148	1	Yang et al., (2014)[31]	71.84	0.87	4.22	4.90	190	287	10.17	32.78	1.22	0.66	28.18	0.66	18.25
NQB	Mangling	ML-28/1B	110.58	33.85	148	1	Yang et al., (2014)[31]	70.42	0.85	4.06	4.10	170	403	11.05	45.84	1.19	0.42	36.43	0.42	26.17
NQB	Mangling	ML-29/1B	110.58	33.85	144	1	Yang et al., (2014)[31]	71.53	0.71	3.73	4.22	164	378	10.79	39.40	1.19	0.43	35.03	0.43	22.49
NQB	Muhuguan	MHG43-1	109.50	34.00	149	1	Liu et al., (2014)[33]	68.38	0.58	3.34	6.46	207	562	14.60	38.92	1.31	0.37	38.49	0.37	20.18
NQB	Muhuguan	MHG43-2	109.50	34.00	149	1	Liu et al., (2014)[33]	68.67	0.56	3.49	5.37	163	575	14.60	37.61	1.22	0.28	39.38	0.28	20.94
NQB	Muhuguan	MHG43-4	109.50	34.00	149	1	Liu et al., (2014)[33]	64.95	1.81	3.88	3.58	165	682	17.20	62.24	1.47	0.24	39.65	0.24	28.76
NQB	Muhuguan	MHG45-3	109.50	34.00	149	1	Liu et al., (2014)[33]	64.72	1.36	3.95	3.36	83	797	24.40	57.91	2.12	0.10	32.66	0.10	18.56
NQB	Muhuguan	MHG45-4	109.50	34.00	149	1	Liu et al., (2014)[33]	66.00	1.28	3.70	3.85	88	781	19.80	49.10	1.79	0.11	39.44	0.11	18.63
NQB	Taibai	ZG239	107.75	34.08	153	1	Zhang et al., (2014)[34]	68.88	0.50	4.35	4.57	129	453	16.90	34.20	1.45	0.28	26.80	0.28	16.02
NQB	Taibai	ZG240	107.75	34.08	153	1	Zhang et al., (2014)[34]	69.92	0.53	3.65	4.38	118	588	15.00	49.80	1.21	0.20	39.20		
NQB	Taibai	ZG242	107.75	34.08	153	1	Zhang et al., (2014)[34]	68.49	0.63	3.85	4.01	103	578	24.60	40.60	1.96	0.18	23.50	0.18	14.07
NQB	Taibai	ZG256	107.75	34.08	151	1	Zhang et al., (2014)[34]	69.56	0.61	5.61	2.53	94	452	10.00	28.30	1.04	0.21	45.20	0.21	18.49

Notes: SQB - South Qinling Belt; NQB - North Qinling Belt.

n denotes the value is normalized to chondritic values of McDonough and Sun (1995)[39].

The blank data of Sr/Y and La/Ybn are discarded outliers.

* represents Rb/Sr corresponding to Sr/Y.

** represents Rb/Sr corresponding to La/Ybn.

Table S9. Data subsets used to calculate Moho depth and discarded data subsets from Qinling Orogenic Belt.

Location	Intrusion	Avg. Longitude	Avg. Latitude	Avg. Age(Ma)	std	Avg. La(ppm)	Avg. Rb/Sr(*)	Median Sr/Y	std	Avg. Rb/Sr(**)	Median La/Ybn	std
SQB	Shahewan	109.71	33.76	212.83	1.85	38.87	0.18	45.87	8.59	0.18	19.41	2.61
SQB	Caoping	109.53	33.78	215.25	0.45	33.50	0.26	33.98	4.68	0.22	13.41	3.33
SQB	Caoping	109.50	33.78	221.71	2.14	34.93	0.20	33.27	3.99	0.20	13.04	1.03
SQB	Zhashui	109.25	33.63	225.00	0.00	28.68	0.30	28.61	4.95	0.35	14.53	4.55
SQB	Zhashui	109.24	33.63	200.92	1.38	35.45	0.35	24.11	8.66	0.35	14.82	3.89
SQB	Dongjiangkou	108.95	33.73	218.79	1.51	31.31	0.14			0.14	16.27	3.59
SQB	Dongjiangkou	108.98	33.75	214.00	0.00	24.85	0.14			0.14	15.12	1.82
SQB	Yanzhiba	108.50	33.42	222.00	0.00	24.00	0.17			0.17	22.94	4.03
SQB	Laocheng	108.35	33.50	210.33	0.58	29.00	0.10			0.10	38.49	7.16
SQB	Wulong	108.25	33.50	232.43	0.98	36.16	0.10			0.10	18.24	4.49
SQB	Wulong	108.25	33.50	227.00	0.00	33.84	0.11			0.12	15.56	1.92
SQB	Wulong	108.25	33.50	218.00	0.00	27.45	0.25			0.25	23.66	4.80
SQB	Longcaoping	107.98	33.75	217.00	0.00	35.67	0.11	21.33	3.57	0.11	9.41	1.35
SQB	Longcaoping	107.96	33.67	189.00	0.00	36.43	0.32	24.01	2.63	0.27	15.78	4.47
SQB	Xichahe	107.75	33.47	212.00	0.00	26.27	0.11			0.11	14.36	5.53
SQB	Huayang	107.50	33.57	207.00	0.00	39.73	0.31			0.30	31.56	1.82
SQB	Huayang	107.38	33.55	195.00	1.41	38.95	0.21			0.21	13.90	1.63
SQB	Xiba	107.20	33.67	218.50	0.52	32.54	0.25			0.25	16.69	4.16
SQB	Taoyuanpu	106.83	33.69	216.00	0.00	25.79	0.18			0.18	13.47	4.06
SQB	Liuba	106.93	33.60	211.00	0.00	25.81	0.10			0.10	10.04	0.51
SQB	Huoshaodian	106.93	33.53	215.00	0.00	32.74	0.12	50.99	6.50	0.12	15.44	2.74
SQB	Guangtoushan	106.63	33.36	224.00	0.00	27.35	0.16			0.16	15.25	4.12
SQB	Zhangjiaba	106.34	33.44	230.00	0.00	40.23	0.14	34.74	2.28	0.14	15.25	4.44
SQB	Xinyuan	106.20	33.49	228.57	0.53	23.97	0.12			0.14	7.66	1.77
SQB	Miba	105.83	33.50	211.00	0.00	40.76	0.16	36.63	6.34	0.16	18.30	2.76
SQB	Huangzhuguan	105.65	33.95	214.00	0.00	23.37	0.13	38.15	1.91	0.13	12.15	0.62
SQB	Meiwu	103.32	34.99	242.00	1.15	32.47	0.34	26.99	9.65	0.34	14.44	4.06
SQB	Xiahe	102.72	35.21	245.82	2.09	32.16	0.27	28.97	7.38	0.27	12.61	2.09
SQB	Xiekeng	102.45	35.56	243.20	1.10	21.22	0.19	14.87	3.96	0.19	5.13	2.06
SQB	Maixiu	101.83	35.27	234.00	0.00	17.90	0.34	12.63	2.57	0.30	7.02	1.24
SQB	XY	109.83	33.63	142.50	0.53	34.58	0.14			0.14	17.07	4.32

Location	Intrusion	Avg. Longitude	Avg. Latitude	Avg. Age	std	La	Rb/Sr(*)	Sr/Y	std	Rb/Sr(**)	La/Ybn	std
SQB	XWY	109.63	33.59	147.23	2.28	30.75	0.10			0.10	12.19	2.61
SQB	BCTS	109.60	33.50	144.82	1.25	45.69	0.07			0.10	16.16	3.24
NQB	Cuihuashan	108.85	33.95	227.00	0.00	30.90	0.35	37.52	9.65	0.35	18.04	5.21
NQB	Qinlingliang	107.20	34.25	210.00	0.00	45.65	0.18	33.85	0.19	0.18	17.32	0.39
NQB	Laojunshan	107.67	34.15	214.00	0.00	49.30	0.16	33.78	9.22	0.16	18.60	2.09
NQB	Baoji	107.22	34.23	212.67	1.63	47.92	0.29	26.24	3.72	0.33	19.14	5.53
NQB	Mangling	110.46	33.83	158.44	1.94	44.37	0.19			0.19	19.24	3.14
NQB	Muhuguan	109.50	34.00	149.00	0.00	49.16	0.22	39.38	2.98	0.22	20.18	4.23
NQB	Taibai	107.75	34.08	152.50	0.00	38.23	0.22			0.22	16.02	2.21

SQB	Dongjiangkou	108.95	33.73	218.79	1.51	31.31	0.14	60.81	15.84	0.14	16.27	3.59
SQB	Dongjiangkou	108.98	33.75	214.00	0.00	24.85	0.14	69.36	3.89	0.14	15.12	1.82
SQB	Yanzhiba	108.50	33.42	222.00	0.00	24.00	0.17	79.51	11.85	0.17	22.94	4.03
SQB	Laocheng	108.35	33.50	218.77	1.64	31.31	0.14	90.98	30.27	0.14	24.70	10.37
SQB	Laocheng	108.35	33.50	210.33	0.58	29.00	0.10	146.92	1.89	0.10	38.49	7.16
SQB	Wulong	108.25	33.50	232.43	0.98	36.16	0.10	64.25	3.45	0.10	18.24	4.49
SQB	Wulong	108.25	33.50	227.00	0.00	33.84	0.11	68.26	10.95	0.12	15.56	1.92
SQB	Wulong	108.25	33.50	218.00	0.00	27.45	0.25	68.01	18.87	0.25	23.66	4.80
SQB	Wulong	108.25	33.50	207.35	0.49	33.99	0.14	98.16	27.08	0.14	41.07	14.86
SQB	Xichahe	107.75	33.47	212.00	0.00	26.27	0.11	56.75	15.50	0.11	14.36	5.53
SQB	Huayang	107.37	33.58	211.75	2.63	37.60	0.41	60.81	56.88	0.41	12.06	4.27
SQB	Huayang	107.50	33.57	207.00	0.00	39.73	0.31	42.09	20.96	0.30	31.56	1.82
SQB	Huayang	107.38	33.55	195.00	1.41	38.95	0.21	35.97	10.99	0.21	13.90	1.63
SQB	Xiba	107.20	33.67	218.50	0.52	32.54	0.25	40.95	15.11	0.25	16.69	4.16
SQB	Taoyuanpu	106.83	33.69	216.00	0.00	25.79	0.18	47.13	15.81	0.18	13.47	4.06
SQB	Liuba	106.93	33.60	211.00	0.00	25.81	0.10	47.44	16.76	0.10	10.04	0.51
SQB	Guangtoushan	106.63	33.36	224.00	0.00	27.35	0.16	103.50	51.81	0.16	15.25	4.12
SQB	Guangtoushan	106.68	33.39	218.00	0.00	25.89	0.18	102.70	42.34	0.20	24.15	10.51
SQB	Xinyuan	106.20	33.49	228.57	0.53	23.97	0.12	50.71	16.52	0.14	7.66	1.77
SQB	Mishuling	105.75	34.03	213.80	1.01	30.63	0.41	14.82	1.64	0.41	16.06	2.72
SQB	Wenquan	105.25	34.60	224.27	1.01	41.37	0.67	22.83	8.17	0.61	19.24	4.15
SQB	Wenquan	105.25	34.60	216.73	0.47	31.38	0.83	15.11	3.71	0.83	14.54	4.36

Location	Intrusion	Avg. Longitude	Avg. Latitude	Avg. Age	std	La	Rb/Sr(*)	Sr/Y	std	Rb/Sr(**)	La/Ybn	std
SQB	Luchuba	105.00	34.08	211.00	0.00	33.17	0.50	23.90	1.74	0.50	15.78	3.25
SQB	Zhongchuan	105.04	34.29	219.17	0.39	38.96	0.79	18.56	3.89	0.79	16.24	3.44
SQB	Zhongchuan	105.00	34.33	234.38	1.56	43.29	0.61	17.73	3.73	0.61	18.12	3.82
SQB	Lüjing	104.58	34.41	221.00	0.00	42.87	0.78	17.12	3.15	0.75	19.85	3.32
SQB	Shuangpenxi	102.33	35.56	242.00	0.00	43.87	0.56	9.74	0.13	0.56	10.74	1.74
SQB	Tongren	101.88	35.45	241.00	0.00	31.57	0.80	14.56	2.42	0.80	9.88	3.17
SQB	XY	109.83	33.63	142.50	0.53	34.58	0.14	67.19	24.47	0.14	17.07	4.32
SQB	XWY	109.63	33.59	147.23	2.28	30.75	0.10	62.29	38.98	0.10	12.19	2.61
SQB	BCTS	109.60	33.50	144.82	1.25	45.69	0.07	63.22	6.24	0.10	16.16	3.24
SQB	Lengshuigou	109.62	33.43	142.00	0.00	25.03	0.82	29.95	25.53	0.82	11.71	3.95
NQB	Mangling	110.46	33.83	158.44	1.94	44.37	0.19	45.00	10.33	0.19	19.24	3.14
NQB	Mangling	110.58	33.85	147.43	1.51	49.53	0.40	34.99	4.75	0.40	24.09	3.44
NQB	Taibai	107.75	34.08	152.50	0.00	38.23	0.22	33.00	10.23	0.22	16.02	2.21

Notes: SQB - South Qinling Belt; NQB - North Qinling Belt; Avg. - Average; std - standard deviation.

XY - Xiaguanfang-Yuanzijie; XWY - Xiaohekou-Wagou-Yuanjiagou; BCTS - Baishagou-Chigou-Tudigou-Shuangyuangou.

n denotes the value is normalized to chondritic values of McDonough and Sun (1995)[39].

The subsets with value in red are discarded due to their higher value of Rb/Sr, Sr/Y or std.

* represents Rb/Sr corresponding to Sr/Y.

** represents Rb/Sr corresponding to La/Ybn.

Tabe S10. Data subsets of calculated Moho depth in Qinling Orogenic Belt.

Location	Intrusion	Avg. Longitude	Avg. Latitude	Avg. Age(Ma)	std	Sr/Y	std	Moho(km)(*)	uncertainty	La/Ybn	std	Moho(km)(**)	uncertainty	Avg. Moho(km)	Moho diff.(km)	Avg. uncertainty
SQB	Xiahe	102.72	35.21	245.82	2.09	28.97	7.38	47.65	8.08	12.61	2.09	40.45	10.55	44.05	7.20	9.31
SQB	Xiekeng	102.45	35.56	243.20	1.10	14.87	3.96	38.19	6.29	5.13	2.06	35.20	10.22	36.69	2.99	8.26
SQB	Meiwu	103.32	34.99	242.00	1.15	26.99	9.65	46.33	9.03	14.44	4.06	44.20	12.55	45.26	2.12	10.79
SQB	Maixiu	101.83	35.27	234.00	0.00	12.63	2.57	36.69	5.86	7.02	1.24	41.89	7.14	39.29	5.20	6.50
SQB	Wulong	108.25	33.50	232.43	0.98					18.24	4.49	50.70	12.45	50.70		
SQB	Zhangjiaba	106.34	33.44	230.00	0.00	34.74	2.28	51.52	6.86	15.25	4.44	45.73	12.81	48.63	5.79	9.83
SQB	Xinyuan	106.20	33.49	228.57	0.53					7.66	1.77	43.74	7.93	43.74		
SQB	Wulong	108.25	33.50	227.00	0.00					15.56	1.92	46.28	10.57	46.28		
SQB	Zhashui	109.25	33.63	225.00	0.00	28.61	4.95	47.41	7.18	14.53	4.55	44.38	13.12	45.89	3.03	10.15
SQB	Guangtoushan	106.63	33.36	224.00	0.00					15.25	4.12	45.74	12.46	45.74		
SQB	Yanzhiba	108.50	33.42	222.00	0.00					22.94	4.03	57.07	12.05	57.07		
SQB	Caoping	109.50	33.78	221.71	2.14	33.27	3.99	50.54	7.13	13.04	1.03	41.37	9.82	45.96	9.17	8.47
SQB	Dongjiangkou	108.95	33.73	218.79	1.51					16.27	3.59	47.52	11.82	47.52		
SQB	Xiba	107.20	33.67	218.50	0.52					16.69	4.16	48.23	12.30	48.23		
SQB	Wulong	108.25	33.50	218.00	0.00					23.66	4.80	57.92	12.45	57.92		
SQB	Longcaoping	107.98	33.75	217.00	0.00	21.33	3.57	42.52	6.47	9.41	1.35	48.13	7.25	45.33	5.61	6.86
SQB	Taoyuanpu	106.83	33.69	216.00	0.00					13.47	4.06	42.28	12.78	42.28		
SQB	Caoping	109.53	33.78	215.25	0.45	33.98	4.68	51.01	7.35	13.41	3.33	42.15	11.85	46.58	8.86	9.60
SQB	Huoshaodian	106.93	33.53	215.00	0.00	50.99	6.50	62.43	8.73	15.44	2.74	46.07	11.13	54.25	16.36	9.93
SQB	Dongjiangkou	108.98	33.75	214.00	0.00					15.12	1.82	45.50	10.47	45.50		
SQB	Huangzhuguan	105.65	33.95	214.00	0.00	38.15	1.91	53.81	6.98	12.15	0.62	39.42	9.51	46.62	14.39	8.25
SQB	Shahewan	109.71	33.76	212.83	1.85	45.87	8.59	58.99	9.29	19.41	2.61	52.43	11.21	55.71	6.57	10.25
SQB	Xichahe	107.75	33.47	212.00	0.00					14.36	5.53	44.06	14.50	44.06		
SQB	Liuba	106.93	33.60	211.00	0.00					10.04	0.51	49.50	6.77	49.50		
SQB	Miba	105.83	33.50	211.00	0.00	36.63	6.34	52.79	8.01	18.30	2.76	50.79	11.22	51.79	2.01	9.61
SQB	Laocheng	108.35	33.50	210.33	0.58					38.49	7.16	71.44	13.55	71.44		
SQB	Huayang	107.50	33.57	207.00	0.00					31.56	1.82	65.93	12.04	65.93		
SQB	Zhashui	109.24	33.63	200.92	1.38	24.11	8.66	44.39	8.46	14.82	3.89	44.93	12.27	44.66	0.54	10.36
SQB	Huayang	107.38	33.55	195.00	1.41					13.90	1.63	43.14	10.25	43.14		
SQB	Longcaoping	107.96	33.67	189.00	0.00	24.01	2.63	44.32	6.39	15.78	4.47	46.68	12.75	45.50	2.36	9.57
SQB	XWY	109.63	33.59	147.23	2.28					12.19	2.61	39.50	11.14	39.50		

Location	Intrusion	Avg. Longitude	Avg. Latitude	Avg. Age(Ma)	std	Sr/Y	std	Moho(km)(*)	uncertainty	La/Ybn	std	Moho(km)(**)	uncertainty	Avg. Moho(km)	Moho diff.(km)	Avg. uncertainty
SQB	BCTS	109.60	33.50	144.82	1.25					16.16	3.24	47.34	11.52	47.34		
SQB	XY	109.83	33.63	142.50	0.53					17.07	4.32	48.86	12.41	48.86		
NQB	Cuihuashan	108.85	33.95	227.00	0.00	37.52	9.65	53.39	9.41	18.04	5.21	50.40	13.11	51.90	2.99	11.26
NQB	Laojunshan	107.67	34.15	214.00	0.00	33.78	9.22	50.88	9.07	18.60	2.09	51.24	10.91	51.06	0.36	9.99
NQB	Baoji	107.22	34.23	212.67	1.63	26.24	3.72	45.82	6.73	19.14	5.53	52.04	13.24	48.93	6.23	9.98
NQB	Qinlingliang	107.20	34.25	210.00	0.00	33.85	0.19	50.93	6.64	17.32	0.39	49.26	10.28	50.09	1.67	8.46
NQB	Mangling	110.46	33.83	158.44	1.94					19.24	3.14	52.18	11.47	52.18		
NQB	Taibai	107.75	34.08	152.50	0.00					16.02	2.21	47.10	10.77	47.10		
NQB	Muhuguan	109.50	34.00	149.00	0.00	39.38	2.98	54.64	7.21	20.18	4.23	53.51	12.15	54.08	1.13	9.68

Notes: SQB - South Qinling Belt; NQB - North Qinling Belt; Avg. - Average; std - standard deviation; diff. - difference.

XY - Xiaguanfang-Yuanzijie; XWY - Xiaohekou-Wagou-Yuanjiagou; BCTS - Baishagou-Chigou-Tudigou-Shuangyuangou.

n denotes the value is normalized to chondritic values of McDonough and Sun (1995)[39].

The value in red are calculated based on equation from Profeta et al. (2015)[40].

* represents estimated Moho depth based on Sr/Y value.

** represents estimated Moho depth based on La/Ybn value.

References for supplementary tables:

1. Wang, X. et al. Triassic granitoids of the Qinling orogen, central China: Genetic relationship of enclaves and rapakivi–textured rocks. *Lithos* **126**, 369–387 (2011).
2. Hu, F., Liu, S., Zhang, W., Deng, Z. & Chen, X. A westward propagating slab tear model for Late Triassic Qinling Orogenic Belt geodynamic evolution: Insights from the petrogenesis of the Caoping and Shahewan intrusions, central China. *Lithos* **262**, 486–506 (2016).
3. Gong, H., Zhu, L., Sun, B., Li, B. & Guo, B. Zircon U–Pb ages and Hf isotope characteristics and their geological significance of the Shahewan, Caoping and Zhashui granitic plutons in the South Qinling orogen. *Acta. Petrol. Sinica.* **25**, 248–264 (2009).
4. Jiang, Y.-H., Jin, G.-D., Liao, S.-Y., Zhou, Q. & Zhao, P. Geochemical and Sr–Nd–Hf isotopic constraints on the origin of Late Triassic granitoids from the Qinling orogen, central China: Implications for a continental arc to continent–continent collision. *Lithos* **117**, 183–197 (2010).
5. Hu, F., Liu, S., Ducea, M. N., Zhang, W. & Deng, Z. The geochemical evolution of the granitoid rocks in the South Qinling Belt: Insights from the Dongjiangkou and Zhashui intrusions, central China. *Lithos* **278–281**, 195–214 (2017).
6. Yang, K. et al. LA–ICP–MS Zircon U–Pb geochronology and geological significance of Zhashui granitoids and Dongjiangkou granitoids from Qinling, central China. *Acta. Sci. Nat. Univ. Pek.* **1**, 36–42 (2009).
7. Qin, J.–F. et al. Origin of LateTriassic high–Mg adakitic granitoid rocks from the Dongjiangkou area, Qinling orogen, central China: Implications for subduction of continental crust. *Lithos* **120**, 347–367 (2010).
8. Yang, P. et al. Geochemistry and zircon U–Pb–Hf isotopic systematics of the Ningshan granitoid batholith, middle segment of the south Qinling belt, Central China: Constraints on petrogenesis and geodynamic processes. *J. Asian. Earth. Sci.* **61**, 166–186 (2012).
9. Dong, Y. P. et al. Triassic diorites and granitoids in the Foping area: Constraints on the conversion from subduction to collision in the Qinling orogen, China. *J. Asian.*

Earth. Sci. **47**, 123–142 (2012).

10. Yang, P. T. et al. Ages of the Laocheng Granitoids and Crustal Growth in the South Qinling Tectonic Domain, Central China: Zircon U–Pb and Lu–Hf Isotopic Constraints. *Acta. Geol. Sinica*. **85**, 854–869 (2011).
11. Qin, J.-F., Lai, S.-C. & Li, Y.-F. Multi–stage granitic magmatism during exhumation of subducted continental lithosphere: Evidence from the Wulong pluton, South Qinling. *Gondwana Res.* **24**, 1108–1126 (2013).
12. Qin, J. F., Lai, S. C. & Li, Y. F. Slab breakoff model for the Triassic post–collisional adakitic granitoids in the Qinling Orogen, Central China: Zircon U–Pb Ages, geochemistry, and Sr–Nd–Pb isotopic constraints. *Int. Geol. Rev.* **50**, 1080–1104 (2008).
13. Zhang, F. et al. Xiba Granitic Pluton in the Qinling Orogenic Belt, Central China: Its Petrogenesis and Tectonic Implications. Acta. Geol. Sin. **86**, 1128–1142 (2012).
14. Deng, Z., Liu, S., Zhang, W., Hu, F. & Li, Q. Petrogenesis of the Guangtoushan granitoid suite, central China: Implications for Early Mesozoic geodynamic evolution of the Qinling Orogenic Belt. *Gondwana Res.* **30**, 112–131 (2016).
15. Sun, W. D., Li, S. G., Chen, Y. D. & Li, Y. J. Timing of Synorogenic Granitoids in the South Qinling, Central China: Constraints on the Evolution of the Qinling-Dabie Orogenic Belt. *J. Geol.* **110**, 457–468 (2002).
16. Li, Z. et al. LA–ICP–MS zircon U–Pb dating, geochemistry of the Mishuling intrusion in western Qinling and their tectonic significance. *Acta. Petrol. Sinica.* **29**, 2617–2634 (2013).
17. Wang, T., Ni, P., Sun, W., Zhao, K. & Wang, X. 2011. Zircon U–Pb ages of granites at Changba and Huangzhuguan in western Qinling and implications for source nature. *Chinese. Sci. Bull.* **56**, 659–669.
18. Cai, Y. et al. Hafnium isotope evidence for slab melt contributions in the Central Mexican Volcanic Belt and implications for slab melting in hot and cold slab arcs. *Chem. Geol.* **377**, 45–55 (2014).
19. Zhu, L.-M. et al. Zircon U–Pb ages and geochemistry of the Wenquan Mo–bearing granitioids in West Qinling, China: Constraints on the geodynamic setting for the

newly discovered Wenquan Mo deposit. *Ore. Geol. Rev.* **39**, 46–62 (2011).

20. Kong, J. et al. Petrogenesis of Luchuba and Wuchaba granitoids in western Qinling: geochronological and geochemical evidence. *Miner. Petrol.* 1–22, doi:10.1007/s00710–017–0501–7 (2017).
21. Zhu, L., Zhang, G., Yang, T., Wang, F. & Gong, H. Geochronology, petrogenesis and tectonic implications of the Zhongchuan granitic pluton in the Western Qinling metallogenic belt, China. *Geol. J.* **48**, 310–334 (2013).
22. Wang, S. et al. Zircon U–Pb and geochemistry of Lüjing granitoid pluton in Western Qinling and their significance. *Acta. Petrol. Mineral.* **35**, 33–51 (2016).
23. Luo, B.-J. et al. The Middle Triassic Meiwu Batholith, West Qinling, Central China: Implications for the Evolution of Compositional Diversity in a Composite Batholith. *J. Petrol.* **56**, 1139–1172 (2015).
24. Jin, W., Zhang, Q., He, D. & Jia, X. SHRIMP dating of adakites in western Qinling and their implications. *Acta. Petrol. Sinica.* **21**, 959–966 (2005).
25. Wei, P. et al. Geochemistry, chronology and geological significance of the granitoids in Xiahe, West Qinling. *Acta. Petrol. Sinica.* **29**, 3981–3992 (2013).
26. Luo, B., Zhang, H. & Lü, X. U–Pb zircon dating, geochemical and Sr–Nd–Hf isotopic compositions of Early Indosinian intrusive rocks in West Qinling, central China: petrogenesis and tectonic implications. *Contrib. Mineral. Petrol.* **164**, 551–569 (2012).
27. Li, X. et al. U–Pb zircon geochronology, geochemical and Sr–Nd–Hf isotopic compositions of the Early Indosinian Tongren Pluton in West Qinling: Petrogenesis and geodynamic implications. *J. Asian. Earth. Sci.* **97**, 38–50 (2015).
28. Li, X.-W. et al. Petrology and geochemistry of the early Mesozoic pyroxene andesites in the Maixiu Area, West Qinling, China: Products of subduction or syn–collision? *Lithos* **172–173**, 158–174 (2013).
29. Wu, F. Research on the magmatite and its metallogenic tectonic setting in the Shanyang–Zhashui area, Middle Qinling Orogenic Belt (Chinese Academy of Geological Sciences, Beijing, 2013).
30. Lü, X. et al. LA–ICP–MS zircon U–Pb dating of Taibai pluton in North Qinling

Mountain and its geological significance. *Miner. Deposits.* **33**, 37–52 (2014).

31. Yang, Y. et al. Zircon U–Pb ages, geochemistry and evolution of Mangling pluton in North Qinling Mountains. *Miner. Deposits.* **33**, 14–36 (2014).
32. Qin, H., Wu, C., Wu, X., Lei, M. & Hou, Z. LA–ICP–MS zircon U–Pb ages and implications for tectonic setting of the Mangling granitoid plutons in Qinling Orogen Belt. *Geol. Rev.* **58**, 783–793 (2012).
33. Liu, R., Chen, M., Tian, X., Hu, H. & Yang, D. Geochemical, zircon SIMS U–Pb geochronological and Hf isotopic study on Lantian and Muhuguan plutons in Eastern Qinling, China: petrogenesis and tectonic implications. *Acta. Mineral. Sinica.* **34**, 469–480 (2014).
34. Zhang, Z., Lai, S. & Qin, J. Petrogenesis and its geological significance of the Late Mesozoic syengranite from the Taibai mountain, North Qinling. *Acta. Petrol. Sinica*. **30**, 3242–3254 (2014).
35. Liu, Z., Sun, F., Ma, F. & Zhang, B. Zircon U–Pb ages and petrochemical characteristics of Zhashui granitic plutons in South Qinling Orogen of Shaanxi and their geological significance. *Global. Geol.* **32**, 236–243 (2013).
36. Li, M., Zhang, C., Yuan, K. & Yan, Y. Geochemical characteristics of Miba intrusion in the South Qinling Belt and its geological significance. *J. Northwest Univ.* **34**, 325–330 (2004).
37. Qin, J. et al. Geochemical evidence for origin of magma mixing for the Triassic monzonitic granite and its enclaves at Mishuling in the Qinling orogen (central China). *Lithos* **112**, 259–276 (2009).
38. Zhang, H. et al. Geochemical and Pb–Sr–Nd isotopic compositions of granitoids from western Qinling belt: Constraints on basement nature and tectonic affinity. *Sci. China, Ser. D Earth Sci.* **50**, 184–196 (2007).
39. McDonough, W. F. & Sun, S-S. The composition of the Earth. *Chem. Geol.* **120**, 223–253 (1995).
40. Profeta, L., et al. Quantifying crustal thickness over time in magmatic arcs: *Sci. Rep.* **5**, doi:10.1038/srep17786 (2015).
